# Single Atom Catalysts Based on Earth-Abundant Metals for Energy-Related Applications

**DOI:** 10.1021/acs.chemrev.4c00155

**Published:** 2024-07-05

**Authors:** Štĕpán Kment, Aristides Bakandritsos, Iosif Tantis, Hana Kmentová, Yunpeng Zuo, Olivier Henrotte, Alberto Naldoni, Michal Otyepka, Rajender S. Varma, Radek Zbořil

**Affiliations:** a Regional Centre of Advanced Technologies and Materials, Czech Advanced Technology and Research Institute, Palacký University, Křížkovského 511/8, 779 00 Olomouc, Czech Republic; b Nanotechnology Centre, Centre for Energy and Environmental Technologies, VŠB − Technical University of Ostrava, 17. Listopadu 2172/15, 708 00 Ostrava-Poruba, Czech Republic; c Department of Chemistry and NIS Centre, University of Turin, Turin, Italy 10125; d IT4Innovations, VŠB − Technical University of Ostrava, 17. Listopadu 2172/15, 708 00 Ostrava-Poruba, Czech Republic

## Abstract

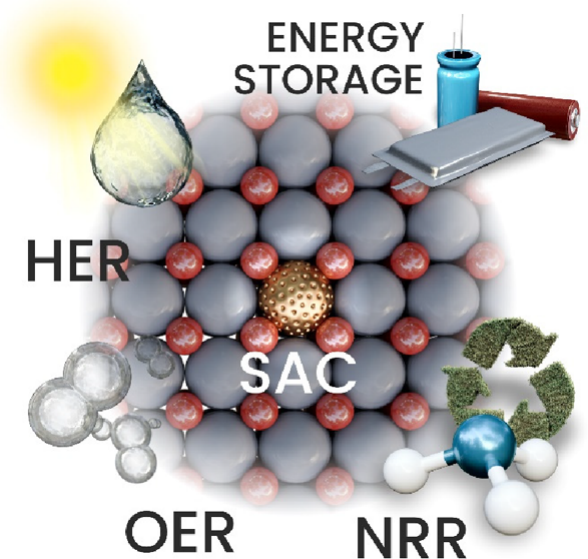

Anthropogenic activities related to population growth, economic development, technological advances, and changes in lifestyle and climate patterns result in a continuous increase in energy consumption. At the same time, the rare metal elements frequently deployed as catalysts in energy related processes are not only costly in view of their low natural abundance, but their availability is often further limited due to geopolitical reasons. Thus, electrochemical energy storage and conversion with earth-abundant metals, mainly in the form of single-atom catalysts (SACs), are highly relevant and timely technologies. In this review the application of earth-abundant SACs in electrochemical energy storage and electrocatalytic conversion of chemicals to fuels or products with high energy content is discussed. The oxygen reduction reaction is also appraised, which is primarily harnessed in fuel cell technologies and metal-air batteries. The coordination, active sites, and mechanistic aspects of transition metal SACs are analyzed for two-electron and four-electron reaction pathways. Further, the electrochemical water splitting with SACs toward green hydrogen fuel is discussed in terms of not only hydrogen evolution reaction but also oxygen evolution reaction. Similarly, the production of ammonia as a clean fuel via electrocatalytic nitrogen reduction reaction is portrayed, highlighting the potential of earth-abundant single metal species.

## 1 INTRODUCTION

The technological evolution of societies has been fueled by their ability to harness energy to boost production, support transportation, and improve the quality of life.^1^ Thus, it is not surprising that global energy consumption grows every year by 1–2%, and predictions foresee a perpetual growth^2^ leading to rapid exploitation of the available natural reserves, with ∼85% being nonrenewable, namely coal, oil, and natural gas. In addition, the processing and utilization of natural resources generate carbon dioxide, SO_*x*_, and NO_*x*_ emissions, resulting in significant environmental pollution. This necessitates the development of new and efficient technologies for energy harvesting from renewable resources.

Renewable energy resources are dominantly intermittent due to natural periodicity and fluctuations of solar radiation and wind. This complicates their establishment as effective alternatives and sustainable energy resources because contemporary major production technologies and the current electricity network require a continuous power supply. Additionally, the past decades have witnessed a portable revolution, i.e., the emerging and enormous global spread of portable devices (laptops, mobile phones, tablets, and medical devices, among many others), which also require mobile energy resources. This trend is crowned by the rapid development of electromobility as sales of electric vehicles doubled in 2021 relative to the previous year,^3^ as well as the production of e-bikes and e-scooters. These developments demand efficient methods for stationary storage of energy harvested from wind and sun for continuous use, in the form of chemical fuels (e.g., hydrogen, alcohols) or rechargeable, safe, and sustainable devices storing electrical energy such as batteries, supercapacitors, or fuel cells for on-site electricity production.

**Figure 1:**
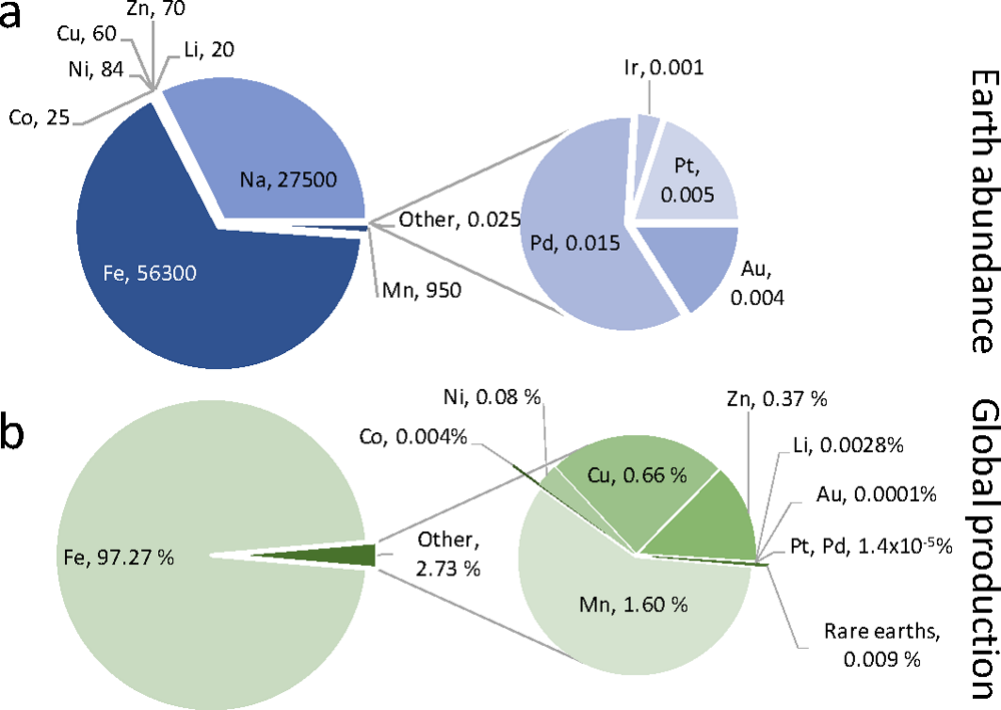
(a) World crust abundance (in ppm) and (b) fraction of the global production (in %) in 2020 of selected metals.^38,39^

Most effective technologies for energy harvesting, conversion, and storage are primarily based on catalytic and electro-, photo-, and photoelectrocatalytic processes demanding catalysts, which often contain rare metal elements. For instance, platinum is by far the best element for electrocatalytic water splitting.^4,5^ The price of such heavy metals is constantly growing because of their low natural abundance in the Earth’s crust (Figure 1) and their availability due to geopolitical reasons, which exemplifies another global concern.^6^ These reasons warrant the development of sustainable and recyclable catalysts, offering full atom economy and urgent and key-enabling technologies for our energy security. Therefore, heterogeneous single atom catalysts (SACs), whereby every single metal atom is fully exposed to the environment and available for interaction with reactants, constitute the Holy Grail in this field.^7^ SACs do not only offer lower prices via reducing the amount of metal to furnish the catalytic reaction, but they also enable newer functions and features. For instance, SACs empower the stabilization of metallic elements in uncommon and mixed oxidation and spin states - offering innovative and more efficient reactivity pathways.^8−10^ Another benefit emanating from this feature is associated with the replacement of rare and precious metals by earth-abundant ones, which can now deliver functions previously unknown in their conventional homogeneous (molecular complexes) and heterogeneous nanoparticulate catalyst forms.^11−13^ Readers may find general information in recent review articles related to single atom engineering applied in conversion/catalysis and energy storage technologies. Previous works involved mostly noble metals with a rather limited discussion on electrochemical energy storage (EES) technologies^14−18^ or were limited to metal-air chemistries,^19^ particularly to Zn-air.^20−23^ Other reviews on SACs focused particularly on Li-S,^24^ and Na-S batteries.^25^ Readers may also find useful the information on energy storage, which covers the EES field more broadly and from the perspective of single atom entrapment methods, up to the mid-2021, though not involving catalytic applications in energy conversion or on earth-abundant SACs.^26^ In the field of energy-related electrocatalytic applications like CO_2_ reduction, electrocatalytic oxygen reduction reactions (ORR), and hydrogen evolution reactions (HER), reviews on single-atom catalysis remain relatively scarce, particularly those delving into non-noble metals based SACs.^27−30^

In line with the ubiquitous presence and pivotal role of molecules and single atoms (SAs) with catalytic action in chemistry and biology, the strategic incorporation of such catalytic species within the field of SACs for energy conversion and storage could open new avenues for chemistries with lower activation energy barriers and promote favorable chemical reactions, thus providing additional and reversible redox sites and lower energy barriers.^31,32^ In this context, SA engineering and SACs can offer maximum atom economy and efficiency, well-defined active sites, and unsaturated coordination spheres for improved interactions with the starting and transient species. SACs are increasingly being explored and employed as electrode components in electrocatalytic and electrochemistry storage applications to enhance the redox kinetics and fine-tune the interactions at the reaction interface, generally boosting the efficiencies.^33,34^ More specifically, SACs can reduce the energy barriers between noncontinuous chemical species due to the low coordinated active sites with high surface energy.^35,36^ Moreover, the strong bonding between matrices and SAs and their well-defined atomic dispersion promotes the charge transfer, thus accelerating the kinetic process of the electrochemical reactions, assuring high activity and conversion efficiency for chemical reactions.^26,37^ In contrast to the nanostructured catalysts, SACs ensure supreme atom utilization, which is extremely valuable in terms of reducing the required metal resources and the environmental burden after the end of life. Thus, SACs refer to hybrid materials where single atomic sites are homogeneously dispersed in a matrix via steady host–guest interactions, combining the advantages of both heterogeneous and homogeneous catalysts.^26,37^ It is indicative that some copper-based SACs, for example, exhibited similar or better catalytic activity than their noble metal counterparts, showcasing the potency of SA engineering.^12,13^

**Figure 2:**
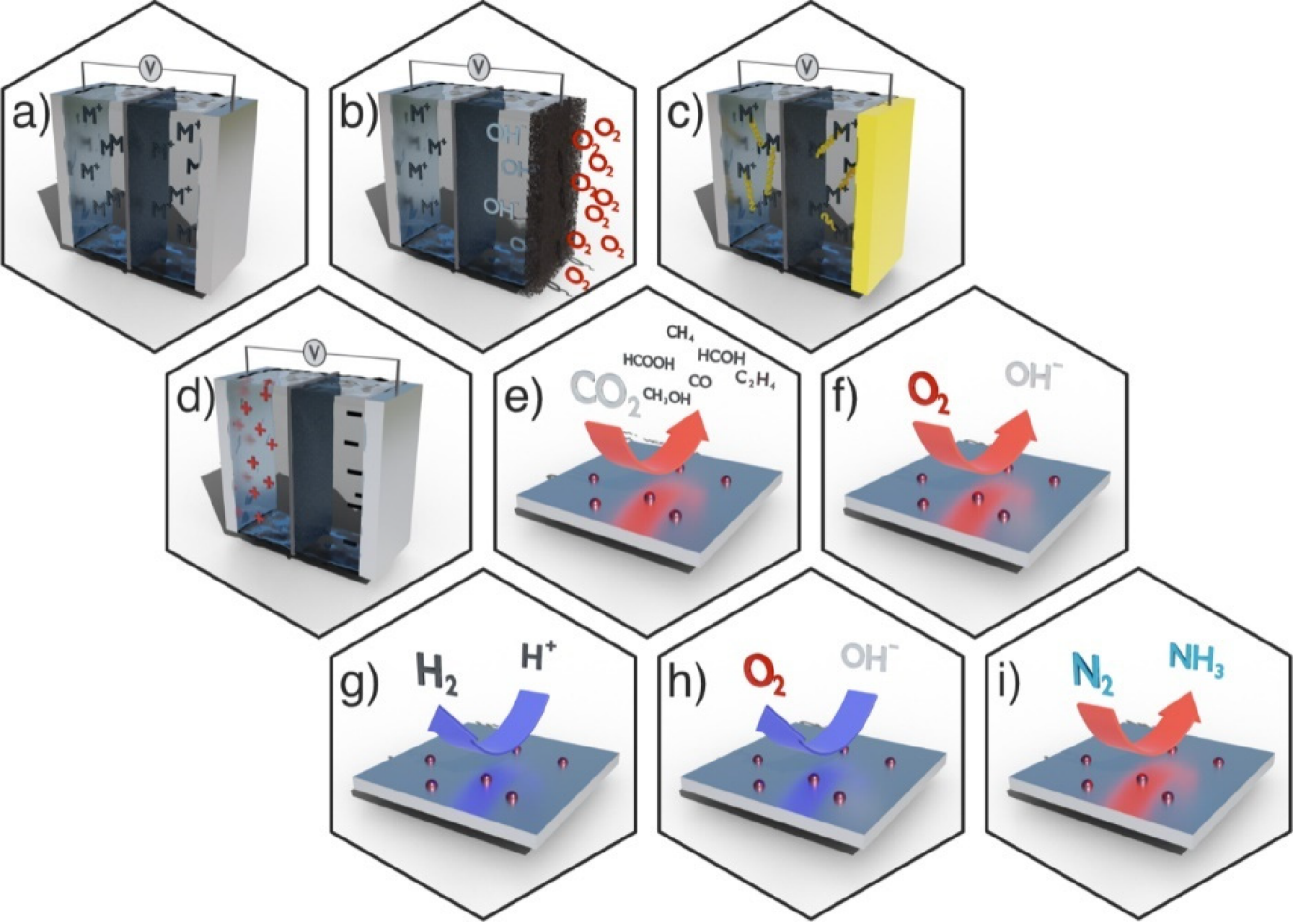
Illustrative representation of various electrochemical processes covered in the review: (a) metal-ion battery, (b) metal/air battery, and (c) metal sulfur battery; (d) supercapacitors and metal-ion supercapacitors; (e) carbon dioxide reduction, (f) oxygen reduction reaction, (g) hydrogen evolution reaction, (h) oxygen evolution reaction, and (i) nitrogen reduction to ammonia.

In this review, we overview the use of SACs based on earth-abundant metals in applications related to energy conversion technologies into chemicals with high energy content and to electrochemical energy storage (Figure 2). Further, we analyze how the different catalysts’ local structures provide distinctive chemical coordination environments, which in turn determine the active sites. Finally, an outlook is provided regarding SACs in terms of scale-up synthesis, high-value products, and high energy efficiency, thus offering a set of criteria that must be fulfilled in transition metal SACs aimed to attain higher performances on a practical scale.

## 2 ELECTROCHEMICAL ENERGY STORAGE WITH EARTH-ABUNDANT SINGLE ATOM CATALYSTS

Electrocatalysts for the ORR, the oxygen evolution reaction (OER), the carbon dioxide reduction reaction (CO2RR), and the carbon dioxide evolution reaction (CO2ER), as well as for the transformation of sulfides/polysulfides and metal oxides formed in metal-air batteries, are at the heart of the next-generation EES chemistries. For example, metal-air, metal–carbon dioxide, metal–sulfur, and pure metal anode batteries promise substantially higher energy contents and charging/discharging rates than current commercial solutions. Appropriately selected SA-engineered materials are also important in surface and interfacial processes (e.g., charge transport, redox cycles, adsorption of charge carriers, and solvent molecules), which, in turn, are critical in supercapacitors or for improving the highly reversible and homogeneous deposition and stripping of pure metal anodes. However, one of the most critical challenges is the sluggishness of the electrochemical transformations at the electrode materials, which leads to an inferior battery performance, including low energy efficiencies due to overpotentials, capacity fading, slow rate capability, and short life.^40^ In order to tackle such challenges, novel electrode materials offering lower energy barriers of conversion kinetics must be developed. Over the past few decades, numerous materials, including noble metals, transition metals, and metal-free carbons, have been explored as electrocatalysts, aiming to achieve high activity, durability, and selectivity for the above-mentioned reactions. Particularly in SACs, the strong interactions between the active metal atomic/ionic centers and the adjacent coordinating atoms may enhance the catalytic activity, selectivity, and durability of the active sites. Remarkable progress has been achieved so far, enabling SACs to outperform conventional metal particle-based catalysts in the race pertaining to the renewable energy landscape.^41,42^ Notably, SACs demonstrate their catalytic activity while employing only a minute fraction of the mass necessary for nanoparticulate catalysts to achieve roughly equivalent activity levels. This characteristic holds particular significance for EES implemented in transportation, autonomous vehicles, and portable devices, where gravimetric and volumetric performances are key features of the devices. In this section, we present the recent progress on EES systems based on earth abundant metal SACs.

### 2.1 SACs in Lithium-Based Batteries

**Figure 3:**
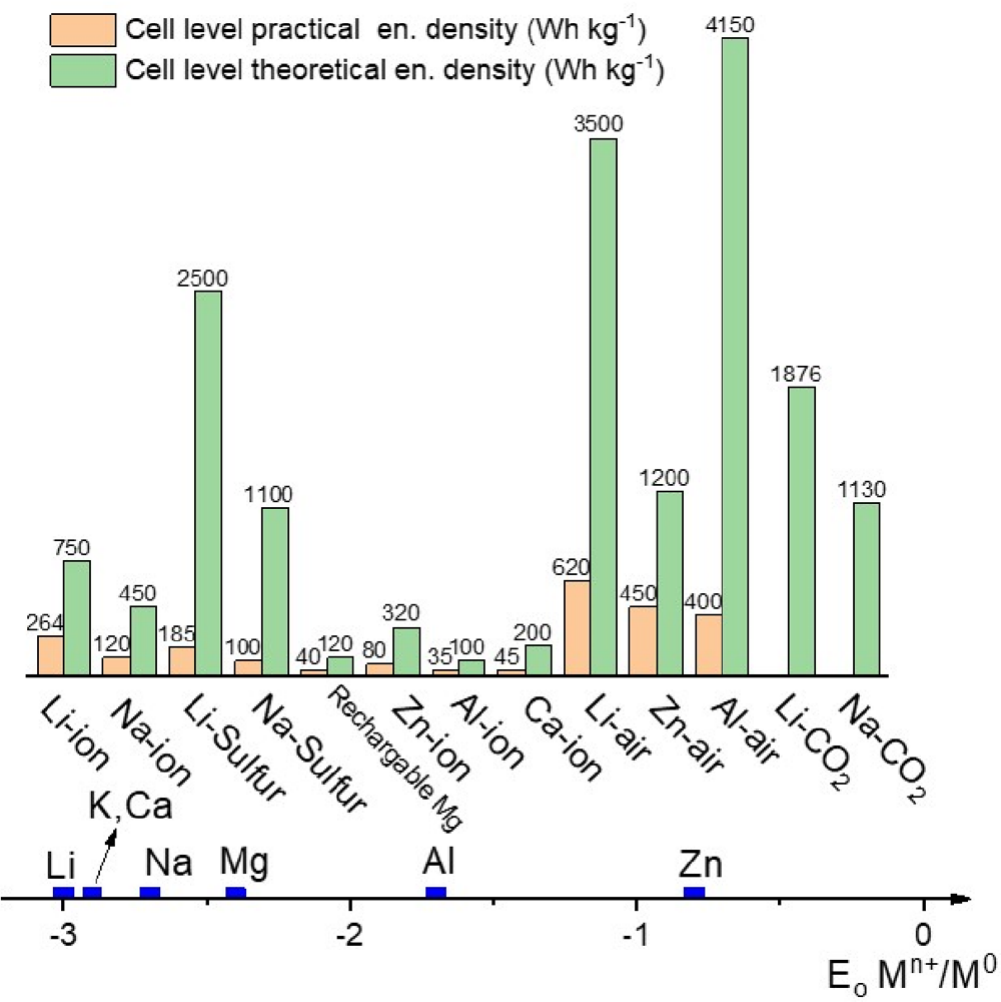
Theoretical and practical technology-specific-gravimetric energy densities and standard reduction potentials of rechargeable battery technologies. Average values are given based on the available cited data and taking into consideration the full-cell systems.^33,49−55^

Lithium-ion batteries (LIBs) have emerged as the primary EES technology within the portable electronics and telecommunications industries, and they have since extended their application into the transportation market. Their widespread adoption in the 1990s has since established them in the forefront of the battery industry.^43,44^ However, LIBs have several shortcomings, including safety, need for costly and critical raw materials, low charging/discharging rates, and insufficient theoretical capacity and energy density, which makes them unsuitable for the future market of electronics, electric vehicles, and large scale energy storage (Figure 3).^45,46^ On this basis, metal SAs could efficiently enhance the electrochemical performance of LIBs.^14^ It is very representative and useful as a benchmark for comparison, although extracted from the noble metal-based SACs, that platinum SAs embedded in carbon anodes in LIBs substantially promoted the formation of Pt-Li_5_ alloy during the charge–discharge process, lowering the lithiation energy and boosting the kinetics. As a result, the Pt SA-decorated carbon material electrode displayed improved electrochemical performances in terms of specific capacity, rate capability, and long-term cyclic stability, retaining a capacity of 846 mAh g^–1^ after 800 cycles at 2 A g^–1^ and 349 mAh g^–1^ after 6000 cycles at a high current density of 5 A g^–1^.^47^ Although the Pt-based SA anode displayed an excellent performance, Pt is among the rarest and most costly elements. Therefore, it is of particular importance to identify earth-abundant elements that may improve the alloy formation with Li atoms. In this direction, Sn (which is at least 3 orders of magnitude more abundant than Pt) SAs were embedded homogeneously into a carbon matrix, where each Sn atom coordinated with two O and two C atoms, forming Sn–O–C and Sn–C bonds.^48^ The Sn SA carbon anode exhibited enhanced lithium storage capability, in comparison with both the carbon alone and the carbon with embedded SnO_2_ nanoparticles, and excellent cyclic stability with a capacity of 478 mAh g^–1^ at 0.05 A g^–1^ after 100 cycles and 281 mAh g^–1^ at 1 A g^–1^ after 7000 cycles.

**Figure 4:**
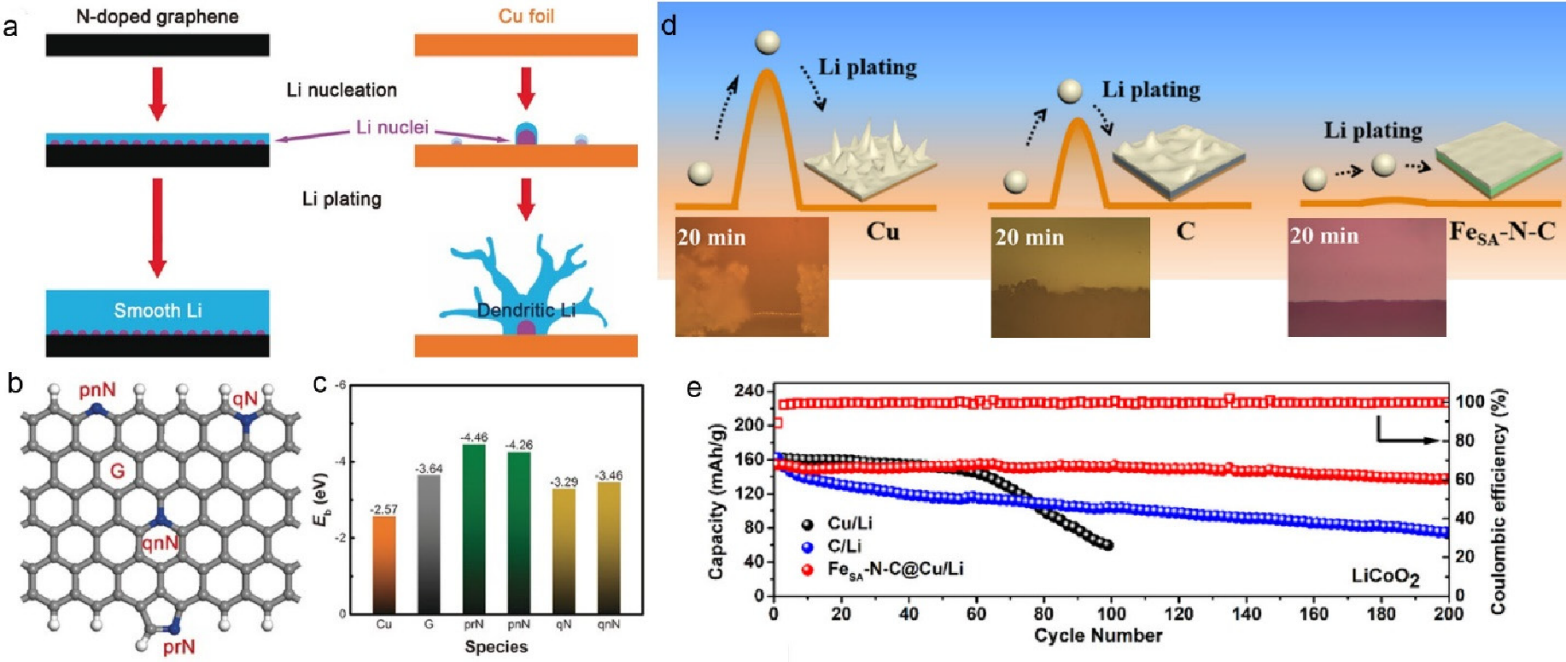
(a) Schematic representation of the Li nucleation and plating process on N-doped graphene coated and uncoated Cu foil electrode. (b) Schematic diagram of N-doped graphene with pyridinic nitrogen (pnN), pyrrolic nitrogen (prN), quaternary nitrogen on the edge (qN), and quaternary nitrogen in the bulk phase (qnN) and (c) their effect on the binding energy of a Li atom with the different functional groups of N-doped graphene, in comparison with pristine Cu and graphene. Reprinted with permission from ref (59). Copyright 2017 Wiley-VCH. (d) Schematic representation of the Li plating process on Cu, C@Cu, and Fe_SA_-N-C@Cu electrodes (the energy barriers represent the overpotential of Li deposition) along with optical microcopy images of dendrite growth on bare Cu, on carbon-coated Cu, and on Fe-SAC coated electrode (Fe_SA_-N-C@Cu). (e) Cycling performances of the full cells with LiCoO_2_ as the cathode and Fe_SA_-N-C/Li (C/Li, Cu/Li) as the anode at 1 C (1C = 274 mA g^–1^). Reprinted with permission from ref (60). Copyright 2019 American Chemical Society.

Apart from lithium-ion batteries, lithium-metal batteries are of broad practical interest in view of their particularly improved theoretical specific capacity of 3860 mAh g^–1^, accompanied by the lowest electrochemical potential of 3.04 V vs standard hydrogen electrode (SHE).^56^ However, plating and stripping of pure Li-metal electrodes face serious reversibility and safety issues due to Li dendrite growth during the charge–discharge process.^57^ To address these limitations, substantial efforts have been made with emphasis on the identification of alternative suitable electrolyte additives or on the addition of interphase layers,^58^ but also on SACs, which have demonstrated the ability to suppress the growth of lithium dendrites, while enhancing the affinity between lithium and electrolyte/active materials. Theoretical and experimental studies have shown that the heterogeneous seeds (e.g., in the form of nitrogen doping) introduced into the carbon matrix serve as lithiophilic sites, tailoring the surface chemistry, reducing the nucleation overpotential, and increasing the binding energy of Li on the electrode surface (Figure 4a–c).^59^ Homogeneously distributed metal SAs also appear to offer great opportunities in this area, since accumulated data demonstrate that they can guide the lithium nucleation and avoid the uncontrolled growth of dendrites. For instance, Yan et al. used Fe SAs in an N-doped carbon matrix (Fe_SA_-N-C) as lithiophilic sites to minimize the Li nucleation overpotential, which is used to quantitatively assess the degree of affinity of the host species on the electrode’s surface.^60^ Both experiments and molecular dynamic simulations showed that the combination of Fe SAs and N-doping of the carbon matrix led to the uniform deposition of lithium on the modified electrode surface, restricting the lithium dendrite growth (Figure 4d), as shown in the optical microscope images after 20 min of plating (Figure 4d, inset). Therefore, the Fe_SA_-N-C electrode exhibited a significantly lower overpotential (0.8 mV) in comparison to the same material but without the Fe SAs (18.6 mV). As a result, the Fe_SA_-N-C on Cu current collector versus LiCoO_2_ as the cathode achieved a significantly improved Coulombic efficiency of 98.8% for 200 cycles in comparison to the case of cycling of metallic lithium on uncoated or on carbon-coated Cu foil (Figure 4e). According to the findings, SA engineered materials can serve as catalysts to boost the utilization of lithium metal by facilitating the stripping and electrodeposition processes. Additionally, they can effectively prevent the growth of Li dendrites, thereby reducing the risk of safety concerns associated with short-circuiting caused by membrane piercing.

**Figure 5:**
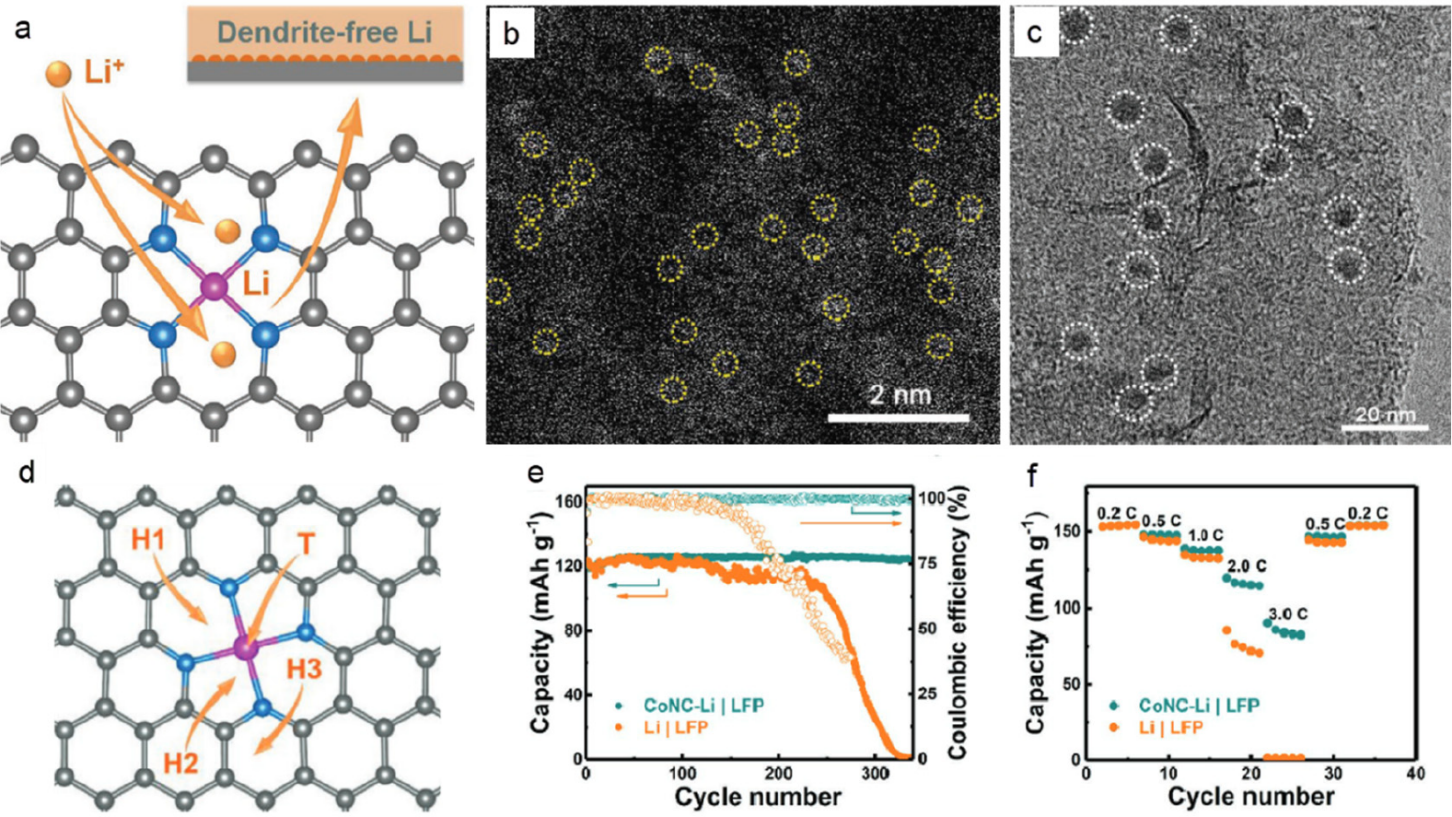
(a) Schematic diagram of CoNC material and preferential Li nucleation sites. The carbon, nitrogen, and cobalt are marked with gray, blue, and dark pink, respectively. (b) HAADF-STEM image of CoNC materials and (c) TEM image of Li nucleation sites on CoNC at 0.1 mA cm^–2^ for 5 min. (d) Li adsorption sites on CoNC, (e) long-term cycling at 1.0 C (1.0 C = 0.170 A g^–1^), and (f) rate capability performance of CoNC-Li|LFP and Li|LFP full cells and. Reprinted with permission from ref (61). Copyright 2019 Wiley-VCH.

Recently, the synthesis of atomically dispersed CoN_*x*_-doped graphene (CoNC) with 0.40 wt % Co was also reported, to achieve dendrite-free lithium deposition.^61^ More specifically, N heteroatoms were coordinated to Co atoms to form Co-N_*x*_-C moieties in conductive CoNC hosts. The strong Co–N interaction contributes to charge transfer from metal dopants to the carbon matrix, and the higher electronegativity of the CoN_*x*_ center in the CoNC matrix ascribes them to stronger affinity for Li-ions (Figure 5a). The atomically distributed Co atoms formed uniform lithium nucleation sites after 5 min of deposition on carbon supports at 0.1 mA cm^–2^, which are clearly shown in high-angle annular dark-field scanning transmission electron microscopy (HAADF-STEM) images (Figure 5b, c). The combination of Co SAs and N dopants in the graphene framework can tune the local electronic structure and facilitate the adsorption of lithium ions and the subsequent nucleation process. First-principle calculations were performed to investigate the lithiophilicity of CoNC materials wherein Li could be strongly absorbed on one top Co atom (site T) and three hollow sites (H1, H2, and H3, Figure 5d). H1 site had the largest binding energy (−1.58 eV) for Li, but all sites showed larger binding energies than that of N sites in NG without Co (−0.86 eV). In addition, it was found that Li interacted with N and Co, simultaneously highlighting the synergy of N and Co for the enhanced lithiophilicity of the CoNC centers. As a result, CoNC anodes in full cells with a lithium iron phosphate (LFP) cathode retained 98.4% of the initial capacity with a CE of 99.9% after 340 cycles, while the capacity retention in a routine Li|LFP cell was lower than 90.0% after 150 cycles, with an average CE of 98.4%. Overall, the results suggest that the CoNC coating on the copper current collector exerts a lithiophilic action, maintaining a stable SEI and a high utilization of Li metal upon the long and challenging cycling. Therefore, the CoNC anode could mitigate the unsatisfactory performance of conventional Li|LFP full cells which is primarily attributed to the depletion of Li metal in the anode, due to the sluggish and highly irregular plating/stripping process. The CoNC-Li|LFP cell also delivered a better rate capability (Figure 5e, f), which was more pronounced at high rates of 2.0 and 3.0 C. A capacity of ∼115 and ∼85 mAh g^–1^ was obtained, respectively, which is much higher than that of routine Li|LFP cells (∼75 and ∼1 mAh g^–1^, respectively). These results convincingly demonstrate a stable and uniform deposition behavior in the CoNC host, which can efficiently increase the reversibility of Li metal anodes upon cycling. Importantly, dendrite formation is not only detrimental for the performance of the device, but also for the safe operation, inducing huge fire risks. It is indicative that in April 2022 two fires involving electric buses were recorded in central Paris, where one vehicle was destroyed. As a safety precaution, the public transport provider decided to temporarily suspend 149 electric buses belonging to the same series.^62^ Automakers are also forced to withdraw some electric vehicle models due to fires, sudden losses of power, and failures to start. Some car companies, for example, spent $800 million to recall a specific model following several reports on battery fires.^63^ Amid several instances documented in the literature,^64^ a fire incident involving an electric vehicle occurred during the charging process, stemming from an error in the onboard charging system. This gave rise to a conflagration that subsequently spread throughout the vehicle, including the battery pack. Upon ignition, the battery pack started to eject sparks and jet flames. The battery pack of the same car model caught fire after collision with a metallic object which penetrated the battery pack. These incidents are characteristic examples of electrical vehicle fires due to battery failure among a series of at least 10 similar accidents since 2019.^65^ Over the past two decades, there have been several reports on fires in portable devices that use LIBs. These have led to the recall of over 9.6 million LIBs from notebooks of prominent computer manufacturers at an estimated direct cost of $360 million. Additionally, 2.5 million smartphones were recalled, with an estimated direct cost of $5.3 billion ($17 billion including the loss of profit).^66^ In China, 31 LIB fires are recorded every year, with the most common cause being sudden ignition (36.9%), followed by charging (26.2%). The USA Federal Aviation Administration has recorded 252 air and airport fire incidents involving LIBs in cargo or baggage since 2006. The USA Consumer Product Safety Commission reported 25,000 fire incidents in more than 400 consumer products between 2012 and 2017.^66^ According to calculations from car insurance companies, fires from hybrid vehicles account for 3.4%, from gas vehicles for 1.5%, and from fully electric vehicles for 0.25%.^67^

The application of earth-abundant SAs in both Li-ion and Li metal batteries is still quite limited, with most of the recent works being focused on Fe-N-C and Co-N-C systems. The experimental data show that the homogeneously distributed metal SAs display high Li utilization and restriction of dendrite growth compared to nanoparticulate counterparts. Moreover, the Li-metal-N interactions contribute to an improved charge transfer of ions and electrons to the carbon matrix, enhancing to the electrode’s conductivity, rate capability, and overall energy efficiency during charging discharging. However, to maintain the high activity of the SAs, it is necessary to develop synthetic strategies that prevent the agglomeration of metal atoms.^68^ As synthesis methodologies advance, it is becoming increasingly feasible to produce SACs on a large scale and loadings, while maintaining their atomic dispersion for securing full active site availability. These developments could have significant practical applications in the field of batteries, further driving technological and commercial innovations.

### 2.2 SACs in Metal-Air Batteries

LIBs involve several critical raw materials (CRMs) both in the anode and in the cathode.^69^ Particularly, the anode is primarily based on natural graphite (which is in the list of CRMs), while lithium itself is also included in the list and the prices are increasing alarmingly with a 5-fold jump during the last year. Regarding the cathodes, the current dominant electrodes are based on nickel, cobalt manganese oxides, where both nickel and cobalt are CRMs. Recent advances in non-noble-metal-based catalyst development have led to comparable catalytic activity and superior fuel tolerance compared to benchmark catalysts, e.g. Pt (∼0.45 V for ORR) and RuO_2_^70^ (∼0.42 V for OER). Carbon-based nanomaterials are of particular interest due to their favorable electrical conductivity, reasonable cost, unique molecular structures with large surface areas, and versatile electronic and microstructural properties. However, pristine carbon is not suitable for practical cathode catalyst application, as it is unreactive and inefficient. Therefore, the research has focused on introducing active sites in carbon-based catalysts through various methods such as doping (single, dual-, and multidoping), defect engineering, and hybridization.^71,72^ With the increasing uncertainty in the global supply chains and with the widening energy crisis, electrochemical energy storage has become one of the key-enabling technologies for a sustainable future, where novel battery chemistries and hybrid supercapacitors will play a central role for the exploitation of renewable, but intermittent, energy resources.^73^ Among them, metal-air batteries are electrochemical cells that may employ a pure metal anode and an external cathode working in ambient air, typically with an aqueous or aprotic electrolyte.^74^ Their theoretical energy density is substantially higher than that of LIBs, making them a promising candidate toward the next-generation EES technology for electric vehicles or grid energy storage (Figure 3). The utilization of air for the reaction at the cathode, operating as a fuel without having to be stored in the device, is particularly attractive for smart and lightweight wearable electronics.^52,75,76^ Several (Li, Si, Zn, Al, Mg)-air cell chemistries are currently being considered,^77^ with theoretical energy densities much higher than those of the current intercalation chemistry LIBs.^78^

#### 2.2.1 Lithium-Oxygen Batteries

Among them, lithium-oxygen batteries have garnered considerable attention as a future EES technology offering one of the highest energy densities,^79^ along with the aluminum–oxygen ones (Figure 3). A Li-O_2_ battery consists of a Li-metal anode and a porous cathode. During the discharge process for the ORR, Li metal atoms are oxidized at the anode to Li^+^, releasing electrons that travel through an external circuit to the cathode. Oxygen molecules are reduced at the cathode (ORR) to form superoxide (O_2_^–^), which then reacts with lithium ions to form lithium peroxide (Li_2_O_2_). In the course of charging, the formed Li_2_O_2_ is oxidized at the cathode and converted back to Li and the OER takes place, forming H^+^ and O_2_.^54^ However, challenging problems have so far limited its commercial viability. These pertain to the high overpotential of the OER (oxidation of Li_2_O_2_) and inferior operation stability, originating from the insoluble (blocking the pores in the cathode) and insulating discharge product of Li_2_O_2_, as well as from decomposition problems of the cathode and the electrolyte due to the high reactivity of peroxide and superoxide.^80^ The sluggish ORR/OER reaction kinetics ought to be enhanced and the overpotentials could be ameliorated by potent electrocatalysts to improve the reaction rates, the electrochemical efficiencies, yields and selectivity, and subsequently the cell performance.^81^ Therefore, the identification and development of highly active and nonprecious metal catalysts for Li–O_2_ batteries, along with homogeneous Li plating and stripping, are crucial toward their commercialization.

To date, platinum, ruthenium, and iridium oxides have been generally regarded as the benchmark electrocatalysts for the ORR and the OER. For instance, a nano-Li_2_O preloaded cathode composed of catalytic Ir nanoparticles and reduced graphene oxide (rGO) substrate provided a flat 2.78 V output vs Li/Li^+^ with a stable reversible capacity of 400 mAh g^–1^ (based on the full cathode mass). This performance sustained over 2,000 cycles with a Coulombic efficiency of up to 99.5%, as confirmed by *in situ* spectroscopic characterizations and chemical quantifications.^82^ However, the insufficient catalytic bifunctionality, scarcity, poor stability, and, importantly, the high cost of the precious metal-based catalysts hinder their practical use.^83^ In order to employ less rare yet electroactive metals, the research has also focused on exploiting molybdenum-based 2D compounds.^84,85^ Molybdenum nitrides displayed high capacities and low overpotentials, primarily due to their superior electronic conductivity, and were able to run for 70 cycles, keeping a 1000 mAh g^–1^ capacity.^85^ Molybdenum sulfide metallic nanosheets (1T-MoS_2_) hybridized with carbon nanotubes also exhibited a high reversible capacity of 500 mAh g^–1^ at a current density of 200 mA g^–1^ for around 100 cycles^86^ or, in another example, of around 400 mAh g^–1^ for 350 cycles.^87^ In another interesting work, MoS_2_ nanosheets as a cathode, with a lithium-carbonate protected anode in an ionic liquid dimethyl sulfoxide electrolyte, consistently delivered 500 mAh g^–1^ for at least 700 cycles at a particularly high rate of 0.5 Ag^1–^. The Li-O_2_ battery operated with the same performance even under simulated-air conditions, i.e. containing both humidity and CO_2_. In 2012, a study on atomically dispersed Fe/N/C SACs as a cathode in a tetra(ethylene glycol) dimethyl ether-based electrolyte^88^ showed substantial advantages over the benchmark systems used until then (α-MnO_2_ on carbon^89^). The Fe/N/C cathode SAC performed the OER during charging at much lower voltage with reduced overpotential during both charging and discharging, thus motivating further research in studying non-noble metal catalysts and particularly SACs for such applications.

**Figure 6:**
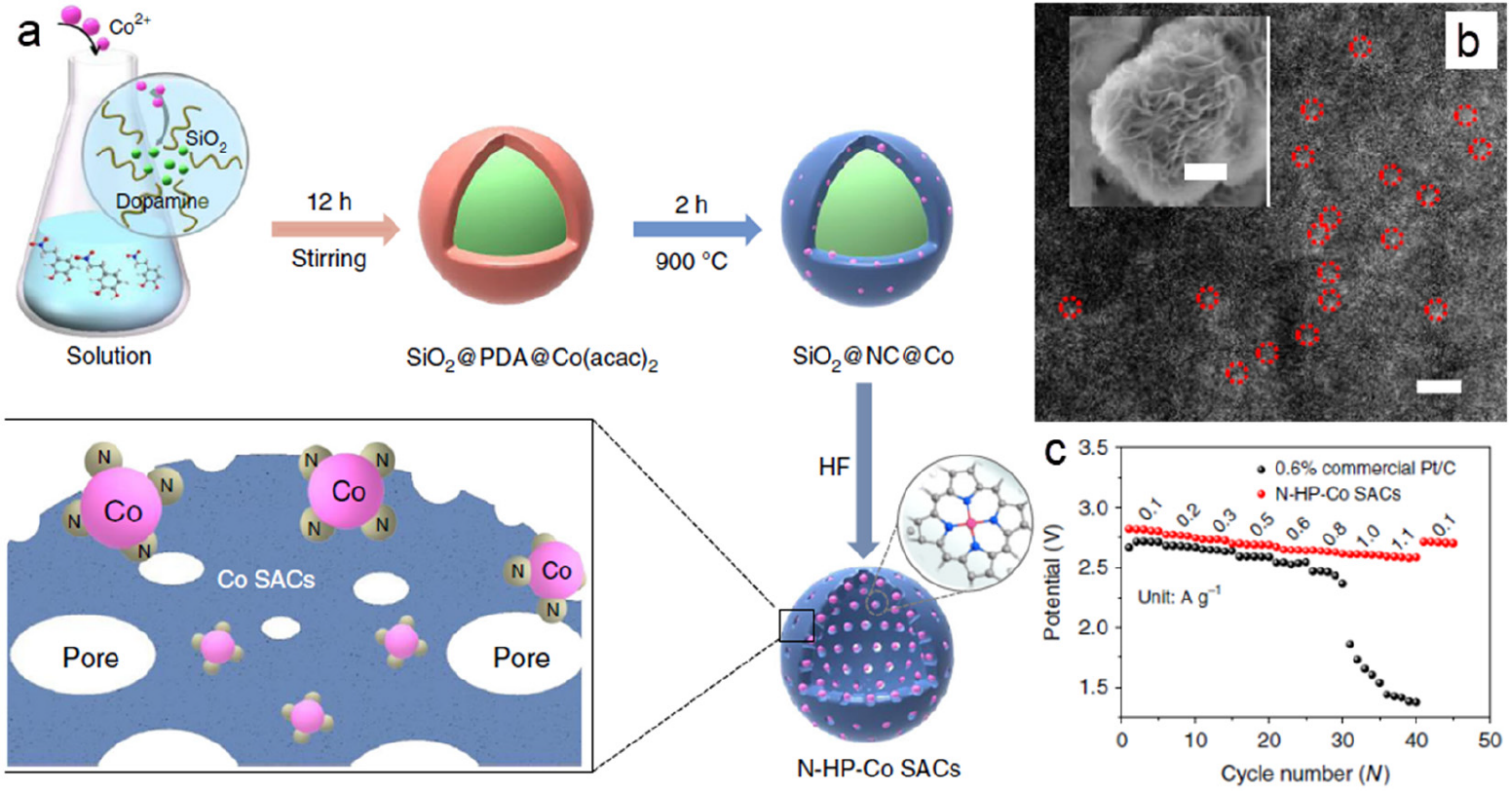
(a) Schematic illustration showing the synthesis of N-HP-Co SACs. (b) HAADF-STEM image of N-HP-Co SACs, 2 nm in, and inset FESEM images of the discharged N-HP-Co SACs cathodes. (c) Full range rate performances of Li-O_2_ batteries at different current densities. Reprinted with permission from ref (90). Copyright 2020 Springer Nature.

With this perspective, earth-abundant SACs have demonstrated improved performance in Li-O_2_ batteries even compared to standard noble metal systems. In an indicative study, a hollow N-doped carbon sphere architecture, with isolated single Co sites (N-HP-Co SACs), was proposed to promote both the ORR and OER reactions and the fast charge and mass transfer of electrons, electrolytes, and O_2_ via their hollow structure.^90^ The synthetic procedure included the polymerization of cobalt complexes mixed with dopamine monomers in spherical silica templates (Figure 6a). Afterward, the product was obtained by pyrolysis to convert the coated poly dopamine into carbon, followed by etching of the silica core with HF. The resulting nanospheres with uniform diameters of ∼400 nm were homogeneously decorated with Co SAs, as confirmed by HAADF-STEM (Figure 6b). The ORR activity of N-HP-Co SACs was studied as the cathode showing a low discharge voltage (0.145 V); the cathode demonstrated a higher onset potential and larger peak current density in comparison to a commercial Pt/C catalyst-loaded equivalent, suggesting an enhanced activity for ORR. Rate performance investigations (Figure 6c) also revealed that the discharge voltage plateau was higher than that of Pt/C catalyst at all current densities. Moreover, the morphology of Li_2_O_2_ on the discharged cathodes was studied *in situ* with scanning electron microscopy (SEM), showing nanosheets grown uniformly on the wall of the porous carbon spheres (Figure 6b, inset), which directly signified the strong interaction of the metal atoms with the supports and the dynamic structural transformation during the discharge process. Benefiting from these merits, Li-O_2_ batteries with these N-HP-Co SACs exhibited relatively low overpotential, high-rate capability, long cycle life (261 cycles at a current density of 0.1 A g^–1^ with a cut off capacity of 1000 mAh g^–1^), and a high discharge capacity (∼14,777 mAh g^–1^ at a current density of 0.1 A g^–1^). The N-HP-Co SAC cathode appears to exhibit improved performance not only in comparison to the nanoparticulate Pt/C catalyst, but even compared to a Pt-SAC cathode with a cutoff capacity of 600 mAh g^–1^ at 0.1 A g^–1^ and reported stability at least up to 100 cycles.

Wang et al. developed an environmentally friendly method to synthesize isolated cobalt atoms embedded in ultrathin and nitrogen-rich carbon as a dual-catalyst for lithium–oxygen batteries.^91^ In particular, the authors developed a green gas-migration-trapping strategy to synthesize desirable Co SAs embedded in ultrathin a Zn-hexamine complex-derived nitrogen-doped carbon matrix (Co-SAs/N-C) as a bifunctional-catalyst for lithium–oxygen batteries. Benefiting from the advantages of both 2D metal organic frameworks (MOFs) and framework (MOF) precursor and of the uniformly isolated dispersion of atomic metal sites, the well-defined Co-SAs/N-C catalyst could provide low-impedance charge transfer pathways and offer large specific surface area for Li_2_O_2_ accommodation. In the lithium-oxygen battery, the Co-SAs/N-C electrode affords remarkably decreased charge/discharge polarization (0.40 V) and long-term cyclability (260 cycles at 0.400 A g^–1^) and high discharge capacity (11098 mAh g^–1^ at 1 A g^–1^).

A universal synthesis strategy for SACs for different metal atoms (Ti, V, Cr, Mn, Fe, Ni, Cu, Zn) immobilized on the surface of Co_3_O_4_ nanosheet arrays grown on a carbon cloth was also reported, aiming at comparing the effect of the different transition metal SAs under the same testing conditions as cathodes for Li-O_2_ batteries.^92^ The results revealed that not all SACs showed enhanced catalytic activity, and some showed decreased catalytic activity in comparison to the pristine Co_3_O_4_ nanosheets. The nickel-doped Co_3_O_4_ displayed the best performance, while the V-doped the worst. The differences in the catalytic activity of SACs with various metal atoms were evaluated by the density functional theory calculations. By combining experimental results, it was concluded that the different SAs mainly affected the energy barriers of the reaction paths on the active sites and the interaction (adsorption energy and electron transfer) between the doped metal atoms and the key intermediate reactants. The Ni-Co_3_O_4_ SAC displayed stability for 128 cycles at a current density of 0.2 A g^–1^ and cutoff capacity of 1000 mAh g^–1^.

#### 2.2.2 Zn-Air Batteries (ZABs)

Apart from the Li-O_2_ batteries, (ZABs also exhibit several advantages, chiefly due to the low cost of zinc, its earth abundance, and safer chemistry in comparison to lithium and other analogues.^93^ Although their energy density is inferior to that of Li-air batteries, it is still significantly higher than that of LIBs (Figure 3), which makes them quite attractive, particularly in synergy with their sustainable and critical raw material-free components. In the various applications of EES devices, safety and weight play a critical role, whereby ZABs are advantageous, since zinc metal is substantially more stable than metals like Li, Na, K, and Mg in the presence of humidity and oxygen, and the cathode is lightweight due to the utilization of oxygen from air as redox component. Furthermore, ZABs can operate both with aqueous or solid electrolytes.^94^ Briefly, a ZAB cell comprises a Zn metal anode while O_2_ acts as the active material at the positive electrode, an alkaline electrolyte, and a separator. During discharge, the oxidation of Zn atoms to Zn^2+^ ions takes place, and the electrons travel from the anode to the air positive electrode. There, O_2_ reduction into OH^–^ occurs at the cathode via the ORR route (O_2_ + 2H_2_O + 4e^–^ → 4OH^–^). The OH^–^ ions combine with Zn^2+^ ions, forming Zn(OH)_4_^2–^, which decomposes to ZnO under supersaturated conditions.^95^ During the charging process, the O^2–^ species must be oxidized back to molecular oxygen, storing chemical energy through the OER.^96^ This process requires electrical reversal of the reaction, using, ideally, a bifunctional catalyst at the air cathode to reduce oxygen during ORR and to oxidize and liberate oxygen upon the discharge reaction during OER, which is especially challenging. It is certainly more viable to offer charge and discharge functions using two unifunctional electrode materials (electrocatalysts); however, this increases the cell size, the weight, and complexity. Thus, bifunctional electrocatalysts to effectively perform both of the reactions are crucial for advancing metal-air battery technologies.^83^ Additional bottlenecks for the effective operation of ZABs include the low stability/reversibility of the processes and the low energy conversion efficiency, since the kinetics of the ORR/OER are relatively slow and high overpotentials are needed to drive the reactions even at moderate rates.^97^

ZABs can also operate in organic electrolytes and perhaps even more effectively with respect to aqueous electrolytes.^98^ A study in 2021 showed that when using a hydrophobic electrolyte, water is excluded from the cathode’s surface, which makes the OER more effective. By eliminating water, instead of the four-electron reduction required in aqueous environment forming Zn(OH)_4_^2–^, the zinc-O_2_/zinc peroxide (ZnO_2_) chemistry dominates that proceeds through a 2e^–^/O_2_ process, enabling more reversible and faster redox reactions.^93^ The nonaqueous ZAB not only tolerates stable operation in ambient air (a common problem for aqueous ZABs due to CO_2_ parasitic reactions) but also exhibits substantially better reversibility than its alkaline counterpart. Although the energy consumed during charging (OER) is still significantly higher than the energy released during discharging (ORR), the process is beneficial for storing energy from renewable resources. Inspired by the catalytic properties of SAs, recent research efforts have targeted SACs as viable and potentially more effective alternatives to noble metal-based catalysts (e.g., Pt) and metal oxides (e.g., RuO_2_ and MnO_2_) traditionally used for ORR and OER, respectively.^83^ In this scope, the potential application of utilizing SACs in ORR and OER catalysis at the air electrode cathode of rechargeable ZABs is enormous.^7^

**Figure 7:**
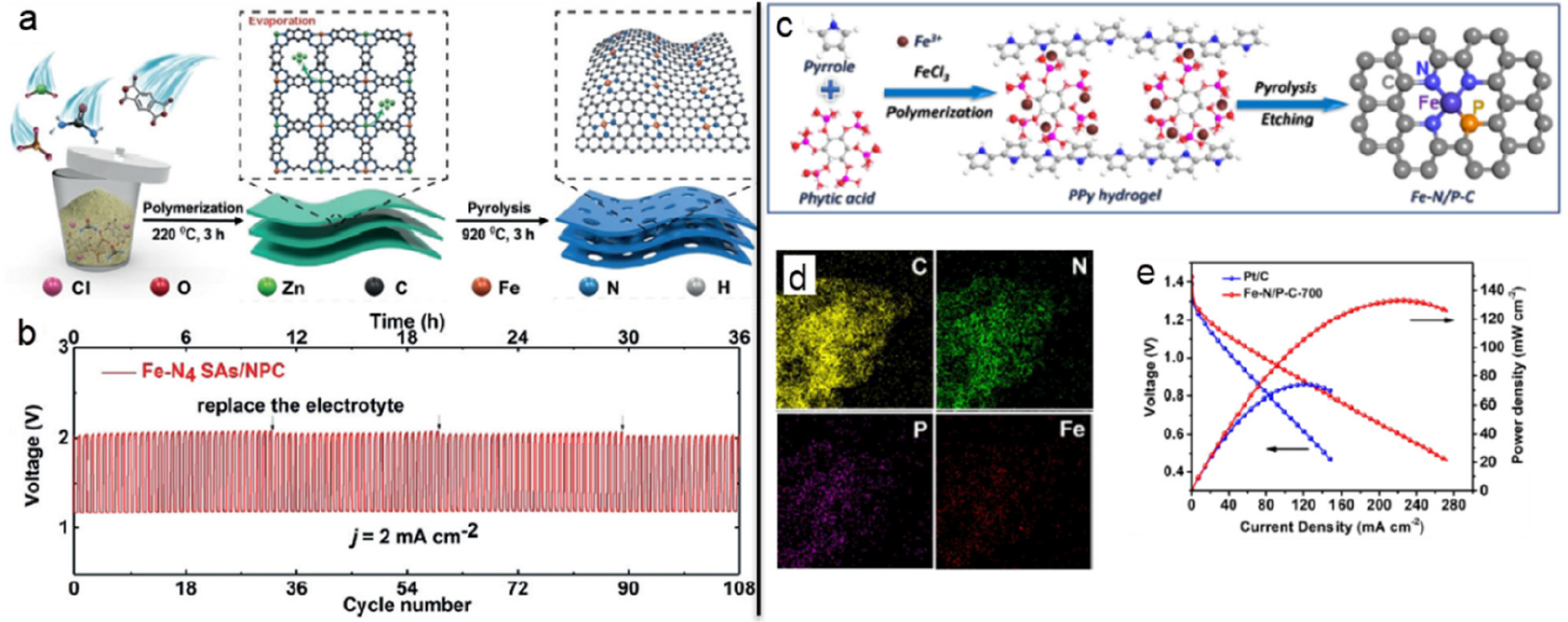
(a) Schematic illustration of the Fe-N_4_ SAs/NPC material synthesis and (b) its charge–discharge cycling performance. Reprinted with permission from ref (110). Copyright 2018 Wiley-VCH. (c) Schematic of the synthesis process of the Fe-N/P-C catalyst. (d) EDS elemental mapping of Fe-N/P-C-700 and (e) polarization and power density plots of the Zn-air batteries equipped with Fe-N/P-C-700 and Pt/C catalysts. Reprinted with permission from ref (113). Copyright 2020 American Chemical Society.

Noncritical metal-ion SACs could transform into the next generation sustainable alternatives to Pt-based electrocatalysts for ORR catalysis. Among the reported noncritical element electrocatalysts, Fe-based catalysts are by far the most extensively studied, owing to their excellent ORR activity in both alkaline and acidic electrolytes.^99−103^ Most of SA-based bifunctional electrocatalysts for oxygen electrocatalysis are supported by N-doped carbon-based materials, encompassing the typical metal–nitrogen–carbon structure.^104−109^ For instance, Chen *et al.* anchored Fe SAs with a loading of 1.96 wt % on a nitrogen doped porous carbon. In order to achieve increased number of active sites, a “polymerization-pyrolysis-evaporation” strategy to synthesize Fe-N_4_ sites from a bimetallic Zn/Fe polyphthalocyanine precursor was accomplished.^110^ Each Fe atom had 2+ or 3+ oxidation state, and after coordination with N atoms the Fe-N_4_ active sites were formed favoring the O_2_ activation for the ORR reaction (Figure 7a). Although Zn atoms evaporated during pyrolysis, they were crucial for attaining the single-atomic state of the Fe cations, since in the absence of Zn, iron-based nanoparticles were formed. Possibly the evaporation of Zn^2+^ cations generated uncoordinated N sites to stabilize Fe SAs during pyrolysis. The catalyst was also very effective for the OER, a very challenging process of the ZAB chemistry. Density functional theory (DFT) calculations revealed the beneficial role of the Fe SAs for the O–O bond formation during the conversion of O* to OOH*, which was identified as the rate-determining step. The catalyst was more active than the commercial RuO_2_ and IrO_2_ for the OER. Benefiting from the synergistic effects between atomically dispersed Fe-N_4_ sites and a porous conductive carbon support, the ensued ZAB showed a power density of 232 mW cm^–2^ without voltage changes after 108 cycles (Figure 7b). In another example, Fe SAs were grown *in situ* while incorporating iron-1,10-phenanthroline (Fe-Phen) complexes into nanocages during the growth of zeolitic imidazolate framework-8 (ZIF-8), followed by pyrolysis under inert atmosphere.^111^ The atomically dispersed Fe-N_*x*_-C catalyst showed an ultrahigh ORR activity and excellent electrochemical performance. However, the activity of the OER was suboptimal and substantially lower than that of RuO_2_. A high open-circuit voltage (OCV) of 1.51 V was recorded, with a power density of 96.4 mW cm^–2^, significantly lower than the previously described Fe-SASC for ZAB, which was more effective for the OER charging process. ZIF-8 was likewise employed to create a functionalized hollow structure and achieve electronic modulation of an active center by near-range coordination with nitrogen and long-range interaction with sulfur and phosphorus (Fe-SAs/NPS-HC).^112^ As primary active sites, the isolated Fe-N_4_ species activated and reduced O_2_. The homogeneously dispersed P and S atoms did not coordinate with Fe atoms directly but modulated the electrical states via long-range interactions, which weakened the binding toward OH* intermediates to release OH^–^, boosting the four-electron ORR during discharging. The positive half-wave potential (*E*_1/2_) value of 0.912 V vs reversible hydrogen electrode (RHE) in acidic media showed outstanding catalytic activity. Moreover, when the Fe-SAs/NPS-HC was used as the air cathode, ZAB exhibited an OCV of 1.45 V, higher than that of the Pt/C-based battery, alongside a higher current density, reaching 195.0 mW cm^–2^ at a current density of 375 mA cm^–2^, while the Pt-C battery cathode delivered 177.7 mW cm^–2^ at 283 mA cm^–2^.^112^ It is observed that despite the excellent catalytic activity of this SAC for the ORR, the system did not surpass the Zn/Fe polyphthalocyanine-derived SAC cathode, which performed better toward OER charging.^110^ The heteroatom employment for tweaking the charge balance via push–pull phenomena was leveraged as well in another recent work, with N and P dual-coordinated iron sites. The carbon nanosheets embedded with nitrogen and phosphorus dual-coordinated iron active sites (denoted as Fe-N/P-C) were prepared via the pyrolysis of a polypyrrole hydrogel accompanied by treatment with sulfuric acid to produce the carbon nanosheets that contain nitrogen and phosphorus dual-coordinated iron active sites (Figure 7c, d). The Fe-N/P-C SAC showed high oxygen activation for the ORR process, with low energy required for the release of adsorbed *OH into reduced OH^–^ form, resulting in accelerated ORR kinetics.^113^ The fast ORR was theoretically found to be promoted by the dual doped system with P in addition to N. However, the performance in the OER process was suboptimal. Thus, the evaluation of the material as a ZAB cathode delivered an OCV of 1.42 V and a maximum power density of 133.2 mW cm^–2^ at a current density of 219.6 mA cm^–2^ (Figure 7e). This relatively low value could be as well connected to the sluggish performance in the OER charging process.

Interest in flexible and low weight wearable electronics has emerged in the past several years, and particularly for systems that can integrate energy storage properties with both high energy and power density for powering various wearable and portable devices.^114^ Thus, extensive efforts have been devoted for developing various types of flexible, thin, and low weight rechargeable batteries and supercapacitors.^115^ In 2022, a flexible and binder-free ZAB cathode was synthesized by a simultaneous construction of carbon nanotube (CNT)-linked N-doped porous carbon nanofibers (NCFs) and the dispersion of cobalt SAs via an electrospinning and carbonization strategy.^116^ The NCFs presence guarantees the active site’s accessibility, while the interior CNTs enhance the flexibility and mechanical strength of the porous fibers. Benefiting from the self-supporting structure obtained by electrospinning, Co SA/NCFs can be used as a binder-free air cathode while a zinc plate employed as an anode and a gel electrolyte (6 M KOH + 0.2 M Zn(CH_3_COO)_2_) completed the all solid state ZAB. The as-prepared catalyst delivered a high specific capacity of 796 mAh g_Zn_^–1^ at a current density of 10 mA cm^–2^, as well as super durability of 600 h at 10 mA cm^–2^ for aqueous ZABs with a small voltage gap for all-solid-state ZABs. Furthermore, the cathode’s increased flexibility paves the way for self-supporting electrodes for aqueous ZABs and flexible all-solid-state zinc-air batteries. Later in the same year, the development of a high-rate and robust quasi-solid-state ZAB using atomically dispersed cobalt sites anchored on wrinkled nitrogen-doped graphene as the air cathode and a polyacrylamide organohydrogel electrolyte was also reported.^117^ This design enabled a cycling current density of 100 mA cm^–2^ over 50 h at 25 °C and a low-temperature cycling stability of over 300 h (at 0.5 mA cm^–2^) with over 90% capacity retention at −60 °C and a broad temperature adaptability (−60 to 60 °C). The highly wrinkled graphene created a large charge gradient around the Co-N4 sites, strengthening the adsorption of oxygenated intermediates. After benchmarking under the same conditions, it became evident that the performance of the developed Co SAC cathode surpassed that of Pt/C-based aqueous ZABs. Copper SAs anchored on nitrogen-doped porous carbon (Cu-N/PC) derived from zeolitic imidazolate frameworks (ZIFs) were also employed as ZAB cathodes.^118^ Zn/Cu bimetallic ZIFs are promising precursors for Cu-N/C catalysts with Zn acting as a “fence” to avoid Co aggregation during pyrolysis and N-groups serving as a “coordinator” to protect and stabilize the copper SAs in nitrogen-doped carbon. Specifically, the spatial isolation of Cu SAs was regulated by tuning the zinc dopant content in Cu-ZIF-8 precursors followed by direct pyrolysis. The sample with 20% of Cu^2+^ precursor with respect to the Zn^2+^ precursor had a polyhedral shape with an average size of ∼120 nm and generally better Cu dispersion. The electron transfer mechanism involved in the ORR of the catalyst in potential ranges of 0.3–0.6 V indicated an efficient 4e^–^-dominated ORR and a low percentage of H_2_O_2_. As a result, Cu SAs, in combination with the micromesoporous structures of the carbon matrix, generated a synergistic effect providing fast electron transfer pathways to enhance the electrocatalytic properties toward ORR. The developed ZAB equipped with Cu-N/PC delivered improved performance, including a specific capacity of 704.9 mAh g_Zn_^–1^ at a discharge current density of 10 mA cm^–2^.

**Figure 8:**
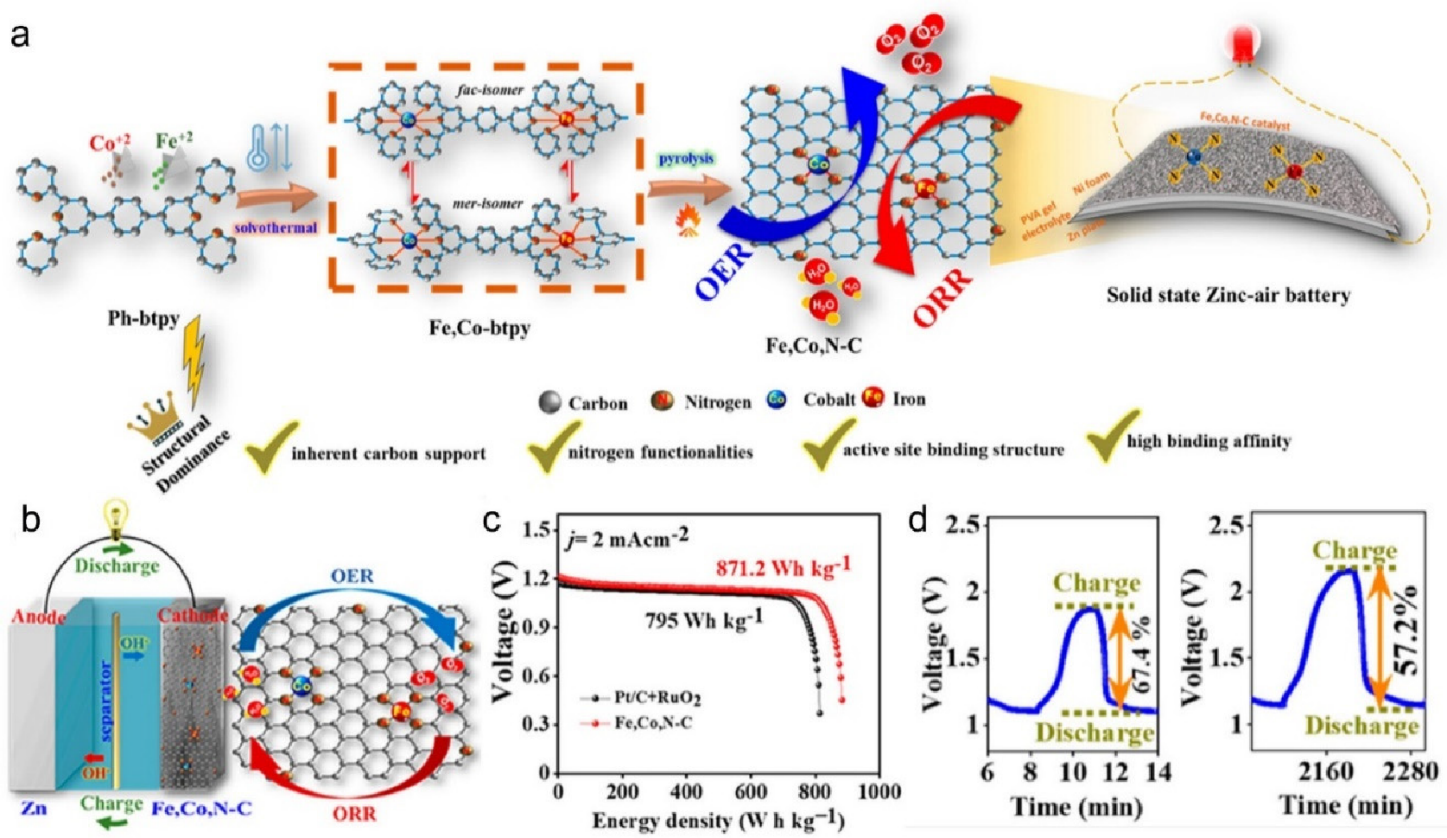
(a) Schematic illustration of the two-step synthesis strategy for the fabrication of the Fe, Co, N-C electrocatalyst toward high-performance zinc-air batteries and the possible chemical structure formed. (b) Schematic diagram of a cell-based rechargeable zinc-air battery with a 6 M KOH + 0.2 M Zn(acetate)_2_ electrolyte. (c) Energy density plots at a current density (*j*) of 5 mA cm^–2^ of cell-based rechargeable zinc-air batteries with liquid electrolyte using the Fe, Co, N-C dual SAC or a Pt/C + RuO_2_ noble metal-based cathode. (d) Initial and final (after 34 h) charge–discharge curves transformation of an all-solid-state rechargeable zinc-air battery with Fe, Co, N-C dual SAC as the air cathode. Reprinted with permission from ref (119). Copyright 2022 American Chemical Society.

The already described SAC electrode materials contained SAs from one metal. In 2022, Dey et al. reported the development of a bifunctional dual-metal SA electrocatalyst with Co and Fe ions in Fe-N_4_/C and Co-N_4_/C isolated active sites, exhibiting a symbiotic effect on overall oxygen electrocatalysis performances.^119^ It was previously known that cobalt oxides are a popular choice as bifunctional catalysts due to their relatively good performance in both ORR and OER compared to other metal oxides. Moreover, the interfacial dynamics developed upon combination of Co oxides with carbon structures has been proven as a potent toll for altering the performance of the resulting catalysts.^120^ The high conductivity of carbon materials, such as graphene, can boost the relatively low conductivity of Co oxides while providing increased catalytic surface area and a host structure for Co to be embedded in to form active sites.^121^ The study was also triggered by theoretical calculations suggesting that the introduction of another metal active center in the presence of Fe-N_*x*_ motifs could favor the ORR/OER activity and that Fe-Co bimetallic catalysts promote oxygen binding with a low activation energy and improve the initial onset of ORR. On this basis, the authors bound the Fe and Co precursors to 4′,4⁗-(1,4-phenylene)bis(2,2′,6′,2″-terpyridine) molecules (denoted as Ph-btpy) during solvothermal treatment (Figure 8a). The Ph-btpy ligand was able to anchor two metal atoms at its two open ends containing pyridinic N centers, which provides a highly consistent metal–ligand coordination site. The final product had a square planar coordination of the M-N_*x*_ moieties, which enabled synergistic function toward a bifunctional SAC for both OER and ORR and which could be utilized as an air cathode in ZABs (Figure 8b). Moreover, the presence of N dopants and the electronic synergism between the metal centers altered the metal orbital positions and led to the downshift of the energies of the metal d-orbitals, which weakened the adsorption of the reaction intermediates. Thus, the dual metal doping reduced the overpotential of the electrocatalytic processes (Δ*E*_ORR-OER_ = 0.74 ± 0.02 V vs RHE), resulting in a battery with an energy density higher even than that obtained by using a noble-metal-based Pt cathode (Figure 8c, d). Finally, the Fe, Co,N-C ZAB showed a high areal power density of 198.4 mW cm^–2^ and 158 mW cm^–2^ in the respective liquid and solid-state ZABs, demonstrating its high proclivity as an air cathode material in ZABs.^119^ The voltaic efficiency degraded by 10.2% after 38 h of operation. Overall, the Fe and Co SACs with the M-N-C structure are the most commonly used catalytically active species as cathode materials in ZABs. Fe-based SACs have shown advantages in terms of ORR performances, while the Co-based SACs appear to be beneficial in rechargeable batteries due to their bifunctional activity.^122^

#### 2.2.3 Li-CO_2_ Batteries

Despite the substantial superiority of Li-O_2_ batteries (with a full-cell theoretical capacity of ∼3500 Wh kg^–1^), their operation is restricted in normal atmosphere by side-reactions which occur due to the presence of H_2_O and CO_2_. Moisture reacts with Li_2_O_2_ discharge products by forming LiOH, which is not easily reversible back to the metallic Li and O_2_. In addition, CO_2_ is quite soluble in the electrolytes and combines with superoxide radicals to form the insulating Li_2_CO_3_, which has higher decomposition potential than Li_2_O_2_ during the recharging, resulting in inferior energy balance and cycle-life.^123^ In 2011, Takechi et al. studied a battery utilizing both O_2_ and CO_2_, presenting a 3-fold capacity higher than that of a pure O_2_-based battery.^124^ This motivated subsequent studies on pure Li-CO_2_ batteries due to their ability to capture CO_2_ and thus contribute to a carbon neutral economy.^125^ In addition, they offer promising energy storage systems for extraterrestrial missions, such as in the CO_2_-rich atmosphere of Mars. Therefore, metal-CO_2_ batteries, which involve the CO2RR and CO2ER, have recently evolved as an attractive sustainable EES technology with high added value due to carbon recycling.^55,126^ Current metal-CO_2_ batteries mainly embrace Li- or Na-CO_2_ and Zn or Al-CO_2_ systems. Due to the high reactivity of Li and Na, their operation requires organic electrolytes, thus allowing for high operation voltages, which translate into higher energy densities (1876 Wh kg^–1^ and 1130 Wh kg^–1^ for Li and Na-CO_2_ batteries, respectively). The driving force for energy conversion and storage in Li-CO_2_ batteries is the reversible redox reaction between a lithium anode and CO_2_ gas cathode to form (during discharge) and decompose (during charging) Li_2_CO_3_, according to the following reaction: 3CO_2_ + 4Li ↔ Li_2_CO_3_ + C.^127^ During discharge, Li-ions are released to the gas electrode and react with CO_2_ to produce Li_2_CO_3_ precipitates. Thus, a large surface area of the gas electrode is required to accommodate these insulating and insoluble salts of the discharge process. Accordingly, the effective processing of Li_2_CO_3_ is a major limitation, since it deposits and accumulates on the cathode during discharge.^128^ Notably, this dramatically hinders the kinetics of CO_2_ evolution during charging, leading to a high voltage (>4.5 V vs Li/Li^+^) required for decomposing Li_2_CO_3_.^129^ The result is poor reversibility and low energy efficiency.^130,131^ Therefore, identifying effective catalysts at the gas electrode for the fast and low-energy decomposition of Li_2_CO_3_ (i.e., the CO_2_ evolution reaction) is among the primary challenges for the practical applicability of Li-CO_2_ batteries. In general, the principles for the design of effective Li-CO_2_ batteries cathode catalysts could be summarized as the following: (i) good CO_2_ capture capability, (ii) uniform and well-defined catalytic sites for CO_2_ reduction/evolution, (iii) fast Li ion transfer pathways, and (iv) efficient electron transfer.^132^

In an interesting work, theoretical calculations were performed to screen SACs on N-doped graphene (noted as SAMe@NG, Me = Cr, Mn, Fe, Co, Ni, Cu) for CO_2_ reduction and evolution reaction.^133^ Among them, Cr SAs showed promising activity as an effective electrocatalyst for reversible Li-CO_2_ batteries due to the superior CO_2_ adsorption and Li_2_CO_3_ decomposition ability. Thus, the SACr@NG with strong Li_2_CO_3_ adsorption had the lowest decomposition potential barriers of 1.674 eV, suggesting that Cr-N_4_ moieties effectively improve the reaction kinetics in the charge process, indicating that SACr@NG is the best candidate for CO2ER and CO2RR, respectively, in Li-CO_2_ batteries. To confirm the applicability of the catalyst as electrode, the authors constructed batteries with a SACr@NG/PCF cathode which exhibited the narrowest overpotential of 1.39 V over 350 cycles at a rate of 100 μA cm^–2^ and showed an extraordinary stability with a long cycle life of over 350 cycles at a current density of 100 μA cm^–2^. Graphene oxide (GO) has been likewise employed for anchoring Co SAs on its surface with a high loading of 5.3 mass% and used as an efficient and durable electrocatalyst for Li-CO_2_ batteries.^134^ The average Co–Co distance between adjacent Co atoms was 1.79 Å, which facilitated the synergistic action of the dispersed Co SAs, providing adjacent catalytically active sites to decompose Li_2_CO_3_. The synergistic Co/GO exhibited greater capacity and Coulombic efficiency and lower charge overpotential in comparison to control systems, such as SA Co/GO and Co nanoclusters/GO. This resulted in a high and sustained discharge capacity of 17358 mAh g^–1^ at 0.1 A g^–1^ for >100 cycles. In terms of better understanding the activity of such cooperative Co SAs, DFT calculations identified an increased energy of adsorption of the LiCO_3_ discharge product on the catalyst only in the case where the Co atoms were in close proximity.

**Figure 9:**
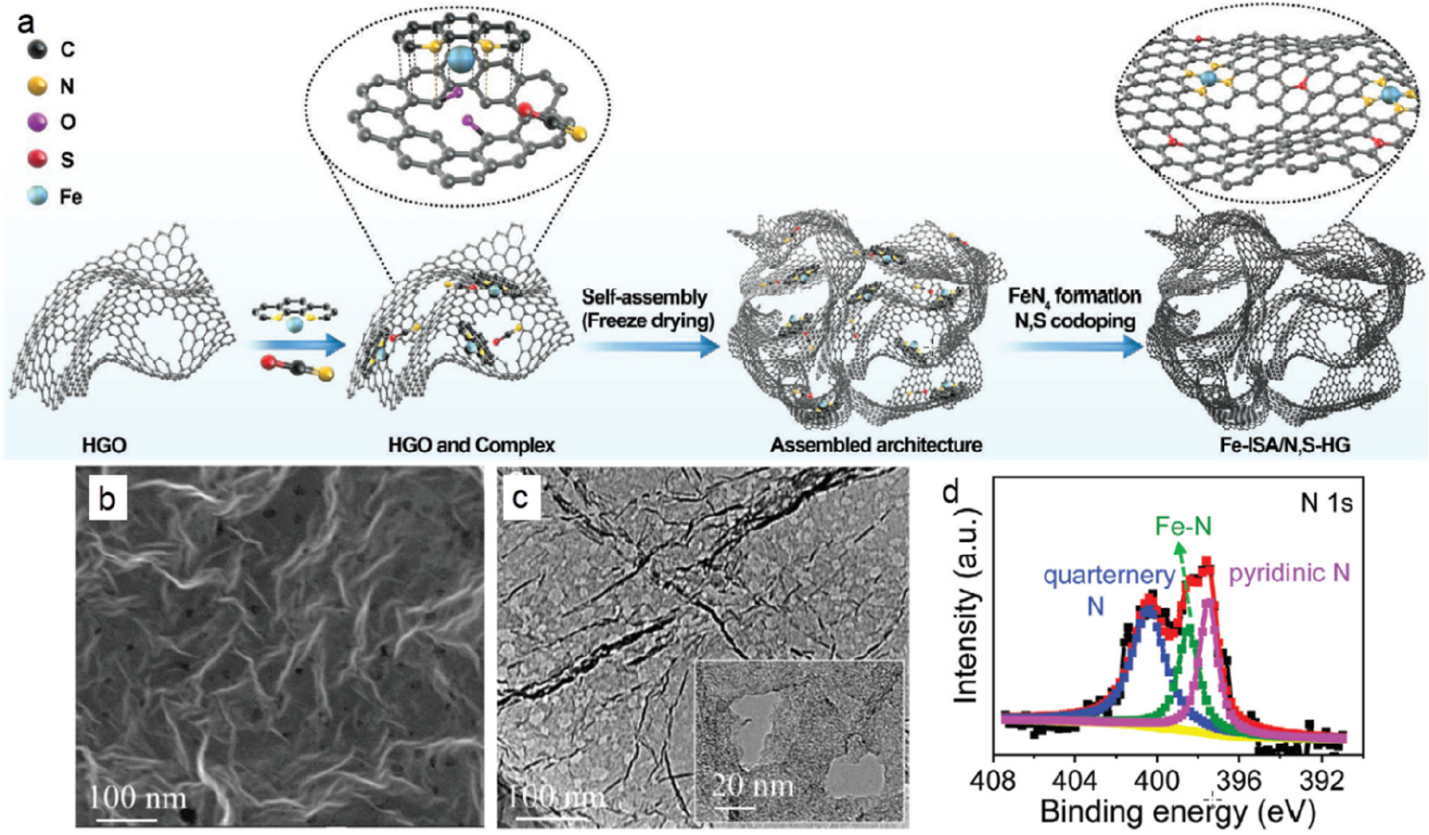
(a) Illustration of the synthesis process of the bifunctional catalyst for CO_2_ reduction and evolution reactions (b) SEM image of the catalyst with the pores evident in the structure. (c) TEM and HR-TEM (inset) images and (d) HR-XPS spectra of the N 1s region. Reprinted with permission from ref (135). Copyright 2020 Wiley-VCH.

Dai et al. implanted iron SAs into 3D porous carbon architectures, consisting of interconnected N,S-*co*-doped holey graphene sheets as a highly efficient catalyst for CO2RR and CO2ER in rechargeable Li-CO_2_ batteries.^135^ The synthesis of catalyst included a two-step approach, involving a complexation reaction of Fe cations between 1,10-phenanthroline units and holey graphene, followed by postannealing in the presence of thiocyanates, leading to the formation of networks via π–π stacking and coordination with the Fe ions (Figure 9a). SEM images confirmed the well-defined and interconnected 3D porous network, while transmission electron microscopy (TEM) analyses demonstrating the presence of 5–20 nm pores and the atomic dispersion of Fe species (Figure 9b, c). Moreover, the characteristic components of the HR-XPS N 1s spectrum centered at 400.4, 398.5, and 397.1 eV were attributed to quaternary N, Fe–N, and pyridinic N, respectively, confirming the incorporation of N into the carbon skeleton and the developed interactions between Fe and N (Figure 9d). Theoretical calculations indicated that both N and S dopants and “FeN_4_” in the final catalyst act as dual active sites for CO_2_ reduction and evolution reactions. Furthermore, the hierarchical porous structure with the interconnected holey graphene framework can facilitate the electron/ion transport channels, while ensuring an effective exposure of the active sites leading to the formation of small Li_2_CO_3_ nanoparticles. As a result, the prepared Li-CO_2_ battery exhibited a high capacity of 23174 mAh g^–1^ based on the catalyst mass, low polarization (1.17 V at 0.1 A g^–1^), and long-term stability over 200 cycles, at a cutoff capacity of 1 Ah g^–1^, at 1.0 A g^–1^.

In 2021, a Li-CO_2_ battery cathode catalyst of a porphyrin-based covalent organic framework (TTCOF-Mn) with single metal Mn sites was reported, via the covalent connection between the electron-donating ligand and the catalytically active moiety of tetrakis(4-aminophenyl)-porphinato manganese(II).^136^ These covalent organic frameworks (COFs) with metalloporphyrin moieties provided a promising platform to construct single-site catalysts due to the spatially separated and unsaturated coordination sphere of the single-metal sites.^137^ Both the electron-donating properties of the ligand and the uniform micropore channels ensured the effective electron transfer, high CO_2_ adsorption, and rapid Li ion transport, while simultaneously contributing to the efficient discharge–charge processes on the TTCOF-Mn cathode catalyst. The battery with TTCOF-Mn exhibited a low overpotential of 1.07 V at 0.1 A g^–1^, a capacity of 13018 mAh g^–1^, as well as an excellent stability of 180 cycles, at a cutoff capacity of 1 Ah g^–1^, at 0.3 A g^–1^. For comparison, a SAC that contains the precious metal Ru absorbed on rGO-templated sandwich carbon sheets with rich nitrogen doping can deliver an ultrahigh capacity of 44.7 Ah g^–1^, an ultralow charge/discharge polarization of 0.97 V at 0.1 A g^–1^ (1.90 V at 2 A g^–1^), and a long-term cycling stability up to 367 cycles at 1 Ah g^–1^.^138^ In general, Ru nanoparticles or SAs of Ru ions provide highly active reaction sites for Li_2_CO_3_ decomposition during charging, resulting in enhanced cycling performance.^139^ However, the significantly high cost and scarcity of noble metals limit their application potential.

#### 2.2.4 Zn-CO_2_ Batteries

The high activity of Li/Na-CO_2_ batteries, in the absence of protic solvents, produces primarily low carbon content products during discharging (CO_2_ reduction), such as carbonates. Zn- and Al-CO_2_, on the other hand, can operate in more safe and cost-effective aqueous electrolytes. However, this limits the operation potential due to water electrolysis, resulting in lower energy densities. Nevertheless, the discharging process of CO_2_ reduction in water affords CO, C_2_H_4_, HCOOH, and C_2_H_5_OH, contributing to a high-level of CO_2_ valorization and carbon circular economy.^55^ Using SACs in this case proved to be particularly beneficial for improving the kinetics of the reactions. In a related work, Fe SAs on a carbon support (Fe_1_NC) demonstrated acceleration for the CO_2_ electron reduction kinetics in rechargeable Zn-CO_2_ batteries, reaching a CO Faradaic efficiency (FE) up to 96% at −0.5 V and a turnover frequency of 2225 h^–1^, along with outstanding stability.^140^ In particular, Fe-N_3_ sites appear to be key for the optimization of the *COOH/*CO intermediates adsorption energies, at neither too strong nor too weak levels, boosting the final conversion to CO. The resulted catalyst achieved an ultrahigh power density of 526 mW cm^–2^ and ran for 72 cycles at 0.5 mA cm^–2^, demonstrating the potential of utilization of SACs in Zn-CO_2_ batteries.^140^

Apart from studies of SACs based on one type of metal, SAs from two different metals may interact with each other through their microenvironment and work cooperatively to activate more effectively the reactants or for improving the sorption–desorption balance of reactants, intermediates, and products. However, often, further improvements are required for lowering the onset potential (reaction energy barriers) or releasing easier intermediates, such as CO, which seriously compromise Faradaic efficiencies and catalyst stability. Moreover, too strong binding strength of TM sites with electron-donating intermediates lowers the catalytic activity for oxygen evolution reaction.^141^ For example, in nature, the Ni-Fe carbon monoxide dehydrogenase with Fe and Ni SA sites bridged by sulfide ligands can synergistically catalyze the efficient interconversion of CO_2_ and CO under mild conditions.^142^ Inspired by this Nature’s blueprint, Jiang et al. synthesized a novel Fe_1_-Ni_1_-N-C catalyst with Fe and Ni SA pairs decorated on nitrogen-doped carbon.^143^ Given the great advantages on structural and component regulation, MOFs represent a particularly attractive platform for the construction of SACs.^144^ Among various types of MOF-derived SACs, SA decorated nitrogen-doped carbons (M-N-C), with planar and conjugated carbon motives, can readily achieve the required long-range electron delocalization and couple adjacent nonbonding single metal atoms.^106^ Theoretical simulations revealed that the Fe SACs can be highly activated by adjacent Ni SAs via nonbonding interactions, significantly facilitating the formation of COOH* intermediate and thereby accelerating the overall CO_2_ reduction. More specifically, by the direct pyrolysis of Fe- and Ni-doped ZnO nanoparticles anchored on zeolitic imidazolate frameworks (ZIF-8) and via a Zn-assisted atomization strategy during pyrolysis, a ZIF-derived nitrogen-doped carbon implanted by adjacent Fe-N_4_ and Ni-N_4_ sites was obtained. Thanks to the synergism of the neighboring Fe and Ni SA pairs, the catalyst offered significantly enhanced performances for electrocatalytic reduction of CO_2_, far surpassing the one-metal type SACs of Fe or Ni SAs. For the Zn-CO_2_ battery testing, an H-shaped cell divided by a bipolar membrane was used with the cathode compartment being bubbled with CO_2_. The charge and discharge voltages under different current densities showed rechargeable behavior and excellent selectivity to CO with a FE up to 93.4% at 1 mA. In the same period, Zeng et al. also described the development of a bimetallic SAC consisting of nickel–iron heterodiatomic pairs anchored on nitrogen-doped graphene for performing both the CO2RR and OER.^141^ The catalyst/cathode was synthesized by pyrolyzing l-alanine, ferric (II) acetate, nickel(II) acetate tetrahydrate, and melamine together in argon atmosphere. adding Fe acetate (or Ni acetate). The synthesis resulted in a mixture of nanoparticles and SA metal cations. Thus, the product was ground and washed by 2 M HCl solution at 80 °C for 24 h under stirring to remove metal particles. The Ni-Fe SAC exhibited extraordinary and stable electrocatalytic performance for CO2RR and OER, and the rechargeable Zn-CO_2_ battery equipped with such bifunctional catalyst showed high FE and outstanding rechargeability. The electronic structure analysis revealed that the Fe cations in the Ni-Fe heteroatomic pairs served as the catalytic centers, while the Fe orbital coupling with Ni lead to a higher oxidation state weakening the binding strength with the intermediates. The Zn-CO_2_ battery equipped with a NiFe bimetallic SAC cathode offered the largest discharge voltage and the lowest charge voltage as compared to the batteries with a Ni-SAC or Fe-SAC cathode. The Zn-CO_2_ battery could be operated under large discharge current densities over 10 mA cm^–2^, delivering a maximum power density of 1.36 mW cm^–2^ at 8.5 mA cm^–2^. The Zn-CO_2_ battery exhibited a high FE of 90% at 5 mA cm^–2^. The authors found that the 3d states of Fe in the NiFe-SAC were less localized than those in Fe-SAC, attributed to the strong d-d orbital coupling between the heteroatoms, leading to decreased orbital energy levels and delocalization of electrons, beneficial to *CO desorption. This finding is of particular importance, considering that the rate limited step of CO2RR for Fe-SAC is the desorption of CO intermediate.^145^

#### 2.2.5 Al-Air Batteries

Having the same principles as ZABs, Al-air batteries are another promising energy storage chemistry. This is mainly due to the low equivalent weight of aluminum leading to high specific energies, its low reactivity with humidity and oxygen rendering it a safe metallic anode, and nontoxic and environmentally friendly charging and discharging products. Theoretically, Al contains approximately half of gasoline’s energy content per unit weight (8100 Wh kg^–1^ for Al-air batteries and 13 000 Wh kg^–1^ for gasoline) and three times the energy per unit volume (21 870 Wh L^–1^ for Al-air and 9700 Wh L^–1^ for gasoline).^146^ However, as presented in Figure 3 that the practical cell-level values are significantly lower, dropping below 400 Wh kg^–1^ for Al-air batteries in comparison to 1700 Wh kg^–1^ for an internal combustion engine.^147^ Although the specific energy of Al-air batteries is the highest after Li-air batteries, Al has the great privilege of low cost, which is only 1/6 of that of Li, and an earth-abundance 4.5 thousand times higher than that of Li.^148^ Al-air batteries consist of an Al or Al alloy anode, an air electrode cathode, and an alkali or salt electrolyte that incorporates additives to suppress corrosion and H_2_ evolution, such as ZnO.^149^ During discharge the anode material (Al) dissolves to cations, while the oxygen molecules are reduced, producing electrical energy. The process can be summarized as the following reaction: Al + 3/4O_2_ + 3/2H_2_O → Al(OH)_3_.^150^ However, a highly negative potential is required for the reverse process of depositing Al metal at the anode, in an aqueous system, making the recharging process of Al-air batteries quite challenging and energy inefficient. Moreover, on Al anode’s surface a passivating oxide layer is formed, which not only reduces the operating cell voltage but also increases both the charge and mass transfer resistance. In turn, the OCV drops from −2.34 V to −1.87 V vs SHE at high pH, limiting the cell operating voltage and thus the energy density.^151^ Last but not least, particularly in alkaline electrolytes, the oxide passivating layer can be removed, which then gives rise to a high corrosion rate, further limiting the operating cell voltage between 1.2 and 1.6 V instead of the theoretical of 2.74 V.^148^

**Figure 10:**
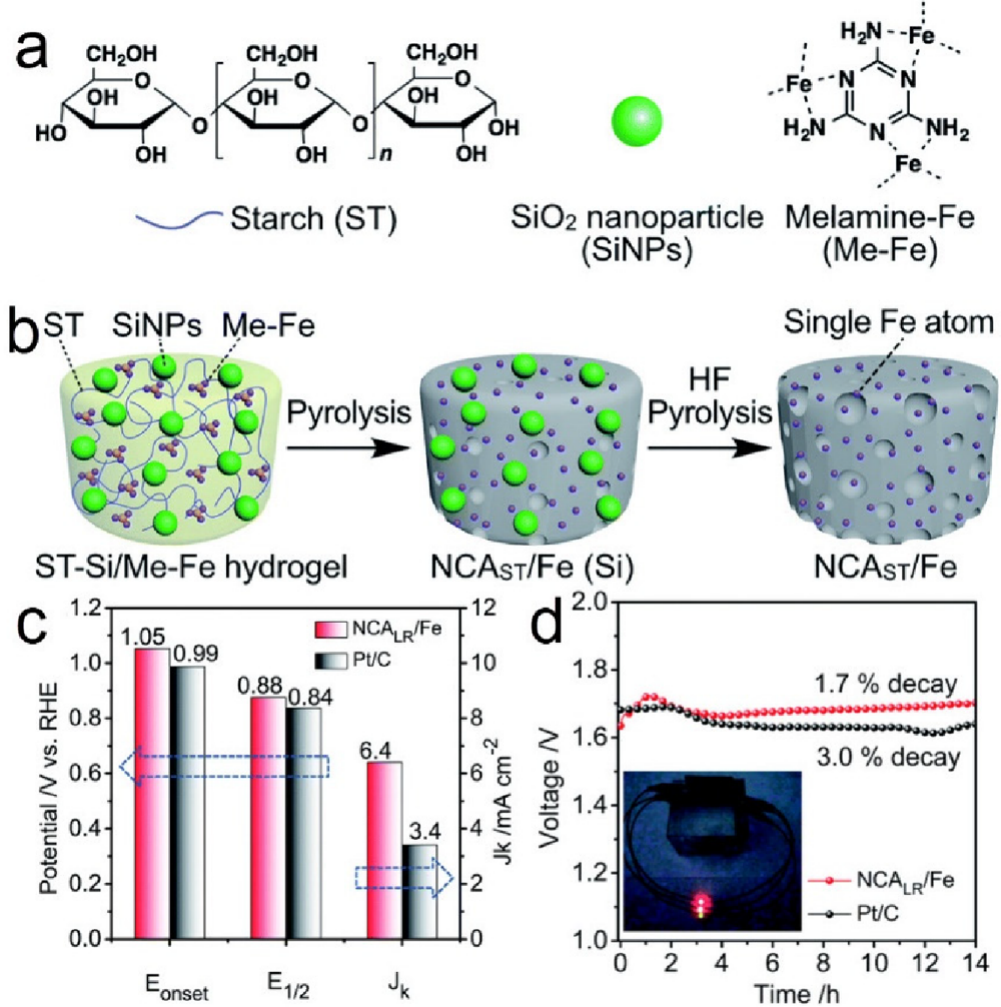
(a) Chemical structure of the starch and schematics representing the pore-forming SiO_2_ nanoparticles and the melamine-Fe complex. (b) Schematic illustration for the synthesis of single Fe atoms dispersed in N-doped carbon aerogels (NCA/Fe). (c) E_onset_, *E*_1/2_, and J_k_ (at +0.85 V) of one type of the NCA/Fe carbon aerogel catalyst and comparison with the performance obtained from a commercial Pt/C catalyst. (d) Constant current discharge tests of the same catalysts in panel c, at the current density of 20 mA cm^–2^; inset: photo of parallel red, yellow, and green LEDs (rated voltages of 1.8 to 2.0 V) simultaneously powered by only one Al-air battery assembled using NCA/Fe as the cathode catalyst. Reprinted with permission from ref (154). Copyright 2019 Royal Society of Chemistry.

To overcome these bottlenecks, the introduction of catalytic sites is studied with high intrinsic ORR activity in order to push the reaction kinetics at the three-phase interface.^152,153^ Recently, 3D N-doped carbon aerogels embedded with Fe SAs were prepared and studied for Al-air batteries.^154^ Three kinds of biomass starch hydrogels were evaluated as 3D templates, mixed and interconnected with Fe ions coordinated with melamine, acting as the metal and N source, respectively (Figure 10a). The hydrogels after pyrolysis formed the Fe SA porous carbon hosts (NCA/Fe, Figure 10b), which showed excellent electrocatalytic performance in O_2_-saturated 0.1 M KOH toward ORR with an onset potential of+1.05 V and half-wave potential of +0.88 V, all more positive than those of commercial 20 wt % Pt/C (Figure 10c), due to the higher activity of the Fe-N_4_ catalytic motifs in the biomass-derived, hierarchical porous carbon aerogels. The onset potential determination is an excellent tool to characterize the catalytic performance of a material, being the highest for cathodic reactions (ORR) and lowest for anodic reactions (OER), in which a reaction product is produced at a given electrode and defined conditions.^155^ Therefore, the application of the aerogel SACs as an Al-air battery cathode exhibited a higher OCV (1.81 V) and power density (181.1 mW cm^–2^) and more stable discharge voltage of 1.70 V at 20 mA cm^–2^, substantially better than those with a Pt/C cathode (Figure 10d).

The elaborate exploration of nonprecious metal catalysts for efficient ORR is imperative for the practical development of fuel cells and metal-air batteries.^156^ Among the reported non-Pt ORR catalysts, the molecular catalyst of iron phthalocyanine (FePc) has aroused much attention due to its special Fe-N_4_ active site and low reaction energy barrier during ORR.^157^ However, FePc, with the typical two-dimensional and plane symmetric structure, leads to the symmetric electron distribution in the FeN_4_-active sites and is not conducive to the O_2_ adsorption and activation.^158^ In order to improve the ORR activity of the FePc catalyst, Liu et al. broke the symmetry of electronic density with a composite catalyst (FeAB-O) by coordination of the FePc molecule with oxygen-containing groups on an O_2_ plasma-treated acetylene black (AB-O) matrix to achieve efficient O_2_ adsorption and ORR.^157^ Theoretical calculations showed that although the number of charges and spin polarization of the symmetrical FeN_4_ site did not significantly change, the axial O coordination accepts partial charges from the Fe-N_4_, breaking the symmetry of the electronic density near the Fe-N_4_ site. As a result, the FeAB-O showed much higher O_2_ adsorption energy by 0.92 eV. Thus, the stable adsorption of oxygen could facilitate the process of ORR having an ORR overpotential of 0.70 V. This FeAB-O catalyst exhibited one of the best half-wave potentials of 0.90 V vs RHE, which is superior to that of commercial Pt/C. Thus, the introduction of axial O coordination in O-FeN_4_ sites makes the catalyst superior to most of the reported Fe-N-C catalysts.

Apart from Fe-based SACs, Li et al. synthesized Co-N_4_ active centers on high specific surface area and pore-rich biomass-derived 3D ultrathin porous carbons, to increase the active sites and boost mass transfer. Using this as catalyst for the cathode in a homemade Al-air battery, the authors achieved a high OCV, reaching 1.80 V, which is comparable to that of Pt/C (1.82 V), displaying a particularly improved peak power density of 494 mW cm^–2^, than that of Pt/C (449 mW cm^–2^). Interestingly, the high activity of the catalyst, boosting the Al-air battery performance during discharging, was supported by theoretical calculations, which revealed downhill free energy changes for the ORR individual reaction steps (or small energy barriers depending on the potential where the DFT calculations were studied). The most demanding step in this case was the adsorption of oxygen, electron/proton transfer, and reduction to OOH* species. On the contrary, the OER reaction steps were very demanding in all cases, reflecting the challenging recharging process of oxygen generation from the Al(OH)_3_ precipitates and Al reduction and homogeneous deposition back to the anode.^159^ In summary, the Fe and Co SAs on nitrogen-doped carbon hosts are most commonly employed and studied in metal-air batteries as active catalytic sites on the cathode. It appears that Fe SACs favor the ORR performance while the Co SACs show some interesting properties with bifunctional catalytic activity, which might be advantageous in metal-air batteries.

The application of nonprecious metal SACs for metal-air and metal-CO_2_ batteries has undoubtedly attracted increased attention over the past few years (Table 1) because SACs can enhance the ORR and OER in metal-air batteries and the CO2RR and the CO2ER in metal-CO_2_ batteries. The results have demonstrated substantial reduction in the overpotentials at the cathode, which is a major limiting factor in the performance of metal-air and metal-CO_2_ batteries regarding their energy efficiency. SACs can also improve the electron transfer between the cathode and the electrolyte, reducing the resistance and improving the rate of the battery. Despite these advancements, further research is needed to overcome several challenges. Metal-air batteries typically struggle in ambient air because of water and carbon dioxide. While membranes that can separate moisture from the atmosphere have shown some success, the difficulty separating oxygen from carbon dioxide in ambient air has led to researching metal-CO_2_/O_2_ batteries, where the gas separation would not be required.^160^ Highly selective and multifunctional catalysts in the cathode would be thus very beneficial in order not only to perform both the ORR and the CO2RR but also to decompose effectively the variable discharge products. Potent multifunctional catalysts based on bi- or even multimetallic cooperative SACs for tandem catalysis will probably play a primary role in the development of these technologies.^141,161^ Effective formations and transformation of solid intermediates which are less corrosive for the battery components is another valuable strategy, such as the reversible formation of Li_2_O instead of Li_2_O_2_.^80^ Li_2_O_2_ involves the superoxide and peroxide species which are detrimental to the stability of the cathode materials and the electrolytes; however, for being thermodynamically more favorable during discharging, it has been the main chemistry in Li-air batteries. In metal-CO_2_ batteries, on the other hand, the matter of carbon capture and sequestration implementation is usually both expensive to build and energy intensive to operate.^160^ SACs could offer promising opportunities toward the acceleration of metal-CO_2_ battery reactions, but the decomposition of their discharge products during charging should be given particular attention in order to achieve good performance in full cells and a long battery life.

**Table 1:** Electrochemical Performance of SAC-Based Materials Studied as Electrodes for Energy Storage Devices in Terms of Their Synthesis Method, Battery Type, Specific Capacity, Power Density and Cycling Stability

Material	SAC	Synthesis method	Battery	Current density	Max. cycles	Capacity (mAh g^–1^)	Power density (mW cm^–2^)	Ref
N-HP-Co SACs	Co	polymerization of cobalt complexes and pyrolysis	Li-O_2_	0.1 A g^–1^	261	1000 cutoff		(90)
Co-SAs/N-C	Co	green gas-migration-trapping strategy	Li-O_2_	0.4 A g^–1^	260	1000 cutoff		(91)
Fe, Co-SA/CS	Fe, Co	heat and acidic treatments of ZIFs grown on carboxylic polystyrene nanospheres	ZAB	5 mA cm^–2^	-	819.6	86.65	(103)
Fe-N-C/N-OMC	Fe	KIT-6 as template and Fe(II)-Phen complex/2-methylimidazole as the Fe, N, C precursors	ZAB	10 mA cm^–2^	-	711	113	(104)
3DOM Fe-N-C	Fe	pyrolysis of ferrocene-encapsulated macro-microporous ZIF-8 precursor	ZAB	20 mA cm^–2^	-	768.3	235	(108)
Fe-N_4_ SAs/NPC	Fe, Zn	polymerization–pyrolysis evaporation strategy to synthesize N-doped porous carbon with atomically dispersed Fe-N_4_ units	ZAB	50 mA cm^–2^	108	-	232	(110)
Fe-Nx-C	Fe	Fe-Phen encapsulated in nanocages during the growth of ZIF-8 and pyrolysis to isolate Fe-Nx-C SACs	ZAB	10 mA cm^–2^	-	641	96.4	(111)
Fe-SAs/NPS-HC	Fe	ZIF-8/Fe@PZS formation via core–shell composites via polymerization followed by pyrolysis	ZAB	375 mA cm^–2^	500	-	195.0	(112)
Co SA/NCFs	Co	one-step electrospinning and carbonization	ZAB	10 mA cm^–2^	-	796	154.5	(116)
Cu-N/PC	Cu	tuning Zn dopant content in Cu-ZIF-8 followed by direct pyrolysis and acid dissolution	ZAB	10 mA cm^–2^	-	704.9	215.8	(118)
Fe, Co,N-C	Fe, Co	Fe and Co precursors bound during solvothermal treatment. The resulted Ph-btpy ligand was able to anchor two metal atoms.	ZAB	330 mA cm^–2^		726	198.4	(119)
SACr@NG/PCF	Cr	two step pyrolysis from natural cotton and metal acetylacetonate mixing with GO	Li-CO_2_	0.1 mA cm^–2^	350	1500 μAh cm^–2^	-	(133)
SA Co/GO	Co	acid leaching of Co nanoclusters/GO	Li-CO_2_	0.1 A g^–1^	100 cutoff 1000 mAh g^–1^	17358	-	(134)
Fe-ISA/N,S-HG	Fe	Complexation reaction of Fe cations with 1,10-phenanthroline and holey graphene followed by annealing with thiocyanates	Li-CO_2_	1 A g^–1^	200 cutoff 1000 mAh g^–1^	23174	-	(135)
TTCOF-Mn	Mn	Solvothermal method via Schiff-base condensation between TAPP-Mn and TTF in the presence of aqueous acetic acid	Li-CO_2_	0.3 A g^–1^	180	13018	-	(136)
Fe1NC/S1-1000	Fe	N-doped porous carbon support derived from ZIF-8 precursor carbonized with diffused ferrocene ligand steam	Zn-CO_2_	0.5 mA cm^–2^	72	-	526	(140)
NCA_LR_/Fe	Fe	Pyrolyzed lotus root-derived hydrogels and HF etching	Al-air	20 mA cm^–2^	-	-	181.1	(154)
Co SANC-850	Co	Complexation of biomass and metal ions combined with a gas-foaming strategy	Al-air	200 mA cm^–2^	-	-	494	(159)

### 2.3 SACs in Metal Sulfur Batteries

**Figure 11:**
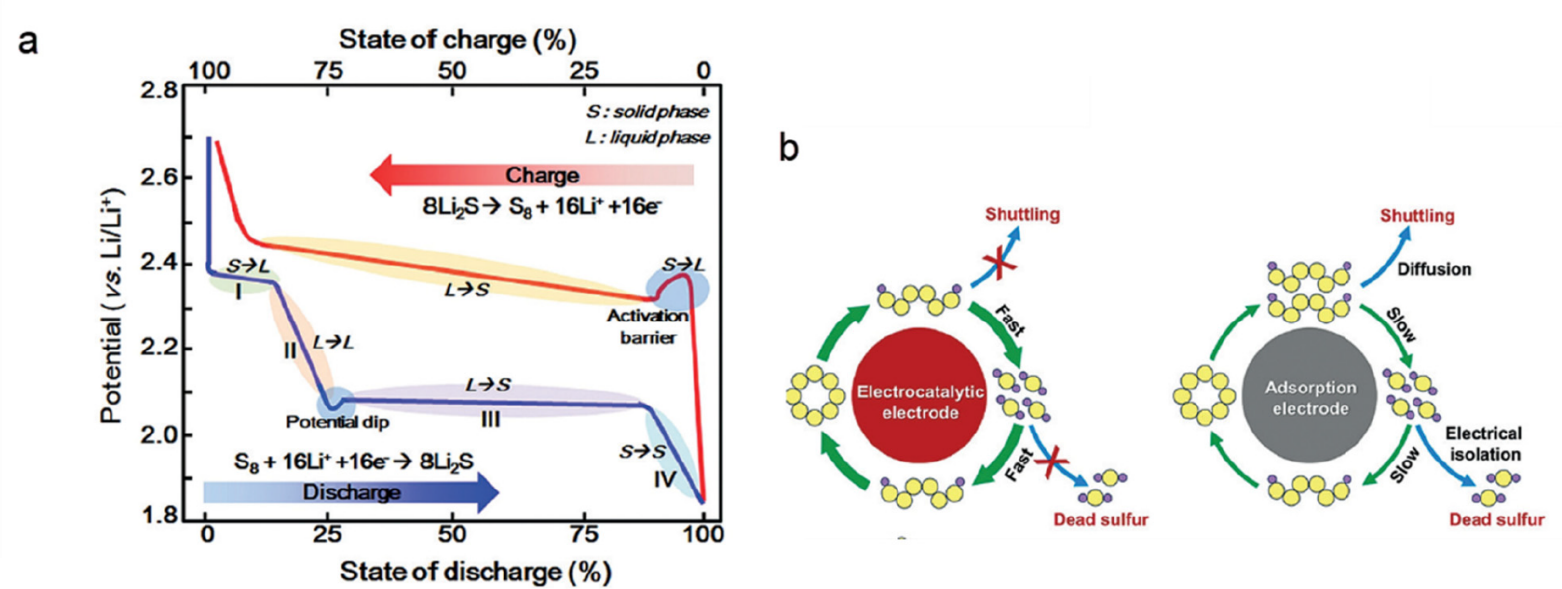
(a) Schematic of operating principles for LSBs. (b) Role of catalytic materials in electrodes compared with polar adsorbents. Reprinted with permission from ref (167). Copyright 2019 Wiley-VCH.

Metal-sulfur batteries (MSBs), based on conversion chemistry, have garnered tremendous attention due to their unparalleled theoretical energy content (in the case of lithium anode) along with the low cost, abundance, and environmental friendliness of sulfur. Among various MSB technologies, lithium-sulfur batteries (LSBs) have attracted the most attention as promising next-gen energy storage systems due to their high theoretical energy density of 2567 Wh kg^–1^,^162^ which counterbalances the smaller potential window of operation by 1/3 in comparison to that of intercalation-type positive electrodes. However, LSBs have several bottlenecks toward commercialization due to three main factors, among others:^163^ the insulating properties of S (both for electrons and ions) which demands the use of electrochemically inactive additives, the large volume expansion during discharge processes, and the “shuttling” effect of the highly soluble Li polysulfides (Li_2_S_*x*_, 4 ≤ *x* ≤ 8).^164^ Thus, in a LSB, the conversion between Li_2_S and sulfur in ether electrolyte undergoes a multistep reaction with multiphase transformations (solid ⇌ liquid ⇌ solid) through soluble polysulfides (Figure 11a).^165−167^ Three quarters of LSBs theoretical capacity comes from the slow liquid–solid phase transformation of soluble Li_2_S_4_ to solid Li_2_S.^168^ The sluggish kinetics of this process leads to accumulation of Li polysulfides (LiPS) in the cathode area and in the electrolyte resulting in precipitation of nonreusable solid Li_2_S on the cathode surface.^169^ This process leads to low sulfur utilization and the corrosion of the Li metal anode, resulting in rapid capacity fade and low lifespan. To address this issue, rapid conversion between soluble and insoluble LiPS on the surface of a catalytic material is required, rather than its adsorption on high surface area porous cathodes, because the catalytic material not only captures LiPS on its surface but also enhances the redox reaction kinetics of adsorbed LiPS by facile transport of ions and electrons (Figure 11b).^170^ Therefore, the exploitation of catalysts, like metal nanoparticles, metal oxides/nitrides, and metal sulfides, has been proposed to accelerate the conversion rates of soluble LiPS to insoluble Li_2_S during discharge and overall improve S utilization and cycle life.^171−175^ Among them, low-cost metal oxides have been widely considered as catalytic materials for the LSB chemistry, including Ti_4_O_7_, MnO_2_, MgO, Co_3_O_4_, Fe_2_O_3_, Fe_3_O_4_, MoO_3_, SnO_2_, ZnO, TiO_2_, ZnCo_2_O_4_, and NiFe_2_O_4_.^176^ Manganese dioxide nanosheets, for example, were discovered to react with the initially formed lithium polysulfides, binding them strongly onto the oxide’s surface. In turn, the bound lithium oligosulfides acted as a redox shuttle to promote and bind higher polysulfides and convert them, after reduction, to insoluble lithium sulfide.^174^ The sulfur/manganese dioxide nanosheet composite cathode with 75 wt % sulfur exhibited a reversible capacity of 950 mAh g^–1^ (with respect to sulfur) at 1 C rate and keeping 800 mAh g^–1^ after 200 cycles. Recently, the use of SACs with a theoretical 100% atom utilization efficiency is currently being explored as an attractive strategy to overcome the aforementioned limitations.^177^ The comparison of the different catalysts is not straightforward because they are generally tested under different conditions and unified evaluation criteria have not yet been applied.^174,178^ The performance evaluation in energy storage systems often involves diverse experimental conditions, configurations, and electrolyte components. Particularly in LSBs, the capacities are mostly provided with respect to the sulfur content in the cathode. While this metric is essential for monitoring sulfur utilization, it fails to reflect the useful capacity and practical performance of the cathode as an integrated electrode material at full mass level.

Cui et al. demonstrated that unmodified graphene has the weakest chemical binding energy to Li_2_S_6_, while SAC@Nitrogen doped graphene substrates increased the binding strength via developed metal–S and/or N–Li bonds.^179^ Recently, many research groups have successfully synthesized SACs and applied them in LSBs, demonstrating excellent performance for the conversion of polysulfides.^180^ Particularly, in order to better understand the advantages of SA catalysts in breaking the kinetic limit of such electrochemical reactions, Zhang et al. illustrated that Fe SAs supported on porous nitrogen-rich carbon matrices could highly accelerate the delithiation of the Li_2_S cathode and promote the reversible conversion processes for long-term operation.^181,182^ Electrochemical assessment combined with theoretical simulations showed that the Fe SAs high catalytic activity lowers the activation voltage of Li_2_S, promotes the Li_2_S delithiation, and facilitates the transport and deposition of Li^+^ in the electrode without sacrificing the rate performance. As a result, the research indicated that the use of SACs could produce Li_2_S/Li batteries with 588 mAh g^–1^ at the ultrahigh rate of 12 C along with a capacity reduction of 0.06% per cycle during 1000 cycles at 5 C, thereby opening a potential approach for practically competitive LSBs.

**Figure 12:**
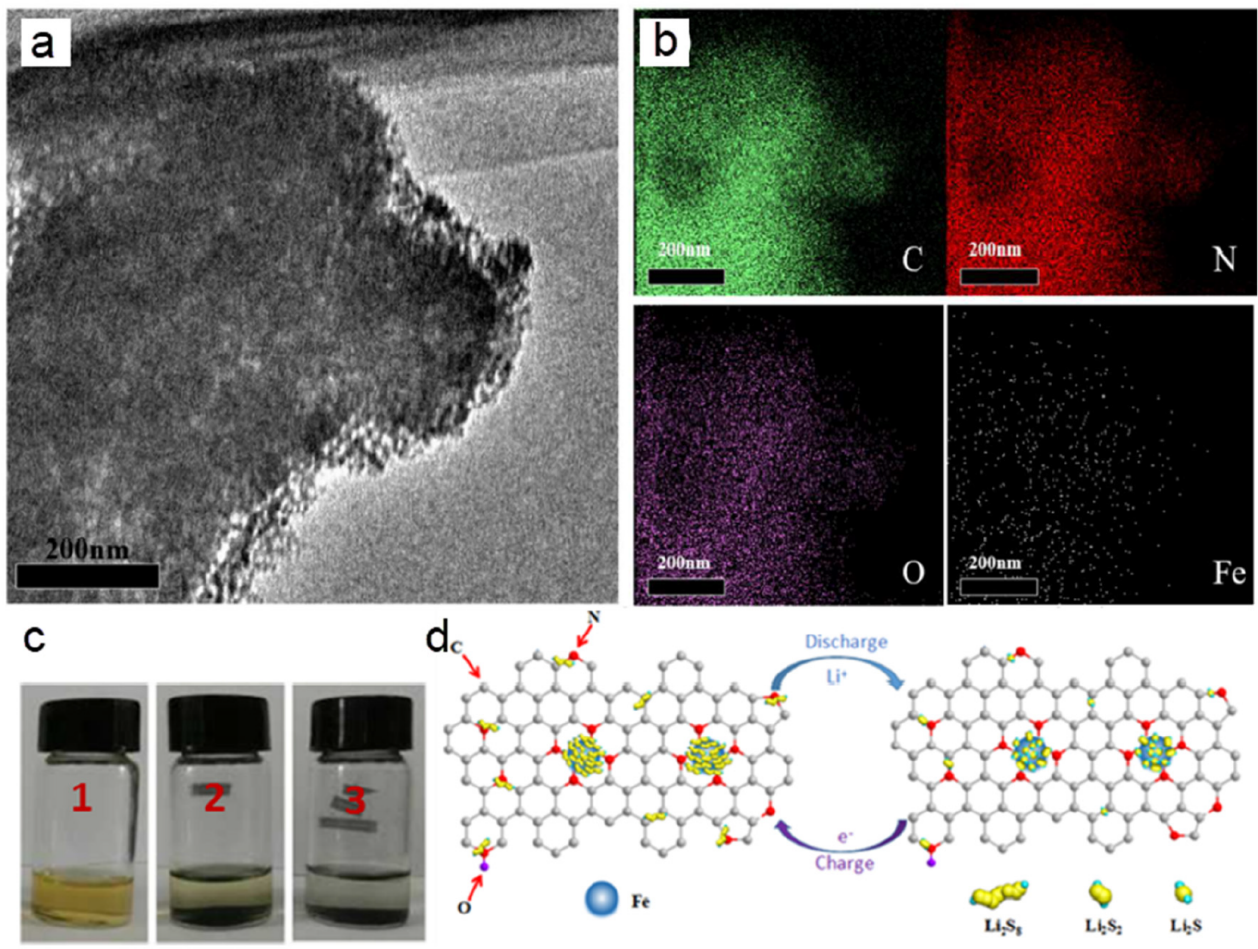
(a) TEM image and (b) corresponding element mappings of C, N, O, and Fe in the Fe-PNC. (c) Photograph showing the variation in color of the polysulfide solution (1) after adsorption by PNC (2) and Fe-PNC (3). (d) Schematic illustration of the conversion process of LPS on the Fe-PNC surface with single-atomic iron catalytic sites. Reprinted with permission from ref (183). Copyright 2018 American Chemical Society.

In another study, Fe SAs on nitrogen-doped carbon host materials (Fe-PNC) were used to accelerate the polysulfide redox conversion in LSBs.^183^ The catalyst was synthesized via a simple nanocasting method using iron phthalocyanine as iron precursor and *o*-diphenylamine as nitrogen precursor. After annealing in a N_2_ atmosphere, atomic-level iron was highly dispersed in porous nitrogen-doped carbon materials with iron–nitrogen coordinated active sites (Figure 12a, b). The initial catalyst (before the sulfur loading) contained 1.0 at. % Fe in atomic composition, as affirmed by XPS analysis. It was proposed that the Fe SAs could suppress the shuttling effect of polysulfides by enhanced interaction and adsorption ability. PNC and Fe-PNC were compared in the Li_2_S_6_/DOL/DME electrolyte, showing a visible decoloration of Li_2_S_6_ solution due to the substantially more efficient adsorption at the surface of the Fe-PNC sample (Figure 12c). Therefore, the Fe-PNC/S composites showed higher capacity and higher rate performance and cycling stability over PNC. The Fe-PNC/S composites showed an initial specific capacity of 1138.6 mAh g^–1^ at 0.1 C rate and maintain a discharge capacity of 427.1 mAh g^–1^ after 300 cycles according to the above-mentioned mechanism (Figure 12d). The capacity decay rate was 0.2% per cycle, and the high Coulombic efficiency of 99.0% is maintained during the discharge/charge process. These two works indicated the kinetic acceleration in the polysulfide conversion process by Fe-SACs to improve the reversible performance and weaken the shuttle effect.

Interestingly, the design of SACs with oversaturated Fe-N_5_ coordination structure (Fe-N_5_-C) for LSBs was studied theoretically and experimentally, extending the portfolio of catalytically active sites beyond the typical metal-N_4_ motif. It was found that the catalytic performance for polysulfide conversion was effectively regulated by the N-coordination number of the Fe sites.^184^ Fe SACs with Fe-N_5_ and Fe-N_4_ centers were controllably developed through the absorption–pyrolysis route of different predesigned conjugated micro-/mesoporous polymer (CMP) precursors. In the case of the Fe-N_5_ oversaturated sites, the chemical adsorption of the polysulfides was enhanced while boosting their catalytic conversion during redox reactions. Considering the particularly low Fe loading in the materials (i.e., lower than 0.02 mass%), one could hypothesize that it is mainly attributed to their catalytic function, promoted by the sorption of the Li_2_S_6_ species.

The impact of the coordination environment around the metal SA active site was similarly demonstrated in the case of a graphene support, wherein the structural deformation of the carbon skeleton was employed to modulate the local coordination sphere of the Fe-N_4_ sites.^185^ In particular, the authors took advantage of the defects in a two-dimensional graphene matrix which can form wrinkles due to the asymmetric distribution of bond lengths in the carbon lattice. This was achieved by hydrothermally treating GO to induce in-plane lattice defects through the removal of oxygenated carbon. Subsequently, the adsorption of iron precursor and carbonization lead to Fe-N_*x*_ sites embedded in wrinkled graphene vacancies. The deformation of the square-planar symmetry of the Fe-N_4_ configuration was shown both experimentally and theoretically that was responsible for the modulation of the electronic structure of Fe SAs. The atomic-level modified Fe-N_4_ active sites with the wrinkled geometric symmetry and electronic structure significantly enhanced the conversion kinetics of LiPS, especially in the most sluggish Li_2_S redox step. This led to efficiently accelerated polysulfide conversion rates, especially the Li_2_S redox reaction, which is known as the rate-determining step in Li-S chemistry. These works demonstrate the promising role of SACs in the development of LSBs but also the potential for further exploration and exploitation via fine-tuning the local atomic environment of the single metal atom active sites.

Apart from Fe, Co SACs embedded in nitrogen-doped graphene are employed in LSBs, in order to activate the surface-mediated process of LiPS via boosting the creation and breakdown of Li_2_S during discharge and charge.^186^ In a representative work, the combination of *operando* X-ray absorption spectroscopy (XAS) and first-principles calculations revealed that the Co-N-C coordination center serves as a bifunctional electrocatalyst to facilitate both the formation and the decomposition of Li_2_S in discharge and charge processes, respectively. Co was coordinated with pyridinic-type nitrogen atoms (rather than pyrrolic or graphitic moieties) in the graphene, as affirmed by the XANES results. The Co-N-C SAC was tested for its electrocatalytic activity for the reversible transformation of Li_2_S_6_ species, showing intense redox peaks (high currents) and very small voltage hysteresis between the cathodic and anodic scans, unlike the control samples, which did not contain Co in the N-pyridinic moieties or where the Co was deposited on the graphene without pyridinic moieties. Li_2_S_6_ species, which were initially added to the electrolyte, were reduced to Li_2_S or Li_2_S_2_ in the cathodic scan and then reversibly oxidized back to Li_2_S_6_ by the oxidation of Li_2_S and Li_2_S_2_ species at the anodic scan. In addition, efficient oxidation of Li_2_S_6_ to elemental S and reduction back to Li_2_S_6_ was also clearly recorded. The SAC was loaded with sulfur via a typical melt intercalation reaching to 90 mass% sulfur contents. The LSB half-cell with the sulfurized Co-N-C SAC-based cathode achieved a capacity of 1210 mAh g^–1^ and a rate reduction of capacity of only ca. 0.03% per cycle at 0.2 C. In 2023, Sun et al.^187^ constructed a Fe-Co diatomic catalyst supported by hollow carbon spheres to achieve the simultaneous high-efficient catalysis of the polysulfides and the Li_2_S decomposition. Thus, the Fe atom center boosts the acceleration of the discharge process, while the Co atom center favors the charging process. Theoretical calculations and experimental work showed that the bifunctional catalytic activity originates from the diatomic synergy between Fe and Co atoms. The assembled battery had a specific capacity of 1000 mAh g^–1^ at 1C (688 mAh g^–1^ at 5 C) with excellent cycling stability with a decay rate of 0.018% for 1000 cycles at 1 C.^187^

Recently, Pan et al. developed a composite catalyst consisting of Co nanoparticles and Zn SAs co-implanted in nitrogen-doped porous carbon nanosheets grafted with carbon nanotubes (Co/SA-Zn@N-C/CNTs) showing that dual active sites of Co and atomic Zn-N_4_ moieties not only strongly confine the polysulfides but also effectively catalyze the conversion reactions by lowering the energy barrier of the rate-limiting step (i.e., the transformation of Li_2_S_2_ to Li_2_S), while the N-doped porous carbon-grafted CNTs enabled a large surface area for more active site exposure and provided a fast electron/ion pathway.^188^ The strongly coupled Co nanoparticles and atomic Zn-N_4_ moieties induced a charge redistribution and a favorable electronic structure, making Zn SA less electron deficient, which could be probably another reason for the improved reduction of Li_2_S_2_ to Li_2_S, while the catalyst’s Gibbs free energy of the rate-limiting step became the lowest. Benefiting from the above-mentioned synergies, LSBs equipped with the Co/SA-Zn@N-C/CNTs-based sulfur cathode exhibited a high reversible capacity of 1302 mAh g^–1^ at 0.2 C and a low-capacity fading rate of 0.033% per cycle over 800 cycles at 1 C, keeping 700 mAh g^–1^. Zn SAs implanted in MXenes were also employed into a sulfur cathode, which could not only catalyze the conversion reactions of polysulfides by decreasing the energy barriers from Li_2_S_4_ to Li_2_S_2_/Li_2_S but also achieve strong interaction with polysulfides due to their high electronegativity on the MXene surface.^189^ In particular, the authors used Zn SAs implanted MXene (SA-Zn-MXene) layers derived from titanium aluminum carbide (Ti_3_AlC_2_) as sulfur host, and the sulfur was added in an aqueous solution. Using several experimental and theoretical characterizations, it was shown that the SA-Zn-MXene layers could efficiently facilitate the nucleation of solid-state Li_2_S_2_ and Li_2_S on their large exposed 2D surfaces. The material delivered a high reversible capacity of 1210 mAh g^–1^ at 0.2 C with good rate capability (640 mAh g^–1^ at 6 C).

**Figure 13:**
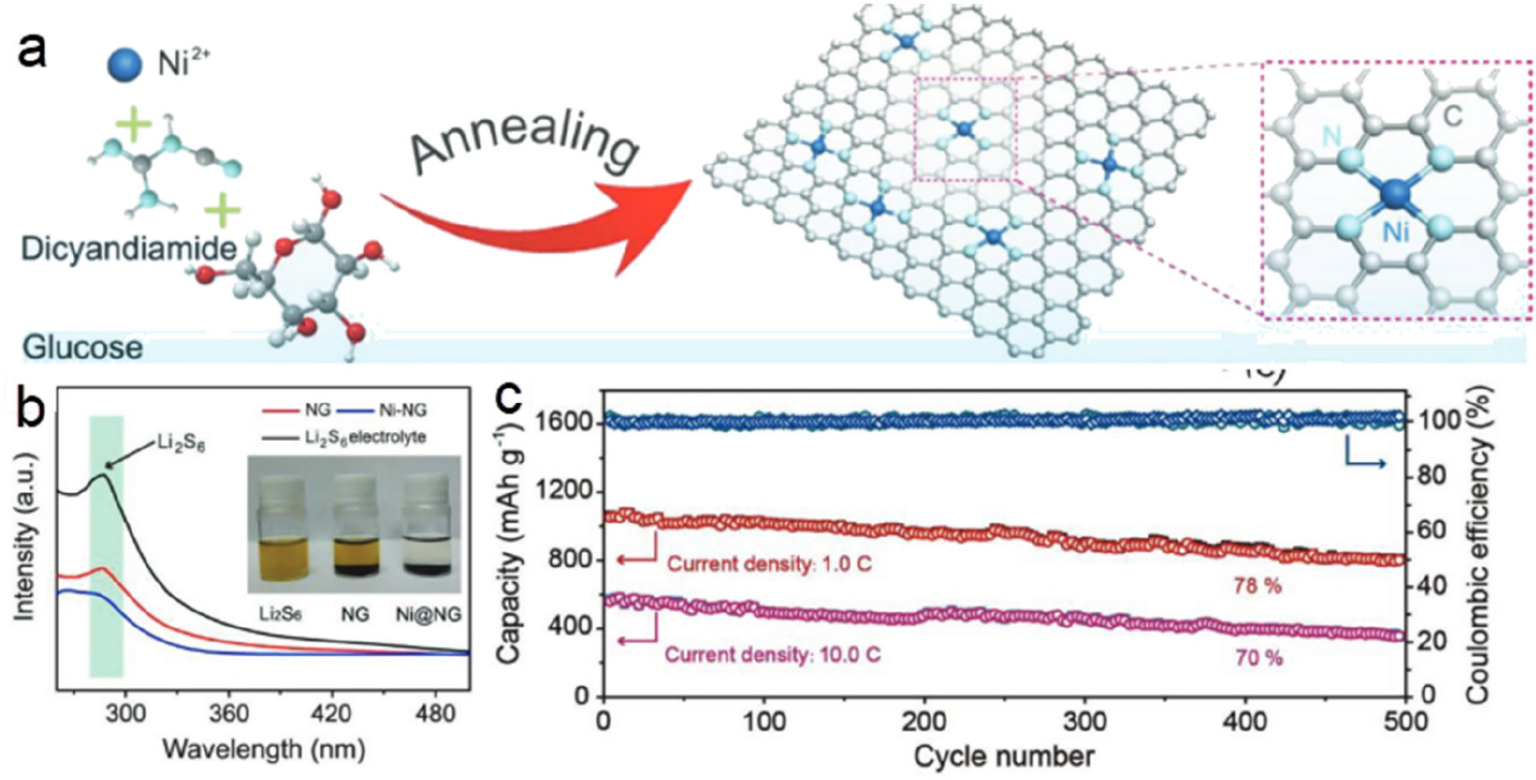
(a) Schematic illustration of the preparation of Ni@NG with the Ni–N4 sites. (b) UV–vis spectroscopy of Li_2_S_6_ solutions (the inset is the digital photographs of the Li_2_S_6_ solution after adding NG or Ni@NG for 6 h). (c) Cycling stability of the Li.S batteries based on the Ni@NG modified separator at 1 and 10 C, respectively. Reprinted with permission from ref (190). Copyright 2019 Wiley-VCH.

Besides functional cathode fabrication, SAC modified membrane separators have been employed to suppress the “shuttle effect” of LSBs by trapping Li polysulfides. In a representative work, Song et al. fabricated a modified separator with Ni SAs distributed in nitrogen-doped graphene (Ni@NG).^190^ The Ni@NG material was produced by a pyrolysis approach whereby the Ni SAs with high spatial density were confined into the N-doped graphene matrix with the assistance of sacrificial graphitic carbon nitride (g-C_3_N_4_) templates (Figure 13a). Furthermore, Ni@NG possessed a continuously cross-linked lamellar structure with the pore size of sub-micrometer and a homogeneous distribution of Ni, C, and N elements. The resistance to polysulfide shuttling by Ni@NG was intuitively presented by Li_2_S_6_ adsorption experiments verifying their particularly effective binding on the membrane (Figure 13b). Thus, when the Ni@NG was added into the Li_2_S_6_ solution and endured for 6 h, the color of the Li_2_S_6_ solution changed from bright yellow to almost transparent (Figure 13b, inset). Owing to its enhanced catalytic performance, the Ni@NG material was utilized to modify the separator of an LSB using the conventional sulfur/carbon composite cathode, and Li_2_S_6_ was added in the electrolyte. The LSB demonstrated a high reversible capacity of 826.2 mAh g^–1^ after 500 cycles at 1.0 C, corresponding to the capacity retention of 78%. A high-capacity retention of 70% was still achieved after 500 cycles (at 10 C) with a low-capacity decay of 0.06% per cycle (Figure 13c). Therefore, the results showed that the Ni@NG can not only immobilize the polysulfides but also accelerate their kinetic conversion during the charge and discharge process. More interestingly, Ni SAs with Ni-N_4_ structure on NG can retain their chemical stability even after long charge/discharge cycles.

At the same time, another multifunctional separator with nitrogen-doped graphene foam (NG) coating was reported, in which various SACs (Fe, Co, Ni) were structurally impregnated.^191^ The sample with the highest bonding affinity to polysulfides was the one with the Fe SAs (Fe_1_/NG). Thus, cyclic voltammetry showed an obvious reduction of the voltage hysteresis and higher current response corresponding to the improvement of polysulfides redox transformation in the case of the Fe_1_/NG-modified separators. The prepared LSBs delivered a similarly high discharge capacity with 891.6 mAh g^–1^ after 750 cycles at 0.5 C.

From a sustainability and economic point of view, sodium appears as a very attractive option, because of the analogous electrochemical properties with Li but much higher natural abundance of Na relative to Li. Thus, sodium sulfur batteries (NaSBs) with high energy density could meet the requirements for the electrification of the global transportation fleet.^192^ However, NaSBs additionally face fundamental challenges due to low cycling stability, slow reaction kinetics (the larger size of Na also contributing), and release of metal polysulfides from the cathodes, like in LSBs. Moreover, the current NaSBs operate at high temperatures of ∼350 °C, adding security risks, significant maintenance expenditure, and difficulty for deployment in mobile energy storage, overall underlining the need to develop room-temperature sodium-sulfur batteries (RT-NaSBs) for future practical applications.^193^ However, the discharge process in RT-NaSBs requires multiple steps, with the coexistence and interconversion of various polysulfide substances (Na_2_S_*n*_, 4 ≤ *n* ≤ 8).^25^ Moreover, subsequent electrochemical reactions are usually not complete, and the growth of Na dendrites (in the fashion of lithium batteries) adds more safety issues.^194^

**Figure 14:**
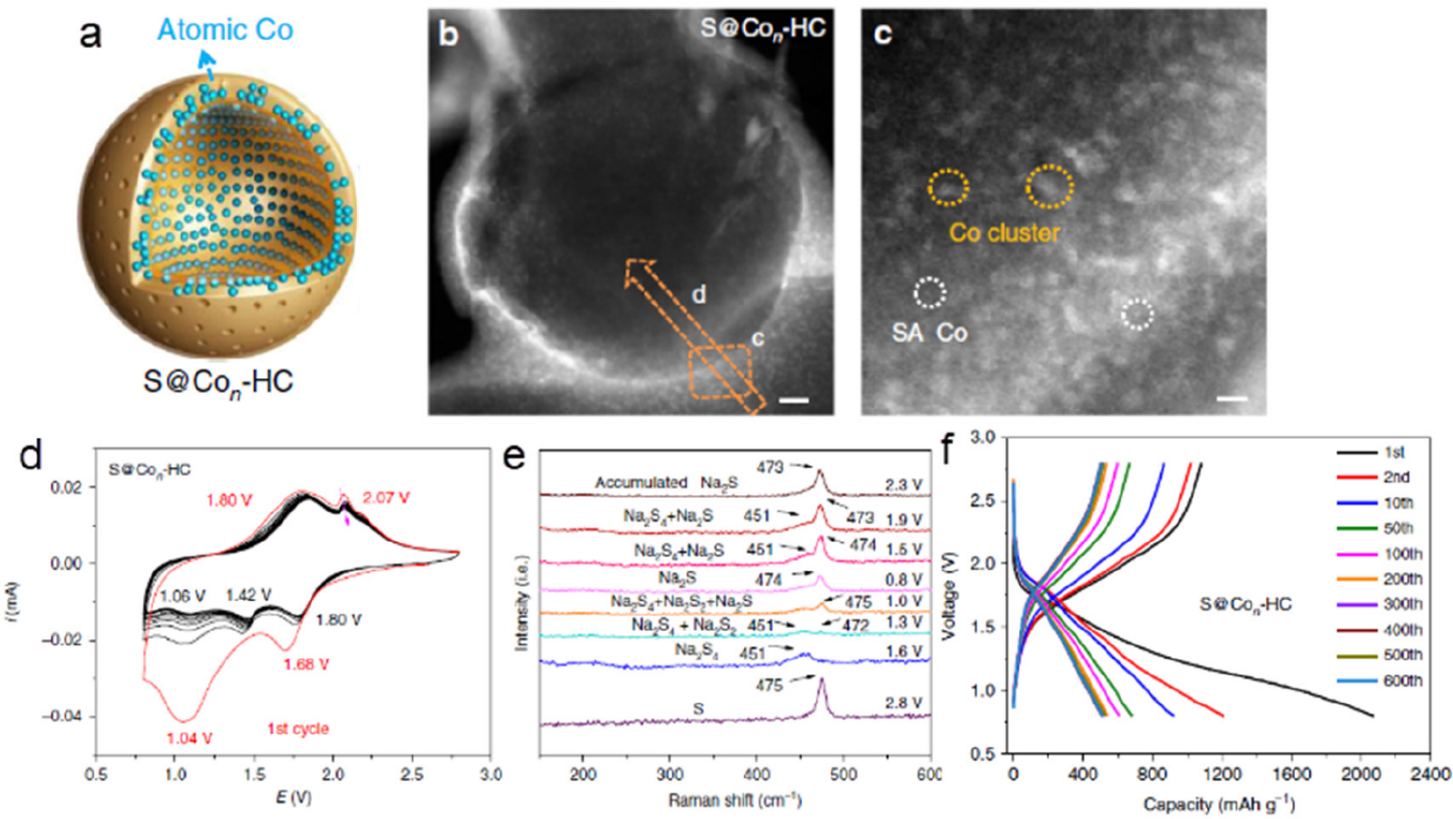
(a) Schematic illustration of atomic Co-decorated hollow carbon as a sulfur host material (S@Co_n_-HC). (b, c) HAADF-STEM images of atomic cobalt-decorated hollow carbon sulfur host (S@Co_n_-HC). Scale bar, 20 nm. (d–f) Room-temperature sodium-sulfur battery test: (d) Cyclic voltammograms, (e) *in situ* Raman spectra, and (f) discharge/charge curves of atomic cobalt-decorated hollow carbon sulfur host (S@Co_n_-HC) at 0.1 A g^–1^. Reprinted with permission from ref (196). Copyright 2018 Springer Nature.

Although the use of SACs in the field of RT-NaSBs appears to be promising, the relevant works are still limited. In a theoretical study, DFT calculations were used to elucidate the interactions of sodium polysulfides on the SACs surface.^195^ According to this study, pristine and nitrogen-doped graphene’s were found ineffective for anchoring and trapping polysulfides. However, the SACs embedded via monodispersed transition-metal atoms in nitrogen-doped graphenes (TM-NG where TM = Cr, Fe, and Co) exhibit adequate binding strength toward Na_2_S_*n*_ species. Thus, the SACs were predicted to serve as effective immobilizers for soluble Na_2_S_*n*_ to prevent shuttling. Furthermore, the electron-deficient SACs were found to substantially reduce the Na_2_S decomposition barrier, which demonstrates effective electrocatalysis in favor of complete reversible conversion of polysulfides. Wang et al. first proposed an efficient strategy to synthesize a range of SACs on a carbon matrix for several applications including RT-NaSBs.^36^ The route included the use of sodium *p*-toluenesulfonate (P-TSNa) to modify polypyrrole (PPy) fiber as side chain. The abscission of sodium ions from P-TSNa would cause the modification of PPy polymer self-attracting metal cations on the side chains due to the charge compensation. Owing to the staggered side chains, the absorbed metal ions were well spaced to keep them from aggregating during the carbonization process. Thus, based on the indiscriminate self-doping of various metal cations, a wide range of SA of metals on nitrogen-doped carbon frameworks could be prepared. In another important experimental work, hollow carbon (HC) nanospheres were employed as the sulfur host while atomic Co (and Co clusters) was embedded in a carbon shell (Figure 14a).^196^ The HC nanospheres acted as ideal frameworks, which allowed the initial anchoring of Co nanoparticles and subsequent S encapsulation. The ensuing composite (S@Co_n_-HC) was used as cathode active material to exploit the electrocatalytic activity of atomic Co and overcome the typical NaSB bottlenecks. The TEM and XPS results showed a combination of Co clusters and single atomic Co in C shells, and the sulfur content in the final material was found at 47 wt % (Figure 14b, c). The influence of Co atoms on the catalysis was further shown by two cathodic peaks at around 1.68 and 1.04 V at the cyclic voltammetry while the reaction mechanism, probed by *in situ* XRD and Raman spectroscopy (Figure 14d, e), confirmed the final reaction product to be the Na_2_S. These findings were translated to a high discharge capacity of 508 mAh g^–1^ at 0.1 A g^–1^ after 600 cycles when the final material was used as a cathode (Figure 14f).

The catalytic ability of Co atoms toward sodium polysulfides was reported by Wang *et al*. when they utilized Co SAs alongside with ZnS quantum dots (QDs) to decorate a high specific surface area hierarchical carbon as a novel sulfophilic carrier (Co_1_-ZnS/C).^197^ The SA Co dopants improved both the electronic conductivity of the cathodes and the dispersibility of ZnS QDs. Moreover, the Co atoms and ZnS QDs synergistically boosted the catalytic capability for both the conversion of NaPS intermediates and the reduction of Na_2_S product via polar–polar interaction enhancement. The final composite maintained a high reversible capacity of 640 mAh g^–1^ over 500 cycles at 0.1 A g^–1^, when employed as a cathode in NaSBs.

Rogach et al. reported a stable sulfur host based on a N, O-codoped carbon composite derived from a bimetallic Cu-Zn metal–organic framework, which ensured high sulfur loading (67 wt %). Most importantly, this composite also included copper SAs, with a high Cu loading of 8 wt %.^198^ The Cu SAC generated from the bimetallic (Cu and Zn) MOF precursor was employed as a cathode for NaSB with an initial discharge plateau starting at 1.65 V, indicating the reduction of short-chain sulfur (S_2–4_). Theoretical studies indicated that Cu SAs possess a high chemical affinity toward S_8_ ring molecules, thus facilitating the ring-opening reaction. As a result, the sulfur-loaded carbon framework containing Cu SAs exhibited a superior specific full mass capacity of 776 mAh g^–1^ with a high sulfur utilization after 100 cycles at 0.1 A g^–1^ and an excellent rate performance with 483 mAh g^–1^ at 5 A g^–1^. Although the majority of the sulfur-based batteries research focuses on LSBs and NaSBs, potassium–sulfur batteries (KSBs) are likewise emerging as a new attractive metal-sulfur battery system.^199^ Since potassium sulfur electrochemistry follows analogous principles with the alkali MSBs works on SACs, KSBs have started to attract interest. For instance, in a pioneering work, SACs were suggested to enhance the K-storage by favoring the conversion kinetics in the K-S chemistry;^200^ in one example, Co SAs on a nitrogen-doped carbon host exhibited substantially promoted K_2_S oxidation. The material was loaded with 56.8 mass % sulfur and was used as a KSB cathode, resulting in superior capacities, reaching 773 mAh g^–1^ and 535 mAh g^–1^ under 1 and 2 C, respectively.^201^ Additionally, the authors, using a combination of DFT computations and *in situ* synchrotron XRD measurements, confirmed a novel synergistic effect of dynamic Co-S and N-K interactions, which promoted the dissociation of the K–S bonds in potassium polysulfides, accelerating the oxidation kinetics of K_2_S.

**Figure 15:**
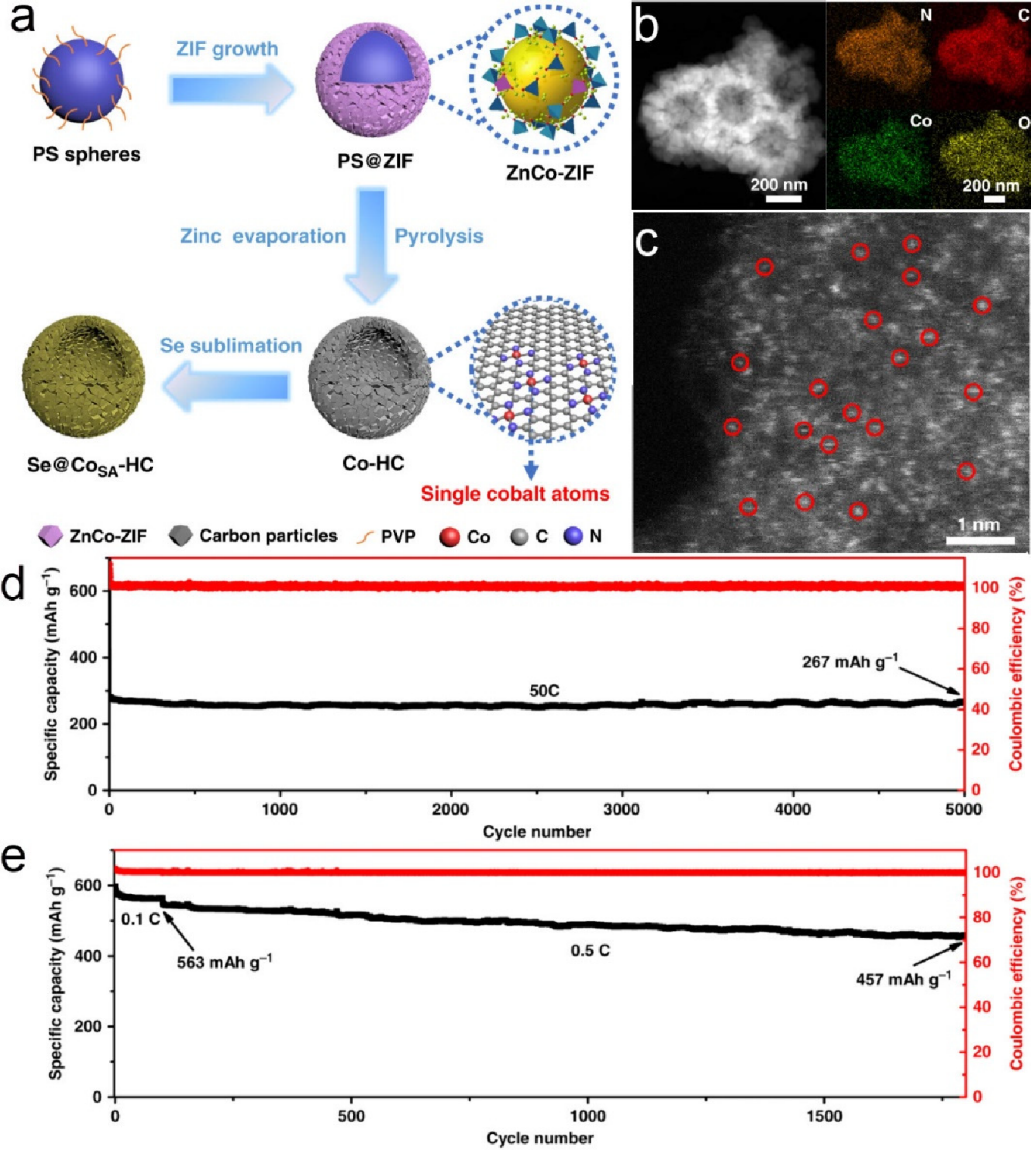
(a) Schematic illustration of the procedures for synthesizing cobalt single atoms/nitrogen-doped hollow porous carbon (Co_SA_-HC) particles. (b) STEM element mapping images. (c) Aberration-corrected HAADF-STEM image of the Co_SA_-HC SAC. (d) Long cycling performance and Coulombic efficiency at 50 C for 5000 cycles. (e) Cycling performance and Coulombic efficiency at 0.1 C for 100 cycles and then 0.5 C for 1700 cycles. Reprinted with permission from ref (206). Copyright 2020 Springer Nature.

Selenium belongs to the same group as sulfur in the periodic table, having similar chemical properties, and has been considered as an alternative battery cathode material for the recently reviewed lithium-selenium batteries.^202^ Se demonstrates a higher theoretical volumetric capacity (3253 mAh cm^–3^) and conductivity (1 × 10^–3^ S m^–1^) compared to sulfur, making it an attractive option as active material with better rate performance.^203^ Moreover, Li-Se batteries can perform better in the conventional carbonate-based electrolytes, where Li polysulfides are chemically unstable.^204^ However, S and Se share the same limitations, such as the Se shuttling issue associated with high-order lithium selenides (Li_2_Se_*x*_, *x* > 4) and the large volume expansion during the charge/discharge process, resulting in a low Se utilization, inferior capacity, and short cycle life.^205^ Apart from incorporating Se particles with conductive materials or encapsulating Se particles within porous carbon matrices, the application of SACs to enable highly effective cathodes for Li-Se batteries with superior rate capability and outstanding long-term cycling performance was proposed using a facile and straightforward approach.^206^ ZIF particles deposited on the surface of polystyrene spheres were converted into hollow structured carbon materials via a pyrolysis process (Figure 15a). Through the evaporation of zinc and tuning of the ratio between Zn and Co, a Co SA electrocatalyst on nitrogen-doped hollow porous carbon was developed. In the following, Se was embedded in the matrix via infusion/sublimation at 300 °C under Ar to obtain the final composite SAC (Se@CoSA-HC). This strategy resulted in isolated and positively polarized Co SAs, homogeneously distributed over the carbon matrix (Figure 15b, c) and integrated through the formation of Co-N_3_ and Co-N_4_ coordination motives. Moreover, the prepared matrix provided accessible storage sites and larger electrode/electrolyte interface area, while the volume expansion during lithiation was inhibited by the large internal void spaces. When applied as cathode materials for Li-Se batteries, the Se@CoSA-HC cathode exhibited a superior electrochemical performance and rate capability (311 mAh g^–1^ at 50 C (Figure 15d) and excellent cycling stability (267 mAh g^–1^ after 5000 cycles with a 0.0067% capacity decay per cycle at a current density of 50 C). Owing to the electrocatalytic effect from Co SAs, the electrode showed the lowest overpotentials compared to control materials without SAs and the SAs remained stable after 1700 cycles, delivering 457 mAh g^–1^ at 0.5 C (Figure 15e), confirming the persistent catalytic properties of the Co SAs. However, the Se loading of the product was relatively low (57 wt %), limiting its performance metrics with respect to full mass.

In summary, noble-metal free SACs have shown advantages in tackling contemporary challenges in LSBs and RT-NaSBs. Most commonly, research efforts have focused on the development of SAC/carbon electrode materials for hosting sulfur, developed by pyrolysis after impregnation or coordination; Fe and Co SACs being the most used and earth-abundant catalysts, while MOFs, graphene, or other N-doped carbons being the most used matrices. Regarding the electrochemistry, the rate and total cycling performance were generally improved by the presence of Fe, Co, and Ni SAs, due to the acceleration of sulfur and polysulfide species redox kinetics, as well as by the improved binding of metal polysulfide species. Such properties have proved to help in limiting the “shuttling-effect” and at the same time improving the kinetics of the polysulfides transformation reactions. However, many challenges still require optimization to attain high-performance sulfur electrodes. For practical applications, the electrolyte:sulfur ratio must be reduced to improve the final cell-level energy density, and the active material loading and the high aerial sulfur loading should also be increased. Moreover, facile, industrially feasible, and environmentally friendly synthetic strategies ought to be further developed and improved. Last but not the least, a better understanding of the SAC catalytic mechanisms will require the concerted efforts of teams with expertise on *in situ* and *operando* experimental characterization and theoretical calculations. Considering that the research for SACs in cathodes and functional separators has developed quite recently and is an active research field, there is still room for design developments toward composition and structural and functional aspects.

### 2.4 SACs in Supercapacitors

Supercapacitors (SCs) have received tremendous scientific attention as alternative electrochemical energy-storage devices to batteries, storing electricity via surface-controlled, fast redox reactions (pseudocapacitors) and ion adsorption on electrode porous surfaces, based on electrical double-layer capacitance (EDLC).^207−209^ Their power and cycling life are much higher than those of batteries, at the expense of the lower energy density.^210^ The energy storage efficiency of a SC is related to the efficient adsorption/desorption of electrolyte ions on the electrode surface.^211^ Typical SC electrodes are mostly fabricated from porous and activated carbon materials because they offer many active sites due to their high surface area.^210^ However, their commercialization in portable electronics and hybrid electric vehicles, and for competing in the broader market landscape of energy storage, will require higher energy storage densities. The single atom metal sites dispersed on carbon surfaces are prone to interact with ionic ligands under applied potential, which can also be regarded as a capacitive process.^212^ In this setting, SACs can be incorporated into carbon electrodes to catalyze surface redox reactions, thus increasing pseudocapacitance and leading to higher energy densities.^109,213^ Nevertheless, introducing SA metal species to modify carbon interfaces and promote supercapacitive performance has not been especially explored yet.

In one of the first reports in this field, Shan *et al*. incorporated K^+^ or Na^+^ SAs through hydrothermal reactions in ultrathin g-C_3_N_4_ decorated with MnO_2_ nanoparticles (K-CNM and Na-CNM, respectively).^213^ The authors found that doping with these SA species improved the conductivity of g-C_3_N_4_ and enhanced the mass transfer of electrolyte ions, thus contributing to the overall improved capacitance. More specifically, K-CNM had a specific capacitance of 373.5 F g^–1^ at 0.2 A g^–1^ in a three-electrode configuration using a neutral electrolyte, which was significantly higher than that of nondoped material. In addition, K-CNM had high cycling stability with 93.7% capacitance retention after 1,000 charge–discharge cycles at 1 A g^–1^. In another effort, a SA Co-doped carbon nanostructure (Co-POM/rGO) was synthesized by depositing polyoxometalates on the surface of a reduced GO aerogel at a mild temperature.^214^ When used as an electrode active material in an asymmetric solid-state supercapacitor with rGO as counter electrode, an energy density of 37.6 Wh kg^–1^ at a power density of 500 W kg^–1^ was recorded, with high capacitance retention of 95.2% after 5000 charge–discharge cycles, which was substantially better than that of the nonmetal doped system. These accumulated data offer motivation toward a strategy for designing SA metal-doped carbon nanocomposites for SC devices with improved capacitive properties. In the same year, Yu et al. embedded SA of Ni by a one-step pyrolysis of a predesigned MOF.^215^ The resulting Ni, P, and N tridoped hierarchical Ni/P/N/C composite was used as a symmetric supercapacitor demonstrating a higher specific capacitance than the nondoped system. Interestingly, the redox inactive Zn(II) was also studied for modifying N-doped carbon materials and applied as a supercapacitor electrode material;^216^ carbon materials with rich Zn-N_4_ moieties were obtained via Zn/benzamide copromoted formamide carbonization (Zn_1_NC). After a thermal treatment, Zn_1_NC retained a Zn content of 2.72 at. % and ∼12.51 at. % N, delivering a much higher specific capacitance, rate capability (retaining 65% capacitance at 100 A·g^–1^), and cycling stability (95.6% retention after 10,000 cycles at 10 A·g^–1^) than the same system without Zn doping. The CV curves showed a quasi-rectangular profile indicating a typical pseudocapacitive behavior. Furthermore, *operando* Raman showed the presence of Zn–OH bonding (OH originating from KOH electrolyte) during cycling, confirmed as well by theoretical simulations and which seemingly has a key role for the capacitance improvement. Thus, with Zn-N_4_ as the initial state, the charge polarization between Zn, C, and N was beneficial for the adsorption of OH^–^ ionic species, while the adsorbed anionic species could communicate via fast electron transfer around the Zn atom through the conductive carbon surface. Meanwhile, positive charges accumulated on the HO-ZnN_4_ surrounding area indicated that additional C and N sites besides Zn atom became available for anion storage, thereby simultaneously enhancing the EDLC and pseudocapacitance. This finding demonstrated that even the conventionally redox inactive Zn could contribute to boost the capacitive performance of N-doped carbon materials via the induction of charge polarization phenomena on the local and broader coordination environment of the carbon matrix.

Apart from SCs and pseudocapacitors, hybrid supercapacitors, such as metal-ion hybrid capacitors (MIHCs), hold particular interest in energy storage.^217,218^ MIHCs are mainly constructed with a high-energy battery-type anode and a high-power capacitor-type cathode so that they can take advantage (up to an extent) of both capacitor and battery properties. Moreover, the charge–discharge processes of the anode and cathode in MIHCs are performed in different potential ranges, thus broadening the operating voltage window, which efficiently enhances the energy density.^219^ In this regard, MIHCs possess attractive features such as high energy density and power density, as well as long cycle life, thus garnering increased attention in recent years.^220^ Among them, sodium-ion hybrid capacitors (SIHCs) hold great promise in large-scale energy storage by combining the merits of sodium-ion batteries and electrochemical capacitors. However, the kinetics and capacity mismatch between battery-type anode and capacitive-type cathode is still the Achilles’ heel of this technology. More specifically the battery-type anode suffers from sluggish redox reaction kinetics due to the relatively large ionic radius of Na cations, whereas the capacitor-type cathode with relatively low specific capacity limits the energy density of the devices.^221^

**Figure 16:**
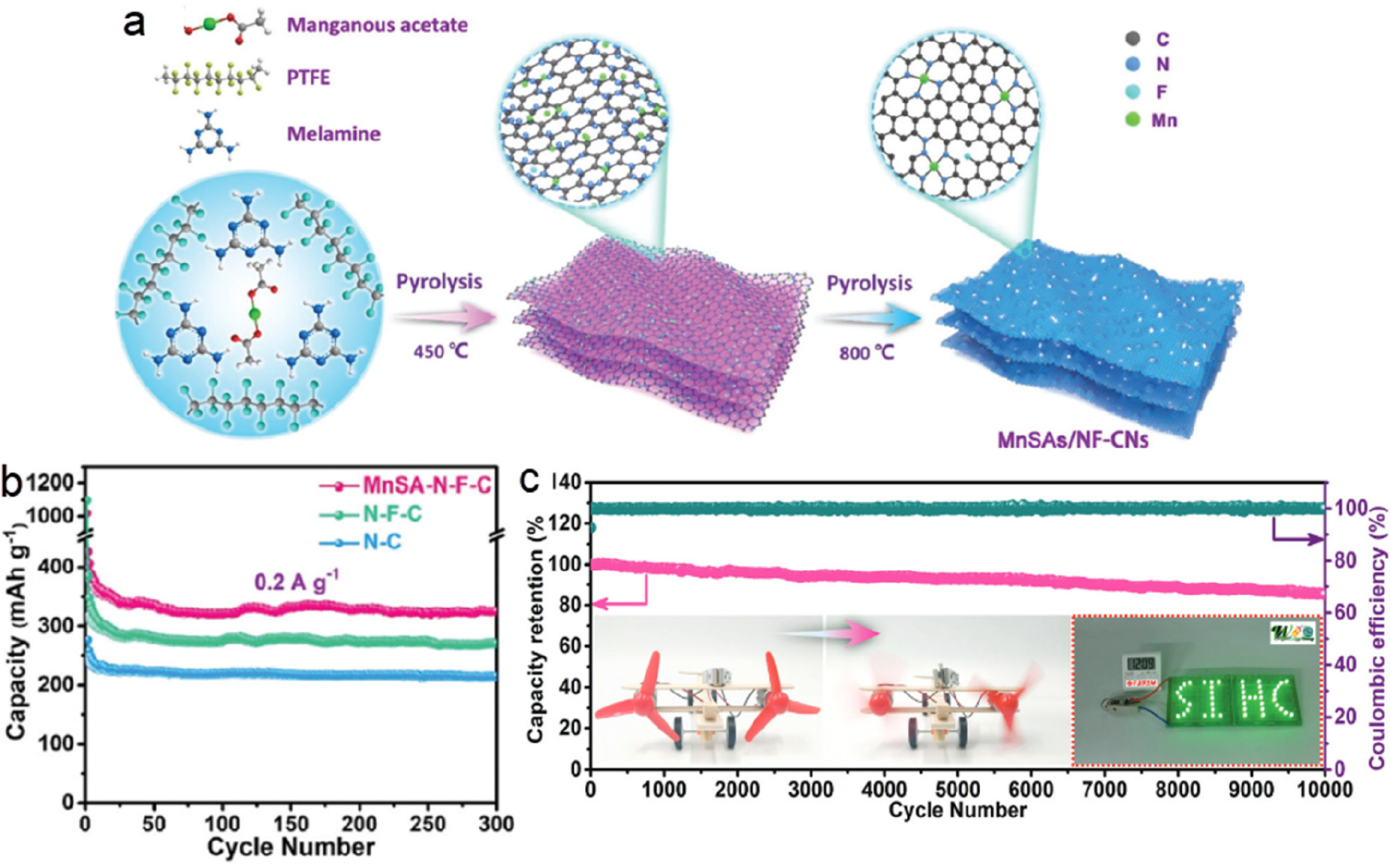
(a) Schematic illustration of MnSAs/NF-CNs synthesis. (b) Cycling performance of MnSAs/NF-CNs and reference materials. (c) Long-term cycle performance of the SIHCs at 1 A g^–1^. Reprinted with permission from ref (32). Copyright 2021 Royal Society of Chemistry.

To address these challenges, Xiang Hu et al. employed Mn SAs implanted within N and F codoped carbon nanosheets (MnSAs/NF-CNs) as both anode and cathode electrodes to accelerate the kinetics for Na^+^ storage and simultaneously improve the reversible specific capacity of anion adsorption/desorption.^179^ The MnSAs/NF-CNs material was prepared via a one-pot strategy by uniformly mixing precursors of Mn acetate, melamine, and polytetrafluoroethylene, followed by calcination in Ar (Figure 16a). The resulting material contained a large amount of atomically dispersed Mn species, and XPS N 1s spectra showed that when the percentage of the Mn-N_*x*_ species increased, the binding energy of the pyridinic N shifted, indicating that pyridinic N more favorably coordinates with Mn atoms to form the Mn-N_*x*_ moieties. The electrochemical properties of a set of such materials storage were initially studied as anodes for SIBs with the CV displaying a rectangle-like shape in the range of 0.5–3.0 V, suggesting a capacitive behavior. The cycling performance showed that the MnSAs/NF-CNs deliver a high reversible capacity compared to the reference materials (Figure 16b). The SIHCs full cell device was set up by employing the MnSAs/NF-CNs as both the battery-type anode and the capacitive-type cathode, presenting a long cycling stability with 85.2% capacity retention after 10 000 cycles at 1 A g^–1^ (Figure 16c). The results demonstrated that a SAC material with a highly conductive network and rich active sites can operate for both the reversible storage of Na^+^ and capacitive adsorption/desorption of the ClO_4_^–^ ions from the electrolyte. Thus, the Mn SAs played a key role in improving the electrochemical performance, offering a Janus feature for the application of MnSAs/NF-CNs as both anode and cathode in SIHCs, delivering high energy/power density.

In summary, it has been demonstrated that the incorporation of SACs can effectively enhance the electrochemical activity and overall performance of electrode materials in supercapacitors. Notably, SACs containing zinc, manganese, and nickel have exhibited remarkable improvements in both capacitance and stability by augmenting the conductivity of electrodes and facilitating the mass transfer of electrolyte ions. The capacitance and the generally low energy density in supercapacitors can be improved by abundant SA sites occupied by redox actives metals or metal pairs, where advancements in methodologies for stable and highly loaded SA-engineered systems will play a key role.^222,156^ In addition, SACs can reduce the resistance at the electrode–electrolyte interface, which is a major limiting factor in the rate performance of the devices, either via providing ionic charge transport pathways or via populating the density of states at the Fermi level, thus boosting electronic conductivity. Finally, SACs, being atomically small active sites, when immobilized on a conductive carbon matrix (or other matrices), may facilitate the electronic cross-talk between the charge carriers adsorbed at the surface of the electrode materials and the current collectors in a much more efficient way than in the case of the bulkier nanoparticles.

## 3 ELECTROCHEMICAL REDUCTION OF CARBON DIOXIDE WITH TRANSITION METAL SACs

The electrochemical CO_2_ reduction reaction (ECO2RR), which enables recycling of waste CO_2_ into carbon-neutral fuels and high value-added products via utilizing sustainable electricity, has received considerable attention over the past decade.^223^ In this section, the progress achieved over the past few years on the CO_2_ electrolysis based on transition metal SACs will be summarized. Further, we will scrutinize how the local structures of different catalysts provide distinct chemical coordination environments, which in turn determine the active sites, alongside elucidating a set of criteria that need to be fulfilled for transition metal SACs to deliver a high performance on a practical scale. Finally, a future perspective will be provided on SACs in terms of scale-up synthesis, high-value products, and higher energy efficiencies for the overall CO_2_ reduction process development.

The ECO2RR is a highly complex operation, as it entails multiple proton/electron transfer steps, implying an important variety of reaction intermediates and products. Table 2 lists the equilibrium potentials for the ECO2RR regarding commonly reported electrochemical products. The presented ECO2RR standard potentials were calculated via the Gibbs free energy of reaction using gas-phase thermochemistry data and, for aqueous products, Henry’s Law data from NIST, with values originating from a previous report.^224^

**Table 2:** Standard Equilibrium Reduction Potential (E^Ø^) for Major Products in Electrochemical CO_2_ Reduction

Product name	Reactions	E^Ø^ (V vs RHE)
carbon monoxide	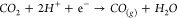	–0.10
formic acid	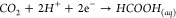	–0.12
methane	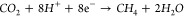	0.17
methanol	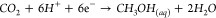	0.03
ethylene	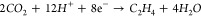	0.08
ethanol	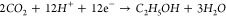	0.09
acetic acid	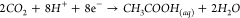	0.11
n-propanol	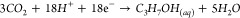	0.10

### 3.1 Reaction Pathways and Mechanisms of Electrocatalytic Reduction of CO_2_

**Figure 17:**
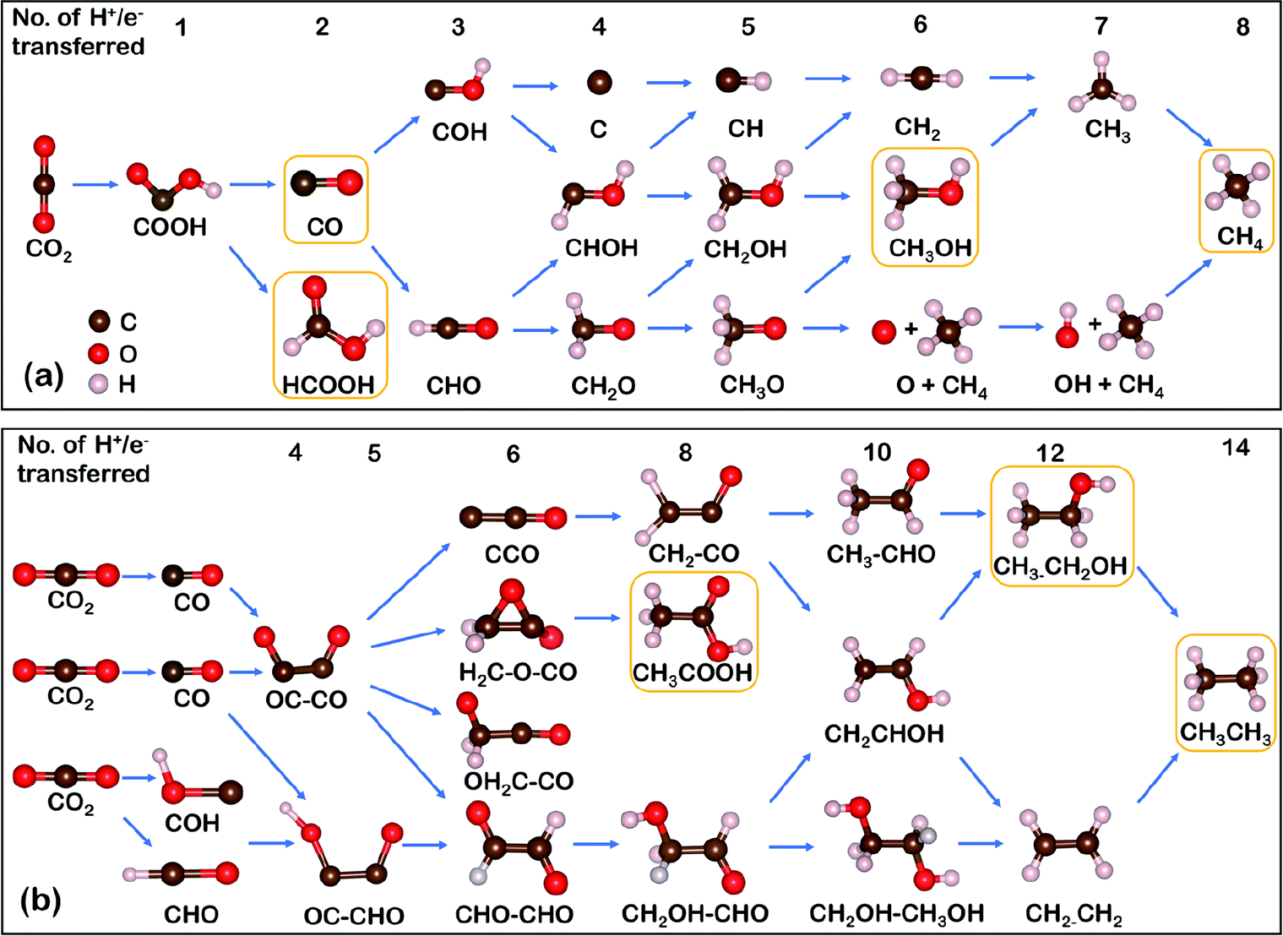
Overview of possible ECO2RR reaction pathways with the number of H^+^/e^–^ transferred: (a) the pathways toward C1 products via CO and HCOOH intermediates; (b) the C2 products formation through CO dimerization. Reprinted with permission from ref (226). Copyright 2022 Royal Society of Chemistry.

The proton–electron coupling transfer (PECT) theory is already well-established in the ECO2RR domain.^225^ The PECT process involves a multiple-step proton (H^+^) and electron (e^–^) transfer, which is profoundly influenced by the pH of the electrolyte. Generally, the first step is the activation of the CO_2_ molecule, followed by the stepwise transfer of H^+^ or e^–^, further generating diverse intermediates and products; Figure 17 represents the product formation based on the number of electrons/protons being transferred. As depicted in Figure 17a, the CO_2_ molecule is adsorbed to form *COOH via a one PECT process - a critical step for determining the selectivity of SACs due to the competition of *H adsorption in aqueous solution. A two-electron (2e^–^) transfer is most frequently observed for SACs with CO or HCOOH as the final products. Further reduction will generate the products, like methanol and methane, requiring six and eight H^+^/e^–^ pair transfer, respectively. Figure 17b illustrates the possible pathways toward C2 products where the CO dimerization is the rate-limiting step. Interestingly, the coupling of *CHO/*COH and *CO corresponds to an alternative route which has been theoretically demonstrated as achievable on bimetal SACs.^226,227^ In principle, all subsequent evolutions of species could come from these C–C coupling processes. Notably, stabilized C2 intermediates can be further transferred to *n*-propanol via CO insertion, but it happens rarely over single metal SACs.^226^

Transition metal SAs are usually coordinated in a well-defined matrix. The interest in transition metal SACs toward the ECO2RR basically arises from three main aspects. First, their extremely high atom efficiency: the single atom is exposed as the reactive site. Second, the low coordination state of the single atom directly affects the electronic and geometric properties, which leads to distinctive catalytic activities. Compared to the bulk catalysts, atomically dispersed 3d block nonprecious metals (especially Fe, Co, and Ni) exhibit high selectivity toward the ECO2RR over the hydrogen evolution reaction (HER). Third, SACs’ coordination into a regular framework is easily simulated using DFT calculation models. Such simplicity of the active regions makes them ideal platforms for designing and high-throughput screening of catalysts. From the reaction aspect, SACs also follow the PECT process. The most notable change is that a SA is mainly a single active center due to the high surface energy density in the surface metal ion exposure. Accordingly, it accelerates surface reaction bonding, resulting in a one-step reaction rather than multistep reactions. Thus, most SACs primarily follow a 2e^–^ transfer route, producing CO as the main product with an excellent performance, comparable to traditional noble metal clusters.^228^ Although many studies have attempted to understand the reaction pathways and interpret the activity and selectivity trends, the reaction mechanism is still disputed, and so is the correlation between the structure and the catalytic performance.

### 3.2 Coordination and Active Site Formation of SACs

The CO_2_ activation originates from the molecular catalysis, with the molecular electrocatalyst being predominantly in the form of metal-four nitrogen coordination (M-N_4_).^229^ Thus, SACs mainly share this particular coordinated structure similar to that of conventional molecular catalysts. Two significant points of concern that can be outlined are (i) the intrinsic properties of individual metals and (ii) the unique local coordination environment, including central elements’ valence state and coordination number.

Conventionally, SACs are catalysts with atomically isolated metal active sites stabilized on a support. Among it, the unit of a single-atom transition metal coordinated in a carbon matrix has garnered considerable attention in electrocatalysis for its superiority. In addition, some heteroatom species in the carbon matrix such as N, S, and P provide anchoring vacancies for the metal atoms and modify the electronic structure in coordinated donors.^230^ Among them, the most frequently used approach is the nitrogen-coordination strategy. Well-identified units, such as M-N_*x*_ moieties, have been commonly reported as active sites, especially the unique M-N_4_ local structure.^231^ In greater detail, these M-N_*x*_ structures function in a manner similar to metal porphyrins, representing coordinated molecular catalysts with a metal–nitrogen coordinated center. Consequently, research focusing on the immobilization of molecular catalysts could harness the strengths of both fields, integrating the precision of molecular catalysis with the versatility of heterogeneous surfaces. For example, Sargent and co-workers reported a low coordinated Cu-complex generated from copper(II) phthalocyanine. The introduction of carbon nanoparticles functioning as moderators facilitated the reduction of the copper coordination number. These low coordinated Cu-complexes exhibited superior performance in CH_4_ generation within a highly alkaline solution. This highlights that a low coordination number promoted C–C coupling and the formation of multicarbon products.^232^ Other strategies to obtain low-coordinated metals and metal complexes, such as porphyrin- and phthalocyanines-based electrocatalysts, through metal-anchoring involve the immobilization of the metal center on the surface through different methodologies.^233,234^ Most investigated molecular catalysts are earth-abundant transition metals comprising Fe,^234^ Cu,^235^ Co,^236,237^ Ni,^238^ and Zn.^239^ Besides the heterogenization of molecular catalysts, some metal-nitrogen-carbon covalently bonded (M-N-C) structures such as porphyrinic triazine framework have also been reported for the ECO2RR.^240^ Similarly to a molecular catalyst, the active unit for the CO_2_ reduction on M-C structures is the ligand-coordinated complex (e.g., porphyrins, phthalocyanines, cyclams, phosphines, polypyridines).^234,241^ The most used complexes are the metal-porphyrin/protoporphyrins comprising four pyrrole groups attached to each other by methine bridges where the metal center is coordinated with four N atoms. If the reaction follows the proton–electron separated transfer mechanism, these ligands could also participate in protonation or CO_2_ activation, and the product could be tuned to multiple carbon products with a synergetic effect. Additionally, this has also inspired researchers to develop supports to anchor heterogeneous structures with this similar metal coordination (M-N_4_).^242^

Correspondingly, the performance from individual carbon supported SACs varies markedly in the ECO2RR because of the differences in the number and nature of the carbon or nitrogen bonds to the metal center.^243^ Owing to their similarity to the molecular M-N_4_ moiety, the M-N_4_ units in the SACs with a planar coordination architecture are believed to be the catalytic active sites.^244^ Besides the central metal, the effect of SACs’ coordination environment on the electrochemical performance has been recently discussed by Guan.^245^ The correlation between the coordination configurations and diverse reduction products is discussed and particularly emphasized by Lei and co-workers.^246^ They believe that the structure of the metalloporphyrin-like active sites is the active site and that complex hydrogen donors also play a significant role in tuning the product selectivity.

Regarding metal-coordinated units, both theoretical and experimental studies have verified that modified carbon materials with isolated single metal-nitrogen sites (M-N_*x*_-C) hold great potential as effective catalysts for ECO2RR, displaying promising performances, as indicated by several studies.^247,248^ However, definitive conclusions about the active sites are still pending. To this end, theoretical chemistry based on DFT calculations can help to elucidate the active site formation. Luo and co-workers extended the standard fixed electron quantum mechanics to build the constant potential grand canonical methodology to describe the kinetics at a fixed potential;^249^ the predicted onset potential (at 10 mA cm^–2^) followed the order Ni-N_2_C_2_ > Ni-N_3_C_1_ > Ni-N_4_, which was in agreement with the experiments. Liu and co-workers developed a general two-step approach to synthesize a model nickel SAC with a precise structure enabling study of the effect of the local environment on the ECO2RR performance.^250^ They built a well-defined Ni-N_4_ moiety on a conductive carbon support prepared by linking Ni- tetra(amino)phthalocyanine to CNTs via C–C coupling, which exhibited a superb performance in the ECO2RR, with the rate-determining step for the ECO2RR being *CO_2_^–^ + H^+^ → *COOH. Further, Liu and Zhao explained the crucial roles of charge capacity and hydrogen bonding on the basis of ab initio molecular dynamics and a “slow-growth” sampling approach.^251^ The authors assert that a hybrid coordination environment (with one nitrogen and three carbon atoms) for the nickel atom is the most active site. The M-N_1_ site showed the highest activity and selectivity for the ECO2RR, highlighting the crucial roles of charge capacity and hydrogen bonding. This can help to elucidate the mechanisms of other heterogeneous electrocatalysts in an aqueous solution and improve catalyst designs. Zhang and co-workers reported a universal approach synthesizing transition metal SACs, including Cr, Mn, Fe, Co, Ni, Cu, Zn, or their combinations.^252^ The key is to use metal complexes (M^2+^ ions coordinated by 1,10-phenanthroline ligands), which facilitates the formation of “porphyrin-like” single metal atom sites on carbon black.

SACs derived from MOFs always showed extraordinary properties in the electrocatalysis field.^253^ MOFs are directly pyrolyzed under inert gas, without the addition of support substance, forming carbon frameworks to anchor SACs. Consequently, transition metal (M) coordinated with N-doped carbon materials - M = Mn, Fe, Co, Ni, Cu - could be synthesized by direct thermal pyrolysis of MOF or COF. In this manner, particular sacrificing ligands are required, such as melamine,^254^ urea,^255^ dicyandiamide,^256−258^*o*-phenylenediamine,^259^ and their copolymers.^260^ The most used MOFs are zeolitic imidazolate-based ones such as ZIF-8^229,261,262^ and ZIF-67.^263,264^ In some cases using Zn-MOFs as precursors, the high-temperature calcination under inert gas will evaporate the Zn species which served as protective ions, limiting the agglomeration of the active metal.^262,264^ The organic ligands are transferred into a conductive N-doped carbon during the pyrolysis, with the metal atoms being anchored on the nitrogen sites. It should be noted that the coordination number and the metal–support bond play important roles in determining the final local atomic structures.

In summary, the activation of CO_2_ is rooted in molecular catalysis, particularly in the presence of metal-four nitrogen coordination (M-N_4_) in molecular electrocatalysts. The SACs share this coordinated structure, notably the M-N_*x*_ moieties within a carbon matrix, which have gained significant attention. Heteroatoms like N, S, and P in the carbon matrix create anchoring vacancies for metal atoms and modify the electronic structure through coordinated donors. Notably, metal-porphyrins/protoporphyrins, with their metal centers coordinated with four N atoms, have shown significant promise in CO_2_ reduction, offering diverse carbon products due to their ligands’ participation in protonation and CO_2_ activation. Furthermore, the structural similarity to metalloporphyrin-like active sites in SACs, specifically the M-N_4_ units in the planar coordination architecture, is believed to be catalytically active. The effect of the SACs’ coordination environment - the central metal and ligands - on the electrochemical performance has been the topic of recent discussions, shedding light on the product selectivity modulation. Theoretical and experimental studies, particularly those involving modified carbon materials with isolated single metal-nitrogen sites (M-N_*x*_-C), show significant promise in the electrochemical reduction of CO_2_, as evidenced by several studies. However, definitive conclusions about the active sites will require further research. The synthesis of SACs from MOFs has also garnered attention, utilizing pyrolysis under inert gas to anchor transition metals (M = Mn, Fe, Co, Ni, Cu, Zn, Bi, etc.) to N-doped carbon materials, often resulting in yielding exceptional electrocatalytic properties. Sacrificial ligands play a crucial role in this process, as they convert into conductive N-doped carbon during pyrolysis, with metal atoms being anchored onto the nitrogen sites. It should be noted that the coordination number and metal–support bond significantly influence the final atomic structures. The exploration of these intricate catalysts opens avenues for advancing CO_2_ electroreduction technologies.

### 3.3 SACs Categorized by Product of Electrochemical CO_2_ Reduction

Thanks to their extraordinary activity and low cost, the most studied transition metal SACs for the ECO2RR encompass Mn, Fe, Co, Ni, Zn, and a nitrogen codoped carbon matrix with metal-N_*x*_ active sites. Table 3 summarizes the catalytic systems based on SACs and their performance for the ECO2RR. Further subsections classify the ECO2RR reactions with respect to their products.

**Table 3:** Catalytic Performance of Transition Metal SACs in ECO2RR^a^

Catalyst	Electrolyte	Main product	Faradaic efficiency (%)	Overpotential (V)	Current density (mA cm^–2^)	Ref
Fe-N-C	0.1 M KHCO_3_	CO	∼80	0.5	∼4	(265)
Mn-N-C	0.1 M KHCO_3_	CO	∼80	0.45	∼3	(265)
FeMn-N-C	0.1 M KHCO_3_	CO	∼85	0.4	∼2	(265)
Fe-N_4_-C	0.1 M NaHCO_3_	CO	91	0.5	4.5	(261)
Ni-N_4_-C	0.5 M KHCO_3_	CO	99	0.71	28.6	(257)
Ni-N_4_-C	0.5 M KHCO_3_	CO	∼70	0.9	∼10	(262)
Ni-graphene	0.5 M KHCO_3_	CO	∼90	0.64	∼12	(266)
Ni-N-carbon	0.1 M KHCO_3_	CO	∼96	0.65	∼10.5	(255)
Ni-N-carbon	1 M KHCO_3_	CO	∼97	0.53	∼30^b^	(267)
Ni(I)-N-graphene	0.5 M KHCO_3_	CO	97	0.61	∼24	(247)
Ni-N-graphene	0.5 M KHCO_3_	CO	∼90	0.45	∼12.5	(268)
Ni-N-carbon black	0.1 M KHCO_3_	CO	∼98	0.7	∼1.5	(252)
Ni-N-carbon black	0.1 M KHCO_3_	CO	∼90	0.55	25	(269)
Ni-N-carbon dot	1 M KHCO_3_	CO	∼90	0.6	40^c^	(270)
Ni-N_4_-F-C	0.5 M KHCO_3_	CO	∼95	0.67	∼25	(271)
Ni-N-carbon sheet	0.1 M KOH + 0.5 M K_2_SO_4_	CO	∼55	0.7	∼1.5	(263)
Ni_2_-N_4_-carbon	0.5 M KHCO_3_	CO	∼96.6	0.7	∼9	(272)
Fe-N-carbon	0.1 M KHCO_3_	CO	87	0.38	∼1.3	(255)
Fe-N-carbon	0.1 M KHCO_3_	CO	∼93	0.48	∼2.5	(229)
FeN_4_-O-C	0.1 M NaHCO3	CO	∼99	0.73	∼9	(273)
Fe-N-carbon	0.5 M KHCO_3_	CO	∼90	0.27	∼8	(224)
Fe-N_5_-graphene	0.1 M KHCO_3_	CO	∼97	0.35	∼1.8	(254)
Fe-N-P-C	0.5 M KHCO_3_	CO	∼97	0.32	∼5	(274)
Co-N-carbon	0.1 M KHCO_3_	CO	∼45	0.5	∼1.3	(229)
Co-N-carbon	0.5 M KHCO_3_	CO	∼90	0.53	∼17.5	(264)
Co-N_5_-C	0.2 M NaHCO_3_	CO	∼90	0.63	∼5	(275)
Co-N_4_-MWCNT	0.5 M KHCO_3_	CO	99	0.49	24.8	(276)
Co-N-3D carbon	0.1 M KHCO_3_	CO	91	0.8	67	(277)
Cu-N_2_-C	0.1 M KHCO_3_	CO	∼75	0.4	∼1	(256)
Bi-N_4_-C	0.1 M NaHCO_3_	CO	∼97	0.4	∼3.9	(258)
Mn-N_4_-Cl-C	0.5 M KHCO_3_	CO	97	0.49	∼10	(278)
Cd-N_4_-C	0.5 M KHCO_3_	CO	∼92.1	0.628	∼5	(279)
Mg-C_3_N_4_	0.5 M KHCO_3_	CO	∼90	1.078	∼32^b^	(280)
Cu-Al_2_O_3_	1 M KOH	CH_4_	62	1.37	153.0^c^	(281)
Zn-N_4_-C	1 M KHCO_3_	CH_4_	85	–1.8 V vs SCE^d^	39.7	(282)
Sb-N_4_-C	1 M KHCO_3_	HCOOH	96	0.68	∼16^c^	(283)
Bismuthene	0.5 M KHCO_3_	formate	∼90	1.05	∼100	(284)
Cu-N_4_-carbon fiber	0.1 M KHCO_3_	CH_3_OH	44	0.93	∼90	(285)
Cu-N_4_-carbon	0.1 M CsHCO_3_	C_2_H_5_OH	55	1.29	∼16.2	(286)
Cu-N doped carbon	0.1 M KHCO_3_	CH_3_COCH_3_	36.7	–0.36 V vs RHE	∼0.4	(287)

a **Note**: The overpotential and current density are mainly calculated according to a long-term electrolysis in a conventional H-cell (with the priority to choose the data with lowest overpotential and high FE) under the optimal conditions shown in the literature for comparisons.

b The value is the partial current densities of CO collected at the relevant potential.

c The results were collected via a gas diffusion electrode in a flow cell electrolysis.

d SCE refers to saturated calomel electrode.

#### 3.3.1 Electrochemical CO_2_ Reduction to CO

In 2015, Strasser and co-workers demonstrated that the M^δ+^-N_*x*_ centers are active for electrochemical CO_2_ reduction.^265^ Most interestingly, they found that Fe single atoms coordinated on N-doped carbon (Fe-N-C) were able to achieve more than 80% of FE for CO and a maximum current density of 35 mA cm^–2^, which is comparable to the activity of metallic Au foil. It was proposed that the M-N_4_ type of moieties accounts for the formation of CO, particularly thanks to the role of nitrogen. Since then, the design of various M-N_*x*_ local structures has become feasible in the electrochemical community, thus enabling the exploration of other metal SACs for ECO2RR. Many SACs show product selectivity toward CO, indicating that the usual competition with HER is well limited. One of the significant advantages of SACs, compared to non-SACs, is believed to originate from the inherently isolated active sites.^288^ In an aqueous electrolyte, the activation of CO_2_ on single atomic sites has been experimentally confirmed, and the competing H_2_O adsorption may be suppressed on these isolated sites. In contrast, nanostructured clusters have continuous surfaces on which the simultaneous adsorption of CO_2_ and H_2_O may occur.^289^ On the other hand, most of those nanostructured metal catalysts are polycrystalline, where the different facets often exhibit different binding strengths toward CO_2_, H_2_O, and their intermediates. Apparently, the metal coordinated moiety in SACs could dramatically tune the reaction selectivity, and the M-N_4_ site is responsible for the CO_2_ reduction activity. In contrast, when the metal is materialized in the form of nanoparticles, it will favor a multiple proton transfer coupling reaction in sequence. Early experimental studies have shown that the trend for the CO_2_-to-CO conversion reactivity in the M-N-type of SACs follows the order Ni ≥ Fe ≫ Co in the coordination configuration of M-N_4_.^259^

**Figure 18:**
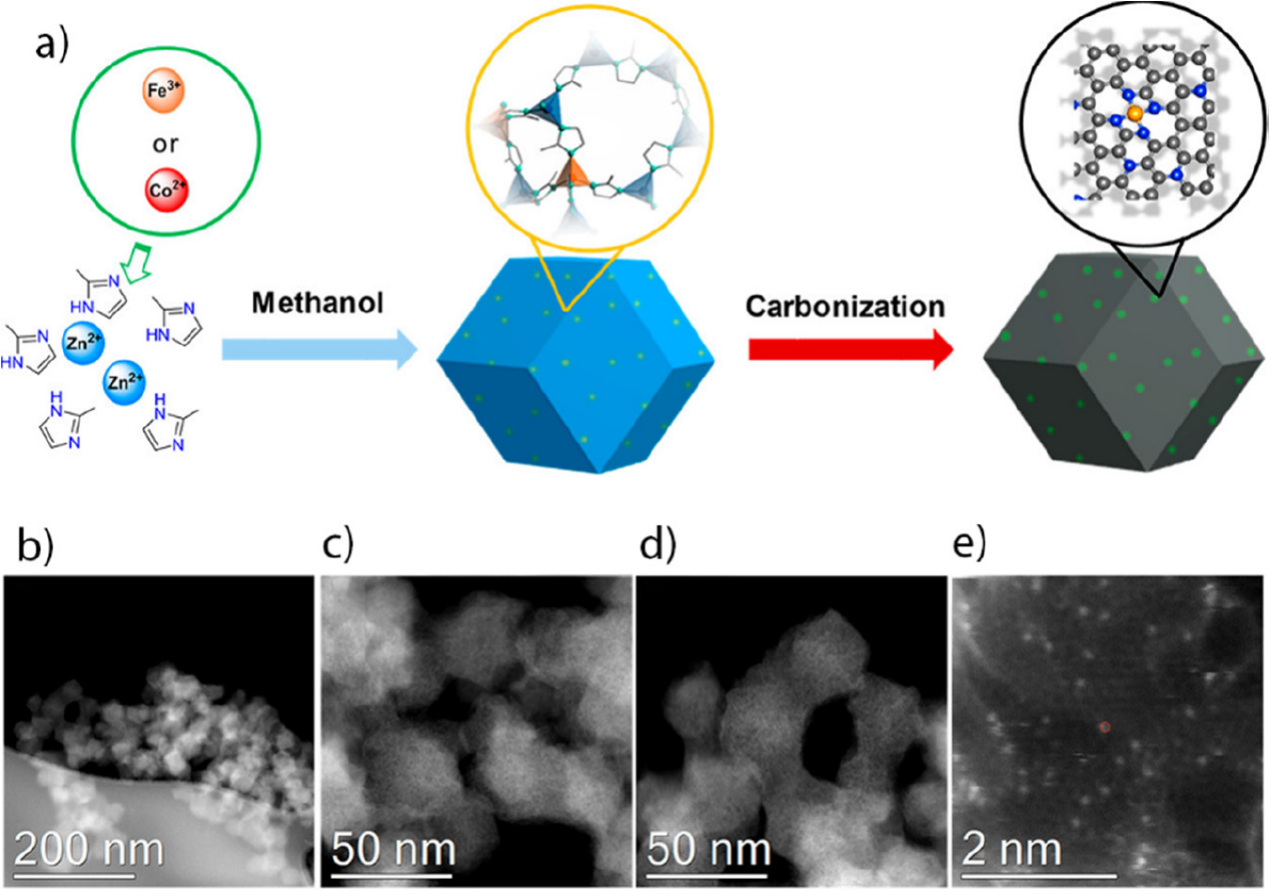
(a) Schematic illustration of the synthesis of single-atomic Co site on N-doped carbon (Co-N-C) catalysts. (b-e) The HAADF-STEM images of Co-N-C catalysts. Reprinted with permission from ref (229). Copyright 2018 American Chemical Society.

The research on Co SACs in the ECO2RR started with the heterogenization of cobalt complexes^290^ and was inspired by cobalt molecular catalysts such as cobalt phthalocyanine and cobalt tetraphenylporphyrin. The immobilization of these active complexes on a substrate executes the heterogeneous CO_2_ reduction process. For example, Han et al. constructed a well-defined, two-dimensional metal-porphyrin complex and deployed it as a platform to perform a coordination-dependent study on the ECO2RR.^291^ The FE of CO could reach 96% at an overpotential of 0.5 V, and the DFT calculations showed that the extra nitrogen bonding significantly reduced the free energy barrier of the *COOH formation by 0.76 eV, compared to the bare Co center complex without coordination to the support. The authors argued that the activity was improved by direct nitrogen coordination from the bottom layer, which elevated the d-orbital energy level of the metal atom. These findings implied that it is possible to adjust the structures and properties of the Co coordination to make the CO_2_-to-CO conversion favorable on the supported Co heterogeneous local structure. In order to generate sufficient Co-N_4_ local structures, Li and co-workers opted for Co-acetate tetrahydrate and 1,10-phenanthroline complex [Co(phen)_2_(OAc)_2_] to synthesize a Co-CNT SAC.^276^ The obtained catalyst displayed a CO FE of 99.4% with a CO current density of 24.8 mA·cm^–2^ at a low overpotential of 0.49 V tested in an H-type cell, and a CO FE over 90% was obtained in a flow cell within a wide current density window (50–600 mA·cm^–2^). Besides the development of the Co-N_*x*_ structure inspired by the molecular catalyst, significant effort has been devoted to exploring the Co-N-C coordination effects through simple complexes. Pan et al. synthesized an atomically dispersed Co catalyst on a N-doped carbon, named as Co-N-C, from a ZIF-8 precursor, which is a zinc-rich MOF.^229^ The synthetic approach is depicted in Figure 18a. The authors concluded that rather than physically absorbed in the pores of ZIF-8, the Co ions are in nodes that chemically bond with 2-methylimidazole, thus stabilizing the atomic Co dispersion over the precursors. In addition, Zn also functions as spacers to disperse Co to avoid agglomeration during the high temperature treatment. As shown in Figure 18b–e, the high-resolution HAADF-STEM demonstrated the existence of atomically dispersed Co sites even after pyrolysis, which were both located at the edge sites and embedded in the carbon matrix. The DFT calculations indicated that the edge-hosted M-N_2+2_ sites bridging two armchair-like graphitic layers were the possible active moiety for the ECO2RR, which is different from the traditional M-N_4_ sites embedded into a compact carbon plane.

**Figure 19:**
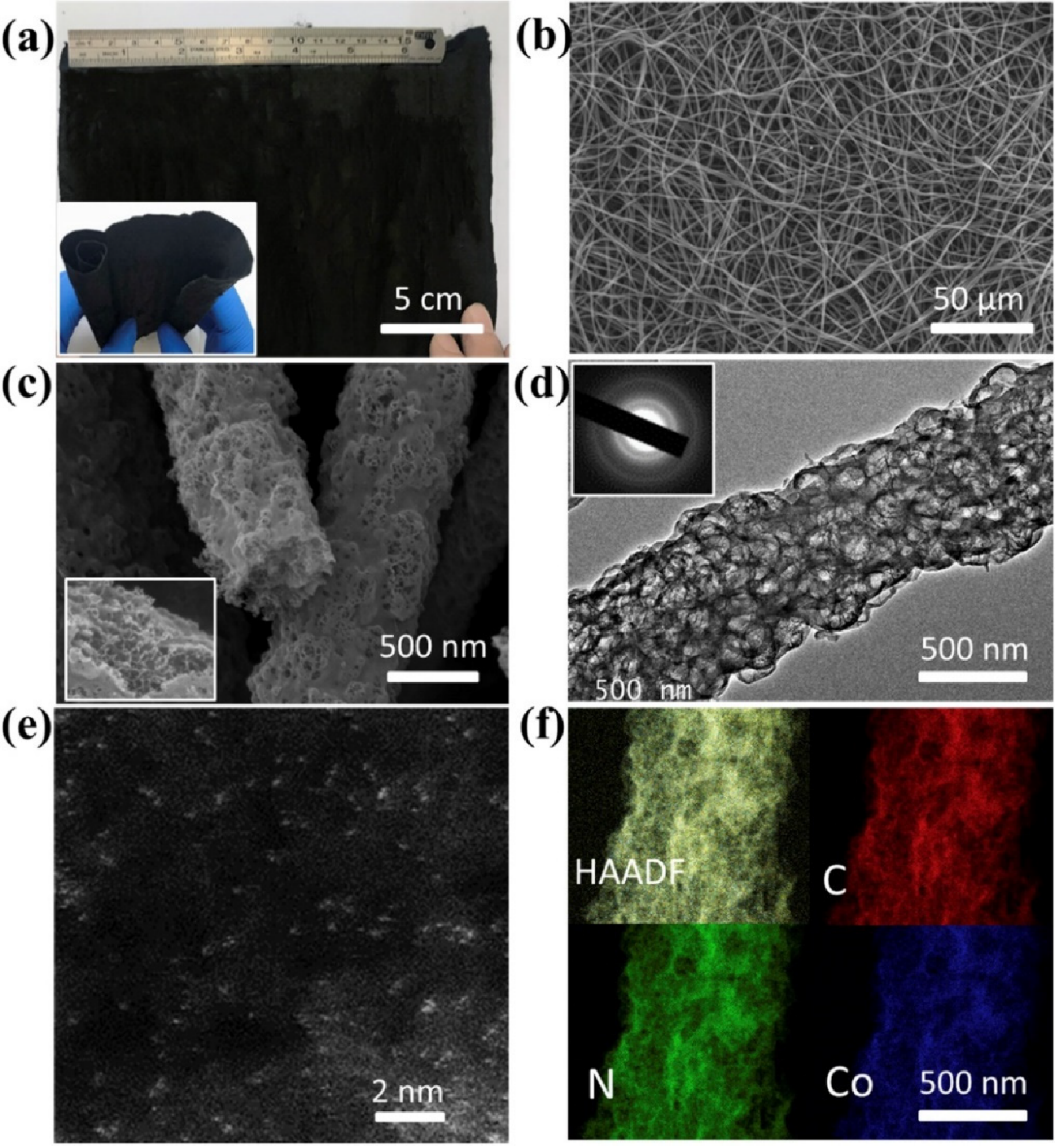
(a) Images of a piece of flexible single-atomic Co site on a high-yield carbon membrane, named the Co_SA_/HCNFs electrode, and the characterization of as-synthesized CoSA/HCNFs. (b, c) FE-SEM, (d) HR-TEM, and (e) aberration-corrected HAADF-STEM images of Co_SA_/HCNFs; the inset of (d) shows the SAED patterns and (f) EDX mapping of a single Co_SA_/HCNFs nanofiber. Reprinted with permission from ref (277). Copyright 2020 Elsevier.

In contrast, Chen and co-workers designed a Co-N_5_ unit serving as an active center for the ECO2RR toward CO; they achieved a FE of CO > 90% in the potential range between −0.57 and −0.88 V.^275^ Further, the DFT calculations suggested a near-zero free energy barrier (0.02 eV) for the COOH* formation on Co-N_5_. Therefore, it culminated in the rapid formation of COOH* and further quick desorption of CO. On another note, Wang et al. synthesized a series of atomically dispersed Co-N-C electrocatalysts with different M-N_*x*_ coordination number (from 2 to 4) by pyrolyzing Co/Zn ZIFs at controlled temperatures.^264^ The catalytic activity for the CO production was found to follow the trend Co-N_2_ > Co-N_3_ > Co-N_4_. The Co-N_2_ centers provided a current density of 18.1 mA cm^–2^ and 94% CO selectivity with an overpotential of 0.52 V. Further, the DFT calculations demonstrated that Co-N_2_-C, compared to Co-N_4_-C, has lower energy barriers for activating the CO_2_ molecules in order to form *CO_2_^–^, which benefits the CO production. Additionally, He and co-workers developed a one-pot method for scalable production of Co SACs-decorated carbon membranes via a electrospinning technique, which could be used directly as a gas diffusion electrode (GDE) (Figure 19).^277^ The as-synthesized electrode (Co_SA_/HCNFs) showed a 3D interconnected network structure formed by randomly aligned carbon nanofibers (Figure 19a–d). The isolated atomic dispersed Co sites recognized by HAADF-STEM as white spots evidence the formation of SACs (Figure 19e, f). Further, the electrode exhibited higher than 90% FE for the CO production and >200 mA cm^–2^ CO partial current densities. Those hierarchically porous, cross-linked, and free-standing carbon structures generate large electrochemical active surfaces and abundant channels for electron and reactant transportation, thus leading to high exposure of effective Co SAs as active sites for the ECO2RR. This strategy might represent a significant step toward a scalable synthesis of SACs and their eventual industrial applications.

**Figure 20:**
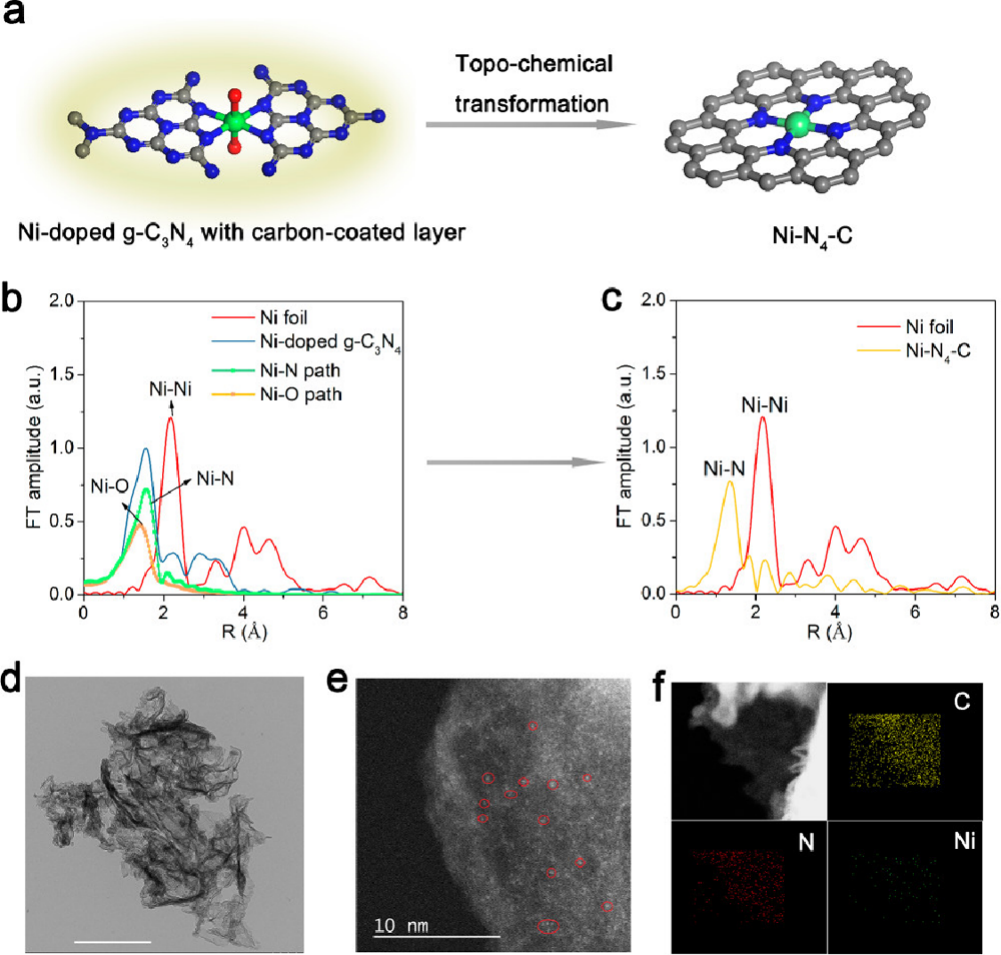
(a) Schematic illustration of the topo-chemical transformation strategy for the synthesis of single-atomic Ni site on a N doped carbon named Ni-N_4_-C (Ni atoms, green; N atoms, blue; C atoms, gray; O atoms, red). (b) Fourier transform of the Ni K-edge EXAFS spectra of Ni-doped g-C_3_N_4_. (c) Fourier transform of the Ni K-edge EXAFS spectra of Ni-N_4_-C. (d) TEM image of Ni-N_4_-C. Scale bar is 500 nm. (e) HAADF-STEM image of Ni-N_4_-C. (f) Element mapping images of Ni-N_4_-C. Reprinted with permission from ref (257). Copyright 2017 American Chemical Society.

Similar to Co, the ECO2RR activity for Ni can be tuned when it is in a single-atom form, whereas bulk Ni and Ni oxides are more favorable for the HER/OER under alkaline conditions for water electrolysis. Although the Ni-N_4_/Ni-N_*x*_ structures have been widely investigated in the ORR field for H_2_O_2_ production, there is still particular research interest in the ECO2RR. Xie and co-workers developed a topochemical transformation method to form a unique Ni-N_4_ structure.^257^ The synthesis strategy is depicted in Figure 20a, where Ni-doped g-C_3_N_4_ is employed as a precursor. With regard to the local chemistry, the Ni–N_4_ structure was formed where the Ni site is associated with N atoms, as confirmed by the Fourier transform of Ni K-edge EXAFS spectra (Figure 20b–c). After pyrolysis in Ar, the Ni atoms are highly dispersed in a N-carbon sheet, as shown by HAADF-STEM and an element mapping analysis (Figure 20d–f). The as-synthesized catalyst achieved a maximum FE of 99% for the CO production at −0.81 V with a current density of 28.6 mA cm^–2^. Additionally, operated from −0.5 to −0.9 V, the catalyst was also able to keep the FE over 90% for CO formation.

**Figure 21:**
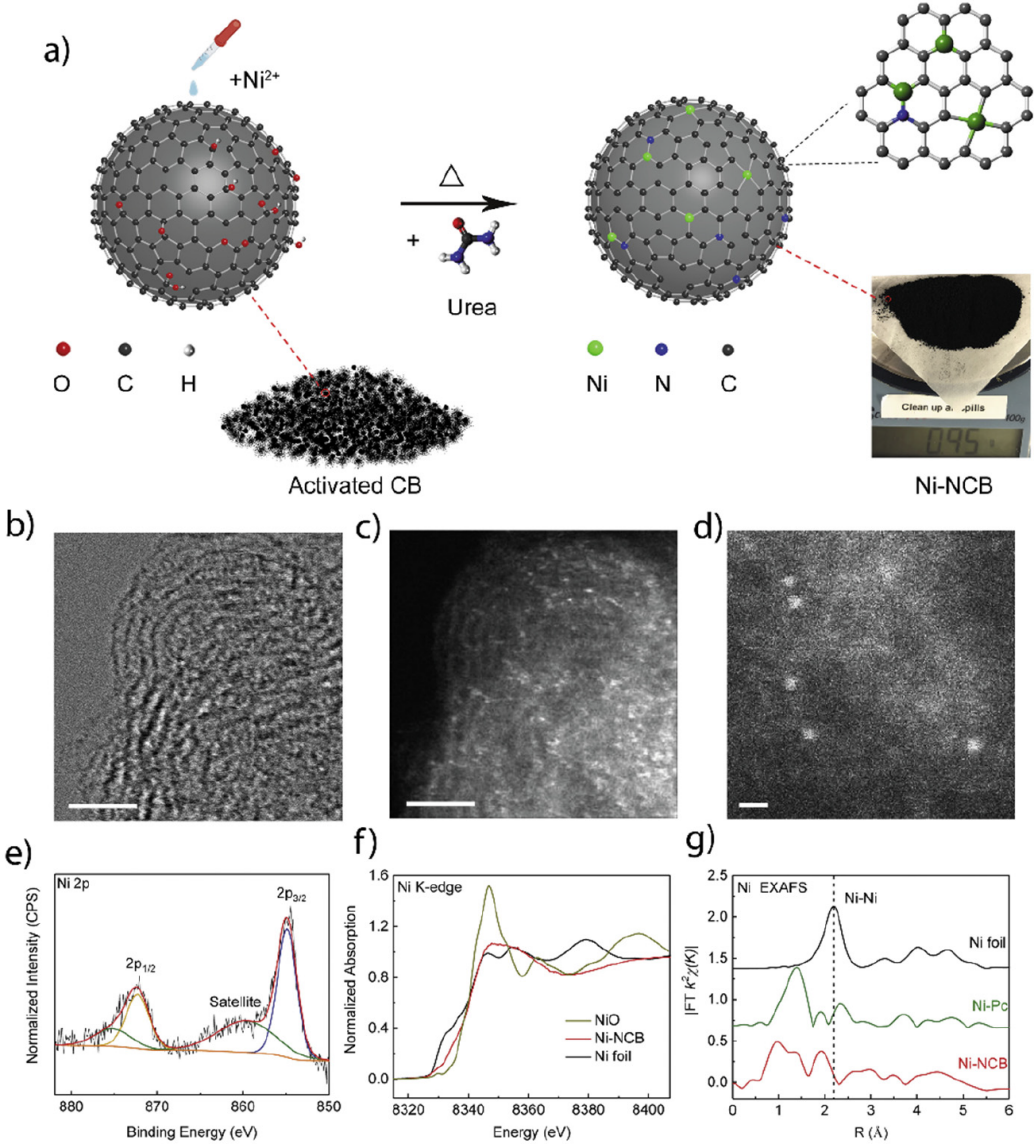
(a) Schematic description of a single atomic Ni site on N doped carbon black (Ni-NCB) and structure characterization. (b) Aberration-corrected bright-field STEM image. Scale bar is 2 nm. (c) Aberration-corrected HAADF-STEM image. Scale bar is 2 nm. (d) Zoom-in HAADF-STEM image showing the isolated Ni single atoms confined in a carbon matrix as represented by these high-contrast dots. Scale bar is 0.5 nm. (e) Ni 2p region XPS spectra, (f) Ni K-edge XANES spectra, and (g) Fourier transform of Ni K-edge EXAFS spectra of Ni-NCB and references. Reprinted with permission from ref (269). Copyright 2019 Elsevier.

Wang and co-workers introduced a simple approach to form Ni SACs anchored in graphene layers.^266^ The as-synthesized Ni single atomic sites were generated via impregnation and reduction, starting from graphene oxide as the catalyst support. This catalyst showed a high CO FE of 95% with an optimal current density of 50 mA cm^–2^. Reported by the same group, Zheng et al. further developed a scalable method for synthesizing Ni SACs embedded in a commercial carbon black.^269^ In this work, they highlighted the simplicity of their synthesis procedure. As depicted in Figure 21a, Ni^2+^ ions were first adsorbed onto water-soluble carbon black. Most likely, they were binding to the oxygen-containing functional groups or defect sites. After mixing with urea (serving as nitrogen source), the obtained composite was then pyrolyzed, generating Ni_SA_-N-C with ∼0.27 wt % in Ni loading, as determined by inductively coupled plasma atomic emission spectrometry. The authors concluded that the Ni atoms in a nitrogen-doped carbon black support were highly dispersed (Figures 21b–d) and had a higher oxidation state than metallic Ni and a lower one than NiO (shown in Figure 21e–g), as determined by XANES and EXAFS analysis. Notably, the selectivity of CO reached ∼99% with a current density ∼80 mA cm^–2^.

Further, Yang et al. expanded the ligand-mediated method, ensuring a large-scale Ni SACs production in kilogram-scale over a carbon support.^252^ The obtained catalyst (Ni_SA_-C) exhibited 2.5 wt % Ni loading and showed excellent activity and stability for the CO formation (FE of 98.9% at −1.2 V vs RHE). Besides employing liquid solvent for a SAC precursor preparation, Pan et al. developed a direct solid-phase pyrolysis route to synthesizing Ni-N-C and Fe-N-C SACs.^255^ The acquired Ni SACs showed a higher CO selectivity (∼96%) and stability for as long as 10 h of electrolysis. Similarly, Jiang and co-workers reported a one-step solid pyrolysis to form Ni SACs on a microwave-exfoliated GO.^268^ The obtained catalyst achieved as high CO FE as 92.1% with a mass activity of 53.6 mA mg^–1^ for an overpotential of 0.59 V. Further, the DFT calculations demonstrated that the edge-anchored unsaturated three nitrogen coordinated Ni sites exhibited better activity than the Ni embedded in-plane structures. Liu and co-workers reported low-valent Ni-site SACs over a nitrogenated graphene substrate via a solid pyrolysis approach^247^ These Ni SACs exhibited a monovalent Ni (I) atomic center with a Ni-N coordination number of 2, which was identified as the catalytically active site. The DFT calculation combined with the XANES analysis demonstrated that the Ni3dx^2^-y^2^ orbital promoted the delocalization of the unpaired electron and spontaneously transferred the charge from Ni (I) to the carbon 2p orbital, thus favoring the formation of CO_2_^δ−^ species. Consequently, this reduced the energy barrier for the electrochemical CO_2_ reduction. Another approach from Han et al. reported Ni-N_4_ active sites by F-doping via the pyrolysis of polytetrafluoroethylene (PTFE).^271^ The authors declared that the high catalytic performance with a CO FE > 95% in a wide potential range was due to the presence of F-coordination. Furthermore, the DFT calculation revealed that the formation energies of *COOH significantly decreased after the F-doping in the local coordination, which can be ascribed to the asymmetrical charge distributions caused by the F-doping. These findings prompted the research work focusing on other element-coordinated environments to enhance the activity.

**Figure 22:**
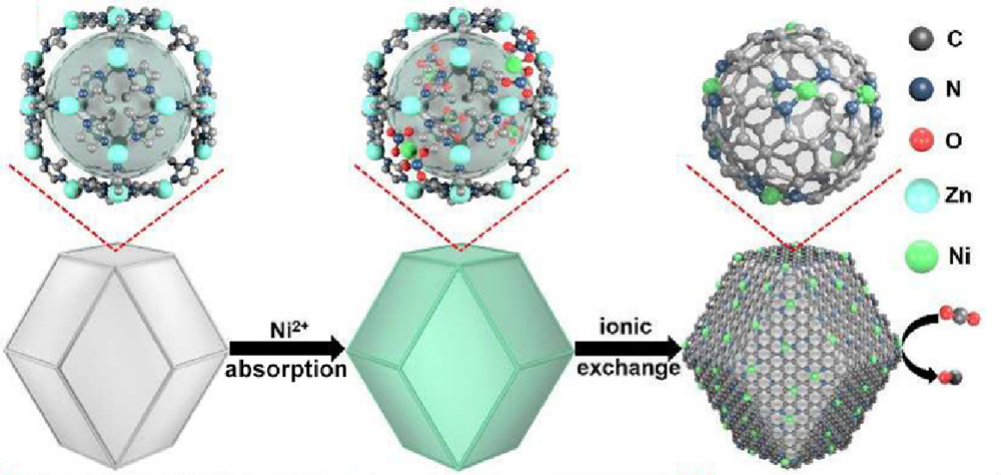
Scheme of ionic exchange method for the formation of Ni atomically dispersed carbon materials (Ni SACs/N-C) via pyrolysis of ZIF-8 MOFs. Reprinted with permission from ref (262). Copyright 2017 American Chemical Society.

In addition to the direct pyrolysis of polymer precursors, carbon frameworks were also employed as a precursor for Ni SACs synthesis. Li and co-workers reported a Ni SACs synthesis through the pyrolysis of a ZIF-8 based MOF precursor (Figure 22).^262^ The ZIF-8 was initially adapted by ionic exchange with adsorbed Ni salts like Ni(NO_3_)_2_ in an organic solvent such as *n*-hexane. After the pyrolysis of Ni-exchanged ZIF under Ar atmosphere, an atomic Ni dispersed N-doping carbon (named Ni SACs/N-C) was formed. The obtained catalyst exhibited a CO FE of over 71.9% and a current density of 10.48 mA cm^–2^ at an overpotential of 0.89 V. Bao and co-workers reported Ni SACs sites on a N-doped porous carbon (Ni-N-C) by the pyrolysis of ZIF-8.^267^ The obtained material exhibited 5.44 wt % Ni loading on the support, with the assumption that the unsaturated Ni-N site was more active than the Ni-N_4_. Further, the DFT calculations confirmed that NiN_2_V_2_ (V = vacancy) was much more active and selective for CO formation. The as-synthesized catalyst exhibited a high FE of CO at ∼97% and reached a maximum current density of 71.5 ± 2.9 mA cm^–2^ at −1.03 V.

**Figure 23:**
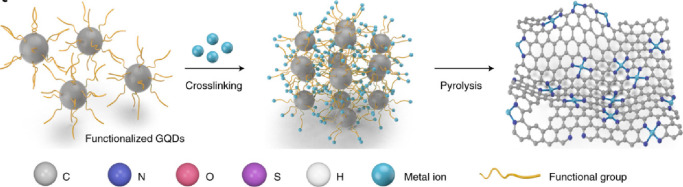
Scheme of the synthesis of single metal atom catalysts over carbon matrix through the cross-linking and self-assembly of graphene quantum dots. Reprinted with permission from ref (270). Copyright 2021 Springer Nature.

Zhang et al. reported a Ni single sites formation through the pyrolysis of pure Ni-ZIF-67,^263^ whereby NaCl was employed as the template to form N-doped carbon nanosheets during a thermal treatment. When the catalyst was tested in a flow electrolyzer, it exhibited near-unity selectivity to produce CO with a specific current density as high as 170 mA cm^–2^. Besides considering the influence of N coordination, the chemical properties of Ni sites also play a significant role to enhance the reaction selectivity. Recently, Zhang and co-workers reported a unique Ni species anchored in channel-rich nitrogen-doped carbon fibers (CFs) via a facile electrospinning method.^272^ The authors demonstrated that the atomic dispersed Ni in the fibers was regularly adjusted by subsequent pyrolysis from a single site configuration (Ni-N_3_-C) to a binuclear Ni bridging structure with the unique connected structure of binuclear nickel atoms with four nitrogen and two carbon atoms (Ni_2_-N_4_-C_2_). The designed Ni_2_-N_4_-C_2_ coordination structure exhibited a maximum CO FE of 96.6% at −0.8 V (vs RHE) and a TOF value of 4.6 × 10^3^ h^–1^ at −1.0 V tested in an H-cell. Very recently, Wang et al. introduced a novel method to largely synthesize Ni-SACs via NH_2_-functional carbon dots.^270^ The authors pointed out that the cross-linking method (Figure 23) through carbon dots enabled obtainment of as high as 15 wt % Ni loading. Further tested in a membrane electrode assembly (MEA) electrolyzer, the as-synthesized catalyst showed a CO FE of 80% with a current density of 50 mA cm^–2^. This study opened a route toward large-scale synthesis and application of high-loading Ni SACs catalyst for CO production using MEA based electrolysis.

**Figure 24:**
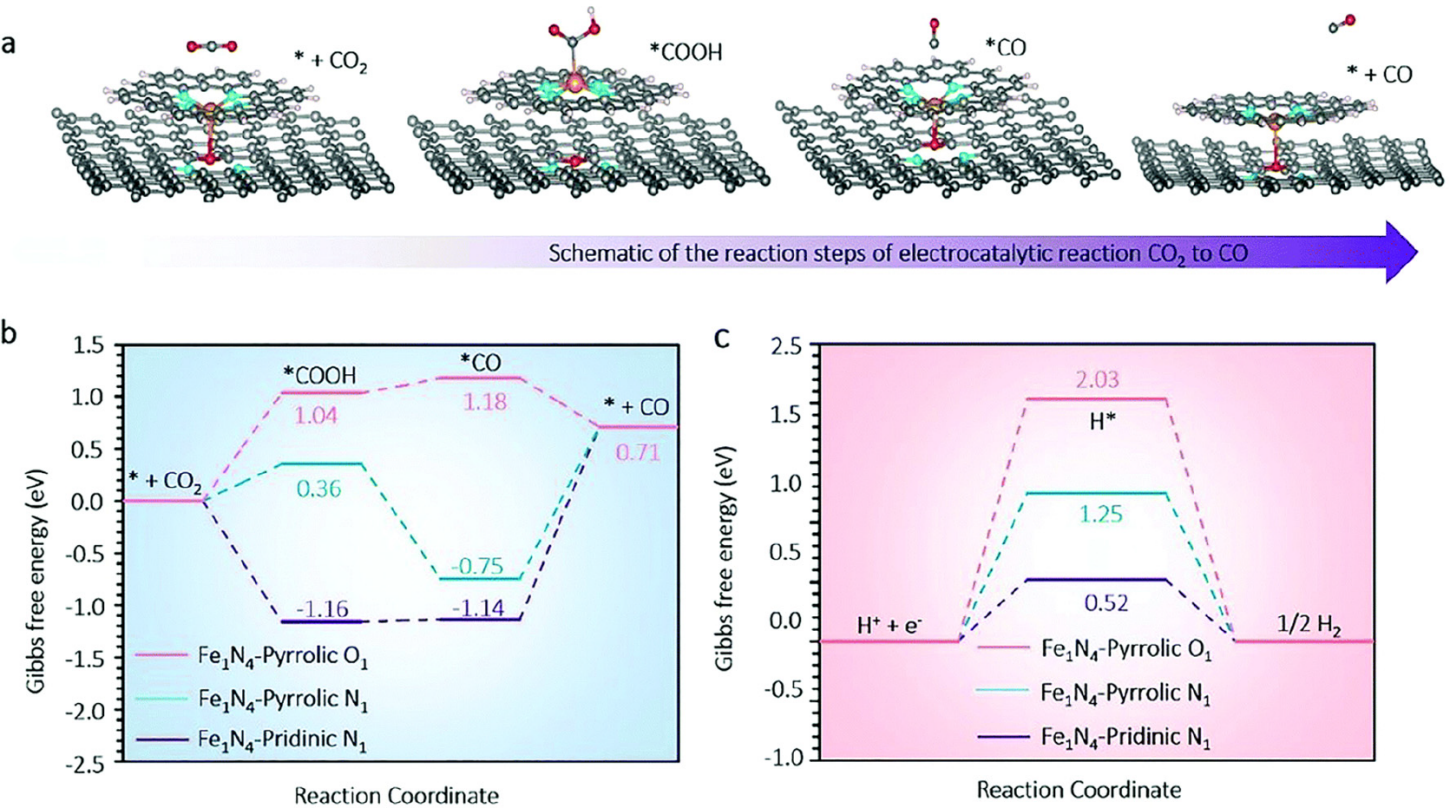
(a) Schematic of the reaction roadmap over the Fe_1_N_4_-O_1_ site for the electrochemical CO_2_-to-CO reaction. (b, c) Gibbs free energy diagrams for (b) ECO2RR and (c) HER pathways over different configurations of Fe_1_N_4_:Fe_1_-pyridinic N_4_-pyrrolic O_1_ (named as Fe_1_N_4_-pyrrolic O_1_), Fe_1_-pyridinic N_4_-pyrrolic N_1_ (named as Fe_1_N_4_-pyrrolic N1), and Fe1-pyridinic N_4_-pyridinic N_1_ (named as Fe_1_N_4_-pyridinic N_1_). Reprinted with permission from ref (273). Copyright 2021 Royal Society of Chemistry.

Analogous to Ni SACs, the most common supports deployed to anchor Fe SACs sites are nitrogen-rich carbon materials. Reportedly, FeN_5_,^254^ FeN_4_,^261,265^ and Fe-N_2_+_2_ in graphitic edges^229^ enabled nitrogen bonding local coordination, thus suppressing the aggregation of Fe atoms. For example, Zhang et al. reported a facile approach to synthesize atomically dispersed FeN_5_ active sites supported on a N-doped graphene using melamine as the N source.^255^ The as-synthesized FeN_5_ catalyst showed an excellent catalytic performance for the ECO2RR toward CO with a high FE of ∼97.0% at a very low overpotential of 0.35 V. The authors emphasized that the unique FeN_5_ active site plays a crucial role in achieving such a high CO selectivity. In contrast to FeN_4_, the axial pyrrolic N ligand depletes the electron density of the Fe_3d_ orbitals and reduces the Fe-CO π back-donation, increasing the rate of CO desorption, thus leading highly selective CO production. In addition, the nitrogen-rich environment effectively suppresses the aggregation of the Fe atoms on graphene during the thermal annealing process. Hu and co-workers reported a catalyst of atomically dispersed single Fe sites that derived from the pyrolysis of Fe-ZIF-8.^224^ The catalyst produced CO at a very low overpotential of around 80 mV. The partial current density of CO reached 94 mA cm^–2^ at an overpotential of 0.34 V. The authors argued that the low overpotential achievement was mainly due to the unique Fe-N_5_ moiety, where Fe^3+^ ions were coordinated to the pyrrolic nitrogen atoms, thus maintaining their 3+ oxidation state during the electrolysis, probably through electronic coupling to the conductive carbon support. The authors also suggested that the superior activity of the Fe^3+^ sites is due to the faster CO_2_ adsorption and weaker CO absorption than those of conventional Fe^2+^ sites. Similar to the preparation of Ni SACs, direct solid pyrolysis is also available for the formation of Fe SACs. Peng and co-workers reported a feasible approach to engineer the surface of Fe-N_4_ sites by direct pyrolysis from Fe precursor chelated carbon support. The authors proposed this new approach to form an additional axial O coordination on the Fe-N_4_ moiety (namely Fe_1_N_4_-O_1_), which enhanced the ECO2RR activity compared to the conventional Fe-N_4_ site.^273^Figure 24a displays the proposed CO_2_-to-CO conversion pathway, which involves two proton/electron transfer steps via two surface intermediates: *COOH and *CO. The DFT calculation (depicted in Figure 24b, c) confirmed that the axial O ligand in the FeN_4_-pyrrolic-O_1_ moieties induced the shift of the d-band center of the Fe 3d orbital to a lower energy level, which resulted in a rapid CO desorption and suppressed the competitive HER, overall steering the reaction toward a highly selective CO production. Similarly, Wang and co-workers reported a phosphorus–nitrogen dual coordinated Fe SACs moiety,^274^ where P coordination served as an electron donor that was able to increase the electron density of the Fe center. In detail, the authors claimed that the incorporated P atoms were in high coordination shells (*n* ≥ 3), and this third coordination shell of the Fe center increased the electron density. Consequently, the unique structure drastically decreased the reaction energy barrier for the *COOH formation and thus enhanced the reaction toward the CO formation. Besides adjusting the chemical properties of the center metal, coordination engineering in moieties could also change the activity of the CO formation.

**Figure 25:**
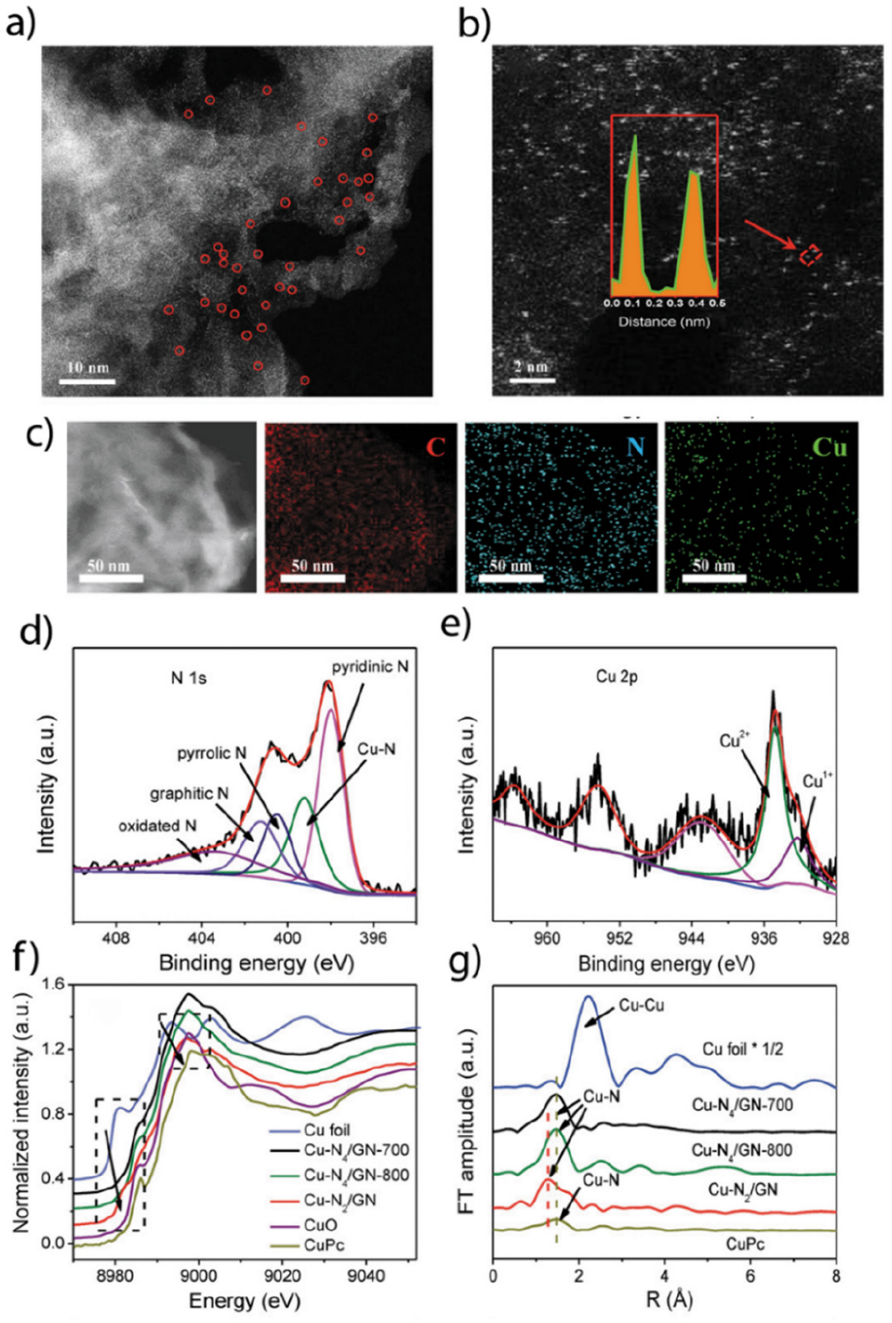
Characterization of single-atomic Cu site on graphene sheet named Cu-N_2_/GN: (a, b) HAADF-STEM images of Cu-N_2_/GN showing the isolated Cu single atoms are labeled by the red circle. The line profile of two Cu single atoms was inserted in panel b). (c) Large scale HAADF-STEM image and corresponding C/N/Cu elemental distribution mapping of Cu-N_2_/GN. The local chemistry analysis for the single Cu sites in Cu-N_2_/GN and control samples named Cu-N_4_/GN-700, -800: (d) High-resolution N 1s XPS spectrum and (e) high-resolution Cu 2p XPS spectrum of Cu-N_2_/GN, (f) XANES spectra of Cu K-edge for Cu foil, Cu-N_4_/GN-700, Cu-N_4_/GN-800, Cu-N_2_/GN, CuO, and Cu phthalocyanine (CuPc), (g) Fourier transform of Cu K-edge of EXAFS spectra for Cu foil, Cu-N_4_/GN-700, Cu-N_4_/GN-800, Cu-N_2_/GN, and CuPc. Reprinted with permission from ref (256). Copyright 2020 Wiley-VCH.

The bulk metallic copper catalyst has always been considered a good candidate for long-chain hydrocarbon formation in ECO2RR, but C^2+^ products are rarely observed on Cu SACs. Instead, like other metal SACs, with a single metal coordinated local structure, Cu-based SACs can also be deployed for CO production. Zheng et al. reported Cu SACs anchored on an ultrathin graphene nanosheet.^256^ The atomically dispersed Cu sites were confirmed by HAADF-STEM (Figure 25a–c). Notably, the authors demonstrated the existence of unsaturated Cu-N_2_ centers: an individual Cu site was associated with two nitrogen atoms (Figure 25d–g). Tested in an H-cell using 0.1 M KHCO_3_, the obtained catalyst exhibited a superior activity and selectivity for the ECO2RR with a maximum FE of 81% for CO production at a low potential of −0.50 V. Further, the authors also showed the potential application from their catalyst when adopted in a Zn-CO_2_ battery as the cathode. The unique activity was ascribed to the unsaturated environment and atomically dispersed nature of the Cu-N_2_ active sites.

In addition to conventional transition metals, some studies focused on more “exotic” metals compared to the typical researched SACs showing potential as ECO2RR catalysts for CO production. For example, Li and co-workers reported the preparation of a bismuth SAC via the pyrolysis of a bismuth-based MOF and dicyandiamide where exclusive Bi-N_4_ sites were formed.^258^ The catalyst showed a high selectivity (∼90% FE) toward CO formation, compared to a conventional Bi particle catalyst, which mainly formed HCOOH product. Recently, Zhang et al. reported dual element coordinated Mn SACs,^278^ where additional chlorine coordinated to the Mn-N_4_ centers (namely MnN_4_Cl). These MnN_4_Cl sites are N, Cl dual-coordinated moieties which induce further electron transfer between Mn and chlorine, thus resulting in the facilitation of CO_2_ and *COOH adsorption and the final CO desorption, thus greatly improving the activity. Additionally, Wang et al. developed cadmium single sites supported on N-doped carbon (Cd-N-C) and applied it for CO production from the ECO2RR.^279^ Similarly to other conventional M-N_4_ structures, the Cd-N-C also had the Cd-N_4_ coordination. Further, the authors employed the *operando* infrared spectroscopy method to reveal the presence of *COO^–^, *COOH, and *CO during the catalytic process, which favored the ECO2RR following a proton–electron separated transfer roadmap. In addition, the DFT simulations indicated that the Cd-N_4_ sites can lower the Gibbs free energy for the hydrogenation of *COOH, which was recognized as the rate-determining step. Finally, Liu and co-workers developed a s-block element SACs: magnesium atomically embedded in g-C_3_N_4_ through a facile heat treatment.^280^ The catalyst showed a high FE (90%) of CO with a current density higher than 100 mA cm^–2^ tested in a GDE. Further, the authors employed CO-TPD, CO adsorption electroresponse measurements, and *in situ* ATR-IR to demonstrate easier desorption of CO on Mg sites, which is mainly responsible for achieving high CO production.

#### 3.3.2 Electrochemical CO_2_ Reduction to Tunable Syngas Production

The conventional approach for producing syngas, a mix of carbon monoxide (CO) and hydrogen (H_2_), generally involves the reverse water gas shift reaction at elevated temperatures, leading to considerable energy and resource consumption.^292^ A promising, eco-friendly alternative is the production of syngas through the combination of CO2RR and the hydrogen evolution reaction (HER), utilizing electricity derived from renewable sources.^293−296^ This method effectively addresses the energy-intensive challenges of traditional processes. The effective Fischer–Tropsch process, which converts syngas to various hydrocarbons, often requires a hydrogen to carbon monoxide ratio between 0.3 and 4.^297^ The catalyst’s performance is key in this process, influenced by factors like its elemental composition, structure, surface characteristics, and particle size - currently even down to single atoms. Operational variables such as the applied electrochemical voltage, temperature, pressure, and pH of the electrolyte also play a significant role in managing the H_2_:CO ratio and improving the syngas production efficiency.^298,299^ Recently, considerable research effort has been invested in developing cost-effective, suitable electrocatalysts for syngas generation. The research attention concerning these electrocatalysts has been paid to the concept of fostering strong interactions with *COOH intermediates and weaker bonds with *CO, along with fine-tuning the CO:H_2_ ratio by modifying the material’s properties. Recent progress indicates that altering the catalyst’s size, structure, and composition can substantially boost its syngas production capabilities.^300,301^

**Figure 26:**
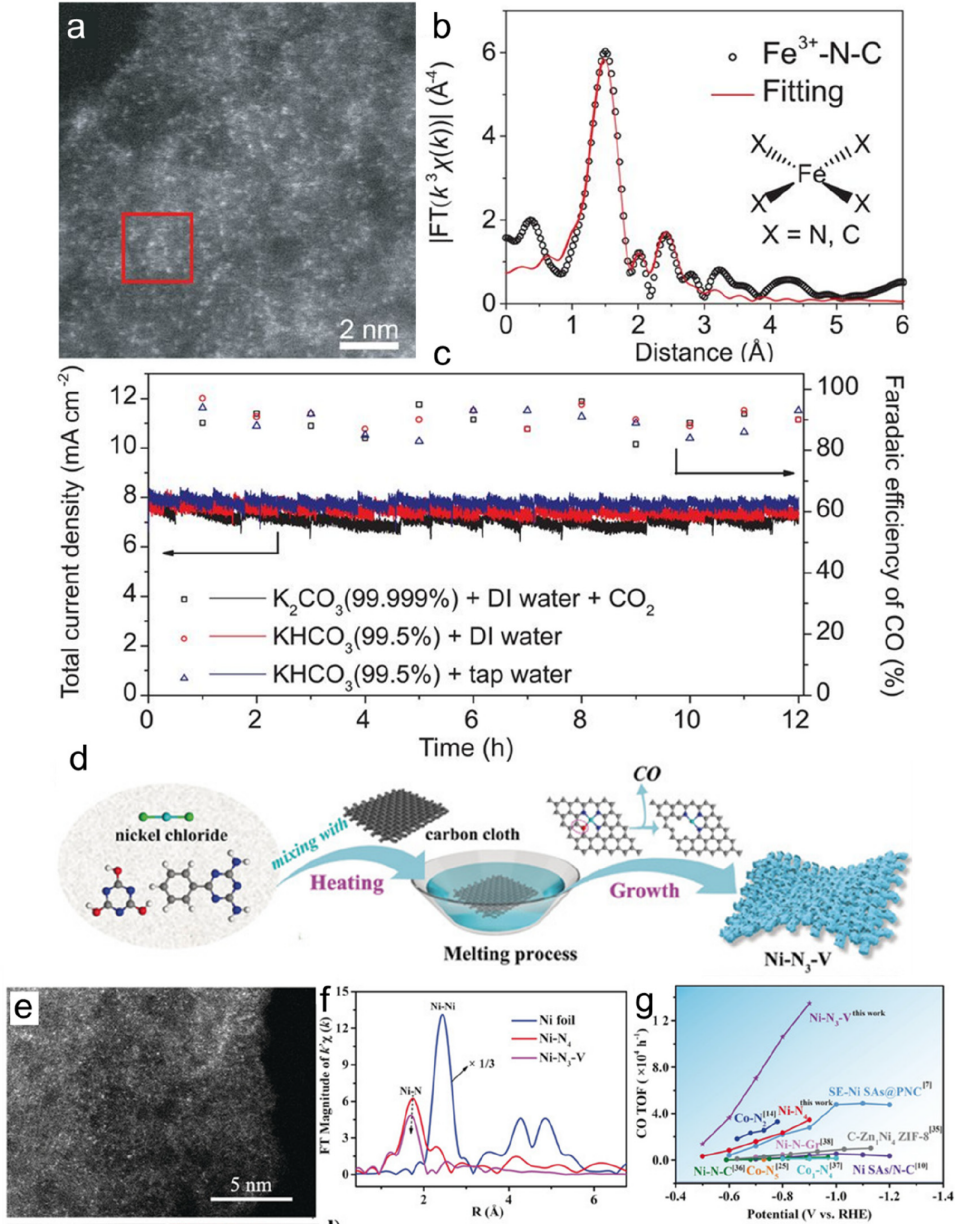
Aberration-corrected HAADF-STEM image (a) and R-space Fe K-edge EXAFS spectra (b) of Fe^3+^-N-C electrode for CO_2_ reduction. (c) Chronoamperometry curve and Faradaic efficiency of CO production (dots) by Fe^3+^-N-C in H-cell at −0.37 V versus RHE. The electrolytes were prepared from potassium carbonate (K_2_CO_3_) (99.999%) and deionized water (18.2 MΩ cm) (black), KHCO_3_ (99.5%) and deionized water (red), and KHCO_3_ (99.5%) and tap water (blue), respectively. Reprinted with permission from ref (228). Copyright 2019 AAAS. (d) Schematic illustration for the synthesis of Ni-N_3_-V electrode. (e) TEM image of Ni-N_3_-V electrode and (f) Ni K-edge k^3^-weighted FT-EXAFS spectra of Ni-N_3_-V, Ni-N_4_, and Ni foil (the EXAFS intensity of Ni foil is shown at one-third value). (g) TOFs of Ni-N_3_-V and Ni-N_4_ electrodes for CO production compared with those of other SACs. Reprinted with permission from ref (303). Copyright 2020 Wiley-VCH.

In Section 5 of this review, we will show, among others, that some of the transition metals, specifically Fe, Co, and Ni, when dispersed as single atoms on a support, exhibit a superior performance in the hydrogen evolution reaction (HER). In addition, as has been summarized in this section, these TMs also demonstrate the capability for effective CO2RR and H_2_O generation, leading to a syngas mixture with adjustable composition. To tackle this, nitrogen-doped carbon-supported single-atom catalysts are typically employed and studied for syngas production via the CO2RR. It was confirmed that a single TM atom could be effectively anchored into N-doped carbon supports due to the favorable TM–N bond formation, thus enabling distinct CO2RR behavior, compared to the typical bulk counterparts, in terms of the ratio and yield of CO/H_2_. In more detail, Gu and colleagues have documented the effectiveness of atomically dispersed Fe^3+^-N-C catalysts (see Figure 26a, b). They observed a notable shift in the potential from −0.2 V to −0.45 V versus RHE. Additionally, the Fe^3+^-N-C electrode demonstrated the ability to modify the CO:H_2_ ratio, ranging from approximately 2:1 to 18:1. In these catalysts, Fe^3+^ ions form a coordination with pyrrole N on the N-doped carbon support, maintaining their trivalent oxidation state.^228^ The catalyst also exhibited exceptional stability, as evidenced by a continuous 12-h electrochemical test reaction (Figure 26c). Following this prolonged electrolysis, the authors conducted an ICP-OES analysis, revealing a weight fraction of 2.6% for Fe in Fe^3+^-N-C. This result confirmed the absence of appreciable leaching of Fe ions from the catalyst. In another work, Fe^2+^ single atom sites were applied as well. To synthesize the Fe^2+^ SA catalysts, a voltage-gauged electrofiltration method was employed at ambient temperature. Fe^2+^ ions were produced from bulk Fe foil by electron release under a positive potential, followed by the movement toward the working electrode in the electric field. Consequently, single Fe atoms were uniformly anchored on the nitrogen-doped carbon electrode. This setup allowed rapid photoelectron transfer from photosensitizers to the atomically dispersed Fe sites, facilitated by the highly conductive N-C support. As a result, the Fe-SAs/N-C catalysts displayed remarkable photocatalytic activity in reducing CO_2_ in water to syngas under visible light, with a flexible CO/H_2_ ratio. The observed gas evolution rates for CO and H_2_ were 4500 and 4950 μmol g^–1^ h^–1^, respectively, with the adjustable CO/H_2_ ratio spanning from 0.3 to 8.8.^302^

Yang et al. demonstrated catalysts consisting of single nickel atoms using a pyrolysis method. These catalysts, with nickel atoms dispersed on nitrogen-doped graphene electrodes (labeled A-Ni-NG and A-Ni-NSG), showed a notable decrease in overpotential of −0.2 V at a current density of 10 mA cm^–2^ for CO production.^247^ Remarkably, the CO to hydrogen ratio for A-Ni-NSG reached 9:1, significantly surpassing the 1:1 ratio observed in Ni-NG at −0.95 V versus RHE. Furthermore, these SA nickel catalysts demonstrated a stable performance for up to 100 h, maintaining consistent CO production throughout this duration. Additionally, Rong and colleagues developed Ni single atoms with vacancy defects through a high-temperature calcination process. The schematic illustration of the synthesis procedure is depicted in Figure 26d. The single-atomic nature of Ni was demonstrated by HRTEM images (Figure 26e) and further confirmed by, e.g., EXAFS measurements (Figure 26f). The FE for the CO production using Ni single atoms with vacancy defects (Ni-N_3_-V) reached 92%, compared to the 80% for Ni single atoms without defects (Ni-N_4_) at −0.8 V versus RHE. Both experimental data and theoretical analyses showed that these defects not only reduce the energy barrier for creating *COOH intermediates but also decrease the energy barrier for the CO desorption.^303^ As a result, the referred electrocatalyst overperformed other state-of-the-art electrocatalysts based on SAs in their CO TOF activities (see Figure 26g).

Furthermore, the valence state of SACs plays a crucial role in determining the syngas production efficiency and the CO:H_2_ ratio. Research by He and colleagues combined SACs of Co and Ni in a bifunctional electrocatalyst, since they individually exhibited selectivity toward the production of H_2_ (Co) and CO (Ni), respectively.^304^ Such electrocatalyst demonstrated enhanced syngas production, achieving total currents exceeding 74 mA cm^–2^ and CO:H_2_ ratios ranging from 0.23 to 2.26, which is ideal for standard downstream thermochemical processes. The DFT calculations further verified that the Co-N_4_ and the Ni-N_4_ sites are key for the HER and the CO2RR, respectively. The coexistence of these sites allows the adjustment of the HER/CO2RR selectivity by altering the Co/Ni ratio. Ni and co-workers introduced another alternative of dual single-atom electrocatalysts, employing cobalt in two different forms.^305^ To put it simply, the created catalyst utilized the CoN_3_ sites and the nitrogen dopants, which jointly formed a hierarchically porous carbon (HPC-Co) framework serving as a base to fix cobalt phthalocyanine (CoPc). This CoPc interacts with both the N dopants and the CoN_3_, forming complex N-CoPc and CoN_3_-CoPc sites through π–π and Co–Co bonding. The HPC-Co/CoPc composite, with a ratio of 5:1, was capable of generating syngas at substantial industrial current densities, achieving over 200 and 880 mA cm^–2^ for H-type and flow cell set-ups, respectively. This high-efficiency syngas production was achieved due to the synergistic catalytic activity between the N-CoPc, which is selective for the CO2RR, and the CoN_3_-CoPc, which preferentially facilitates the HER.

#### 3.3.3 Electrochemical CO_2_ Reduction to Formic Acid and Formate

**Figure 27:**
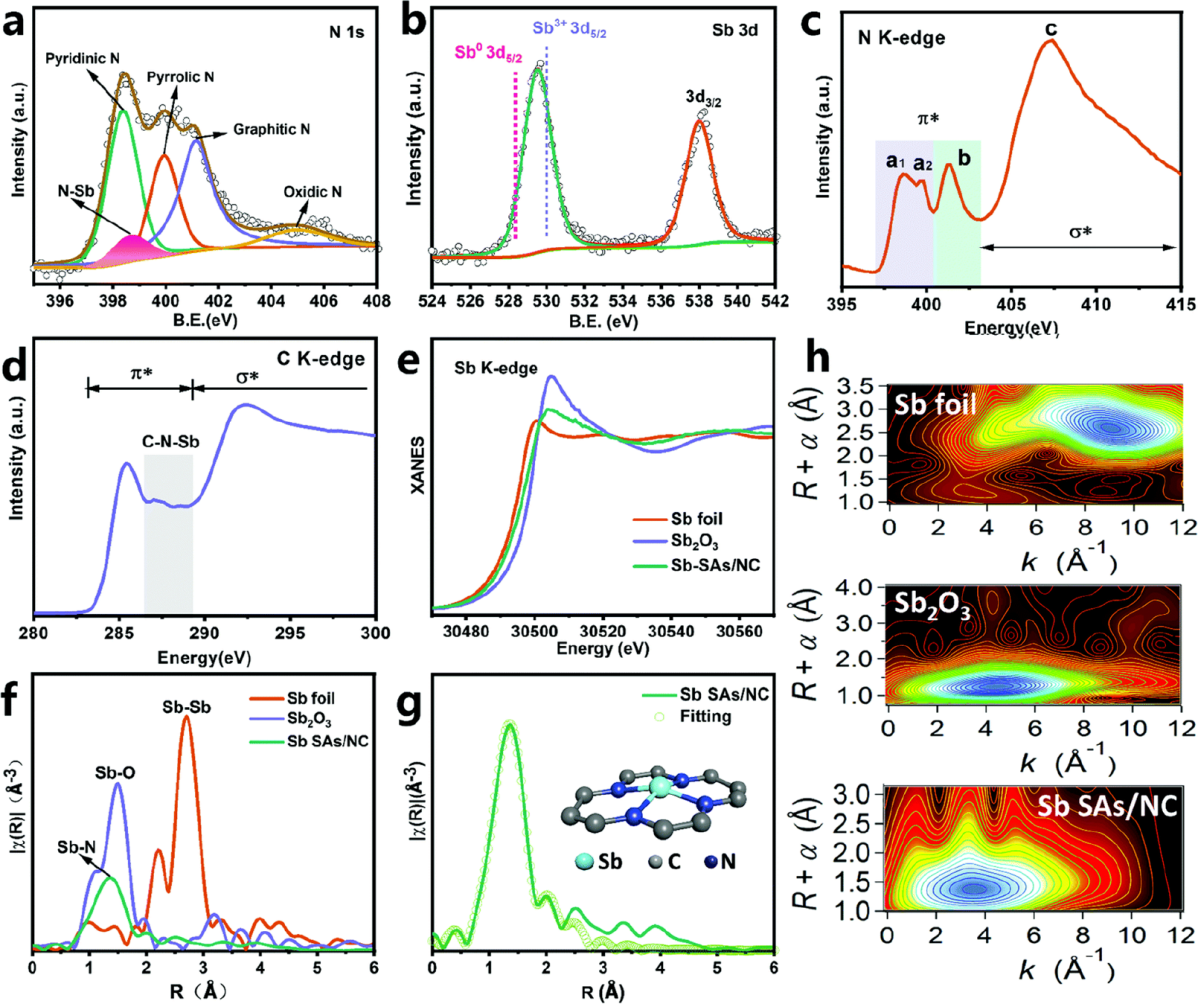
Chemical state and atomic coordination analysis of single-atomic Sb site on N-doped carbon nanosheet (named Sb SAs/NC): (a) N 1s XPS analysis and (b) Sb 3d XPS spectra of Sb SAs/NC. (c) N K-edge and (d) C K-edge soft XAS spectra of Sb SAs/NC. (e) Sb K-edge XANES spectra of Sb SAs/NC Sb_2_O_3_ and Sb foil. (f) Fourier transform of EXAFS spectra in R-space of Sb SAs/NC, Sb_2_O_3_, and Sb foil. (g) Fourier transform of EXAFS spectra and fitting curve of Sb SAs/NC with the schematic model as the inset image. (h) Wavelet transform of EXAFS plots of Sb foil, Sb_2_O_3_, and Sb SAs/NC. Reprinted with permission from ref (283). Copyright 2020 Royal Society of Chemistry.

The group of metals including bismuth (Bi), antimony (Sb), indium (In), and tin (Sn) species are promising candidates for ECO2RR to produce formic acid and formate, which is an important hydrogen storage material and a key chemical intermediate in many industrial reactions. In this context, Li and co-workers reported a Sb single atom site consisting of Sb-N_4_ moieties anchored on a N-doped carbon named Sb SAs/NC.^283^ The authors revealed that the pyridinic type of N accounted for the largest proportion of the N contents from N 1s XPS analysis (Figure 27a) and the formation of Sb–N–C bonds from XAS analysis (Figure 27c–e). The Sb atoms were monodispersed on the carbon matrix and associated with the N atoms in the Sb-N_4_ structure (Figure 27f–h). The obtained catalyst produced formate with a high FE of 94.0% at −0.8 V vs RHE. Further, combining *in situ* X-ray absorption fine structure analysis and DFT calculations, the authors proposed that the excellent ECO2RR activity originated from the positively charged Sb^δ+^-N_4_ (0 < δ < 3) active sites also determined by Sb 3d XPS analysis (Figure 27b). These findings provide significant guidelines for the rational design and accurate control for such metal SAC formation (e.g., Sb, In, Sn, Bi, etc.) toward the ECO2RR. With the same principle in mind, Zhu and co-workers developed an atomic layer of bismuthene possessing a 3D porous conductive network.^290^ When tested in a H-cell, the as-synthesized catalyst achieved a FE of over 90% for the formate production in a broad and mild potential window from −0.72 to −1.17 V. Moreover, an optimal FE value of ∼95% at −0.9 V vs RHE in a CO_2_-saturated 0.5 M KHCO_3_ electrolyte was obtained. Furthermore, the authors tested the catalysts in GDEs, and the catalytic performances reached 560 mA cm^–2^ at −0.97 V in a flow cell fed with 1 M KOH electrolytes. In addition, a long-term stability evaluation was carried out in a GDE assembly using 1 M KOH or 1 M KHCO_3_ electrolytes. In KHCO_3_, a stable formate production with FE above 90% was achieved at a high current density of 200 mA cm^–2^ during 110 h of continuous operation.

#### 3.3.4 Electrochemical CO_2_ Reduction to Methane and Methanol

The SACs M-N-C electrocatalysts commonly reduce CO_2_ via a two-electron reduction process to produce CO or formic acid. However, methane has appeared in many cases as a byproduct formed over some SACs when applied with a very negative potential. The key step to produce deep reduction products (e.g., CH_4_) requires the generation of the *CHO intermediate by the protonation of *CO, which necessitates overcoming a large energy barrier.^306^ Therefore, CH_4_ is preferentially produced at more negative potentials. Because copper nanostructures showed high potential to produce hydrocarbons from CO_2_, an early study of SACs targeting multiple carbon end-products mainly focused on novel Cu-based SAC developments.^281,307^ For instance, Wang and co-workers introduced a Lewis acid support to anchor Cu SACs, which showed significant enhancement for the CH_4_ formation.^281^ DFT calculations confirmed that the formation barrier of HCOO*, which was lower than that of COOH*, enhanced the proton–electron coupling transfer step. Accordingly, they confirmed that the Lewis acid favored the CH_4_ and CH_3_OH pathways rather than the CO and HCOOH because of the Lewis acid sites provided by the presence of metal oxides (e.g., Al_2_O_3_). Experimentally, Cu SACs could generate CH_4_ with a reasonable FE, but the main catalytic site and the corresponding mechanisms have been debated because the dynamic structure of Cu could evolve under reaction conditions. In addition, when the coordinated environment changes, such as using O to bond Cu, the selectivity to CH_4_ will be tailored consequently. Reported by Zheng and co-workers, CeO_2_ was chosen as support to anchor single Cu sites.^307^ The authors concluded that oxygen vacancy bound Cu sites and enabled a high FE for CH_4_ generation. Besides oxygen bonding, N-coordination could also act as an active site for the CH_4_ formation.^232^ The authors revealed that a low coordination Cu complex favored methanation. The catalyst operated for 110 h at a current density of 190 mA cm^–2^ in MEA electrolyzer with an average CH_4_ FE of 56%. In addition, theoretical chemistry could help to design and screen catalysts toward CH_4_ production. Reported by He and Jagvaral, DFT calculations predicted that Ag SACs supported on defective graphene could be promising for the CH_4_ formation with a starting reduction potential (−0.56 V vs RHE) smaller than those of prevailing Cu catalysts (around −0.83 V vs RHE).^308^ Then, Xin and co-workers reported a Zn-based SAC embedded in a N-doped carbon, showing a high CH_4_ FE of 85%, with a CH_4_ partial current density of −31.8 mA cm^–2^ at a potential of −1.8 V versus SCE.^282^ The authors reported that N atoms from N-doped carbon were stabilizing the Zn single sites and promote preferential CO_2_ adsorption due to a strong electronic coupling effect. Interestingly, the Zn single site in Zn-N_4_ moieties has been revealed as similar to another conventional M-N_4_ moiety.

Despite few reports using SACs for methanol synthesis in the ECO2RR, guidelines for catalyst design according to theoretical chemistry studies were provided and can act as a starting route to pave the way in this research effort. For example, Lu and Zhao predicted by first-principles DFT calculations that Cu-based SA alloys could be exceptional electrocatalysts for the ECO2RR.^309^ They predicted that a Co-decorated Cu SA alloy (Co@Cu SAC) could be promising in the production of methanol due to its low overpotential and high selectivity. The isolated Co atoms lead to a narrowed d-band and an upshift of the d-band center, which can stabilize the CO_2_ linear chemisorbed configuration through a C atom on a surface, thus significantly lowering the reaction barrier. Furthermore, the narrowed Co d-band increases the bonding to a key intermediate, enabling a selective and efficient production of CH_3_OH through the pathway of CO_2_ → *COOH → *CO → *COH → *CHOH → *CH_2_OH → CH_3_OH. This prediction was partially confirmed by a Co-SACs molecular catalyst prepared from Co phthalocyanine complexes,^236^ wherein the main product was methanol with an average FE of around 44%, and the only gas products were CO and H_2_, implying that Co-SACs are a key factor to accelerate the CO intermediate to further process proton coupling. Furthermore, Cui et al. investigated a series of nitrogenated 2D graphene-supported SACs, namely M@C2N (M = Ti, Mn, Fe, Co, Ni, Cu, Rh, and Ru) for the ECO2RR.^310^ They found that the relative carbophilicity and oxophilicity of the metal atom determined which intermediate will be formed. Interestingly, this influences the hydrogenation step forming *CH_3_O intermediate or *CH_2_OH intermediate, while the formation of the second intermediate *CH_2_OH turns out to be crucial for the methanol production. The authors predicted that Co, Ni, and Fe with certain M@C2N structures are promising ECO2RR catalysts in the methanol production because of lowered overpotentials (from 0.58 to 0.80 V). Like bulk Cu catalysts with intrinsic properties enabling multiple proton–electron transfer reactions, atomically embedded Cu in a matrix could also provide more than 2e^–^ transfer CO_2_ electroreduction processes. However, the low selectivity on the products has always been the main issue with these catalysts. For instance, Yang et al. reported isolated Cu decorated through-hole N-doping carbon nanofibers.^285^ The obtained catalyst achieved 44% FE of methanol in the liquid phase, with CO as the main byproduct. Moreover, the authors revealed that the pathway toward methanol formation only occurred over the Cu-N_4_ site. DFT calculations suggested that the Cu-N_4_ sites exhibited a relatively higher binding energy for the *CO intermediate and a slightly positive free energy (0.12 eV) for the *CO desorption. Thus, *CO could be easily reduced to products like methanol rather than being released from the catalyst surface as a CO product.

#### 3.3.5 Electrochemical CO_2_ Reduction to C^2+^ Products

In order to obtain multiple C–C bond products, coordination engineering has been employed to tune the single metal coordination chemistry environment, such as O-enrichment and oxygen vacancy bonding. Consequently, a single active site combined with the metal-oxide interaction has been introduced as an interesting route to reach multiple carbon products.^311^

Another strategy is to anchor an entire single metal organic complex, such as a molecular scaffolding strategy or surface covalent chemistry. The underlying mechanism entails a homogeneous molecular catalysis of CO_2_ reduction; however, the influence of the support remains unclear.

**Figure 28:**
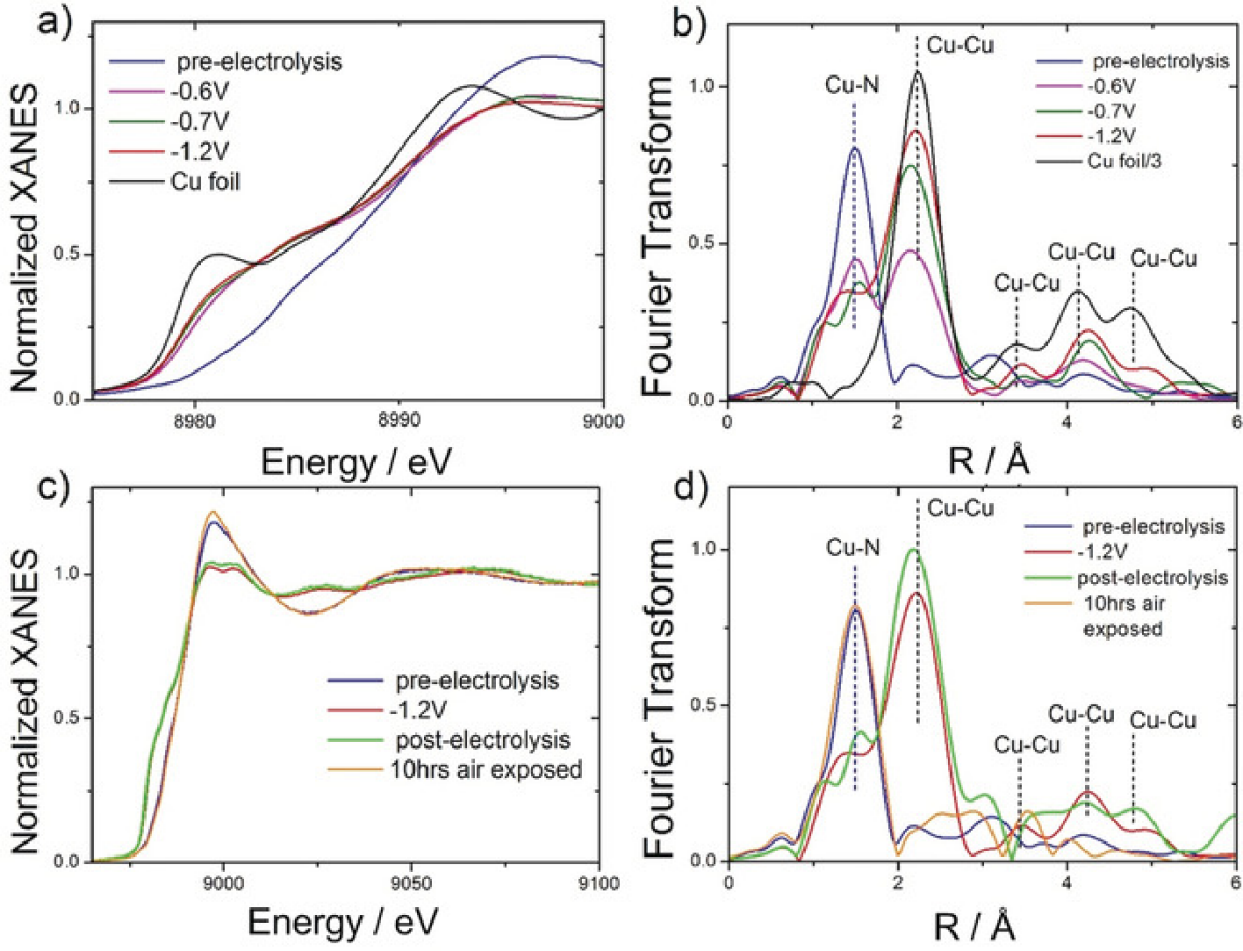
*Operando* XAS characterization of Cu_0.5_NC at the Cu-K edge: (a) K-edge XANES spectra of Cu_0.5_NC without applied potential (blue line); Cu_0.5_NC during electrolysis at −0.6 V vs RHE (pink line), at −0.7 V vs RHE (green line), and at −1.2 V vs RHE (red line); and metallic copper (black line). (b) Fourier transform of EXAFS spectra of Cu_0.5_NC without applied potential (blue line); Cu_0.5_NC during electrolysis at −0.6 V vs RHE (pink line), at −0.7 V vs RHE (green line), and at −1.2 V vs RHE (red line); and metallic copper (black line). (c) Comparison between the K-edge XANES spectrum of Cu_0.5_NC without applied potential (blue line), Cu_0.5_NC during electrolysis at −1.2 V vs RHE (red line) and after electrolysis under no potential applied (green line), and Cu_0.5_NC after electrolysis at −1.2 V vs RHE then sample exposed to air (orange line). (d) Fourier transform of EXAFS spectra of Cu_0.5_NC under no potential applied (blue line), Cu_0.5_NC during electrolysis at −1.2 V vs RHE (red line) and after electrolysis under no potential applied (green line), and Cu_0.5_NC after electrolysis at −1.2 V vs RHE then sample exposed to air for 10 h (orange line). Reprinted with permission from ref (286). Copyright 2019 Wiley-VCH.

Besides the contribution from the coordinated frame, an *in situ* generated cluster has also been treated as an active catalyst for beyond C1 products formation. A copper-based single-atom catalyst has been reported to generate ethanol from CO_2_ in a flow cell^286^ wherein the Cu-N_4_ coordination provided a unique environment that governed the catalytically active species evolution under reaction conditions. Notably, it was discussed that the active site derived from the reconstruction of the Cu-N_4_ site during the reaction, and the restructuring process was reversible. However, after long-term electrolysis at a negative potential (−1.2 V vs RHE), the Cu-N_4_ sites partially converted into Cu nanoparticles. *Operando* spectra of the catalyst (namely Cu_0.5_NC), shown in Figure 28a–d, confirmed the *in situ* formation of Cu nanoparticles, thus implying large ensembles of copper atoms. Specifically, the Fourier transform of the EXAFS spectrum clearly indicated that Cu was essentially in the form of nanoparticles after prolonged electrolysis at −1.2 V vs RHE (Figure 28d). The authors stated that the Cu nanoparticles promoted the C–C coupling reaction. The structure was derived from Zn-ZIF-8, which could exclude the contribution from the residual Zn atom. The work provided the methodology of using single atom materials as precursors to *in situ* reconstructions of some active sites for the CO_2_ reduction. In some respects, active ECO2RR to multicarbon products is most likely to occur over copper clusters rather than over SA anchored structures. As a conventional prediction and previously discussed, the M-N_4_ coordination structure favors the CO formation.

Recently, Zhao et al. reported the atomic distribution of Cu on a N-doped porous carbon where the Cu species existed as Cu^2+^ coordinated by four pyrrolic N atoms.^287^ The as-synthesized catalyst can produce both liquid and gas products, including formic acid, acetic acid, methanol, ethanol, acetone, H_2_, and CO. Among them, acetone was the major product, with a maximum FE value of 36.7%. The DFT calculations showed that the *CO species dimerized on the Cu-pyrrolic-N_4_ site but not on the Cu-pyridinic N_4_ site. The authors clarified that the higher selectivity originated from the unique Cu-pyrrolic-N_4_ active sites, which could stabilize the reaction intermediates involved in the acetone production as well as facilitating the C–C coupling reactions due to the Cu–N synergetic effect. Since the productivity is still at a low value, it creates the opportunity to adjust the local structure with well-coordinated Cu SACs, thus accelerating the ECO2RR toward long-chain hydrocarbons.

In summary, in this section it was demonstrated that metal atoms coordinated with nitrogen in a carbon matrix, particularly iron, are highly effective for electrochemical CO_2_ reduction to CO, offering over 80% Faradaic efficiency (FE) and high current density. Further extensive exploration of various metal–nitrogen structures revealed a tendency of many SACs to favor CO production over hydrogen evolution, which is attributed to the isolated transition metals active sites (Fe, Co, Ni, Cu, etc.) that limit competing water adsorption. Significant research effort has focused on cobalt, nickel, and iron SACs. Cobalt SACs, inspired by molecular catalysts like cobalt phthalocyanine, have shown high selectivity and efficiency in CO_2_-to-CO conversion, with notable advances in creating stable Co-N_4_ structures and exploring Co-N-C coordination effects. Similarly, nickel SACs in the form of Ni-N_*x*_ and iron SACs, particularly FeN_5_ and FeN_4_, have been synthesized with high selectivity for the CO production, benefiting from nitrogen coordination that provides suitable electronic properties and suppresses metal aggregation. This research also extends beyond traditional transition metals, exploring metals like bismuth, manganese, cadmium, and magnesium in SAC configurations. These studies revealed unique coordination environments and electron transfer dynamics, leading to high efficiency in the CO production from ECO2RR. Metal species such as bismuth, antimony, indium, and tin as catalysts have also been extensively studied for the electrochemical reduction of CO_2_ to formic acid and formate, which is essential for hydrogen storage and further industrial applications. Strategies like coordination engineering and molecular scaffolding have been employed to create single active sites combined with metal-oxide interactions, resulting in diverse carbon products. While challenges in selectivity persist, recent Cu-based catalyst research, specifically on Cu-pyrrolic-N4 sites, has enabled the production of various liquid and gas products. Furthermore, the opportunity to enhance local structures through the use of precisely coordinated copper single-atom catalysts presents a promising path for improving the electrochemical reduction of CO_2_, specifically in the production of long-chain hydrocarbons.

## 4 EARTH-ABUNDANT SACs IN OXYGEN REDUCTION REACTION (ORR)

The main applications of ORR are represented by fuel cell technologies which are suitable for renewable energy. Extensive research efforts focusing on ORR pursue the encouraging prospects to generate sustainably valuable chemicals and fuels. Membrane-exchange fuel cells and metal-air batteries represent the main devices adopting ORR as their cathodic reaction. However, ORR represents the major challenge in fuel cell technologies due to its sluggish kinetics. This drawback is directly related to the multistep process involving O_2_ reduction to water. The ORR process is highly dependent on the pH. As such, in alkaline conditions H_2_O will provide protons (eq 1–4):
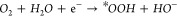
1
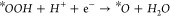
2
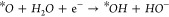
3
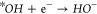
4whereas in acidic media it will be H_3_O^+^ (simplified as H^+^) (eq 5–8).
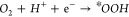
5
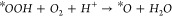
6
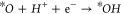
7
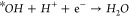
8Interestingly, under certain conditions, these four-electron (4e^–^) processes toward the H_2_O production compete with the two-electron (2e^–^) reduction toward hydrogen peroxide (H_2_O_2_, eq 9 for alkaline and eq 10 for acidic conditions).
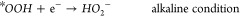
9
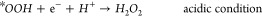
10

Thermodynamically speaking, 4e^–^ ORR E^Ø^_eq_ = 1.23 V vs RHE while 2e^–^ ORR E^Ø^_eq_ = 0.7 V vs RHE.

As already mentioned, the major challenge in fuel cell technologies is the sluggish kinetics of the ORR at the cathode. Among catalysts, precious metal-based materials (e.g., Pt, Ir, Pd) have been extensively studied for the ORR. The platinum group metals (PGMs) represent the standard electrocatalysts with promising activity and durability, especially in acidic electrolytes. Due to their scarcity and high costs, reducing the content of PGMs, particularly Pt, while still maintaining an efficient fuel cell performance is highly desirable so that the cost requirements for scale-up commercialization of fuel cells are met. On another hand, PGM-free catalysts have been extensively researched in parallel due their promising outcomes and cost effectiveness despite numerous challenges. Selected metal complexes that serve for molecular catalysis have been already reported for ORR applications. Jasinski and co-workers first discovered the ORR activity of Co phthalocyanine complexes in 1964.^312^ After that, the concept of forming a M-N_*x*_ structure employing nitrogen groups to bind transition metal species and anchor them to a heterogeneous carbon surface became common practice.^313^ Due to the similarity of the M-N_*x*_ coordination with a molecular catalyst, the M-N-carbon materials, which were usually obtained via a pyrolysis process of composite precursors containing metal ions, nitrogen, and carbon, have been extensively investigated for the ORR. Recently, isolated single atomic sites coordinated with nitrogen in a carbon matrix, represented as M-N-C (M = Mn, Fe, Co, Ni, Cu, etc.), were introduced as promising candidates for the ORR enhancement.^314^ Among them, the essential structure usually exhibit active metal centers (e.g., Mn, Fe, Co, etc.) that are coordinated to heteroatoms (e.g., N) on a conductive support (e.g., carbon framework). In other words, these N-doped carbon materials with metal ion atomically dispersed are an emerging family of interesting electrocatalyst for ORR. Notably, PGM-free SACs have already demonstrated promising activity for the 4e^–^ reduction of O_2_ with modest stability. In this section, we will focus the discussion on the newly discovered transition metal M-N-C SACs for ORR. The major developments in the design and fabrication of such SACs in electrochemical devices focused on ORR are also presented in this segment with the discussions divided between the different SACs followed by the focused reaction pathway.

### 4.1 Active Sites of SACs toward ORR

**Figure 29:**
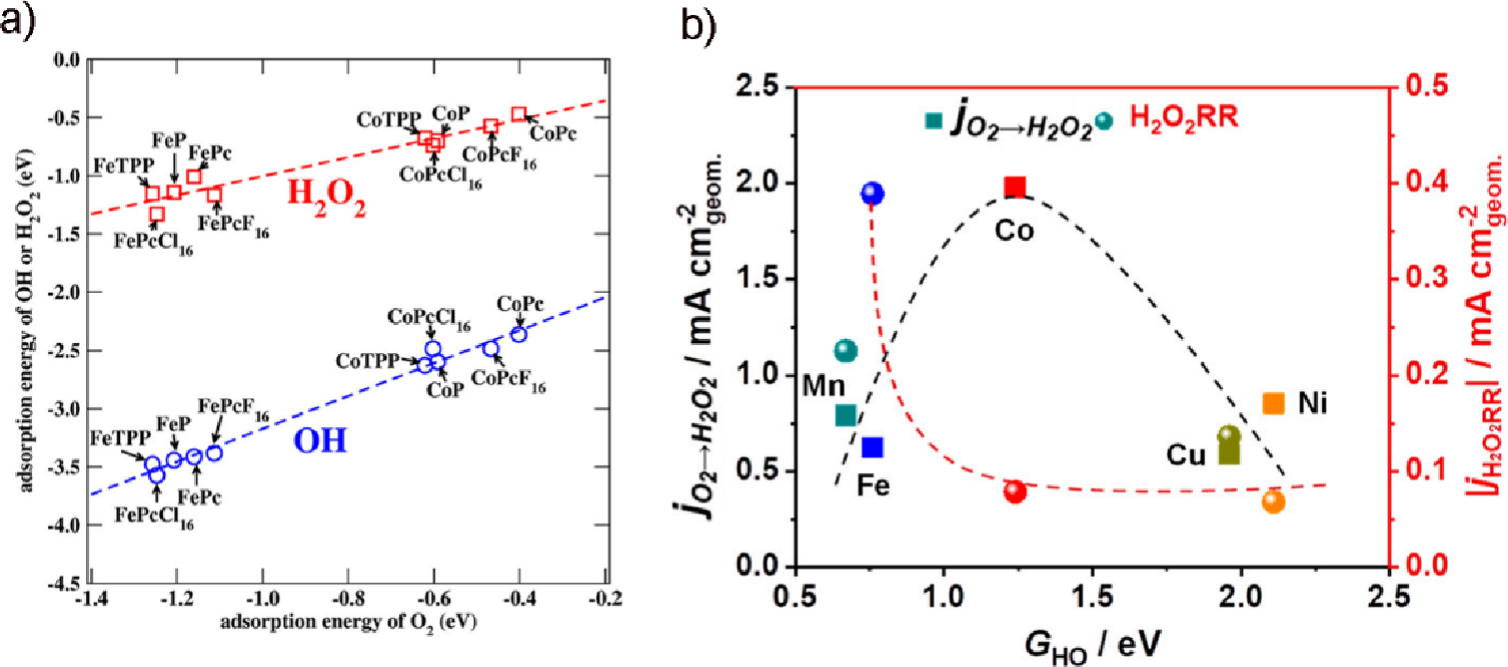
(a) Correlation plot showing the variation of the calculated adsorption energy of different intermediates over the Fe and Co macrocyclic complexes: the OH (blue circles) and H_2_O_2_ (red squares) as a function of the calculated adsorption energy of the O_2_. The linear trend was draw in the dashed line. Reprinted with permission from ref (316). Copyright 2012 American Chemical Society. (b) The correlation of *HO binding free energy with specific current density: H_2_O_2_ formation current density black line and H_2_O_2_ deep reduction current density red line for M (M = Mn, Fe, Co, Ni, and Cu)-N-C materials. Reprinted with permission from ref (317). Copyright 2019 American Chemical Society.

The intrinsic catalytic activity of SACs usually arises from the orbital interactions of adsorbates with the active sites. Therefore, considerable progress has been achieved in altering the electronic structures by preparing diverse types of metal SACs for M-N_4_ matrices. Moreover, some studies show that extra heteroatoms (e.g., C, O, S, P, etc.) can tailor the coordination environment of the active catalytic sites, thus improving the catalytic performance. The electrocatalytic activity of M-N-C materials toward 4e^–^ ORR to H_2_O represents a mainstream line of research into replacing PGM-based catalysts at the cathode of fuel cells. However, fundamental and practical aspects of their electrocatalytic activity toward 2e^–^ ORR to H_2_O_2_, a future green “dream” process for on-site H_2_O_2_ production, remain barely understood. Zagal and Koper proposed several descriptors (such as donor–acceptor hardness, M-O_2_ binding energies, and the M^+n^/M^+(n–1)^ formal potentials) to predict the ORR activity in the M-N_4_ porphyrins complex structure.^315^ Particularly, the authors draw the correlation between the M^III^/M^II^ redox potential of the M-N_4_ moiety and the M-O_2_ binding energies. Notably, the CoN_4_ moiety, which shows a weak binding with O_2_, yields mainly H_2_O_2_ as a product, with an ORR onset potential being independent of the pH value on the standard hydrogen electrode (SHE) scale. In contrast, catalysts with stronger O_2_-binding yield H_2_O as a product, with the expected pH-dependence on the SHE scale. These descriptors were also applicable for heat-treated M-N_4_ heterogeneous catalysts. Coincidentally, Wang and co-workers revealed that the type of metal center is the most determining factor on the adsorption energies of O_2_, OH, and H_2_O_2_ in phthalocyanine macrocyclic complexes and the ligand modifications can modulate the binding strength among the adsorbed O_2_, OH, and H_2_O_2_ as well.^316^ As shown in Figure 29a, the Fe-N_4_ catalysts bind H_2_O_2_ and OH more strongly than the Co-N_4_ catalysts, which explains why Fe-N_4_ sites are more favorable than the Co-N_4_ sites to catalyze the splitting of O–O bonds and thus trend to 4e^–^ ORR. Similarly, Peter Strasser and co-workers summarized the activity-selectivity trends over a series of M-N-C materials by a combination of computational and experimental efforts.^317^ The authors discovered that a Co-N-C structured material displayed the best performance for the H_2_O_2_ formation. As evidence, the surface binding energy of intermediates *OH, which was chosen as a descriptor, is at the top of the “volcano plot” showing Co-N-C as a great candidate for H_2_O_2_ production (Figure 29b). Chen et al. reported a feasible SiO_2_-templated approach for a Fe single-atom dispersed N-doped carbon hollow sphere synthesis. Such catalyst showed high activity for ORR in alkaline conditions.^318^ The key to achieving a dispersed atomic Fe is to use histidine (a biomaterial) as the N and C precursors. Because numerous atomically dispersed Fe-N_4_ moieties were formed, the as-synthesized Fe SACs exhibited an excellent ORR performance toward the 4e^–^ pathway in alkaline medium. More recently, Tan et al. reported Fe SACs with a square-pyramidal Fe-N_4_ moiety with defect-modulated O-coordination.^319^ The authors pointed out that regulating the tuning power of the O-coordination can significantly influence the final Fe SACs local structure, thus enhancing its ORR activity.

**Figure 30:**
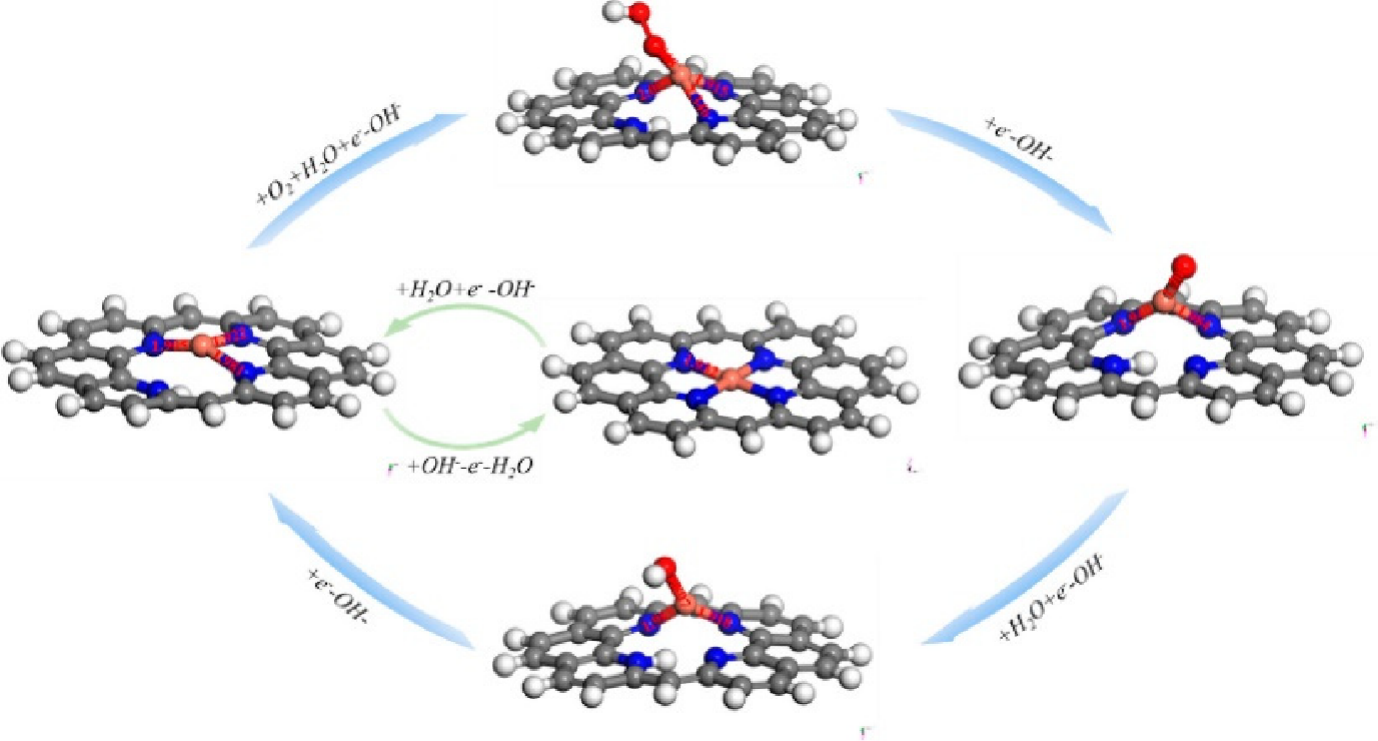
Schematic description of proposed catalytic mechanism for the alkaline ORR pathway on the single-atomic Cu sites on carbon named as Cu-N-C SACs. Reprinted with permission from ref (325). Copyright 2021 American Chemical Society.

Typically, SACs are prepared by the pyrolysis of templates with predefined metal–nitrogen coordination. Interestingly, MOFs have been identified as ideal precursors to obtain porous N-doped carbon materials. Therefore, MOFs with defined N coordination could serve as an attractive precursor for the N-coordinated SAC synthesis. Recently, MOFs have already been intensively used as precursor for SACs synthesis because of their structural diversity, high specific surface area, and porosity.^132^ Moreover, carbonization of MOFs can create porous N-doped carbon structures offering the structure to embed SACs and facilitating the electrolyte transport. Among them, ZIFs have emerged as a new platform for M-N-C SAC synthesis because ZIFs provide carbon and nitrogen atoms from the ligands and the flexibility to dope active transition metals into these frameworks. Moreover, the original M–N bond connected with hydrocarbon networks could directly generate M-N_*x*_ sites after pyrolysis, thus ensuring an active site control for the ORR. Xu and co-workers disclosed a strategy for the incorporation of Fe and N atomic doping in the hierarchical graphitic porous carbon architectures (Fe/N-SACs) from an amino functional MOF composite (MIL-101-NH_2_). The activity enhancement is from the synergetic effects of the doped N atoms and the Fe-N_*x*_ species in the catalyst.^320^ High ORR catalytic activity with an onset potential of 0.85 V and a half-wave potential of 0.63 V vs RHE was observed for the as-synthesized Fe/N-SACs electrode. The reaction follows a 4e^–^ ORR pathway from 0.4 to 0.2 V in acidic media. Li and co-workers introduced a general strategy enabling a practical-scale and controlled synthesis of Co SACs supported over N-doping carbon through a bimetallic MOF.^321^ Generally, the bimetallic MOF served as a host, which contained homogeneously dispersed Zn^2+^, Co^2+^, and cheap ligands (2-methylimidazole). After pyrolysis in inert gas, the MOF started to form N-doping carbon and the metal species (Zn and Co) were further reduced *in situ* to metallic Co and Zn in the presence of carbon. Importantly, the Zn atoms evaporated away at 800 °C because of their low boiling point. Finally, a stable SA Co/N-doped porous carbon electrocatalyst with a high metal loading (over 4 wt %) was successfully obtained. As a result, Zn^2+^ was added to substitute a portion of Co^2+^ sites and to expand the spatial distance of adjacent Co atoms. The same group reported an Fe atomically dispersed on carbon ORR catalyst via a cage-encapsulated-precursor pyrolysis strategy,^322^ where ZIF-8 was employed as molecular-cage to separate and encapsulate the metal precursor iron(III) acetylacetonate (Fe(acac)_3_). After pyrolysis in Ar at 900 °C, ZIF-8 was transformed into N-doped porous carbon. Meanwhile, Fe(acac)_3_ within the cage was reduced to form isolated single Fe atoms anchored on nitrogen species. Besides Co- and Fe-doping carbon structures, other MOF-derived metal SACs, such as Cu, Ni, and Mn, have rarely been published for ORR, probably due to their poor catalytic activity and stability. In addition, using cheap metal complexes to develop the transition metal SACs (e.g., Fe, Co, Cu, Ni, and Mn), decorated N-doped carbon structures could also achieve high ORR activity, owing to the unique electronic structures and the synergetic effects between the metal and the N coordination. Particularly, Fe-N-C species have emerged as a class of promising electrocatalysts for ORR activity due to Fe earth abundance, tunable surface chemistry, modified electronic structure, and optimal oxygen absorption.^318,112^ Besides the metal center, the local coordination environment also influences the ORR activity (e.g., some covalent local structures) with an N-doped carbon matrix and nitrogen, phosphorus, and sulfur codoped hollow carbon.^112^ All these reports proved that the heteroatom doped substrate firmly stabilized the highly energetic SAs through the M-N interaction in order to mitigate the aggregation of metal atoms, alongside effectively facilitating the transport of the ORR relevant species (i.e., *O, *OH, *OOH, and *O_2_) during the electrocatalytic process. It has been reported that the electrocatalytic activity of the M-N-C catalysts followed the order Fe > Co > Mn > Cu > Ni in both acid and alkaline electrolytes.^323,324^ Apparently, the Cu–N–C materials were also recognized as alternative ORR catalysts. Wang and co-workers demonstrated an active site in Cu-N_*x*_ SACs with a dynamic evolution during the ORR.^325^ The authors revealed that the Cu-N_3_ site was the most favorable active site for the ORR steps in comparison with the usual Cu-N_4_ moiety. As shown in Figure 30, under the reduction potential, the dynamic evolution of Cu^2+^-N_4_ to Cu^+^-N_3_ was easily initiated by the ionization of water into free OH^–^ and H^+^ via the subsequent hydrogenation of pyridinic N. Next, the produced Cu^+^-N_3_ single-atom active sites simplified the activation of O_2_ by an electron transfer to the antibonding orbital of the oxygen molecule, leading to the dissociation of oxygen as well as the successive reduction into OH^–^. The authors point out that the oxygen activation step was associated with the transformation of Cu^+^-N_3_ to Cu^+^-N_2_.

### 4.2 SACs in 2e^–^ ORR Pathway

**Figure 31:**
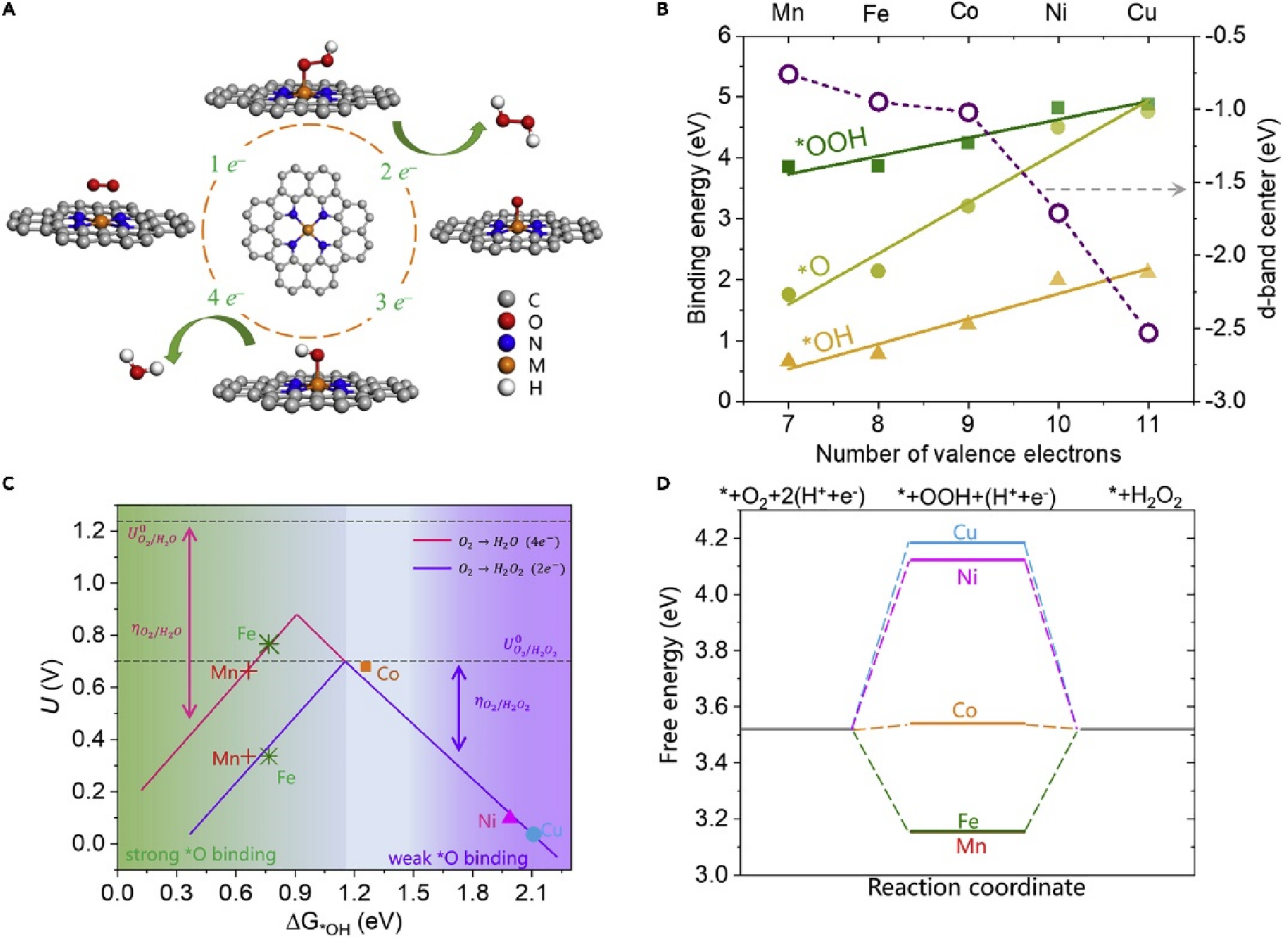
Correlation of transition-metal single-atom-catalyst (M-SAC) in N-doping graphene and the production of H_2_O_2_, where the metal is Mn, Fe, Co, Ni, and Cu: (a) Schematic of ORR along the 2 e^–^ or 4 e^–^ pathway on transition metal SACs moiety. (b) Binding energy of *OOH, *O, and *OH on M-SAC and d-bond center (open circle) of metal atom in M-SAC moiety. (c) Activity-volcano curves of ORR via the 2 e^–^ or 4 e^–^ pathway. The limiting potential is plotted as a function of Δ*G* of *OH. The gradual change in color indicates the catalyst window for producing H_2_O_2_. (d) Free energy diagrams of 2 e^–^ ORR on the SACs at U = 0.7 V vs RHE. Reprinted with permission from ref (328). Copyright 2020 Elsevier.

**Figure 32:**
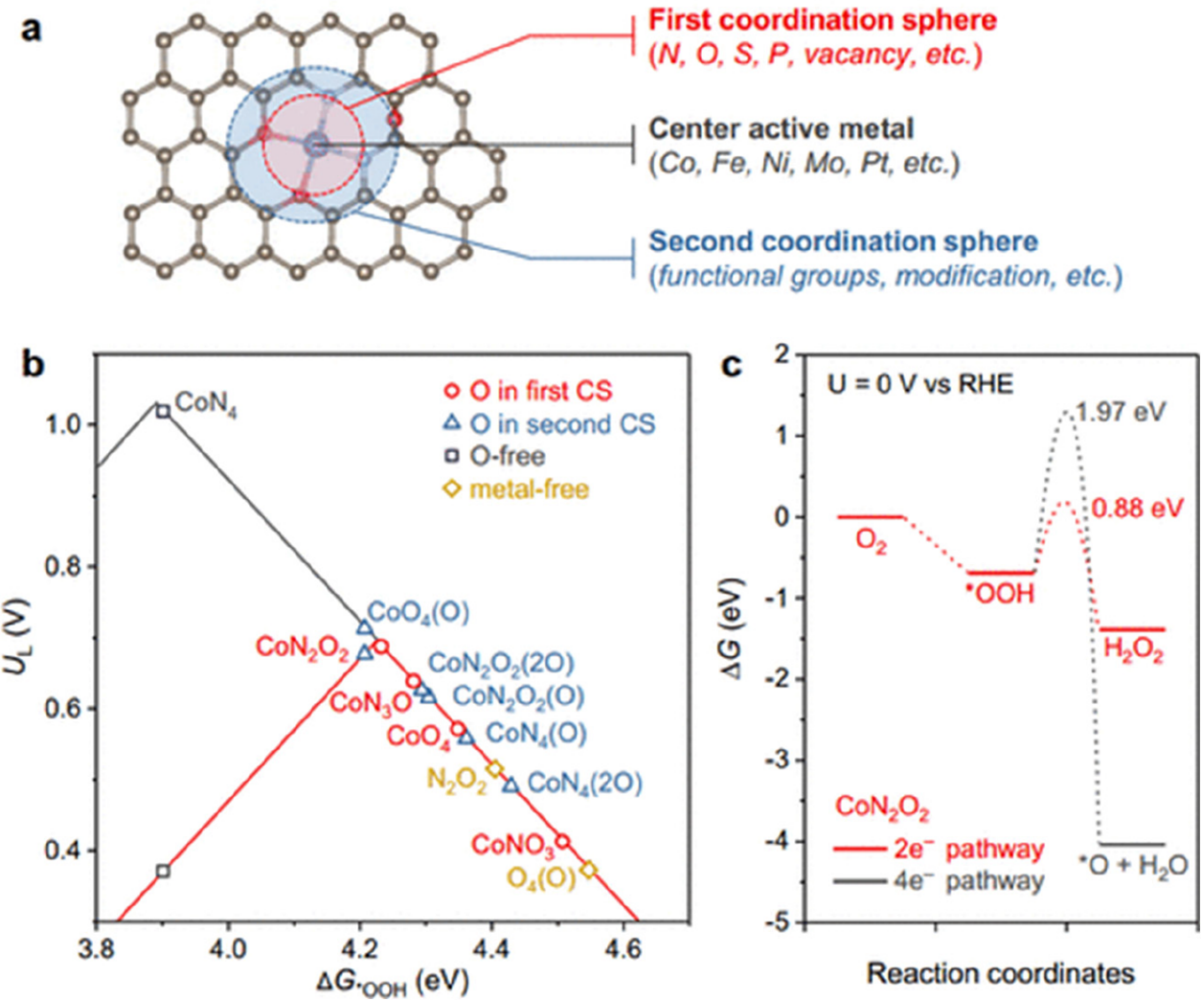
Theoretical predictions N-corrdinated Co-SACs for 2e^–^ ORR in molecular-level: (a) Schematic of Co SACs, highlighting first and second coordination spheres and center active metal. (b) Computed activity volcano plots of ORR via the 2e^–^ (red color) or 4e^–^ (black) pathway for SACs with varied configurations. (c) Free energy diagram for the 2e^–^ (red) or 4e^–^ (black) ORR pathway on the CoN_2_O_2_ moiety, where account for *OOH and *O intermediates. Reprinted with permission from ref (329). Copyright 2021 American Chemical Society.

The main goal of using transition metal SACs is to replace the noble metals in order to achieve higher atom utilization efficiency and cost effectiveness, thus decreasing the use of scarce resources while maintaining great activities. Interestingly, when Co,^326−329^ Fe,^327^ Ni,^328,330^ and Mo^331^ were properly adapted in a specific local configuration, it was possible to tune the reaction pathway to the 2e^–^ ORR process. The DFT calculations indicated that the process always needed the *OOH surface intermediate formation, with Co as the best candidate for the H_2_O_2_ generation in acidic conditions.^328^ As shown in Figure 31, the Co-N-C site has the optimal d-band center, exhibiting high activity and selectivity toward the H_2_O_2_ production when compared with other transition metal (Mn, Fe, Ni, and Cu) SACs. In addition to the intrinsic properties of the metal center, the local coordinated environment also influences the H_2_O_2_ production; i.e., the coordination of O- and S- derived from carbon support could accelerate the *OOH generation.^331^ Qiao and co-workers found that nearby carbon structures such as the C-O-C group influenced the reaction pathway as well (Figure 32).^329^ The authors pointed out that the first and second coordination spheres synergistically determine the ORR activity. Interestingly, DFT calculations confirmed that the optimized *OOH adsorption site on Co-N_4_ was the center Co atom, while for Co-N_2_O_2_ and Co-O_4_(O) it was the C atom adjacent to the coordinated O atom, highlighting the importance of the coordinated environment of the metal center even at further distance than the first coordination sphere to design efficient SACs for selective reaction pathways. Besides the oxygenated group, introducing a Lewis acid also enabled tuning of the ORR reaction pathway. Chen and co-workers discovered theoretically and experimentally that atomically dispersed Lewis acid sites (octahedral M-O species, M = Al, Ga) regulated the electronic structure of the adjacent carbon catalyst sites.^332^

Apart from lab-scale investigations, Zhang and co-workers accomplished a practical electrolysis employing a flow cell with a GDE assembly. This configuration significantly enhances the H_2_O_2_ production using Ni-O_2_N_2_/C SACs materials in alkaline conditions.^330^ The GDE containing Ni-N_2_O_2_/C can generate a high current density using a three-phase flow cell feeding with air, and the H_2_O_2_ generation rate can achieve 5.9 mol g_catalyst_^–1^ h^–1^. Similarly, Lu and co-workers fabricated a GDE with Co-N_4_-carbon SACs enabling translation of lab-scale catalytic systems into practical flow-cell reactors under acidic conditions.^326^ The long-term electrolysis in a flow cell showed a high H_2_O_2_ production rate of 4 mol g_catalyst_^–1^ h^–1^ at a constant concentration (∼1100 ppm). These works bridged the gap between the development of electrocatalysts and their practical applications by scaling up the ORR electrode design for the on-site H_2_O_2_ production technology. However, the issues of scaling up the ORR process and using air as feeding gas still remain important challenges for reliable on-site H_2_O_2_ production.

### 4.3 SACs in 4e^–^ ORR Pathway

Reactions involving the 4e^–^ ORR transfer have found extensive applications in fuel cell technologies, metal-air batteries, and various electroredox coupling reactions involving an oxygen electrode. In recent times, significant focus has been placed on specific transition metal single atom catalysts (SACs), particularly Fe, Co, and Mo, due to their comparable ORR activities while significantly reducing catalyst costs. Isolated single atomic sites coordinated with nitrogen on carbon supports stand out as highly promising alternatives to precious metal-based catalysts. As for ECO2RR, the most investigated architectures were M-N_4_-C structures such as Fe-N_4_-C,^319,333^ Co-N_4_-C,^103^ and Cu-N_4_-C.^334^ Predominant applications of such SACs were used as fuel cell cathodes as well as in the field of rechargeable metal-air batteries, which requires efficient bifunctional catalysts for both the ORR and OER. Recently, Jiao and his co-worker reported a p-block metal SAC (Sb-N_4_) performing as a highly efficient 4e^–^ ORR catalyst, which boarded the category of metal SACs applied for ORR.^335^ Alternatively, the Cu-N-C ORR catalysts such as Cu-N_2_,^336,337^ Cu-N_3_,^338^ and Cu-N_4_^334,339^ have all been claimed to be the active site structures. However, their influence on the ORR selectivity has yet to be provided. As previously discussed, Wang and co-workers demonstrated through *operando* XAS that the active site Cu^+^-N_3_ is more active than the conventional Cu-N_4_ local structure for ORR.^325^ Presumably, the reason is that Cu^+^-N_3_ facilitated the activation of O_2_ by an electron transfer to the antibonding orbital of the oxygen molecule. Thus, it should be noted that tuning a local Cu-N_*x*_ structure could change the ORR reaction pathway. Importantly, it is not only the metal center that plays a significant role; as previously presented, the coordinated elements around this metal center also play a critical role in the selectivity and activity. As Li and co-workers reported, square-pyramidal Fe-N_4_-O-defect sites enabled high intrinsic 4e^–^ ORR activity and durability, where the O-defect served as a promoter.^319^

**Figure 33:**
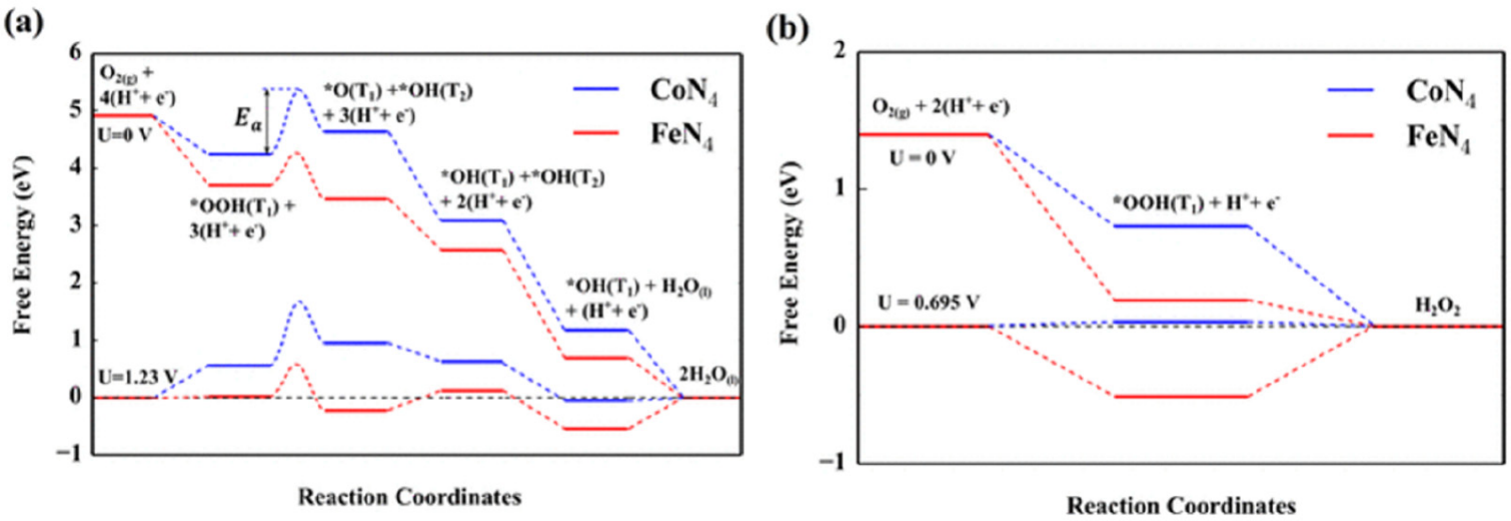
Free energy diagrams of four N-coordinated single atom moiety for ORR in acid medium, including Co sites named CoN_4_ and Fe sites named FeN_4_: (a) the O_2_ reduction through 4e^–^ associative pathway to produce H_2_O and (b) the O_2_ reduction through 2e^–^ pathway to produce H_2_O_2_ on the CoN_4_ and FeN_4_ active sites in acid medium. Reprinted with permission from ref (341). Copyright 2016 American Chemical Society.

Besides the experimental approach, the reaction pathways of ORR on typical M-N_4_ active sites embedded in graphene could be predicted by first-principles DFT calculations. Kattel and Wang calculated the adsorption energy of ORR intermediates and most possible ORR elementary reactions.^340^ They compared the activation energy of the rate-determining steps in three possible pathways: (i) O_2_ dissociation pathway, (ii) *OOH dissociation pathway, and (iii) HOOH dissociation pathways. The *OOH dissociation pathway is kinetically favorable for Fe-N_4_ moieties, with the lowest activation energy of 0.56 eV for the rate-determining step, the dissociation of *OOH, implying the unique role of Fe-N_4_ sites. More importantly, they explained how the 4e^–^ ORR pathway occurred over the single active site. In the initial stage of the reaction, the OOH adsorbs on the metal center of Fe-N_4_. Once the breaking of the O=O bond happens, the dissociated O is adsorbed on the Fe center, while OH is adsorbed on the adjacent carbon atom in the final stage. Similarly, the importance of the metal center was also elucidated by Liu et al.^341^ They compared Co-N_4_ and Fe-N_4_ moieties and revealed that the O_2_ molecules could be favorably adsorbed, while the end product of 4e^–^ ORR-H_2_O could be easily removed from the Fe-N_4_ and Co-N_4_ sites. As shown in Figure 33, the key to achieve high ORR activity over Fe-N_4_ lies in the modest energy barrier (0.56 eV) for the rate-determining step, the O–O bond dissociation of *OOH, compared to the higher one for Co-N_4_ (1.11 eV). The authors demonstrated the pivotal role of the metal center in the M-N_4_ moieties toward the ORR selectivity, since there are different metals in a similar M-N_4_ structure. In other words, the Fe-N_4_ moiety promotes 4e^–^ ORR, whereas Co-N_4_ mainly produces H_2_O_2_ via a 2e^–^ process.

### 4.4 SACs Applied in ORR as an Oxygen Electrode

**Figure 34:**
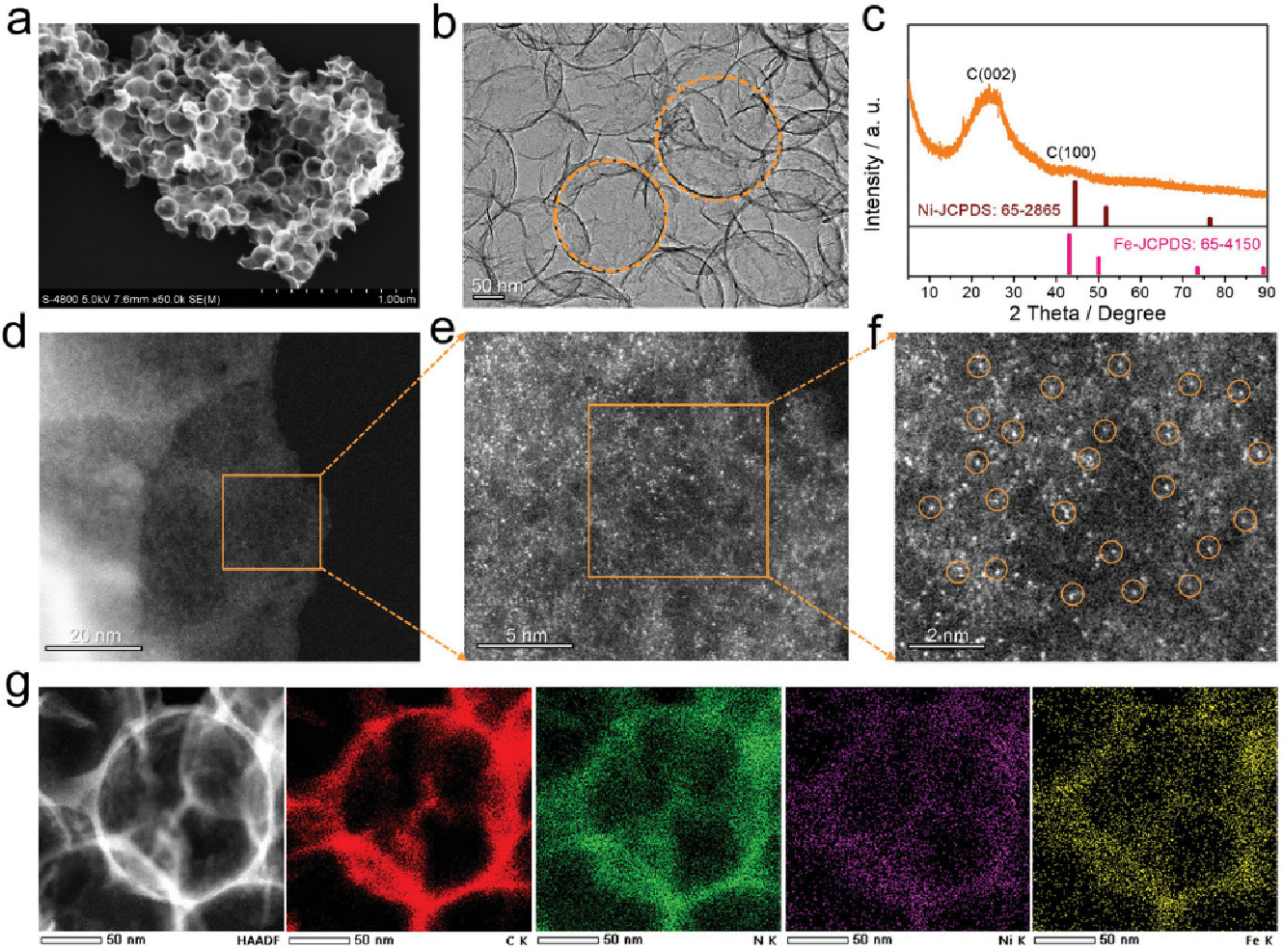
Characterization of Ni, Fe dual-metal over hollow carbon spheres named Ni-N_4_/GHSs/Fe-N_4_: (a) SEM and (b) TEM images of Ni–N_4_/GHSs/Fe-N_4_. (c) XRD pattern of Ni-N_4_/GHSs/Fe-N_4_. (d–f) Aberration-corrected STEM images of Ni-N_4_/GHSs/Fe-N_4_. (g) EDX element mapping images. Reprinted with permission from ref (102). Copyright 2020 Wiley-VCH.

Developing efficient bifunctional ORR/OER electrocatalysts consists in a great challenge, as most of these transition metal SACs could not exhibit high activities in both ORR and OER. Researchers on this topic show significance in evaluating the catalyst stability, since the activity can compare well using noble metal SACs. The replacement of precious metal SACs in the proton exchange membrane fuel cell (PEMFC) requires improvement in stability, compared to the Pt-based system. Some other breakthroughs could be achieved by exploring an anion exchange membrane fuel cell (AEMFC) where transition metals exhibit better catalytic performance or where some other ORR reaction takes place via a microbial fuel cell or metal-air battery. Consequently, some bimetallic SACs are considered as a desirable direction, which could bring hybrid properties rather than an isolated single metal site. Rechargeable metal-air batteries have been under intensive investigation due to their high-energy density, low price, and good safety. Owing to the slow kinetics of reversible oxygen reactions, the charge and discharge processes of the metal-air battery must be catalyzed by the bifunctional catalysts that are active for both OER and ORR. Consequently, atomically dispersed dual-metal catalysts were studied.^102,103,342−344^ Ma and co-workers reported a Ni-Fe bimetallic SAC anchored on hollow carbon spheres named Ni-N_4_/GHSs/Fe-N_4_.^102^ The as-synthesized catalyst inherited the spherical shape with multiple graphene layers from the colloidal SiO_2_ template (Figure 34a–c). The authors observed atomically dispersed Ni and Fe species separately located on the inner and outer walls of hollow graphene, as shown in Figures 34d–f. Interestingly, this material showed homogeneous distribution of N, Ni, and Fe elements throughout the hollow carbon spheres (Figure 34g).

Furthermore, the author assembled the catalyst in a rechargeable ZAB which was able to deliver a superior specific capacity (777.6 mAh g_Zn_^–1^) and energy density (970.4 W h kg_Zn_^–1^), compared to a conventional Pt/C+RuO_2_ air-cathode. Another strategy from Peng and co-workers produced Fe-Ni bimetallic single atoms embedded in a N-doped carbon matrix derived from a Fe/Ni MOFs precursor.^342^ The DFT calculation indicated that the Fe site serves as the active center, while the Ni site regulates the electronic structure of the Fe site, thus reducing the energy barrier of *OOH intermediates formation, the rate-determining step. The authors further assembled this material in quasi-solid-state Zn-and Al-air batteries, achieving the highest power density of 42.22 mW cm^–2^. Bu and co-workers reported another Fe/Co bimetallic SAC embedded in an N-doped graphitic carbon. This catalyst exhibited an excellent electrochemical performance as oxygen electrode and was applied in a rechargeable ZAB.^343^ The author proposed a “pre-constrained metal twins” mechanism, which enabled the construction of highly dispersed and homogeneous adjacent Fe-Co atomic sites. To achieve this target, metal phthalocyanines dimer molecules were implanted into ZIF-8 as precursors. After the pyrolysis, uniform dispersion of dual-metal-center units (containing Co-N_4_ and Fe-N_4_ sites) was achieved. Similarly, Jose et al. designed a Co, Fe bimetallic SAC on N-doped carbon using an imidazole framework to entrap and stabilize the metal precursor.^103^ After calcination, the obtained materials (Fe/Co-SACs) showed an efficient bifunctional catalytic activity for ORR and OER. These Fe/Co-SACs showed an ORR onset potential and half-wave potential of 0.96 and 0.86 V, respectively. For OER, the catalyst attained its anodic current density of 10 mA cm^–2^ at an overpotential of 360 mV. Moreover, it also showed a desirable specific capacity and cyclic stability when serving as an air cathode in a ZAB. Besides dual-metallic, in some cases monometallic Fe single site catalysts could also serve as an excellent oxygen electrode. Yang and co-workers reported a high concentration of Fe-N_4_/C sites embedded in a self-supported flexible carbon membrane, which showed excellent performance as an air cathode in a ZAB.^333^ When tested as the air cathode in a liquid-state ZAB, the catalyst was able to deliver a large peak power density of 255.84 mW cm^–2^ and exhibited long-term cycle durability over 1000 h. Furthermore, the catalyst also showed stable cycling performance when tested using solid electrolytes in various flat/bent states, indicating its potential use in the field of wearable electronics. Zhang and co-workers reported Fe SACs using NH_2_-MIL-101 MOFs as precursors which contain trimeric metal(III) octahedral clusters as nodes and terephthalate ligands as the organic linkers.^344^ This MIL-101-NH_2_ acted as the host due to its high surface area and large pore size. The optimal catalyst was the one calcined at 1000 °C (Fe SAC-MIL101-1000) showing unique and atomically dispersed Fe active sites in the Fe-N_*x*_ moiety. When tested in an aqueous primary ZAB, Fe SAC-MIL101-1000 achieved an impressive energy density of 984.2 Wh kg_Zn_^–1^ (∼91% of the theoretical value). The Fe SAC-MIL101-1000 was also used to assemble a solid-state ZAB, demonstrating a high specific capacity of 724.0 mAh kg_Zn_^–1^. Wu and co-workers reported an atomic dispersion of Fe-N_*x*_ species on N and S codecorated hierarchical carbon layers and employed the material as a bifunctional catalyst for ORR and OER.^105^ This material was applied as an air electrode and tested in a rechargeable ZAB device using KOH as the electrolyte. The cell showed an OCV of 1.35 V, and the maximum power density was as high as 102.7 mW cm^–2^. Moreover, after long-term cycling tests, over 100 cycles, there was no obvious voltage change in the assembling cell. These results clearly demonstrated that the Fe SACs in a N/S codecorated carbon catalyst has great potential in the field of rechargeable ZABs. Similarly, then for Fe SACs, Cu-based SACs were presented as a promising candidate for bifunctional electrocatalysts. Indeed, Zhang et al. proposed a strategy to synthesize scalably densely populated Cu single-atom coordinated with hollow nanospheroids of nitrogen deficient carbon nitride frameworks (namely CuSA@HNCNx).^325^ They obtained impressive performances for both ORR and OER (half-wave potential of 0.91 V for ORR and OER overpotential of 1.55 V at 10 mA cm^–2^ with Δ*E* = 0.64 V after 5000 cycles). Moreover, this SAC was tested as ZAB cathode, revealing high power (212 mW cm^–2^) and energy (1031 Wh kg_Zn_^–1^) densities. In this study and as previously discussed, the critical presence of Cu-N_3_ active sites was highlighted, which could be the reason for such performances.^325^ Furthermore, their synthesis strategy is applicable on other transition metals such as Fe and Co.

In summary for this section, the research into the ORR is crucial for renewable energy applications, especially with respect to fuel cell technologies. The ORR faces challenges due to slow kinetics and multistep reactions, influenced significantly by pH levels. SACs, particularly those based on transition metals coordinated with nitrogen in a carbon matrix (M-N-C), have shown promising ORR activity. Metal–organic frameworks (MOFs) serve as ideal precursors for the SAC synthesis, with recent strategies utilizing MOFs to create SACs like Fe/N-SACs and Co/N-doped porous carbon, which exhibit high ORR catalytic activity. The SACs’ performance varies depending on the different metals, with Fe-N_4_ promoting the 4e^–^ ORR and Co-N_4_ mainly producing H_2_O_2_ through a 2e^–^ process. SACs for ORR reactions require stability improvements, particularly for proton exchange membrane fuel cells (PEMFCs). Nevertheless, recent fuel cell tests with Fe and Co SACs, like 20Co-NC-1100, have demonstrated durable performance, achieving high power densities and voltage stability in PEMFCs. Bimetallic SACs, such as Ni-Fe, Fe-Ni, and Fe-Co, exhibit promising catalytic activities and find applications in zinc-air batteries (ZABs) and fuel cells, showing high power densities and energy densities. Host–guest strategies incorporating Fe-Co dual sites in N-doped porous carbon (Fe/Co-N-C) and Fe SACs embedded in carbon nanotubes (CNT/PC) exhibit exceptional stability and activity, enhancing the ORR selectivity and the membrane electrode assembly (MEA) performance across various electrolytes. These advancements showcase the significance of high ORR activity in advancing fuel cell technologies.

### 4.5 Deployment of SACs as Cathodes Applied in Fuel Cells

**Figure 35:**
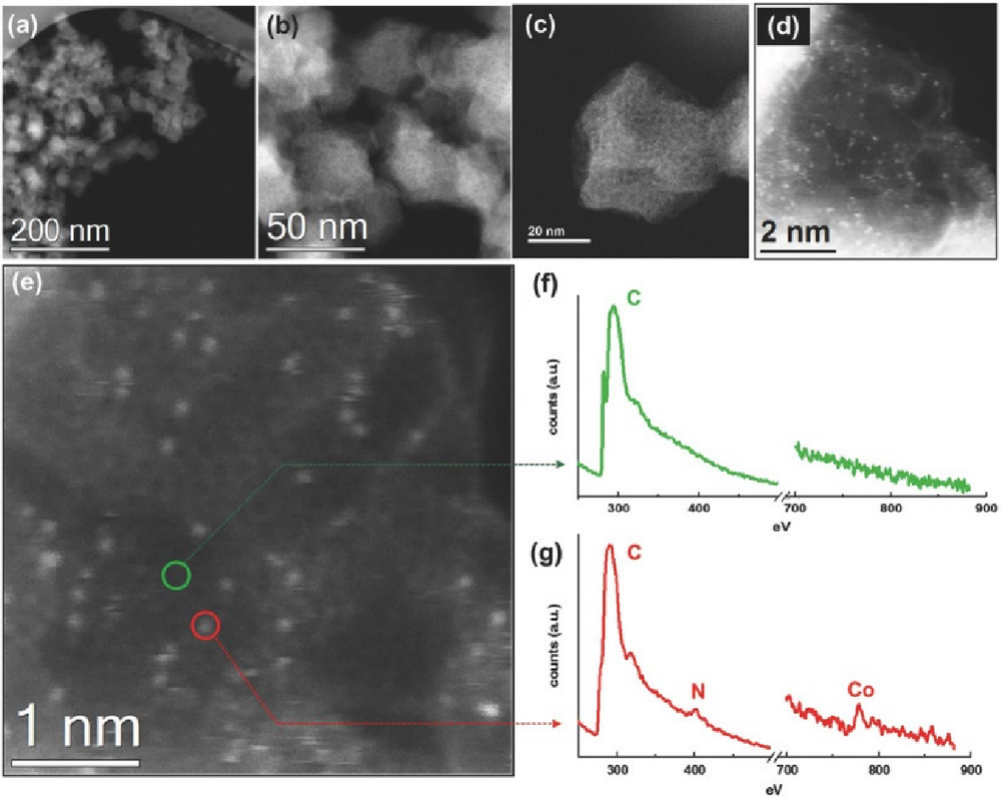
Characterization of atomically dispersed Co single site on N-doped carbon derived from Co-ZIF named as 20Co-NC-1100: (a–d) Aberration-corrected MAADF-STEM images, (e) atomic resolution STEM analysis, and (f–g) EEL point spectra analysis. The point spectrum in (f) was taken at the dark neighboring support area in (e) and only shows C and no N and Co. The point spectrum (g) was taken on the bright atom in (e) and shows both Co and N, indicating that Co is coordinated with N at the atomic scale. Reprinted with permission from ref (346). Copyright 2018 Wiley-VCH.

Beyond the screening of ORR active SACs in a half-cell setup, valuable evaluations were carried out in H_2_ fuel cell devices which were extensively delivered to the public. For example, Zhang and co-workers reported a Fe SAC for ORR in PEMFC starting from a MOF-5 precursor.^345^ These as-synthesized materials showed an ultrahigh density of Fe SAs (2.35 wt %), delivering a half-wave potential of 0.83 V vs RHE in a 0.5 M H_2_SO_4_ electrolyte, and achieved a peak power density of 0.84 W cm^–2^ tested in a 0.2 MPa hydrogen PEMFC. Furthermore, the material was also tested under 0.1 MPa H_2_-air conditions and achieved a maximum power density of 0.31 W cm^–2^. Li et al. reported a Fe SAC supported on a nitrogen, phosphorus, and sulfur codoped hollow carbon (Fe-SAs/NPS-HC) acting as ORR cathode in PEMFCs.^112^ When tested in H_2_/air PEMFC at 60 °C, Fe-SAs/NPS-HC-based MEA exhibited a remarkable current density of ∼50 mA cm^–2^ at 0.8 V. The maximum power density of Fe-SAs/NPS-HC-based MEA was 333 mW cm^–2^ at 0.41 V, which was ∼92% of the power density of a commercial Pt/C-based MEA under identical test conditions. When tested at 80 °C, the Fe-SAs/NPS-HC-based MEA reached a high-power density of 400 mW cm^–2^ at 0.40 V, approaching a commercial Pt/C-based MEA. Wu and co-workers reported a high-performance atomically dispersed Co catalyst (20Co-NC-1100) synthesized by a facile one-step thermal activation of chemically Co doped ZIF precursors.^346^ This catalyst revealed homogeneous carbon particles around 50 nm in diameter (Figure 35a–c). The author further concluded that the Co sites were highly dispersed on the carbon matrix with most possibly a Co-N_4_ coordination local structure and have a similar local chemical environment to the Co-N_4_ in Co-porphyrin structures (Figures 35d–g).

**Figure 36:**
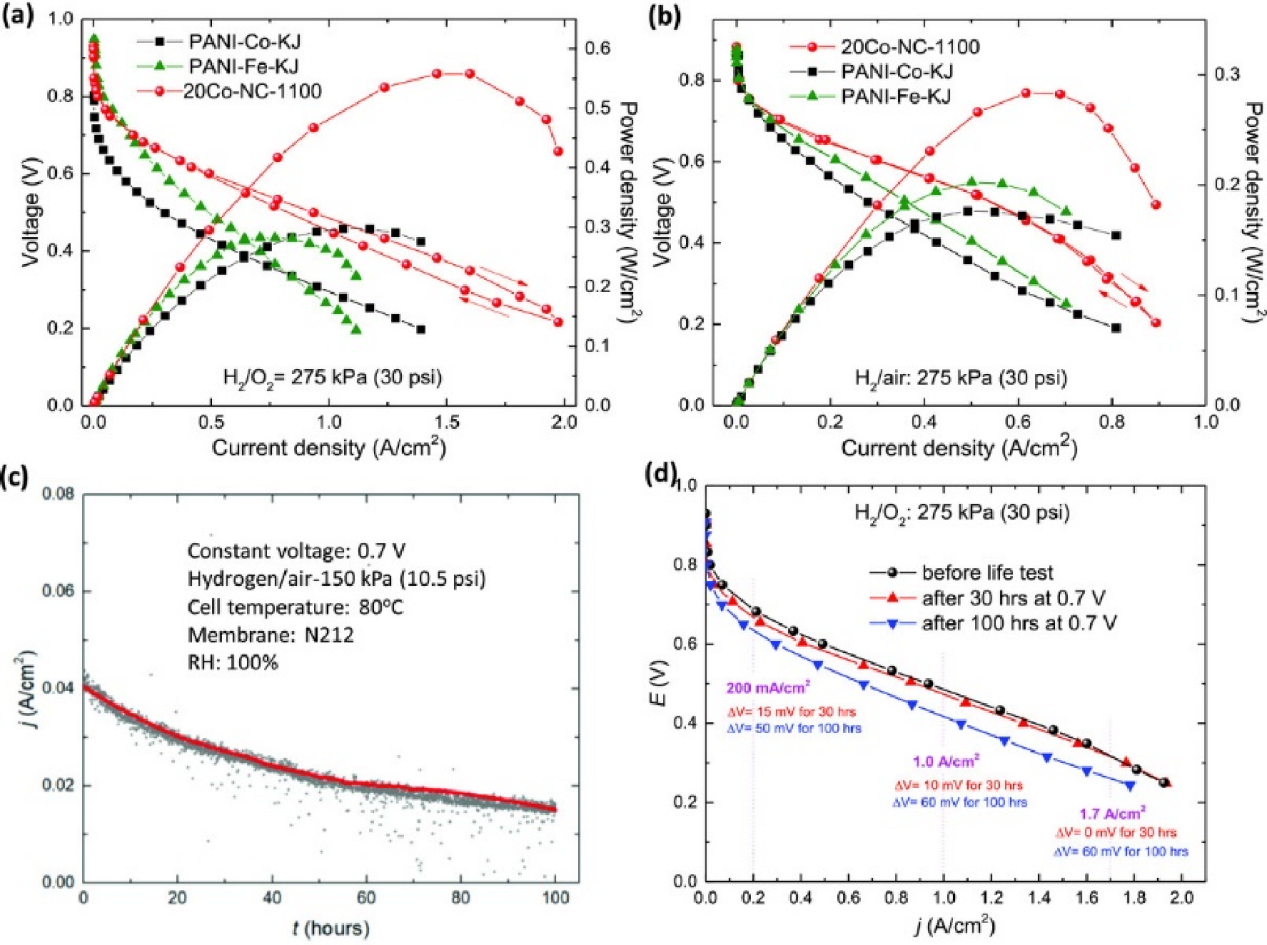
Fuel cell performance and durability tests of single-atomic Co site on N doped carbon named as 20Co-NC-1100 and reference materials. (a) H_2_/O_2_ fuel cell polarization plots. (b) H_2_/air fuel cell polarization plots. Test condition: the cathode 4.0 mg cm^–2^; O_2_ (air) 200 mL min^–1^; 100% relative humidity (RH); 275 kPa (30 psi) backpressure; anode: 0.2 mgPt cm^–2^ Pt/C; H_2_ 200 mL min^–1^; 100% RH; membrane: Nafion 212; cell: 80 °C. (c) 100 h durability test under H_2_/air fuel cell test conditions at 0.7 V with 150 kPa (10.5 psi) back pressure. (d) H_2_/O_2_ polarization plots before and after the 100 h lifetime test at 0.7 V. Reprinted with permission from ref (346). Copyright 2018 Wiley-VCH.

The H_2_/O_2_ PEMFC performance was tested using the optimal Co SACs (calcined at 1100 °C and named 20Co-NC-1100) as the cathode. As shown in Figure 36a, the highest power density of 0.56 W cm^–2^ and the OCV up to 0.95 V were attained. This performance enhancement was also observed when H_2_/air was used for fuel cell tests. The power density of 20Co-NC-1100 achieved 0.28 W cm^–2^ when tested in a H_2_/air fuel cell (Figure 36b). The authors conducted the durability test at the fully viable voltage of 0.7 V for 100 h, when feeding air in the cathode, and collected the voltage–current polarization curve to monitor the possible degradation. During the initial 30 h, there were insignificant losses (less than 15 mV) at all current density ranges, and the 100 h continuous operation eventually resulted in a loss of ∼60 mV. All observations showed that the 20Co-NC-1100 catalyst exhibited a durable performance in a practical fuel cell test (Figure 36c, d).

**Figure 37:**
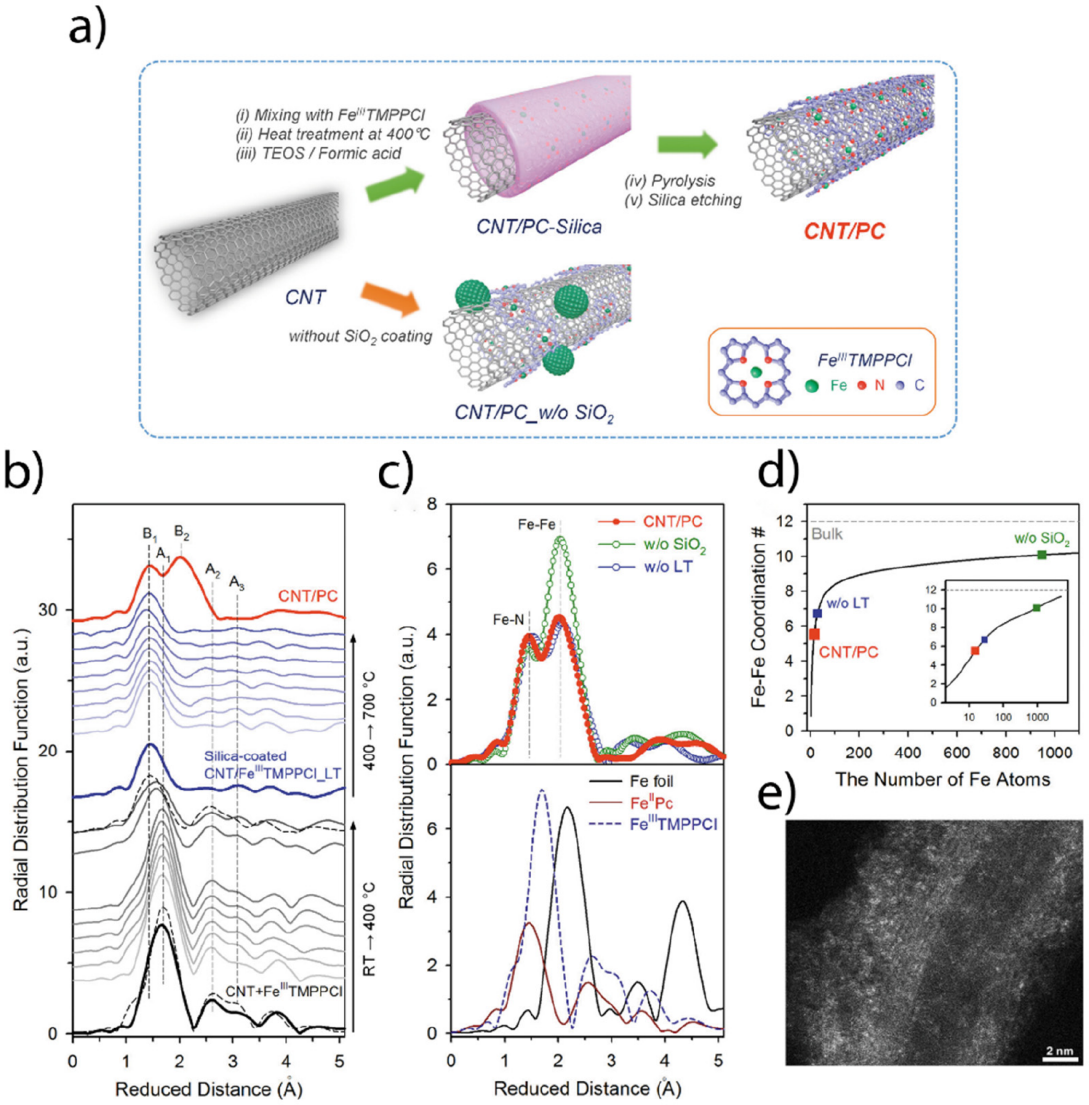
(a) Schematic of single-atomic Fe site on CNT, named as CNT/PC catalysts. (b) Temperature-dependent radial distribution functions of Fourier transform of k3-weighted Fe K-edge EXAFS spectra during two-step pyrolysis of CNT/PC. Those of Fe(III) TMPPCl (bottom) and Fe(II) Pc (top) are indicated by the dashed lines. (c) RDFs of Fourier transform of k3-weighted Fe K-edge EXAFS spectra of CNT/PC and control samples. (d) Plot for the relation between Fe-Fe coordination number and the number of Fe atoms; the inset is the logarithmic representation. (e) HAADF-STEM image of CNT/PC. Reprinted with permission from ref (349). Copyright 2016 American Chemical Society.

The same group exploited a Fe SAC (ZIF-NC-0.5Fe-700) as a cathode in acidic PEMFCs with air feeding.^347^ The authors evaluated its performance under standard pressure (1.0 bar) for H_2_ and O_2_. The catalyst exhibited an OCV up to 0.98 V and a remarkable current density of 0.030 A cm^–2^ at 0.9 V cell potential. Under practical conditions with 1.0 bar of air feeding, the current densities generated at 0.8 and 0.7 V were 0.075 and 0.302 A cm^–2^, respectively. The values are comparable to the record performance of Fe-N-C catalysts in MEA fuel cells. Li and co-workers developed a host–guest strategy to design electrocatalysts with Fe-Co dual sites embedded on N-doped porous carbon (Fe/Co-N-C) and demonstrated their activity for ORR in acidic condition.^348^ The obtained Fe-Co bimetallic SACs seem to be one of the most active ones among the reported Pt-free catalysts tested in H_2_/O_2_ and H_2_/air fuel cells. In addition, this cathode fabricated with this Fe/Co-N-C composition is stable during a long-term operation comprising 50 000 cycles of the electrode measurement and 100 h of electrolysis in a H_2_/air single-cell operation. The DFT calculation confirmed that this superb ORR activity was due to the acceleration of the rate-limiting step - the O_2_ activation. Ultimately, dual-site SACs can reduce the cleavage barrier of the O–O bond to achieve high activity and high selectivity for the 4e^–^ ORR pathway. Joo and co-workers reported Fe SACs embedded on CNTs with Fe-N_*x*_ coordination (named CNT/PC) via high-temperature treatment with a silica-assisted hard-template protection.^349^ As depicted in Figure 37a, the synthesis proceeds with a SiO_2_ template coated together with the Fe complex (iron porphyrin) precursors over a CNT, which suppresses the Fe particles formation. Compared with the samples without the silica coating (named CNT/PC_w/o SiO_2_) and without pyrolysis (named CNT/PC_w/o LT), the author demonstrated that the Fe single sites with a Fe-N_*x*_ moiety were generated during the heating treatment (Figures 37b, c), thus confirming the critical dependence of the metallic cluster growth during the silica coating step. The number of Fe atoms presented as metallic Fe in the CNT/PC was estimated to be ∼10, corresponding to a few angstroms in size, whereas CNT/PC_w/o SiO_2_ contained on average 1000 Fe atoms per Fe particle (Figure 37d). The authors also clarified that a very small amount of Fe clusters in the CNT/PC could be directly observed from the HAADF-STEM images (Figure 37e), corresponding to sub-nanometer particles corresponding to a few Fe atoms or even to the monatomic dispersion of Fe-N_*x*_ sites.

**Figure 38:**
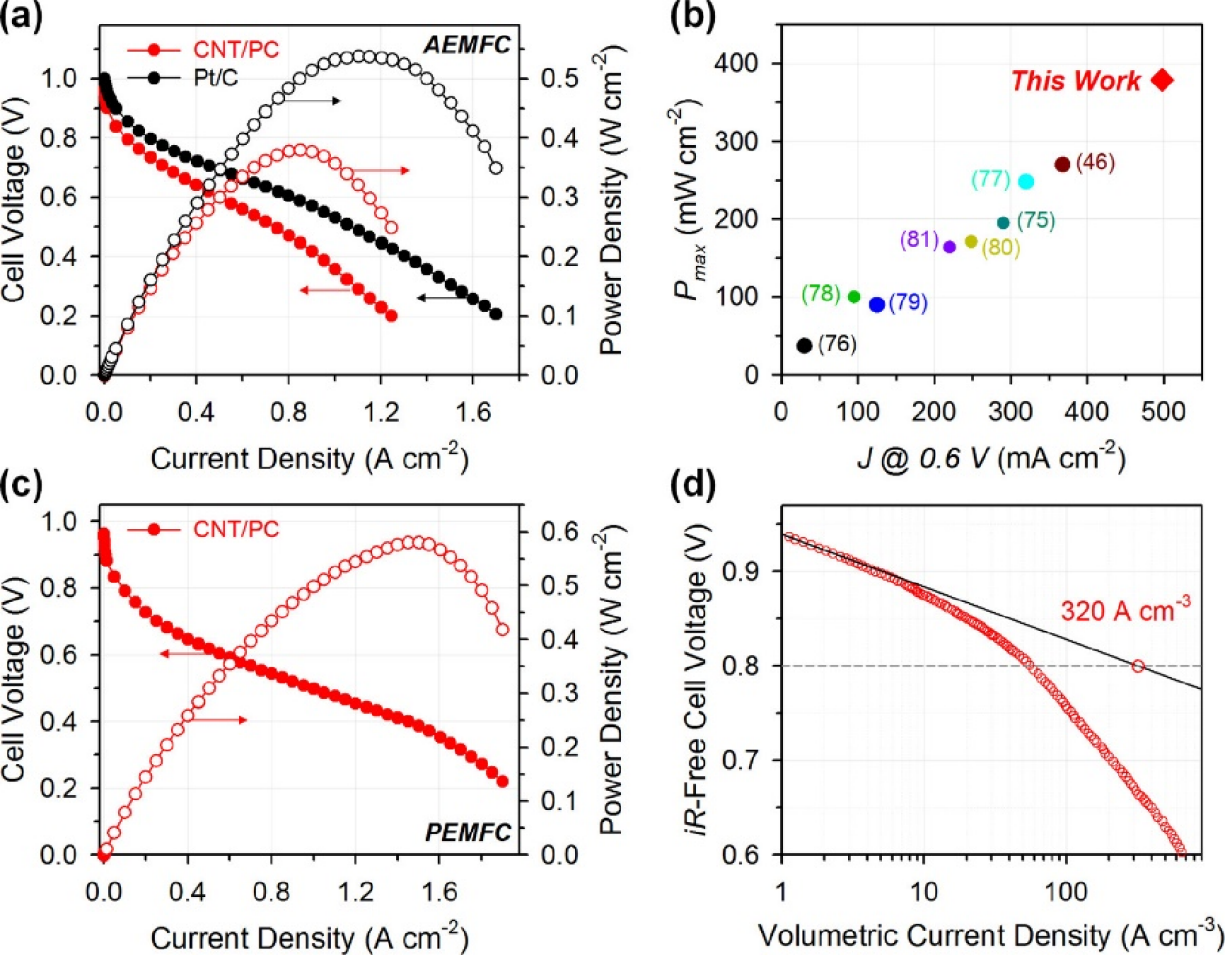
Single cell performances of Fe single site catalyst on N-doped carbon named CNT/PC. (a) Alkaline AEMFC performances of CNT/PC-based MEA and Pt/C as cathode catalysts. (b) Comparison of current density at 0.6 V and peak power density of CNT/PC-based AEMFC with those of previously reported AEMFCs based on NPMC-based MEAs. The numbers in the parentheses denote the reference numbers. (c) Acidic PEMFC performance of CNT/PC-based MEA. (d) Volumetric current densities of the CNT/PC tested in the PEMFC. Reprinted with permission from ref (349). Copyright 2016 American Chemical Society.

The authors fabricated PEMFC and AEMFC using these CNT/PC catalysts as the cathode. The polarization and power density curves indicated that the CNT/PC-based MEA exhibited nearly as high performance as AEMFC (Figure 38a). The current density at 0.6 V and the peak power density of the Fe SACs-based MEA in the AEMFC were 498 mA cm^–2^ and 0.38 W cm^–2^, respectively. The Fe SACs-based MEA in the PEMFC exhibited a current density of 550 mA cm^–2^ at 0.6 V and a peak power density of 0.58 W cm^–2^, respectively. As shown in Figure 38d, the test in a PEMFC showed a high volumetric current density of 320 A cm^–3^. The excellent single stack performances indicated that the high ORR activity of the SACs Fe-CNT/PC catalyst significantly enhanced the MEA performances in both alkaline and acidic electrolytes.

In conclusion, there has been recently a notable emphasis on improving PEMFCs through the development of non-noble metal ORR catalysts. Synthesis methods, particularly sacrificial-template approaches involving MOFs, significantly influence the catalyst activity and stability. However, some challenges prevail, including cost reduction, precise control of synthesis conditions, exploration of different transition metal atoms, optimization of carbon carriers, and improving the catalyst performance in acidic conditions. While some non-noble metal catalysts have shown remarkable activity, achieving real-world durability remains a hurdle. Future efforts should prioritize catalysts that are highly active, durable, affordable, and scalable, thus aiming for large-scale production and application.

## 5 EARTH-ABUNDANT SACs IN HYDROGEN EVOLUTION REACTION (HER)

Driven by energy and environmental issues, the research on developing green energy to replace fossil fuels has garnered immense attention.^350−352^ As one of the most ideal green energy sources, the effective acquisition of hydrogen has important practical significance.^353,354^ To date, hydrogen energy mainly comes from oil cracking. However, this process produces large amounts of pollution. Recently, researchers found that water splitting through electrocatalysis for HER based on renewable energy can effectively alleviate the current environmental problems.^355−357^ The most commonly used catalysts for HER are PGMs, which seriously limit the applicability for wide commercial applications because of their low crustal reserves and prices.^358^ Among the emerging materials, SACs exhibit a large potential to facilitate energy conversion due to the atomic utilization maximization during the catalysis.^34,359^ It is theoretically possible to reach the same or even higher activity for SACs with loading of earth-abundant transition metals (e.g., Co, Fe, Ni, Mo, etc.) compared to the commercial PGM catalysts. Moreover, nonmetallic SACs can also be developed with high HER catalytic activity. This section will discuss the different possible reaction routes for HER followed by details on the recent advances on the earth-abundant SAC synthesis applied to H_2_ production.

### 5.1 Reaction Routes and Mechanisms of HER

The reaction mechanism of HER is greatly affected by the pH of the electrolyte. Generally, HER consists of multiple electron–proton transfer processes, including adsorption, reduction, and desorption. The accepted possible HER mechanisms in acidic and alkaline media include at least three reaction pathways.^27,360^ The detailed mechanisms are summarized below.

In acidic solution:
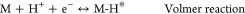
11
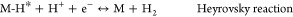
12
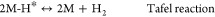
13
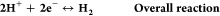
14

In alkaline solution:
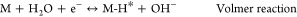
15
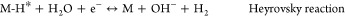
16
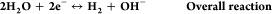
17

The HER always involves the hydrogen adsorption (H_ads_) step. This step, called the Volmer step (eqs 11, 15) is the reaction between the surface of the catalyst and the proton source available in the electrolyte (H^+^ in acidic and H_2_O in alkaline conditions) to form M-H* intermediates at the catalyst surface. On one hand, the M-H* intermediates can react with themselves to generate H_2_ gas, known as the Tafel reaction (eq 13) which can happen in both conditions. On another hand, the M-H* intermediate continues to react with the proton source from the electrolyte to produce H_2_; this process is called the Heyrovsky reaction (eqs 12, 16). The overall HER reaction in acidic solution is 2H^+^ + 2e^–^ ↔ H_2_ (eq 14). In comparison with the acidic condition, the HER mechanism in alkaline solution depends on the water adsorption and dissociation, as, there, water is the proton source. The overall HER reaction in an alkaline solution is 2H_2_O + 2e^–^ ↔ H_2_ + OH^–^ (eq 17).

### 5.2 Construction of SACs for HER Applications

**Figure 39:**
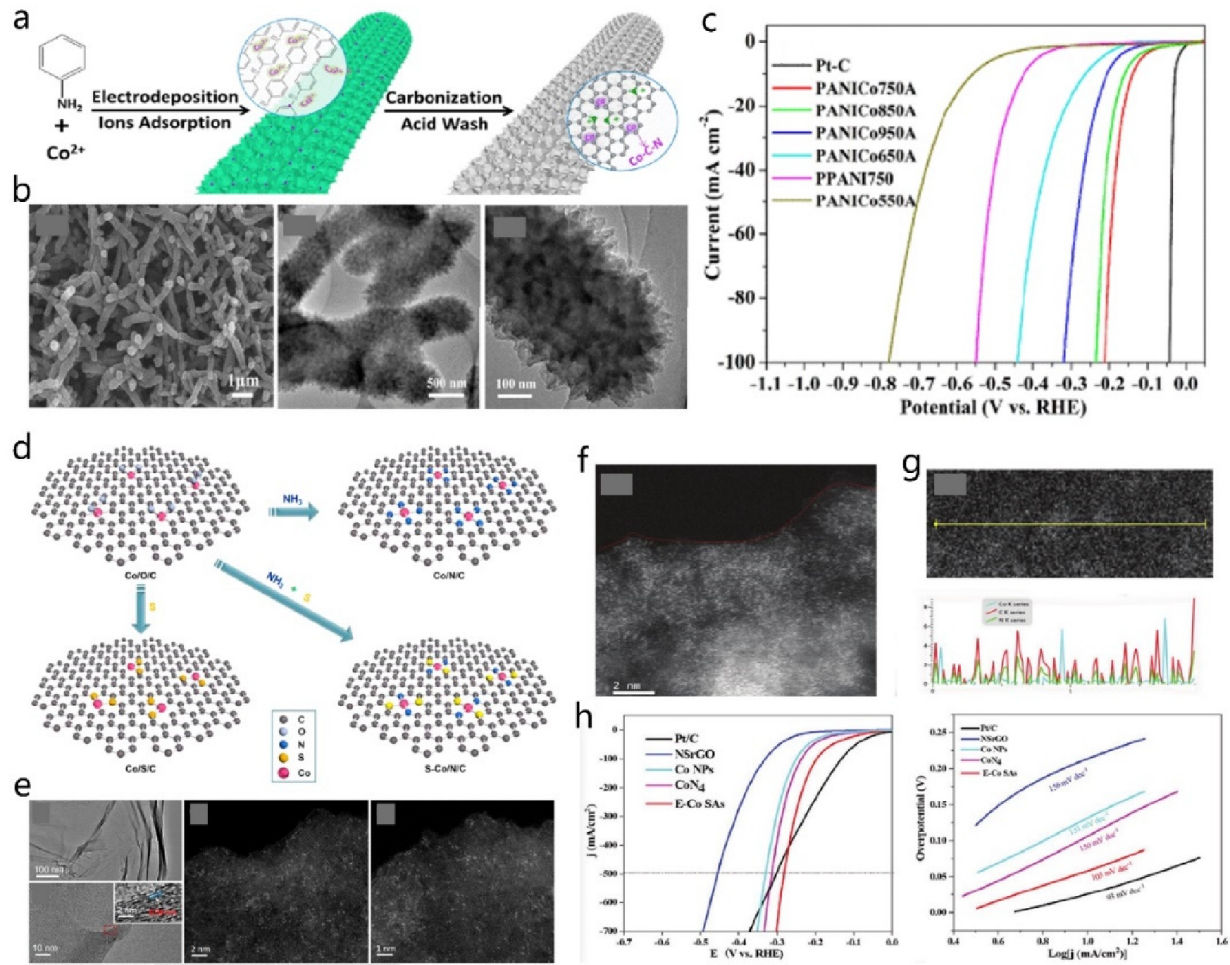
(a) Schematic diagram of the synthesis of Co-N-C&CF complex. (b) SEM and TEM images of the Co-N-C&CF. (c) Polarization curves of the series of Co-N-C&CF materials processed under different temperature in 0.5 M H_2_SO_4_. Reprinted with permission from ref (361). Copyright 2015 American Chemical Society. (d) Schematic illustration of the reparation process for Co-N-C-S-O SACs. (e) HR-TEM images and corresponding STEM images of the Co-N-C-S-O SACs. Reprinted with permission from ref (362). Copyright 2021 American Chemical Society. (f, g) STEM image and EDS line scanning of the edge-rich Co SACs. (h) Linear sweep voltammetry (LSV) curves and the Tafel slopes of the synthesized materials. Reprinted with permission from ref (363). Copyright 2021 Wiley-VCH.

The chemical hybridization between the SAs and the vicinal atoms in the supported materials offers the special coordination environment of SACs in comparison to surface atoms in nanoparticles. These unique electronic properties endow the SACs with remarkable electrocatalytic performance. To develop different SACs, various substrates were synthesized to provide matched atomic coordination environments for HER application. Wang et al. developed a novel method to fabricate a 2D porous Co-N-C complex supported on the CF through the carbonization.^361^ As illustrated in Figure 39a, the schematic diagram shows the simple synthesis process. First, the polyaniline (PANI) precursor was electrodeposited on the surface of CFs which absorbed the cobalt ions. Subsequently, the Co–N–C bond was formed after carbonization at high temperature. The final structure of the Co-N-C&CF hybrid materials could be obtained after the acid wash dissolving the nanoparticles present on the carbon support. The SEM images in Figure 39b show that the 1D CF was coated by active Co-N-C with an average diameter of 500 nm. The TEM images in Figure 39b show that 2D Co-N-C nanosheets were randomly dispersed on the CF surface. The sample treated at 750 °C exhibited the closest HER activity to that of commercial Pt/C with an overpotential of 138 mV at 10 mA cm^–2^.

To change the local electron configuration around Co SAs for higher HER activity, Sun and co-workers used a heteroatom doping strategy to prepare Co-N-C-S-O SACs. The mixture of prepared Co-O-C sample was treated in high temperature under ammonia gas and S vapors at 600 °C to form the Co-based SACs composed by four nonmetallic elements (N-C-S-O).^362^ Three types of Co-SACs were designed by changing the coordination with vicinal atoms to obtain S-Co/N/C, Co/N/C, and Co/S/C SACs as illustrated in Figure 39d. The HR-TEM and atomic resolution STEM images (Figure 39e) clearly show the dispersion of isolated metal atoms on the substrate. As expected, the S-Co/N/C SACs exhibited excellent HER activity, especially under high current density.^15^ Similarly, Liu et al. synthesized edge-rich Co-N-C SACs where the Co atoms are mainly located on the edge of the substrate.^363^ Based on the Athena fitting analysis, 65.49% Co atoms located on the Co-4N plane, 13.64% Co atoms existed in the Co-2N-armchair, and 20.86% Co atoms existed in Co2N-zigzag.^363^ The STEM image (Figure 39f) showed the atomically dispersed metals on the C-N substrate, and the EDS line scanning profiles (Figure 39g) further verified the dispersed Co metal atoms. More importantly, the edge-rich Co SACs showed higher HER activity than commercial Pt/C at high current density above 400 mA cm^–2^, revealing the great application potential of Co-SACs for HER (Figure 39h).^363^

**Figure 40:**
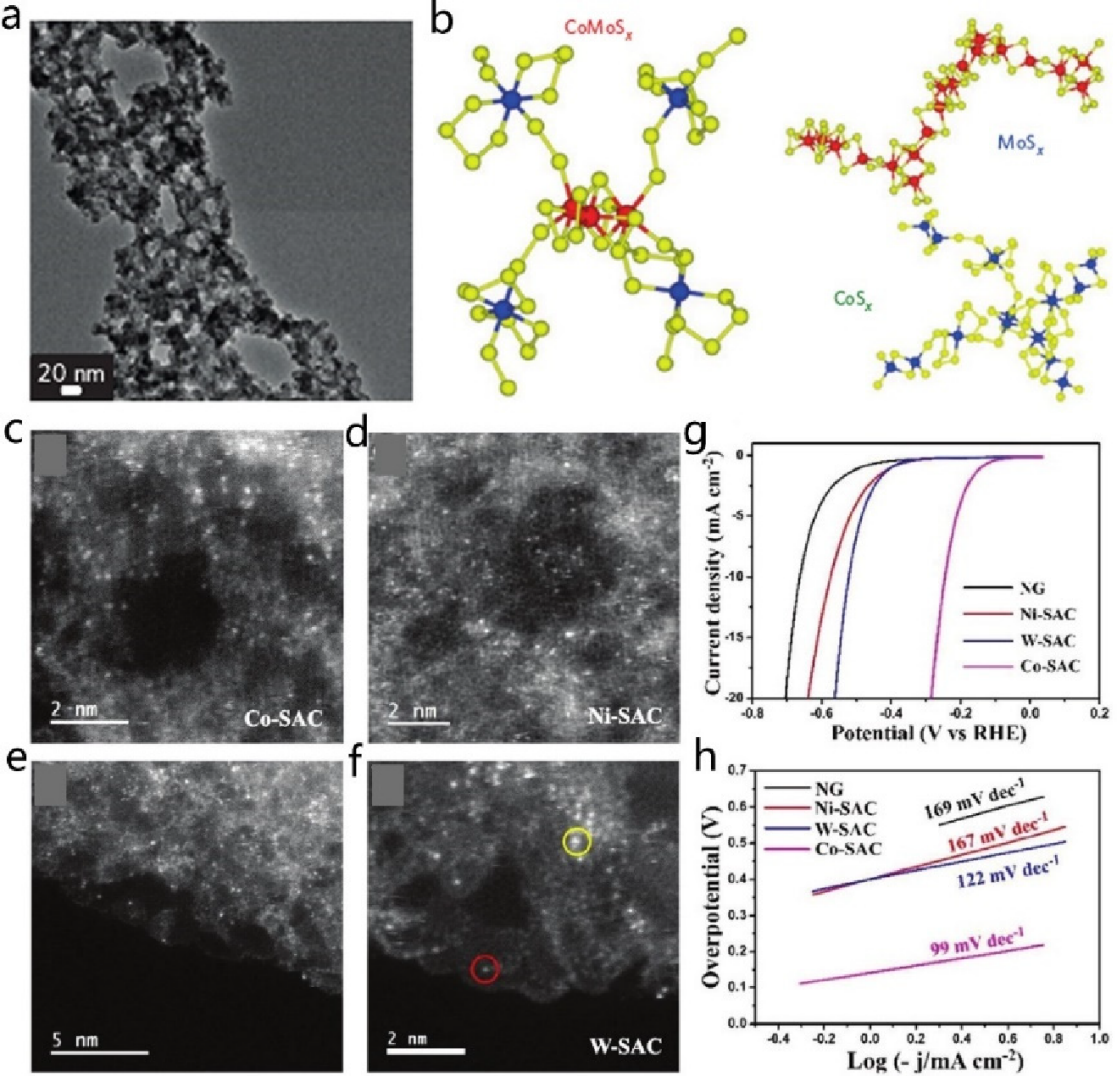
(a) TEM image of the CoMoS_*x*_ catalyst. (b) The proposed CoMoS_*x*_ model structure. Reprinted with permission from ref (364). Copyright 2015 Springer Nature. (c, d) ADF-STEM images of the Co-SAC, Ni-SAC, and (e, f) W-SAC, respectively. (g, h) LSV polarization curves and the corresponding Tafel slopes of the Co-SAC, Ni-SAC, and W-SAC catalysts. Reprinted with permission from ref (365). Copyright 2019 Wiley-VCH.

The metal site position and coordination environment play a crucial role for the catalyst activity. Investigating the working mechanism of the catalytic site has been instrumental to understand the catalytic mechanism. Staszak-Jirkovský and co-workers prepared a CoMoS_*x*_ catalyst, and they found that Co^2+^ and Mo^4+^ sites promote the original decomposition of water in alkaline solutions.^364^ The morphology of CoMoS_*x*_ was characterized by TEM (Figure 40a). The porous structure was composed of aggregated nanoparticles distributed between 50 to 100 nm in size. The proposed crystal models in Figure 40b clearly revealed the chain units of CoS_*x*_ and MoS_*x*_, while the CoMoS_*x*_ showed the MoS_*x*_ cluster connected to CoS_*x*_, revealing that the structure coordination is critical on the catalytic activity. Carbon materials are ideal support to load SAs, due to their affordable cost and excellent electrical conductivity, yet their weak coordination ability represent a limitation. Fortunately, doping N atoms into the carbon materials can improve the coordination ability of the substrate. For instance, Hossain et al. used acrylamides as nitrogen precursors to prepare N-doped carbon support.^365^ A variety of SACs including Co-SAC, Ni-SAC, and W-SAC were synthesized based on this support preparation strategy. Atomic-resolution STEM characterization results (Figures 40c–f) showed the abundant single atoms dispersed on the surface of those C-N substrates. Surprisingly, the W-SACs presented better HER activity than Co-SAC and Ni-SAC (Figures 40g, h).

**Figure 41:**
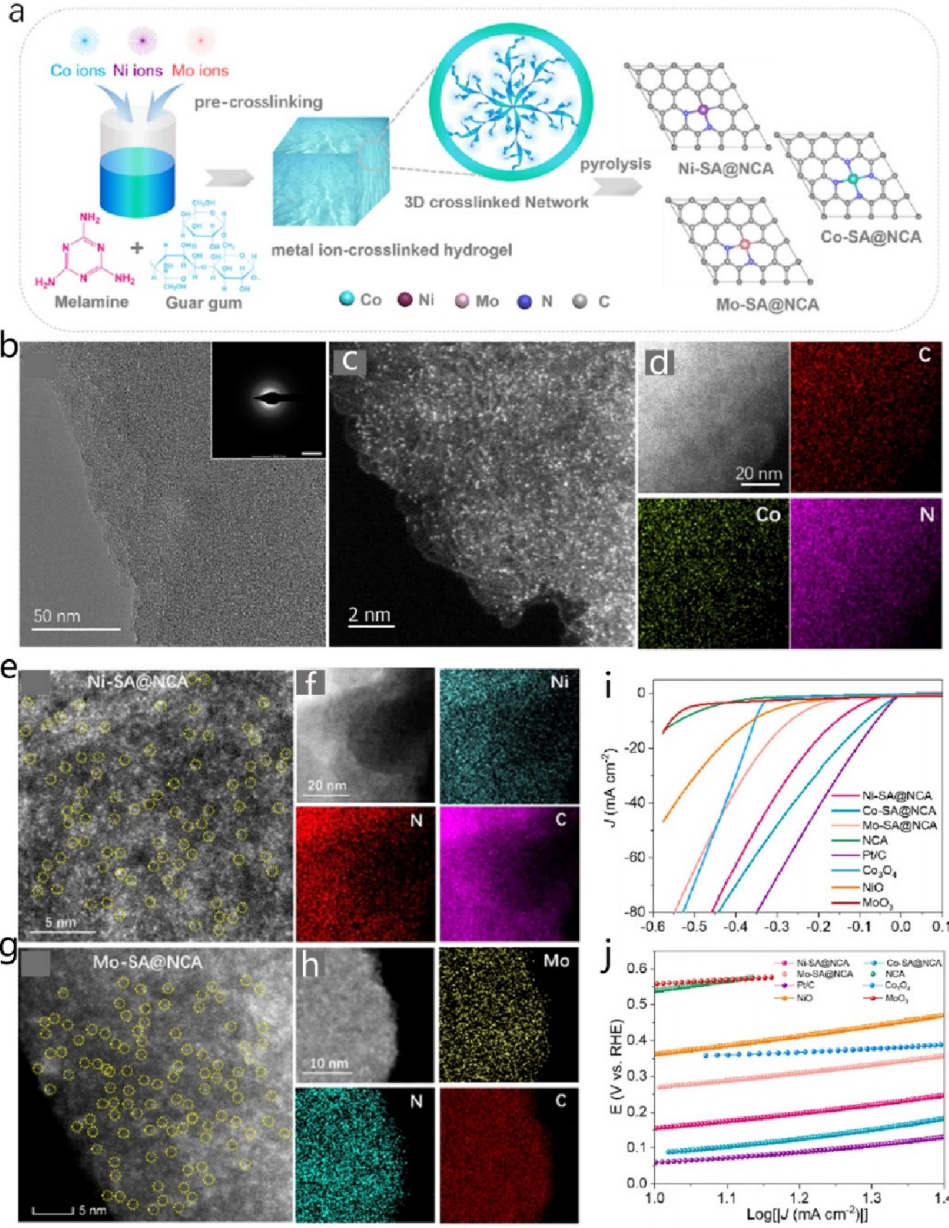
(a) Synthesis schematic of the Ni-SAC, Co-SAC, and Mo-SAC. (b) HRTEM and (c) STEM images of the Co-SAC. (d) The STEM and the corresponding EDS mapping images of Co-SAC. (e, f) Ni SA highlighted by the yellow cycles and the EDS mapping images. (g, h) Mo SA highlighted by the yellow cycles and the EDS mapping images. (i, j) The LSV polarization curves of the SACs compared with the Pt/C, Co_3_O_4_, NiO, and MoO_3_. Reprinted with permission from ref (366). Copyright 2021 Elsevier.

The expansion of the synthetic methodology has been of great significance for the practical application of SACs. Cheng et al. used an *in situ* pre-cross-linking method to prepare Co, Ni, Mo-SACs.^366^ First, melamine was mixed with guar gum and metal ions to complete the pre-cross-linking. The metal ion-cross-linked hydrogel showed a 3D cross-linked network structure. After pyrolysis under high temperature, the different types of SACs were obtained as shown in Figure 41a. The TEM and STEM images (Figure 41b–h) reveal the atomic distribution of the Co, Ni, and Mo SACs. The elemental species of Co, Ni, Mo were further verified by EDX mapping results (Figure 41f, h). However, the HER performance of the synthesized SACs and the corresponding Tafel slopes (Figure 41i, j) did not meet the desired results, which might be related to the loading of SA metals.

**Figure 42:**
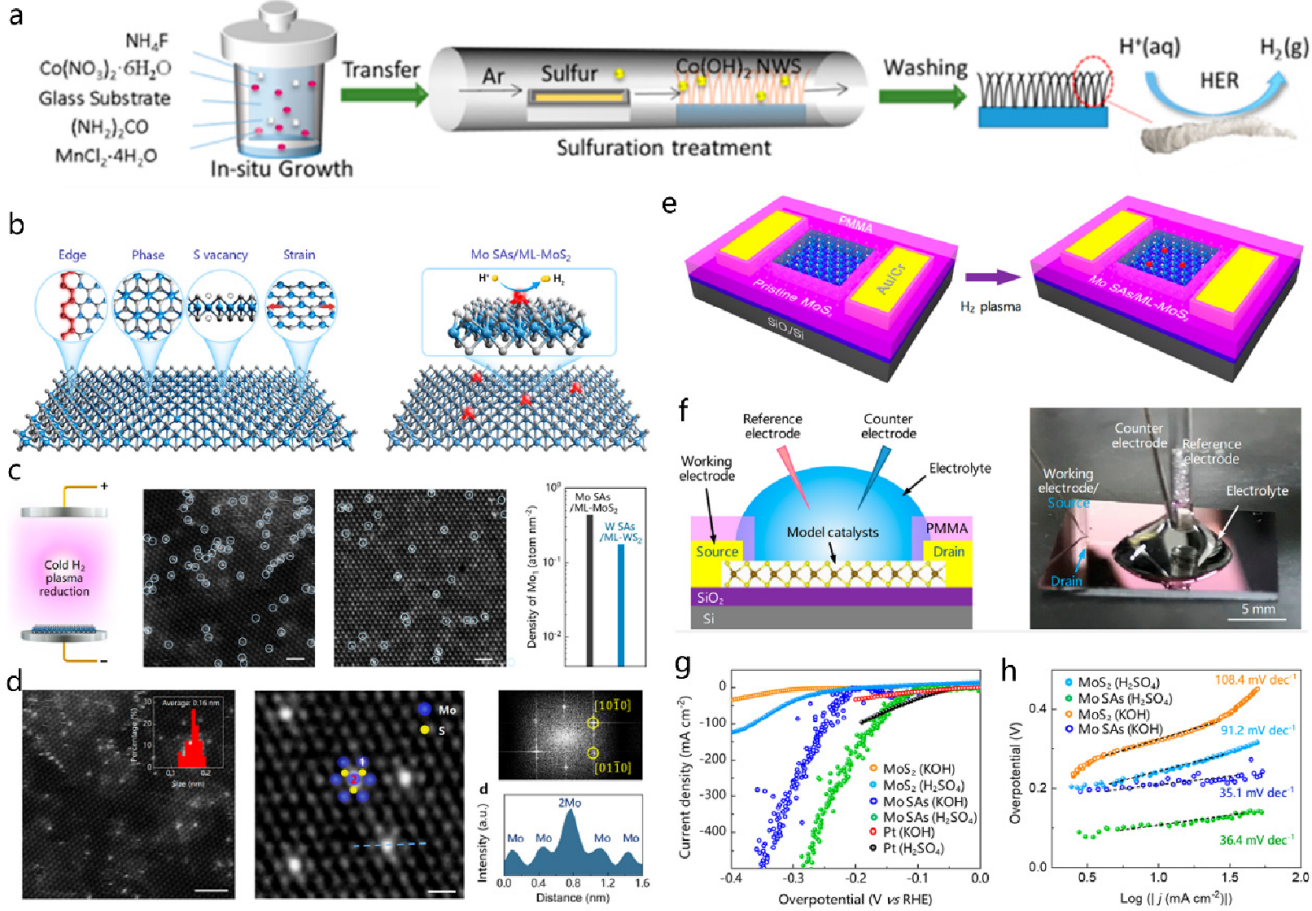
(a) Schematic of Mn-doped CoS_2_ nanowires preparation progress. Reprinted with permission from ref (367). Copyright 2018 American Chemical Society. (b) Formation of Mo SAs on 2D MoS_2_ with different loading sites, including S vacancies, edges, and strain. (c, d) Atomic-resolution STEM images of the Mo SAs supported on the monolayer MoS_2_. (e) Experimental schematic for the preparation of Mo SAs/MoS_2_ complex. (f) Electrolytic cell construction for the HER test. (g, h) LSV curves and the corresponding Tafel slopes of the different catalysts. Reprinted with permission from ref (368). Copyright 2019 American Chemical Society.

For other improvements of the activity performance of SACs, the SA sites are anchored on the active substrate to attain impressive HER catalytic activities. Zhang et al. prepared Mn-doped CoS_2_ catalysts through gas phase reaction in a tube furnace as shown in the preparation schematic of Mn-doped CoS_2_ catalysts (Figure 42a).^367^ During the HER test, the 5% doped CoS_2_ presented higher HER activity (43 mV at 10 mA cm^–2^) than 1% doped CoS_2_ with the overpotential close to that of commercial Pt/C. The doped Mn atoms could either activate the neighboring Co atoms or function as new HER active sites, thus ultimately lowering the Gibbs free energy to enhance the HER activity of those Mn-CoS_2_ complexes. Furthermore, establishing a suitable method for *in situ* fabrication of SA sites on supported substrates could effectively improve the catalytic activity. Luo et al. used the cold hydrogen plasma reduction method to prepare SACs on 2D monolayers.^368^ This *in situ* physically driven synthesis approach could construct SACs without additional surfactants, which would not interfere with the activity of SAs (Figures 42b). The STEM images (Figures 42c, d) showed highly dispersed Mo SAs on the MoS_2_ monolayer. Notably, the MoS_2_ monolayer was supported on a silica substrate, while liquid electrolytic cells were constructed for the HER test. In the acidic condition, the Mo SACs on MoS_2_ exhibited the overpotential as low as 261 mV at a current density of 400 mA cm^–2^, together with the lowest Tafel slope of 36.4 mV dec^–1^, revealing better HER activity than that of MoS_2_.^368^

**Figure 43:**
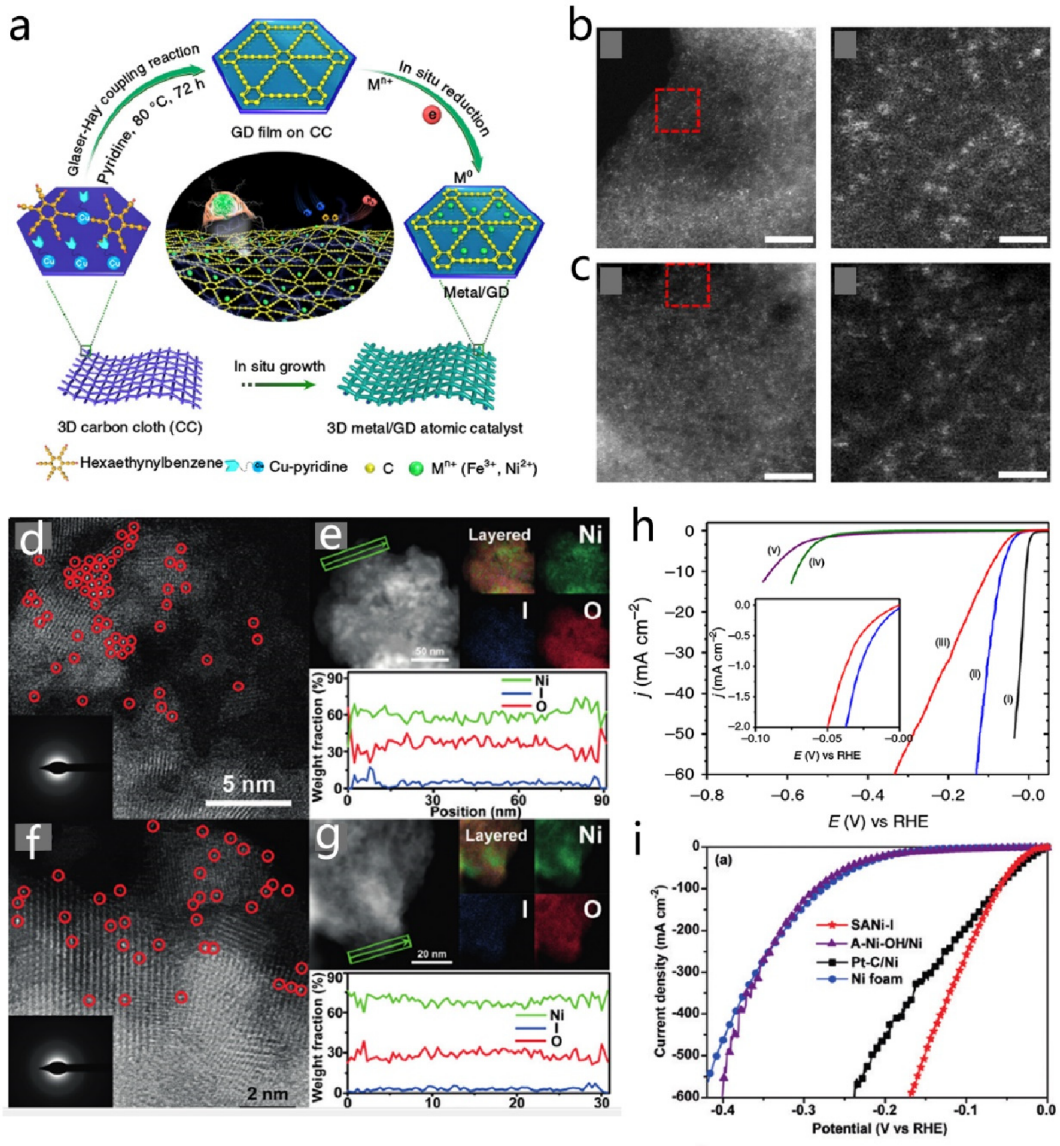
(a) Schematic diagram of the synthesis process of M/GD. (b, c) STEM images of the Ni/GD and Fe/GD. Reprinted with permission from ref (369). Copyright 2018 Springer Nature. (d, f) Enlarged STEM images of the SANi-I and SANi-I-96 samples. The I atoms are highlighted by red cycles. (e, g) Line scanning profile of SANi-I and SANi-I-96. Reprinted with permission from ref (370). Copyright 2019 Wiley-VCH. (h) The HER performance of the obtained Ni/GD and Fe/GD. Reprinted with permission from ref (369). Copyright 2018 Springer Nature. (i) The LSV plots of the SANi-I and SANi-I-96 catalysts. Reprinted with permission from ref (370). Copyright 2019 Wiley-VCH.

Another strategy to improve the performance of SACs consisted in adopting 3D carbon cloth (CC) to load SAs. CC could provide a 3D accessible structure to fully use the supported materials. Xue et al. applied a two-step strategy to prepare a multilevel structure (CC@graphdiyne@SAs) for HER.^369^ As illustrated by the protocols (Figure 43a), the CC@graphdiyne was synthesized through the Glaser–Hay cross-coupling reaction. Then, the loaded Ni^2+^ and Fe^3+^ species were reduced to zerovalent atoms by electrochemical reduction. The HAADF-STEM images (Figure 43b, c) showed the atomically dispersed Ni and Fe SAs. DFT calculations revealed that the Ni^0^ could stabilize the charge density distribution, thus inducing the fast charge exchanges of the H^+^ + e^–^ reaction and facilitating the HER. Apart from the isolated metal atoms doping the substrates, nonmetal SAs also could be loaded on the surface of the metal surface to change the electronic configuration for the catalysis. Based on this principle, Zhao et al. constructed single atom nickel iodide (SANi-I) electrocatalyst with unusual Ni-I species.^370^ STEM images in Figure 42d–g clearly showed the randomly dispersed iodine atoms on the Ni. By the analysis of XAS, the iodine atoms could bond with O to form I-O. Moreover, the *in situ* Raman spectra uncovered the presence of H_ads_ on iodine atoms, which suggested the presence of I–H_ads_ bonds. The synergistic effect between the I–O, I–H_ads_, and I–Ni bonds provided the attractive electronic environment to expedite the dissociative adsorption of water. The SANi-I complex demonstrated excellent HER activity that was directly comparable to that of commercial Pt/C (Figure 43h, i). Nonmetal SAs could also change or improve the electronic environment of metal active sites, making them more suitable for catalyzing water splitting and achieving excellent HER performance.

In this section various strategies to create SACs tailored for HER applications have been reviewed. Cobalt has been identified and studied as one of the highly active transition metal SACs for HER applications. It has been fabricated as a 2D porous Co-N-C complex supported on carbon fibers (CFs) through carbonization, achieving remarkable HER activity, close to that of commercial Pt/C. Another technique introduced heteroatom doping, forming a Co-N-C-S-O SACs electronic channel and stabilization environment with an excellent HER performance, especially at high current density. Understanding the crucial role of the metal site position and coordination, researchers designed CoMoS_*x*_ catalysts, highlighting the significance of the structure coordination in the catalytic activity. Enhancing the SACs’ catalytic activity involved doping nonmetal elements like Mn, reducing the Gibbs free energy for an improved HER activity. *In situ* fabrication methods such as the cold hydrogen plasma reduction were explored, and using 3D carbon cloth (CC) to support SACs, creating a multilevel structure (CC@graphdiyne@SAs). Additionally, the loading of nonmetal SAs onto the metal surfaces altered the electronic configurations, leading to an outstanding HER performance, comparable to that of commercial Pt/C. These strategies demonstrated significant progress in developing SACs for efficient and durable HER catalysis. However, exploring new support materials with an enhanced coordination capability for SACs and investigating the impact of the introduction of various transition metal atoms on the catalyst’s performance still require extensive research.

## 6 EARTH-ABUNDANT SACs IN OXYGEN EVOLUTION REACTION (OER)

Water splitting involves two half-reactions. As discussed in the previous section (Section 5), considerable research has been dedicated to SACs for hydrogen production, primarily focusing on the HER mechanism. However, the HER mechanism, although important, is not the primary bottleneck in water splitting devices. While higher HER activity leads to increased hydrogen production, the water oxidation process, specifically the OER, poses the rate-limiting challenge in water splitting systems. Similar to the ORR, OER involves a 4-electron process, resulting in sluggish kinetics compared to the HER.^371^ Therefore, developing advanced OER catalysts will effectively improve the efficiency of water splitting for H_2_ production. To date, the most widely used OER catalysts are IrO_2_ and RuO_2_, which are PGM-based catalysts and as such extremely costly and scarce, nullifying their usage on large-scale applications.^371^ For this reason, earth-abundant transition metal elements correspond to an attractive choice to effectively alleviate this issue. Moreover, as discussed in sections 2.2 and 4.4, the interest in bifunctional materials allowing efficient OER activity increased with the promising opportunities brought by metal-air batteries. As a result, earth-abundant transition metal OER-SACs connect greatly the necessity for cheap and available catalysts with the rising demand for superior performance catalysts focused on green source of energy. This section will briefly discuss the different reaction pathways for OER depending on the pH conditions, followed by insights on the research efforts done on the earth-abundant SAC synthesis applied to OER.

### 6.1 Reaction Pathways and Mechanisms of OER

Like HER, the OER process is greatly affected by the pH of the electrolyte and the mechanism exhibits differences as pH changes. The typical OER mechanisms are described as follows.^372^

In alkaline solution:
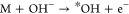
18
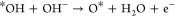
19
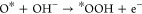
20
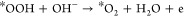
21
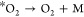
22
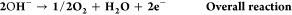
23

In acidic solution:
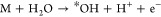
24
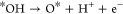
25
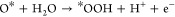
26
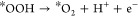
27
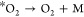
28
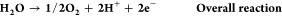
29

As represented in these eqs (eqs 18–29), the intermediates (*OH) are generated in the first step. In the acidic solution, the hydroxide source comes from H_2_O; thus in both cases, the *OH will adsorb on the active sites of the catalyst surface. Importantly, the step to form *OOH requires the highest energy for the O_2_ formation, which is considered the rate-determining step. Designing suitable catalysts to accelerate this rate-determining step will effectively promote the OER activity.

### 6.2 Fabrication of SACs for OER Applications

The major research efforts for OER focus on the reaction in alkaline conditions due to the usually lower overpotential for OER and higher catalyst stabilities in alkaline condition.^373^ For this reason, we focus on the recent advances in SACs in alkaline electrolyte. Extensive studies have been performed to synthesize highly active and cost-effective SACs for OER. For instance, Zhong and co-workers established a method to obtain Fe-N-C SACs by performing a facile Lewis acid (FeCl_3_) pretreatment and carbonization process on natural wood.^374^ The FeCl_3_ processing on the wood either produced a large amount of microchannels or introduced Fe-N species into the wood.

**Figure 44:**
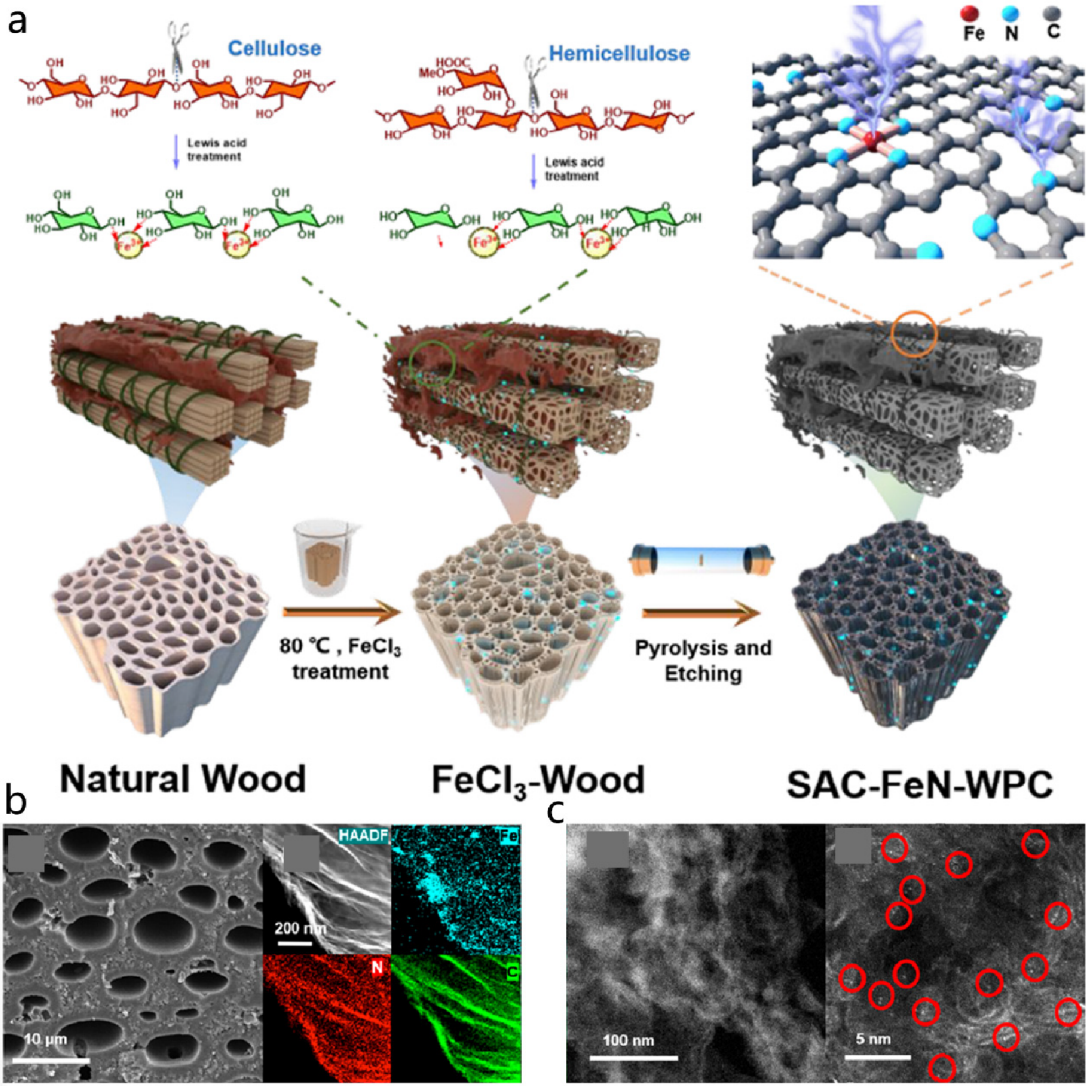
(a) Schematic diagram of the synthesis process for SAC-FeN-WPC. (b) SEM and STEM and EDS mapping images of the SAC-FeN-WPC material. (c) Enlarged STEM images of the SAC-FeN-WPC. Reprinted with permission from ref (374). Copyright 2021 American Chemical Society.

**Figure 45:**
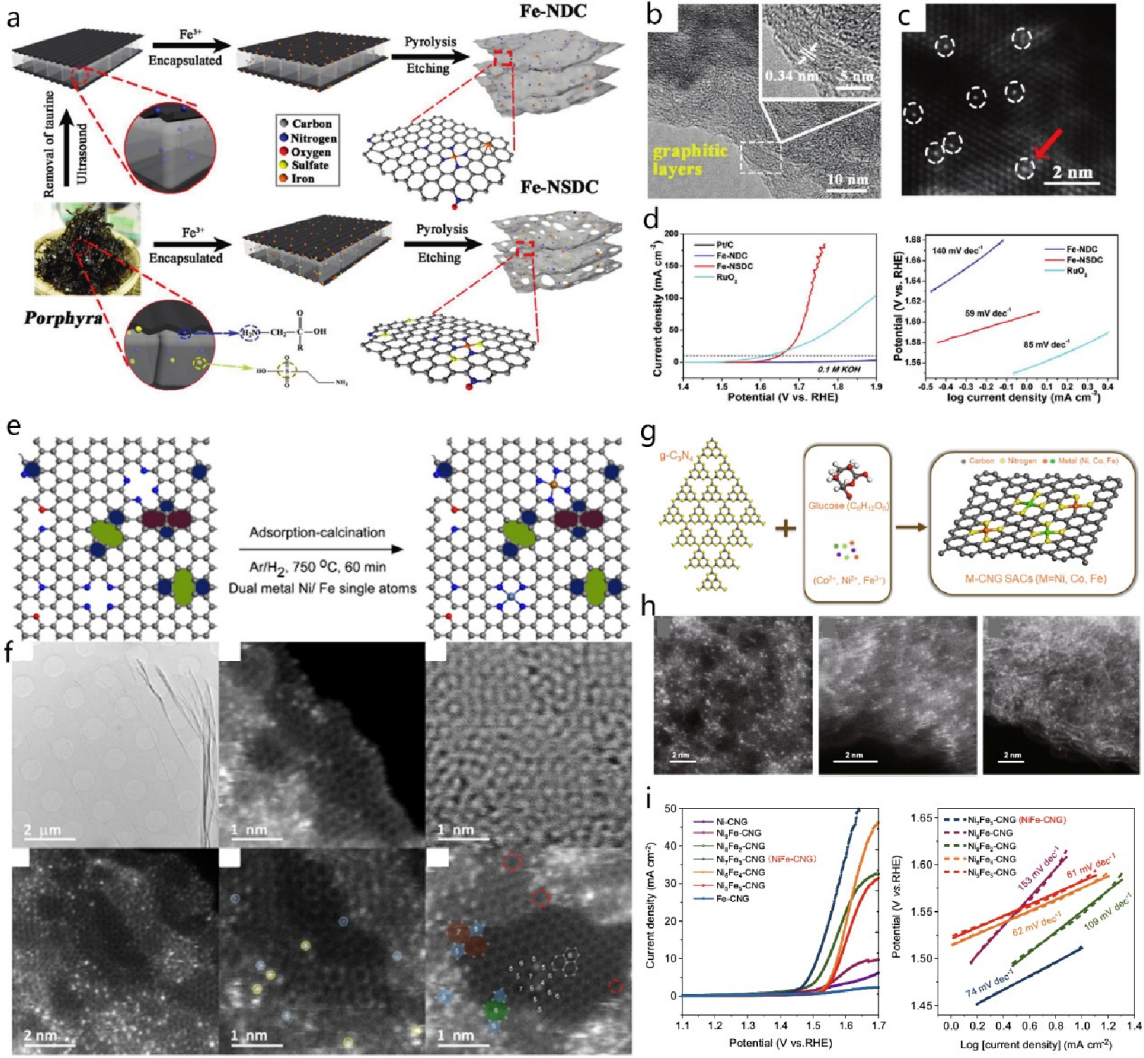
(a) Preparation process of Fe-NSDC. (b) HR-TEM and (c) STEM images of the Fe-NSDC. (d) LSV and Tafel curves of the Pt/C, Fe-NDC, Fe-NSDC, and RuO_2_. Reprinted with permission from ref (375). Copyright 2019 Wiley-VCH. (e) Synthesis schematic of the dual metal Ni/Fe SACs. (f) HR-TEM and the magnified STEM images of the Ni/Fe SACs. Reprinted with permission from ref (376). Copyright 2021 Elsevier. (g) Synthesis diagram of the M-CNG SACs (M = Ni, Co, Fe). (h) Typical STEM images of the Ni-CNG, Co-CNG, Fe-CNG SACs. (i) LSV and Tafel curves of dual Ni/Fe SACs. Reprinted with permission from ref (161). Copyright 2021 Springer Nature.

After the pyrolysis at high temperature under inactive gas, the Fe-N-C SACs were prepared (Figure 44a).^374^ Because the natural wood has a lot of impurities, the Fe-N-C SACs could only be obtained after strong acid washing of the carbide, named SAC-FeN-WPC. The SEM image (Figure 44b) shows the porous hierarchical structure of the SAC-FeN-WPC with micron-scale holes. The HAADF-STEM and the corresponding EDS mapping images (Figure 44b) display the uniform distribution of Fe, C, and N elements in the SAC-FeN-WPC. Enlarged STEM images (Figure 44c) reveal the randomly dispersed isolated Fe atoms. Such uniformly dispersive Fe SAs in SAC-Fe N-WPC effectively enhanced the OER performance with a low overpotential of 400 mV at 10 mA cm^–2^ that was comparable to that of RuO_2_ (340 m V at 10 m A cm^–2^). However, the activities of the Fe-N-C SACs still need to be further improved for practical applications. Doping other nonmetallic heteroatoms into Fe-N-C SACs could effectively regulate the local electronic environment around Fe sites, thus benefiting the OER catalysis. Zhang and co-workers developed an encapsulation-pyrolysis strategy to introduce S atoms into the Fe-N-C nanosheets.^375^ The dried porphyra was pulverized with FeCl_3_ adsorbing Fe^3+^ on the surface. The proteins and taurine in porphyra contained abundant proteins with amino and carboxyl groups, which could form the Fe^3+^-amino acid complexes. After annealing at 900 °C in Ar atmosphere, the porous S-doped Fe-N-C nanosheets were obtained (Fe-NSDC). Notably, only well-defined S-doped Fe-N-C nanosheets without porous structure could be synthesized after initial removal of taurine.^375^ The detailed preparation schematic is shown in Figure 45a. The HR-TEM image (Figure 45b) showed the 2D morphology of Fe-NSDC and the typical lattice spacing of graphitic (d = 0.34 nm). Atomic resolution STEM characterization (Figure 45c) results identified many isolated Fe atoms on the graphite layer. As depicted in Figure 45d, the LSV of Fe-NSDC SACs presented excellent OER activity with the overpotential of 410 mV at 10 mA cm^–2^ and the Tafel slope of 59 mV dec^–1^ in 0.1 M KOH, revealing the remarkable OER performance. The improved OER performance of Fe-N-C indicated that the S-doping displayed an effective pathway for highly active SACs development.

The synergistic effect of bimetallic catalysts could unleash the catalytic activity of each metal and superimpose the activity to a certain extent. Many studies have found that the synergistic effect still works even when the size of the bimetallic catalyst is reduced to the atomic level. Khan et al. reported a defect-mediated approach to prepare SACs based on the defect-rich carbon-supported graphene (*d*-CN).^308^ The as-prepared *d*-CN powder with acrylamide, NiCl_2_, and FeCl_2_ precursors was physically mixed to generate an inhomogeneous solution. Then, the material was frozen by liquid nitrogen and sintered at various temperatures (650 °C, 700 °C, and 750 °C) under Ar/H_2_ mixture gas to obtain Ni-DG SAC, Fe-DG SAC, and bimetallic Ni/Fe-DG SAC (Figure 45e). The TEM image (Figure 45f) shows that there are no obvious clusters or particles on the *d*-CN. The enlarged STEM images in Figure 45f exhibit a large number of scattered SAs on the *d*-CN. With the same principle, Wan et al. fabricated single/dual-atom Ni/Fe-based SACs with the g-C_3_N_4_ as the support.^161^ To ensure the appropriate amount of metal ions on the surface of g-C_3_N_4_, glucose was applied as the linker to coordinate with metal ions (Co^2+^, Ni^2+^, Fe^2+^) on the surface of g-C_3_N_4_ (Figure 45g). Figure 45h shows the STEM images of Ni-CNG, Co-CNG, and Fe-CNG, respectively. The Ni sites were favorable to facilitate the bridge Ni–O–Fe bonds in dual-site Ni/Fe-CNG SACs. The bridge Ni–O–Fe bonds could serve as spin channels for electron transfer, accelerating the OER activity with the smallest overpotential (270 mV, 10 mA cm^–2^) compared with the Ni, Fe-CNG (Figure 45i).

**Figure 46:**
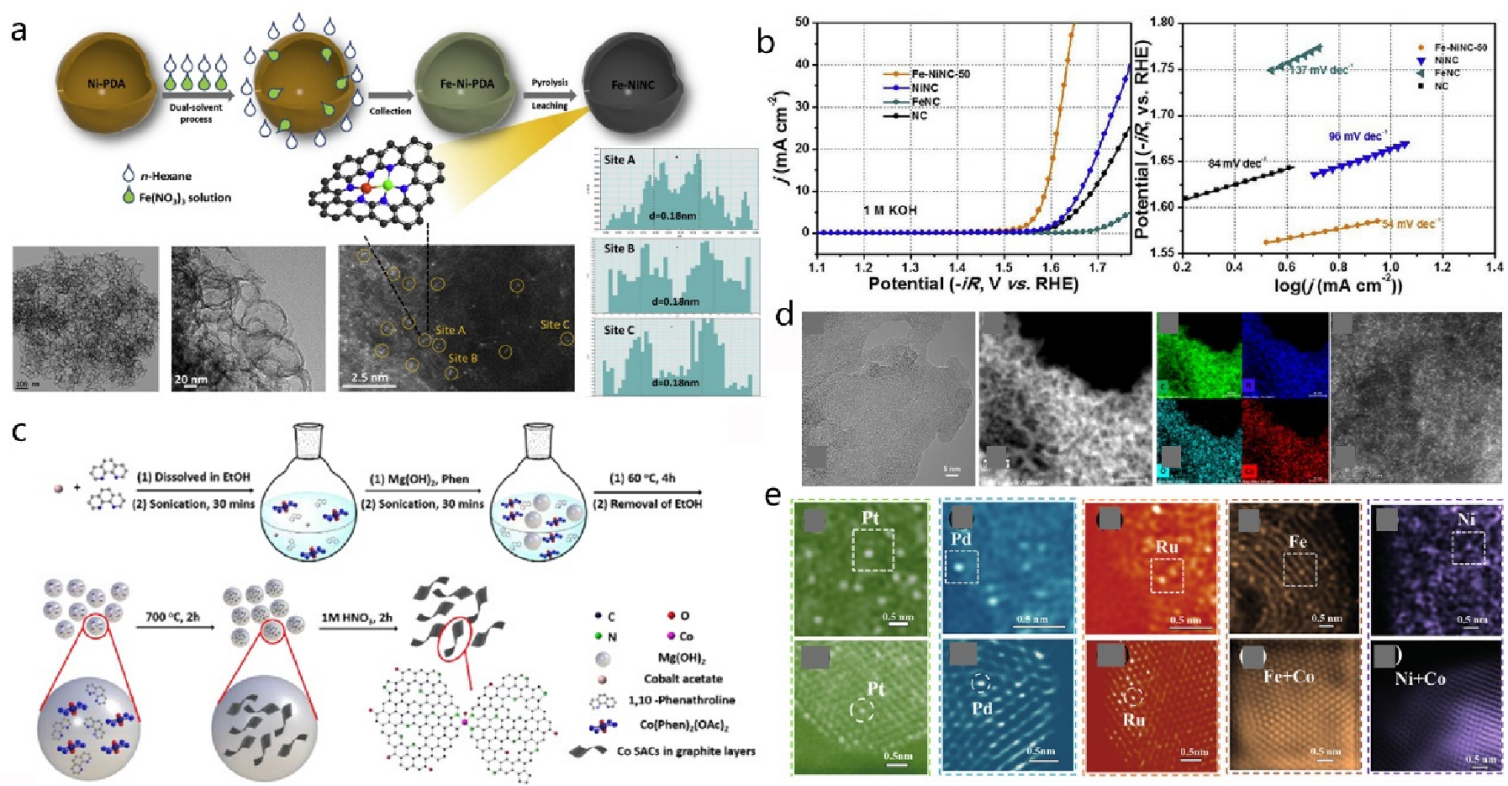
(a) Fabrication process of the Fe-Ni-N-C and the HR-TEM/STEM images and the EDS spectrum of the Fe-Ni-PDA. (b) The LSV and Tafel curves of Fe-N-C, Ni-N-C, and Fe-Ni-N-C. Reprinted with permission from ref (377). Copyright 2020 Elsevier. (c) Synthesis of Co-N-C SACs. (d) The HR-TEM, STEM images and the EDS mapping images of the Co-N-C SACs, together with the atomic resolution STEM image. Reprinted with permission from ref (378). Copyright 2019 American Chemical Society. (e) STEM images of various M@Co/NC types of SACs, M = Pt, Pd, Ru, Fe, Ni. Reprinted with permission from ref (379). Copyright 2019 Wiley-VCH.

Equivalently, Zhu et al. used porous CN spheres to embed the paired Ni-Fe single atoms into the CN spheres.^377^ The paired Ni-Fe SACs were successfully prepared by using Ni-PDA as the template, as illustrated in Figure 45a. The Ni-PDA was mixed with Fe(NO_3_)_3_ in *n*-hexane to form Fe-Ni–PDA. After the pyrolysis and leaching, the dual paired Fe-Ni SACs embedded in nitrogen-doped carbon were successfully synthesized. The HR-TEM and STEM images verified the existence of bimetallic single atoms (Figure 46a). Interestingly, the Fe-Ni-N-C delivered an overpotential of 340 mV, which was 90 mV and 120 mV lower than that of Ni-N-C or N-C (Figure 46b). Additionally, Fe-Ni-N-C presented the smallest Tafel slope (54 mV dec^–1^) among the Fe-N-C, Ni-N-C, and N-C catalysts (Figure 46b). Bai and co-workers also applied a polymer method with the coordination between the metal ions and organic molecule to enrich Co/Fe ions in the materials (Figure 46c).^378^ Based on the TEM and STEM images of the obtained Co-N-C SAC, there were no metal clusters or nanoparticles in CN. The EDS mapping images verified the C, N, O elements in the Co-N-C SAC, consistent with the enlarged STEM image (Figure 46d). Moreover, Lai and co-workers developed a general π-electron-assisted strategy to synthesize Ir, Pt, Ru, Pd, Fe, and Ni based SACs.^379^ By adding the metal precursors (M = Ni/Fe/Ru/Pt/Pd/Ir ions) into the ZIF-67 dispersion, the metal ions would strongly coordinate with imidazole to form the intermediates. After carbonization, various M@Co/NC types of SACs could be obtained showing interesting performance for HER/OER applications. The STEM images in Figure 46e presented the FFTI-STEM results of M@Co/NC SACs materials.

Water splitting in acidic environments provides several advantages, such as high ionic conductivity, a decrease inside reactions, plentiful protons, lower ohmic losses, and opportunities for compact system design. Yet, these acidic conditions greatly restrict suitable electrocatalyst choices, especially for the OER, which typically requires detrimental high overpotentials. Creating high-performance OER electrocatalysts for acidic media, with the right electronic, surface, and structural properties, is a substantial challenge. Consequently, research and development in this area lag behind that in the alkaline conditions. The current focus is on developing OER catalysts that are both highly effective and acid-resistant, which is driven by the urgent need for practical applications. Presently, the most efficient electrocatalysts for the OER in acidic conditions are still RuO_2_ and IrO_2_. Alternatively, nickel, cobalt, and iron are more affordable and abundant but tend to lose their OER activity quickly in acidic media. Those SACs that have been successfully developed often display multifunctional electrocatalytic abilities across various pH levels, not just in the OER but also in the ORR and the HER. Doan and colleagues have developed a novel bifunctional electrocatalyst based on single-atom cobalt-decorated MoS_2_ nanosheets, which are supported on three-dimensional titanium nitride (TiN) nanorod arrays, referred to as CoSAs-MoS_2_/TiN NRs.^380^ This electrocatalyst demonstrated remarkable activity for overall water splitting in electrolytes effective across all pH levels. When used as an anode, the CoSAs-MoS_2_/TiN NRs achieved low OER overpotentials of 454.9, 340.6, and 508.0 mV at a current density of 10 mA cm^–2^ in acidic, alkaline, and neutral environments, respectively. Additionally, this same electrocatalyst, serving as the cathode, contributed to a highly efficient and robust full electrolyzer system. The combined anodic and cathodic CoSAs-MoS_2_/TiN NRs electrodes delivered an exceptional performance in overall water splitting, bearing also sufficient stability and durability under varying pH conditions. Wang and colleagues^381^ introduced another noteworthy example in this field. They developed an overall water oxidation catalyst composed of metallic Co_9_S_8_ decorated with single-atomic Mo (0.99 wt %), labeled as Mo-Co_9_S_8_@C. Similarly to the previous case, this electrocatalyst exhibited high water oxidation activity in acidic, alkaline, and neutral solutions, as indicated by onset potentials of 200, 90, and 290 mV, respectively. Additionally, it showed an exceptional HER performance across a broad pH range. Remarkably, this catalyst surpassed the efficiency of benchmark noble metal Pt/IrO_2_-based catalysts for overall water splitting, requiring only 1.68 V in acidic and 1.56 V in alkaline media. Its exceptional stability was demonstrated through 24 h of continuous operation in 0.5 M H_2_SO_4_ and 72 h in 1.0 M KOH, maintaining the performance at a consistent current density of 10 mA cm^–2^. The DFT simulations further revealed that the synergistic interaction between atomically dispersed Mo- and Co-containing substrates significantly modifies the binding energies of intermediate species, thereby reducing the overpotentials for water splitting. Further studies have been mostly focused on the theoretical prediction of the most suitable noble-metal-free electrocatalysts for the OER working in acidic media. For example, Deng et al. employed DFT calculations to systematically investigate the bifunctional electrocatalytic potential of 2D transition metal-based tetracyanoquinodimethane (TM-TCNQ), with TMs including Cr, Cu, Ru to Ag, Pt, and Ir) monolayers. These layers featured single transition metal atom catalysts distributed at relatively high densities.^382^ Focusing on the OER in acidic electrolytes, the team found out that Ni-TCNQs exhibited the lowest calculated overpotential of 0.46 V among the compared electrocatalysts. In contrast, Fe-TCNQ showed a remarkably lower overpotential of 0.33 V for the ORR, suggesting higher predicted activity than that of Pt (0.48 V). The study also revealed that the introduction of axial ligands and the application of external strain could further improve the activity of Mn-, Fe-, and Ni-TCNQs for either the ORR or the OER. Specifically, Fe-TCNQ-Cl (with overpotentials of 0.27/0.55 V) and Fe-TCNQ-CO (0.67/0.43 V) emerged as promising high-performance bifunctional OER/ORR catalysts. Their calculated limiting overpotentials are comparable to those of leading commercial electrocatalysts: Pt for the ORR (0.48 V) and RuO_2_ for the OER (0.42 V). A promising 2D bifunctional electrocatalyst based on an organometallic framework with a pyridinic-type FeN_4_ ligation environment, specifically the (phen_2_N_2_)FeCl monolayer, was identified as another SACs-based bifunctional electrocatalyst for the OER/ORR in acidic media, potentially surpassing the benchmark Pt/IrO_2_ catalysts.^383^ Using comprehensive first-principles calculations and microkinetic modeling, it was demonstrated that the (phen_2_N_2_)FeCl monolayer offers bifunctional activity surpassing that of the benchmark Pt/IrO_2_ catalysts. Similar to the previously mentioned study, Liu and colleagues expanded their research to include a series of (phen_2_N_2_)MnCl monolayers (M = Mn, Co, Ni), uncovering that in particular (phen_2_N_2_)MnCl could potentially offer exceptional OER/ORR electrocatalytic activity. The study further elucidates that this enhanced activity is linked to specific coordination environments, such as the pyridinic-type MN_4_ moieties.

Recent progress in SACs for the OER in alkaline conditions has been widely studied due to their lower overpotential and enhanced stability. Innovative methods, such as Lewis acid pretreatment and carbonization of natural wood, have been employed to produce Fe-N-C SACs with isolated Fe atoms uniformly dispersed on a porous hierarchical structure. Introducing sulfur doping into Fe-N-C nanosheets through an encapsulation-pyrolysis strategy has significantly boosted the OER activity. At the atomic level, bimetallic catalysts have demonstrated synergistic effects, as seen in Ni-Fe-based SACs embedded in carbon spheres. Alternative strategies involving the coordination of metal ions with organic molecules have resulted in SACs promising for diverse electrochemical applications including the OER/HER. The OER mechanisms of non-noble metal SACs have also been broadly studied. For instance, both dual-site and single-site mechanisms have been proposed for the high OER activity of NiN_4_C_4_ moieties. Furthermore, a certain spin configuration in Ni-O-Fe bonds, with high-valent Fe^4+^ species exhibiting an optimal spin channel, has been identified as a crucial factor influencing the OER activity. Additionally, spin density has emerged as a novel property influencing the SAC activity. For example, adjusting the spin density of Co single atoms on a TaS_2_ support optimizes their interaction with oxygenated intermediates. The exceptional performance and potential cost-effectiveness of specific SACs make them promising for further exploration in electrocatalysis. The OER exhibits distinct features in acidic conditions, particularly regarding the reaction mechanism, catalyst requirements, and efficiency. In acidic environments, the OER generates oxygen primarily from water molecules. The reaction typically involves the deprotonation of water, leading to the formation of oxygen through intermediate OH species. This contrasts with alkaline conditions, where the OER predominantly involves hydroxide ions (OH^–^). The OER in acidic conditions often requires a higher overpotential, leading to generally less efficiency than in alkaline environments. Nevertheless, water splitting in acidic environments carries several advantages, such as increased ionic conductivity, fewer secondary reactions, a rich supply of protons, lower ohmic losses, and opportunities for more compact system designs. Particularly noteworthy are SACs like iron, cobalt, nickel, manganese, and molybdenum, which are coordinated with nitrogen in a carbon matrix (e.g., Fe-N-C). These SACs have demonstrated promising activity and stability for the OER under acidic conditions. Their unique electronic structure not only facilitates the OER but can also provide other electrocatalytic processes like the ORR and the HER across various pH levels. This versatility enables them to function as bifunctional electrocatalysts, adding to their application in diverse electrochemical applications.

## 7 EARTH-ABUNDANT SACs IN NITROGEN REDUCTION REACTION (NRR)

Ammonia (NH_3_) is an important chemical raw material with broad applications in many industrial fields. The traditional Haber–Bosch method is currently used to synthesize ammonia in industry. However, harsh conditions, such as high temperature (300–550 °C), high pressure (200–350 atm), serious environmental pollution, and high energy consumption, do not adhere to the sustainable development of society.^384,385^ Therefore, it is imperative to develop a green technology to replace traditional unsustainable production. During the past decades, many alternative methods have been continuously developed, including the photo-/electrocatalysis, enzymatic catalysis, and the optimized chemical looping processes.^386^ Among these methods, photo-/electrocatalytic synthesis of ammonia at ambient temperature proved to be a promising technical route, which directly deployed water as a proton source with a mild operating condition. Especially this method is among the cleanest when the power source comes from solar energy, wind energy, or hydropower generation. However, several significant challenges must be addressed to achieve practical applications on a large scale.^387−389^ Among them, improving the efficiency and reducing the overpotential necessary for the NRR are critical challenges because NRR typically requires high overpotential and suffers from low reaction efficiency. Other challenges entail designing or discovering new catalysts enhancing the NRR kinetics - the NRR involves multiple proton-coupled electron transfer steps, making it kinetically challenging. As such, catalysts need to be designed or discovered to accelerate these steps, enhancing the overall reaction rate. As another example, achieving high selectivity for ammonia formation over other nitrogen reduction products, especially amidst highly competitive hydrogen evolution reactions, is a significant challenge as well. Catalysts must be precisely tuned to favor the desired product, maximizing ammonia yield while minimizing the formation of unwanted byproducts. The list goes on and includes catalyst stability - catalysts that show promising activity for the NRR suffer from poor stability over extended periods of operation. Further, cost and availability of catalyst materials are vital, as some efficient catalysts for the NRR contain precious or rare metals, making them expensive and less sustainable for large-scale applications. Developing catalysts based on earth-abundant and low-cost materials is crucial for the economic viability of the process. Another challenge is the electrode design - designing efficient electrodes that provide high surface area, good electrical conductivity, and effective mass transport of reactants and products is essential. Another example involves the reaction mechanisms - an understanding of the complex reaction mechanisms can guide the rational design of catalysts and reaction conditions for improved efficiency and selectivity. Last but not least is the integration with renewable energy sources - to make the process truly sustainable, integrating the electrochemical NRR with renewable energy sources (such as solar or wind power) is crucial.

### 7.1 Reaction Pathways and Mechanisms of NRR toward Ammonia

**Figure 47:**
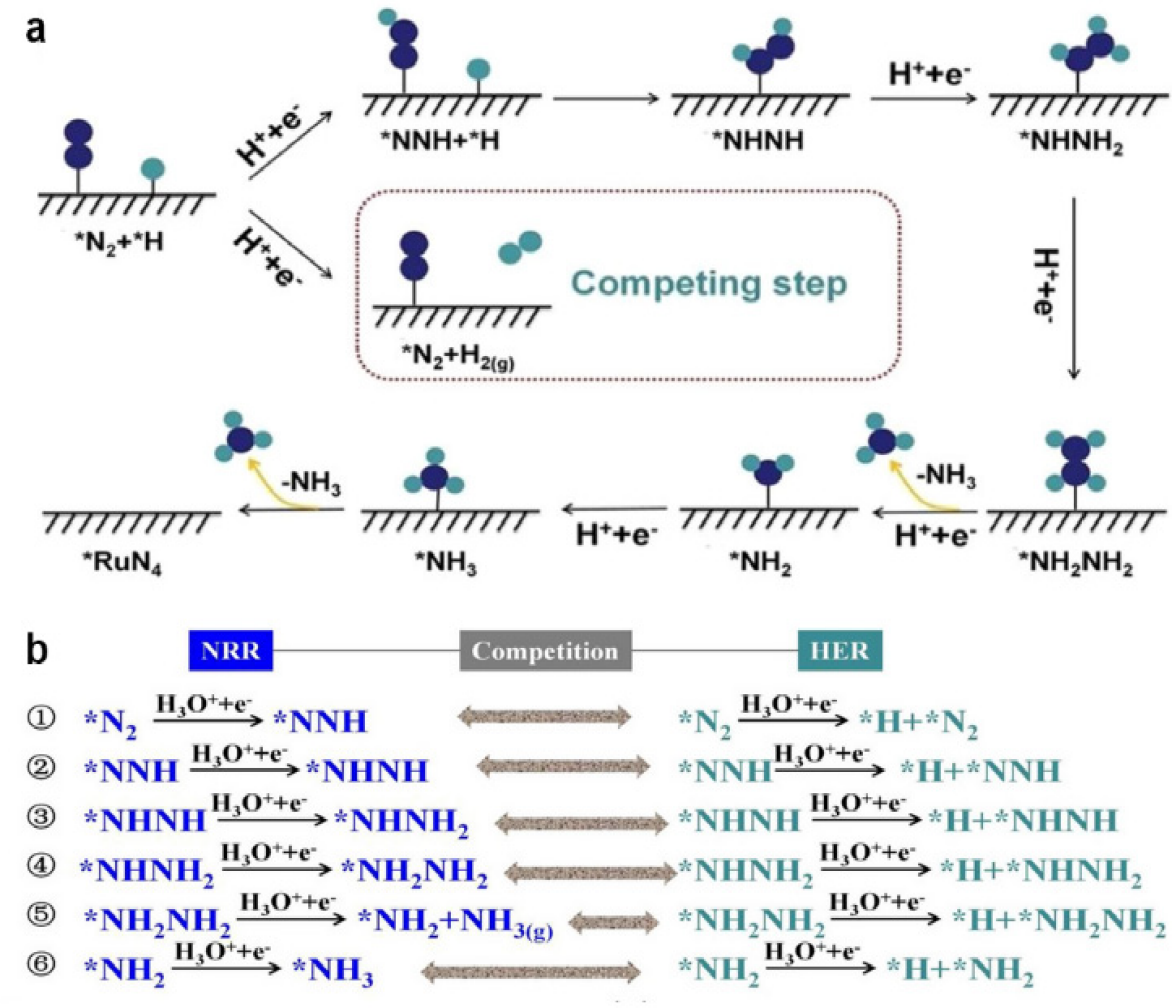
(a) Schematic of the different steps involving the NRR and the competing step for HER on a model catalyst - Ru-N_4_. (b) The competing PCET steps in NRR and HER. Adapted with permission from ref (396). Copyright 2022 American Chemical Society.

The conversion from nitrogen to ammonia faces a great challenge because of the strong N≡N triple bond (941 kJ mol^–1^) and the large first bond cleavage energy (410 kJ mol^–1^).^384,385^ Moreover, the NRR is in direct competition with the HER due to the Volmer step (see Section 5.1). Indeed, the formation of M-H* intermediates is necessary for the NRR. Theoretical approaches, including DFT and machine learning techniques, have played a pivotal role in elucidating the underlying mechanisms of the SACs in the field of NRR. By combining these theoretical approaches, substantial progress in understanding the fundamental principles governing the activity and stability of earth-abundant single-atom catalysts has been made. These insights are crucial for the rational design of efficient catalysts, significantly contributing to the development of sustainable and energy-efficient nitrogen reduction technologies. As part of the DFT studies, particularly the electronic structure and adsorption energies of nitrogen-containing intermediates on the SACs’ active sites have been explored. These calculations provide crucial insights, e.g. into the binding strength, reaction pathways, and the role of the solvent used, generally shedding light on the catalyst’s efficiency.^390−393^ Machine learning algorithms, on the other hand, have been employed to predict the catalytic activity of various SACs based on their structural features, facilitating the identification of promising candidates from a vast pool of possibilities.^394,395^ Through the DFT calculations, Wu et al. recently proposed different intermediates and reaction pathways on a Ru-N4 catalyst as a model catalyst due to its promising results for NRR (Figure 47a).^396^ Notably, every step including the M-H* intermediate seems more favorable through the Heyrovsky step than through the NRR intermediates formation, while the presence of adsorbed *N_2_ and NRR species can significantly suppress the HER competition (Figure 47b).

Ultimately, in the case of the photo-/electrocatalytic NRR, the catalysts are critical for accelerating the reaction and the selectivity by favoring the *NNH intermediate and hindering the HER. Despite these difficulties regarding the NRR, the investigation of efficient NRR catalysts attracted immense attention in recent years wherein the elemental metals, oxides, nitrides, carbides, MXenes, and SACs have been studied because of the huge impact that the NRR-clean processes could have on society. Advantageously, SACs can effectively bond with nitrogen due to their special electronic coordination environment, which is desirable for specialized studies.^384^

### 7.2 SACs Design for NRR Applications via DFT Calculations

**Figure 48:**
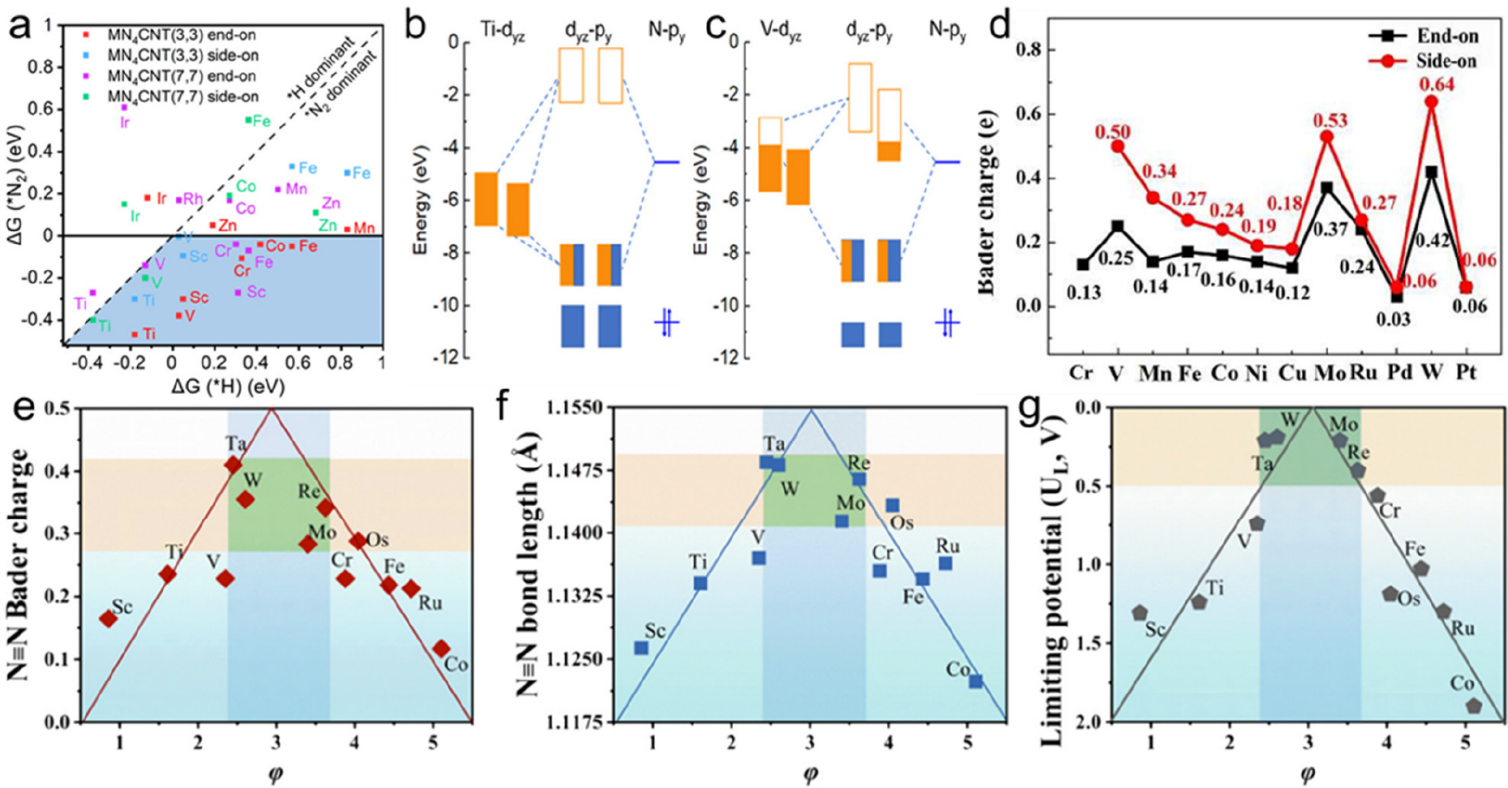
(a) Calculated Δ*G*(*H) and Δ*G*(*N_2_) under standard conditions (pH = 0, p(H_2_) = 1 bar, U = 0 V vs NHE) with respect to different studied TMs atoms in N_4_CNT. The dashed line indicates Δ*G*(*H) = Δ*G*(*N_2_). The blue shadow represents the N_2_ stable adsorption region, (b, c) Band structure of M d_*yz*_ orbital, N-p_*y*_ orbital of the NH_2_NH_2_ molecule, and their interaction of the *NH_2_NH_2_ adsorption for (b) TiN_4_CNT(5,5) and (c) VN_4_CNT(5,5). Reprinted with permission from ref (397). Copyright 2022 American Chemical Society. (d) The Bader charge of the N_2_ adsorbed on TM-*g*-C_3_N_5_. Reprinted with permission from ref (398). Copyright 2022 Elsevier. (e–g) Volcano plots for descriptor φ vs (e) N_2_ Bader charge, (f) N≡N band length, and (g) the computed NRR limiting potential (*U*_L_). Reprinted with permission from ref (399). Copyright 2022 American Chemical Society.

The DFT calculations have been widely employed to predict numerous catalytic systems, involving various supports with embedded noncritical elements in the form of SAs, aiming to enhance the NRR reaction. A few examples will be provided in this section. Guo et al. used first-principles DFT calculations to systematically assess the synergetic effect between the substrate geometric/electronic structures and the catalytic centers on the NRR. The authors applied several types of supports including carbon nanotubes with different chirality, defects, and chemical functionalization to support 15 transition metal atoms. Three SACs, TiN_4_CNT(3,3), TiN_4_CNT(5,5), and VN_4_CNT(3,3), simultaneously possess high NRR selectivities with respect to the HER and low overpotentials of 0.35, 0.35, and 0.37 V, respectively. Figure 48a illustrates the changes in the adsorption Gibbs free energy (Δ*G*) for the N_2_ molecules on single-atom catalysts (SACs) positioned in the *N_2_ domination region (Δ*G*(*N_2_) < Δ*G*(*H)), demonstrating the enhanced NRR selectivity. To ensure a stable N_2_ adsorption (Δ*G*(*N_2_) < 0), the focus should be directed specifically toward SACs within the shaded blue region. The electronic structure analysis elucidated that larger metal atoms anchored on CNTs with higher curvature and doped with N atoms facilitated the rupture of the N–N bond in *NH_2_NH_2_ to lower the overpotentials.^397^ In more detail, the authors elucidated the interaction between M-dyz and N-py of *NH_2_NH_2_ by employing band diagrams, as depicted in Figure 48b, c. These diagrams illustrate the rupture and integrity of the N–N bond. The interaction between M-dyz and N-py is responsible for the formation of bonding and antibonding π orbitals between M and N in *NH_2_NH_2_. The “ruptured and intact” cases exhibit larger and smaller energy differences, respectively, reflecting varying interaction strengths between M-dyz and N-py. This interaction induces the occupation of the antibonding orbital, leading to the weakening of the M–N bond and elongation of the bond length in the “intact” case (Figure 48b). Consequently, it further hinders the electron transfer from dz2 to the pz antibonding orbital. Conversely, in the scenario where *NH_2_–NH_2_ is ruptured (Figure 48d), the dyz-py antibonding orbital remains unoccupied, predicting a stronger and shorter M–NH_2_NH_2_ bond. The research conducted by Quan et al. extensively explored the electrocatalytic NRR comparing various transition metal (TM - V, Cr, Mn, Fe, Co, Ni, Cu, Mo, Ru, Pd, W, Pt) SACs embedded within g-C_3_N_5_ nanosheets.^398^ The study systematically investigated the activity, selectivity, and stability of these catalysts. Notably, V-*g*-C_3_N_5_ exhibited remarkable NRR activity with an onset potential of 0.30 V and excellent selectivity. Through analysis of partial density of states (PDOS) results, the study unveiled a strong hybridization between N_2_ and the d orbital of V. This interaction allowed the V atom, serving as an active site, to efficiently transfer electrons to N_2_. The Bader charge analysis reveals that, in the end-on configuration, N_2_ receives 0.25 |e| and 0.37 |e| from V and Mo, and, in the side-on configuration, it receives 0.50 |e| and 0.53 |e| from V and Mo, respectively. The V and Mo atoms, serving as active sites, demonstrated a flexible modulation of electrons, potentially enabling the precise transfer of the right number of electrons from V and Mo atoms to N_2_, compared to the rest of the tested TM SACs. This electron transfer is expected to facilitate subsequent reactions. Moreover, the study demonstrated a linear correlation between the NRR and the hydrogen evolution reaction (HER) activity (represented by Δ*G*(*NNH) and Δ*G*(*H), respectively) and the d-band center (DF) for 12 different TM-*g*-C_3_N_5_ configurations. However, V-*g*-C_3_N_5_ followed the first linear relation (Δ*G*(*NNH) vs DF) accurately but deviated from the second linear relationship (Δ*G*(*H) vs DF). This indicated that V-*g*-C_3_N_5_ possessed an optimal DF for catalyzing the NRR but an inappropriate DF for the HER, making it a promising material for NRR applications. Two-dimensional COFs represent another promising substrate for hosting SACs in the context of the NRR. These frameworks offer numerous stable hollow sites where various TMs can be securely anchored as single atoms, potentially overcoming the stability challenges. Wang et al. presented rather extensive first-principles calculations using DFT to explore 26 different TM atoms embedded in a 2D COF structure, known as dioxin-linked metallophthalocyanine (TMPc-TFPN), as platforms for an ammonia synthesis under ambient conditions. The NRR activity on TMPc-TFPNs was elucidated through the analysis of parameters such as N≡N bond length, Bader charge, ΔG*N_2_, and integrated crystal orbital Hamilton population (ICOHP). Furthermore, to understand the underlying mechanisms, the researchers developed multiple-level descriptors. Among these, a straightforward descriptor, φ, based on the electronegativity and the number of d electrons of TM atoms, clearly illustrated a volcano plot trend, indicating the relationship between the limiting potential in the NRR and these descriptors. In conclusion, by utilizing this comprehensive five-step screening strategy, wolfram-based Pc-TFPN (WPc-TFPN) emerged as the most promising SAC for the NRR. It exhibited a low limiting potential of −0.19 V and demonstrated high NRR selectivity over the competing and undesirable HER.^399^

### 7.3 Fabrication of SACs for NRR Applications

As demonstrated in previous sections, extensive DFT-based studies have been dedicated to describing the electrochemical NRR catalyzed by diverse single-atom metals under ambient conditions. However, experimental studies in this field are still relatively scarce. The key challenge in electrocatalytic NRR remains due to its low overall productivity, marked by low ammonia yield rates and poor FE. Despite these challenges, several promising examples addressing these practical issues have recently been presented.

**Figure 49:**
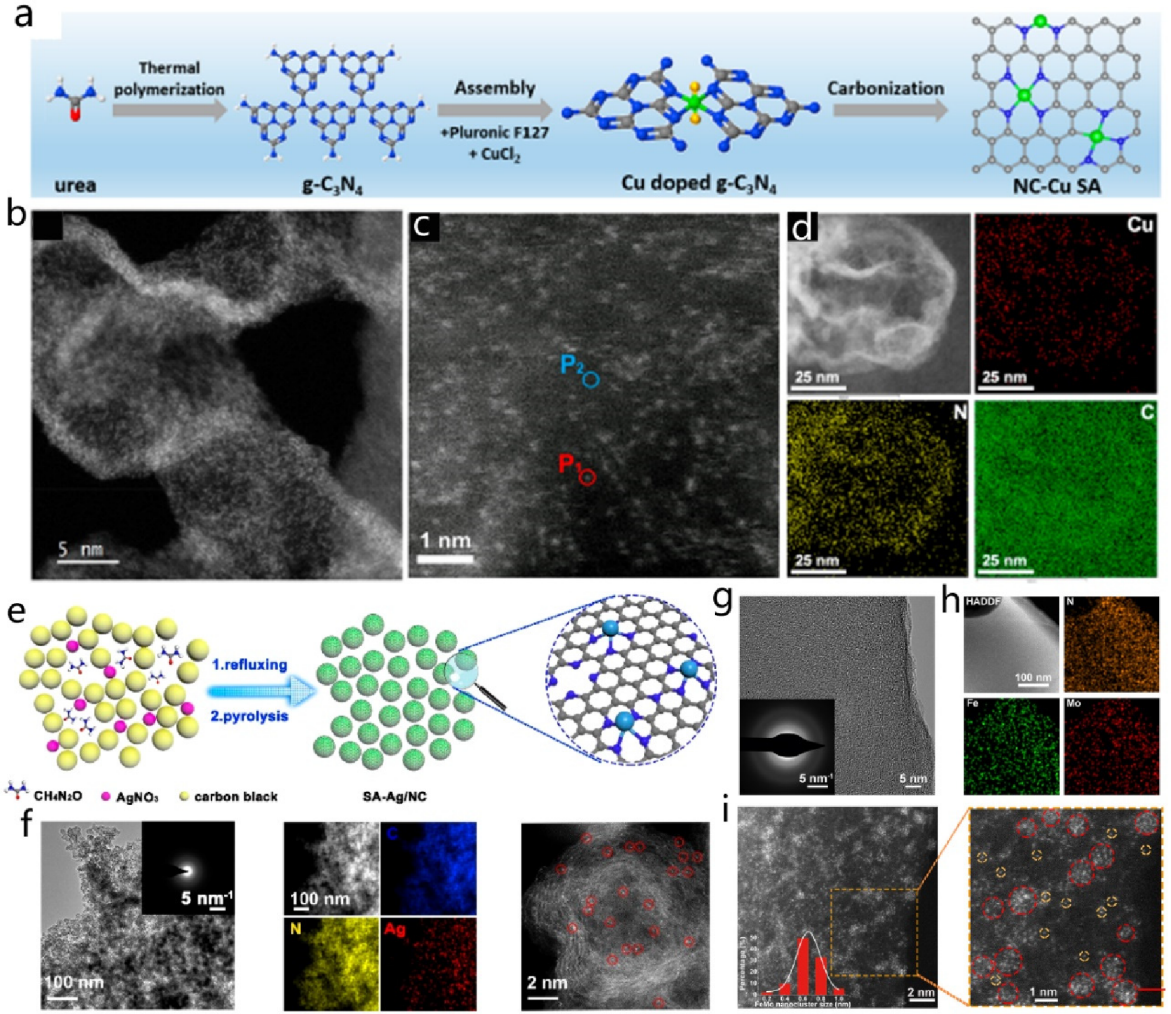
(a) Synthesis diagram of NC-Cu SA. (b, c) Magnified STEM images of NC-Cu SA. (d) Typical EDS mapping results of NC-Cu SA. Reprinted with permission from ref (400). Copyright 2019 American Chemical Society. (e) Preparation of SA-Ag/NC. (f) The HRTEM image, EDS mapping, and enlarged STEM images of the SA-Ag/NC. Reprinted with permission from ref (401). Copyright 2020 American Chemical Society. (g) TEM image of FeMo/NC. (h) STEM and the corresponding EDS mapping images of FeMo/NC. (i) Magnified STEM image to atomic level for FeMo/NC. Reprinted with permission from ref (402). Copyright 2020 Elsevier.

Zang and co-workers combined the experiments and simulation to discover that the Cu SAs immobilized into porous CN materials could provide highly efficient NRR property.^400^ A special preparation process (Figure 49a) provided a high level of porosity for the CN-Cu SA. As shown in Figure 49b, c, the enlarged STEM images of the catalysts present the porous structure of the CN substrate. The EDS mapping proved the uniform distribution of Cu, N, and C (Figure 49d). Cu SAC showed an excellent NH_3_ yield rate of ∼53.3 μg_NH3_ h^–1^ mg_cat_^–1^ in 0.1 M KOH and ∼49.3 μg_NH3_ h^–1^ mg_cat_^–1^ in 0.1 M HCl, as well as an FE of 13.8% under 0.1 M KOH and 11.7% under 0.1 M HCl, revealing the pH-universal. Apart from Cu catalysts, Ag-based SACs also had good potential for NRR application. The admolecule-targeting strategy was developed to anchor Ag monoatoms in carbon black.^401^ CH_4_N_2_O agent was used as the binder to bond Ag ions. After pyrolysis (Figure 49e), the well dispersed SA-Ag/NC powder was obtained. The HRTEM image in Figure 49f showed no particles on carbon black, and the EDS mapping results displayed that there were three elements of silver, carbon, and nitrogen. The enlarged STEM images of the SA-Ag/NC revealed the existence of isolated Ag atoms. Ag-N_4_ coordination sites in SA-Ag/NC played a key role for the superior NRR performance with the high NH_3_ yield rate of 270.9 μg_NH3_ h^–1^ mg_cat_^–1^ and FE of 21.9%.^401^ Additionally, Liu and co-workers prepared the FeMo sub-nanoclusters/single atoms on the CN material for NRR.^402^ The TEM and EDS mapping result (Figure 49g, h) showed that there were FeMo atoms dispersed on the CN material, while the magnified STEM images exhibited the highly dispersed metal atoms as highlighted by the red cycles in Figure 49i. FeMo/NC achieved the FE of 11.8 ± 0.8% at −0.25 V and NH_3_ yield rate of 26.5 ± 0.8 μg_NH3_ h^–1^ mg_cat_^–1^ at −0.3 V in neutral electrolyte.

**Figure 50:**
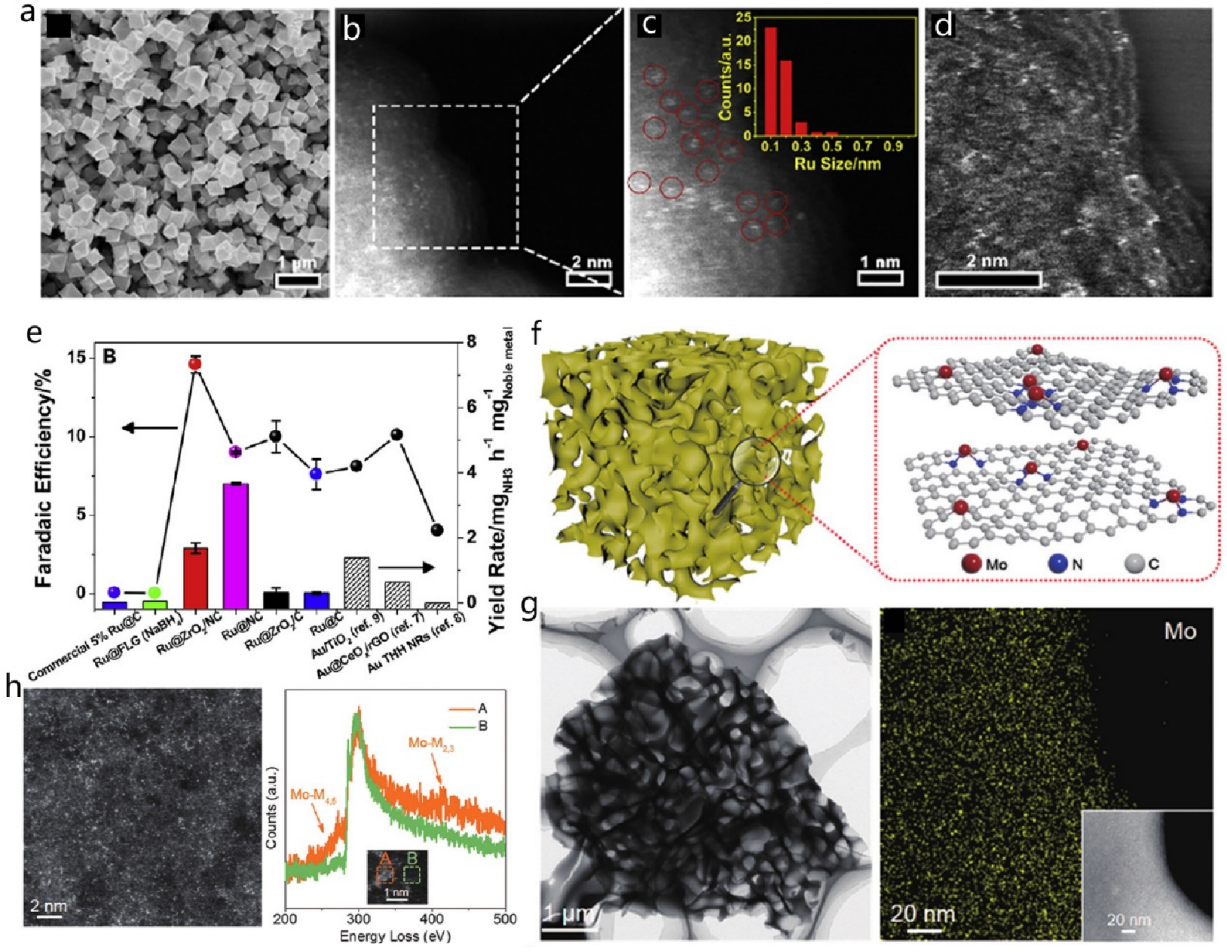
(a) SEM and (b, c, d) Gradually enlarged STEM images of the Ru@ZrO_2_/NC complex. (e) The FE and yield rate of NH_3_ over the Ru@ZrO_2_/NC, Ru@C, Ru@CN, and reported Au-based catalysts. Reprinted with permission from ref (403). Copyright 2019 Elsevier. (f) Model diagram of Mo-N-C SACs. (g) TEM and EDS mapping of the Mo-N-C SACs. (h) High resolution STEM image and the EELS spectra of the Mo-N-C SACs. Reprinted with permission from ref (404). Copyright 2019 Wiley-VCH.

MOF-based materials are the ideal structure for the preparation of SACs, due to the unique metal skeleton structure. Tao et al. used the UiO-66 MOF as the supporter to confine the Ru ions to generate Ru@ZrO_2_/NC complex after pyrolysis.^403^ The SEM image (Figure 50a) shows that the Ru@ZrO_2_/NC particles had a uniformly distributed size about submicron level. The gradually enlarged STEM images in Figures 50b–d present well-dispersed Ru single atoms in Ru@ZrO_2_/NC. Interestingly, the FE of Ru@ZrO_2_/NC at −0.21 V was about 15%, higher than those of reported Au-based SACs (Figure 50e), such as Au@CeO_*x*_/rGO (10.1%), Au/TiO_2_ (8.1%), and Au nanorods (4.0%), revealing the great potential for the MOF-based strategy. Mo-N-C SACs were designed by anchoring Mo atoms into nitrogen-doped porous 3D carbon frameworks^404^ wherein the 3D porous structure evolved from the decomposition of hydroxylamine hydrochloride precursor as illustrated by Figure 50f. This porous channel structure was 3D accessible based on the TEM image (Figure 50g), which could effectively ensure the sufficient exposure of Mo-N active sites for NRR. The Mo element evenly dispersed on the 3D porous CN, as verified by EDS mapping. The STEM and EELS results (Figure 50h) of the Mo-N-C SACs illustrated the massively dispersed Mo-N. As predicted, the Mo SAs on the highly porous carbon framework gave a high NH_3_ yield rate (34.0 ± 3.6 μg_NH3_ h^–1^ mg_cat_^–1^) and FE (14.6 ± 1.6%) in 0.1 M KOH.

Among Earth-abundant SACs, iron (Fe) stands out as one of the most commonly utilized elements for catalyzing the electrochemical NRR relatively selectively (particularly with respect to HER) and efficiently.

**Figure 51:**
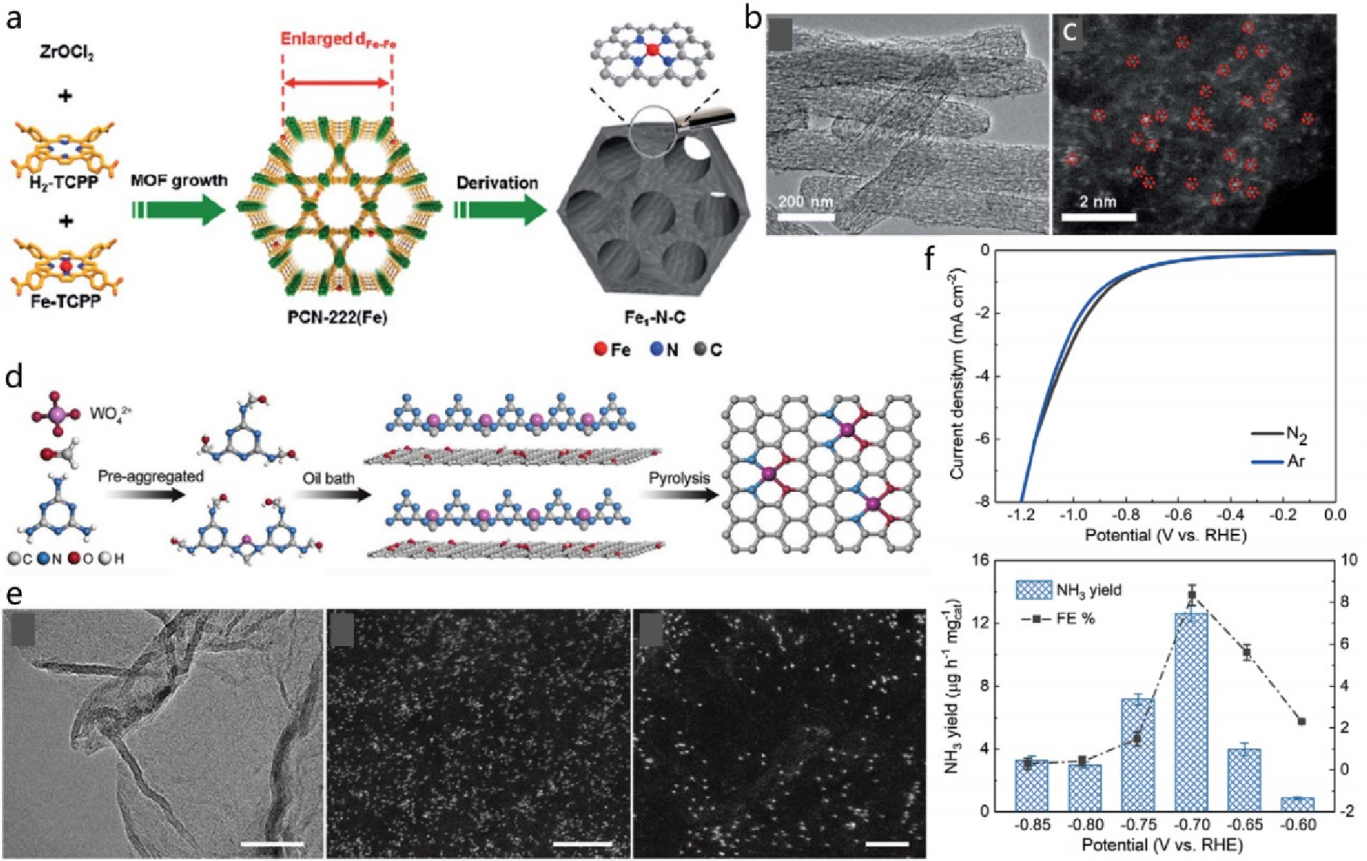
(a) The preparation of Fe-N-C SACs. (b) The TEM and (c) STEM images of the Fe-N-C SACs. Reprinted with permission from ref (405). Copyright 2019 Royal Society of Chemistry. (d) The fabrication of the W-N-C SACs. (e) The HRTEM and STEM images of the prepared W-N-C SACs. (f) Polarization curves of W-N-C in 0.5 M LiClO_4_ under Ar- and N_2_ and the corresponding NH_3_ yields and FEs at different potentials. Reprinted with permission from ref (406). Copyright 2021 Wiley-VCH.

Similarly to the previous case, the Fe-decorated porphyrinic MOF method was employed by using the H_2_-TCPP, Fe-TCPP, and ZrOCl_3_ agents.^405^ The prepared PCN-222 (Fe) MOF precursor could eventually evolve into hollow Fe-N-C tubes as shown in Figure 51a. The HRTEM image in Figure 51b displays the porous Fe-N-C tubes with an approximate radius of 200 nm. The STEM image points out the Fe SA sites in the Fe-N-C, revealing the successful approach to obtain the Fe SACs (Figure 51c). Hierarchically porous Fe-N-C showed a FE of 4.51% and a yield rate of 1.56 × 10^–11^ mol cm^–2^ s^–1^ at −0.05 V.^405^ Gu and co-workers found the W-based SACs could also exhibit attractive NRR catalytic activity.^406^ Specifically, W-metal salts with an O-group were applied to introduce W-O sites and block the aggregation of W atoms during the pyrolysis process (Figure 51d). The TEM image in Figure 51e shows the ultrathin 2D morphology of the synthesized W-N-C catalysts. Particularly, the STEM images displayed abundant W SAs on CN substrate. The difference of the current density based on NRR polarization curves (Figure 51f) under N_2_ and Ar indicated the activity of W-N-C SACs. As observed in Figure 51f, W-N-C SACs offered a superior yield rate of 12.62 μg_NH3_ h^–1^ mg_cat_^–1^ and a good FE of 8.35% at −0.70 V. Computational simulation results found that the special coordination bond W-NO/NC optimized the binding energies between the active sites and NRR intermediates.

Further, the mechanism of an electrochemical NRR to NH_3_ catalyzed by individual iron (Fe) atoms supported in a layered C_2_N material was elucidated by DFT-based calculations. The study revealed that Fe atoms situated within the pores and bonded to nitrogen (N) atoms of the C_2_N framework act as the active sites for the reaction. Interestingly, in the Fe-C_2_N catalyst, both distal and alternating pathways are favored over the enzymatic pathway. The onset potential for both distal and alternating pathways is as low as −0.7 V, in contrast to the enzymatic pathway with an onset potential of −1.2 V. This phenomenon was attributed to the formation of a *N-NH intermediate, representing the rate-determining step in the NRR. Consequently, computational predictions suggest that Fe-C_2_N has the potential to be a superior catalyst compared to Au-C_2_N due to its lower onset potential. However, despite this, the use of an Au-based catalyst still slightly outperforms the Fe-based one. This discrepancy arises from the competitive nature of the HER and the higher desorption energy of ammonia molecules in Fe-based catalysts.^407^

Li et al. introduced a novel strategy to enhance the performance of iron-based SACs for the NRR. The authors created atomically dispersed FeN_3_S_1_ sites anchored on a carbon matrix codoped with sulfur and nitrogen (NSC).^408^ This catalyst exhibited promising activity, yielding the NH_3_ production rate of 30.4 μg h^–1^ mg^–1^ cat and a FE of 21.9% in an H-cell setup. Additionally, it achieved even higher NH_3_ FE of approximately 10% at 10 mA cm^–2^ in a flow cell configuration. This remarkable performance was attributed to the introduction of sulfur-coordinated atoms into the isolated Fe-N moieties, which regulated the spin state of the central Fe atom, transitioning it from high-spin to medium-spin. In this medium-spin state, the Fe(I) species effectively activated the N≡N triple bond in the NRR process, serving as the catalytic active site. Theoretical calculations and *in situ* ATR-FTIR analysis further revealed that the isolated FeN_3_S_1_ sites reduced the energy barrier for the formation of *NNH intermediate and accelerated the hydrogenation reaction kinetics, thereby enhancing the electroreduction of N_2_ into NH_3_.

Guo and colleagues recently introduced a carbonless support using TiO_2_ to anchor Fe SAs. In their approach, Fe atoms were dispersed on TiO_2_ and employed as a Janus electrocatalyst, simultaneously facilitating both the nitrogen oxidation reaction (NOR) and the NRR in a two-electrode system. To mitigate the competitive reactions, namely the HER and the OER, the pulsed electrochemical catalysis (PE) was applied. This innovative technique resulted in a remarkable catalyst performance, yielding the NOR and the NRR reaction rates exceeding 7000 μmol h^–1^ g^–1^ cat. and 12 000 μmol h^–1^ g^–1^ cat. at the applied voltage of 3.5 V, respectively. The DFT calculations indicated that the presence of single-atom Fe stabilized oxygen vacancies reduced the energy barrier for the N≡N triple bond separation, thereby enhancing the N_2_ fixation. Additionally, the introduced PE method effectively increased the N_2_ supply by minimizing the competitive O_2_ and H_2_ clustering. It also hindered the formation of electrocatalytic byproducts by stabilizing the *OOH and the *H intermediates, thus promoting the nonelectrocatalytic process of the N_2_ activation.^409^

Theoretical studies have suggested the potential cooperation of multiple atoms within small clusters as the NRR catalysts.^410^ However, the distance between these single-atom catalytic centers, even when just a few nanometers apart, is still too large to achieve effective cooperative functioning in the NRR. Therefore, it is important to reduce the interatomic distance to approximately 0.1–0.2 nm, corresponding to the length of the N≡N triple bond.^411^ On the other hand, certain catalysts exhibit a quasi-semiconducting surface due to a metal/nonmetal coordination manner (e.g., Fe-N_*x*_ or Co-N_*x*_), enabling an efficient electron transfer for the multielectron NRR process. For example, Wang and colleagues demonstrated the utilization of ordered subnano spaces within the surface cavities of g-C_3_N_4_ to host multiple Fe and Cu atoms, thus forming subnano reactors.^386^ The strong coordination among the confined Fe and Cu atoms within these reactors resulted in significant synergy between the two species, markedly enhancing the NRR performance. It led to considerably higher ammonia yield and efficiency compared to monometallic counterparts. In particular, the CNT@C_3_N_4_-Fe&Cu catalysts exhibited the highest ammonia yield of NH_3_ of 9.86 μg mg^–1^ h^–1^ and the highest FE of 34%, surpassing the performance of the Fe and Cu single-metal counterparts. First-principle DFT calculations revealed that the coordination between Cu and Fe within these subnano reactors simultaneously accelerated the N_2_ adsorption and optimized the reaction pathway, substantially reducing the energy barrier and greatly facilitating the NRR process.^386^

Furthermore, Zhang and colleagues explored the synergistic interaction among the neighboring Fe atoms in multiatomic Fe clusters for the NRR. They used MoS_2_ as the supporting material for various Fe_*x*_ clusters. Through theoretical computations employing spin-polarized DFT coupled with a hydrogen electrode (CHE) model, they found that Fe_2_/MoS_2_ exhibited promising NRR catalytic activity in comparison to its counterparts, Fe/MoS_2_ and Fe_3_/MoS_2_ systems. These SAC clusters displayed a preference for the enzymatic mechanism toward the NRR, exhibiting a remarkably low overpotential of 0.21 V and a reasonable adsorption energy of −0.65 eV. The presence of MoS_2_ lead to a synergistic effect, depleting the electron density on the Fe_2_ cluster. This effect created a vacant orbital, acting as a Lewis acid active site for stable N_2_ adsorption. Simultaneously, electronic back-donation from the Fe_2_ cluster to N_2_ facilitated the activation of the N≡N bond, overall enhancing the NRR process.^412^

## 8 SUMMARY AND OUTLOOK

Developing highly efficient hybrid catalysts for sustainable energy applications involves a meticulous bottom-up strategy, where the features of inorganic and metallo-organic chemistry play a key role. This approach enables customization of the catalysts’ physicochemical and catalytic properties, making them suitable for specific chemical transformations. Such transformations are critical in various electrochemical processes, including energy storage solutions and electrochemical reactions such as ECO2RR, ORR, HER, OER, and NRR. Among the most promising candidates for such reactions are new generation electrocatalysts, especially those based on transition metals (e.g., Co, Cu, Ni, Fe, Ti, etc.) configured as SAs within a supportive matrix. Over the past decade, single-atom catalysis has emerged as a groundbreaking field in the fields of heterogeneous photo- and electrocatalysis. Transition to single-atom configurations dramatically transforms the physicochemical properties of metals, enhancing their catalytic activity through improved charge separation/transfer efficiency and an increased count of catalytic reaction sites. These benefits collectively enhance the catalytic performance. Furthermore, the synthesis of SACs allows precise surface modifications, enabling tailored adsorption of reactant molecules, which in turn elevates the selectivity and the catalytic efficiency.

### 8.1 SACs in Electrochemical Storage

SACs can be applied in several branches and chemistries in the field of electrochemical energy storage, including (so far) lithium-ion, metal-air, metal-CO_2_, metal-sulfur batteries, and supercapacitors, leveraging the full atom economy offered by these systems and the newly developed electronic phenomena at the atomic interfaces, creating previously unexplored pathways for effective interaction with the reactive species. Earth-abundant SACs have demonstrated promising results for the replacement of noble and critical metal-based systems. Indicatively, molybdenum nitrides and sulfides have delivered high capacities in Li-air batteries.^85−87^ Bimetallic Zn-Fe SACs coordinated in N-doped carbons were more effective for the OER in ZABs than RuO_2_ and IrO_2_-based catalysts.^110^ In general, SAs@N-doped carbon hosts are currently the most widely used and promising ORR nonprecious metal catalysts, required during the discharging of the metal-air batteries. The most studied SACs for the ORR are the Fe and Co@N-doped carbon hosts, with the Fe SAC-based ZABs demonstrating relatively higher power density than the others. In metal-sulfur batteries, SACs efficiently promote the overall performance because of the improved adsorption and high activity in metal-sulfur species conversion. The development of SACs using nonprecious metals is a research field of profound interest, expected to evolve dramatically in the years to come due to the unique properties, cost effectiveness, eco-friendliness, and availability, but also due to the necessity for the identification of greener technologies for energy storage. Nonprecious metal SACs might offer a green avenue for future sustainable electrocatalysis for energy transformation and storage. However, significant effort and time investment will be required in the field, since the development of (i) well-controlled coordination environments, going beyond the N4-M motives, (ii) methodologies for the preparation of SACs on large-scale, (iii) high metal loading, in order to achieve better productivities in catalysis and energy densities in energy storage, (iv) enabling also metal–metal cooperativity (of homo or hetero bimetallic nature) and metal–support cooperative synergies, and (v) deep understanding of the mechanisms behind SACs is required to reach the goals for high practical applicability for energy storage from renewable resources and transition to an energy self-sustainable society. In several battery chemistries, multiple catalytic reactions take place on the same electrode. For instance, the ORR and the OER transpire during the discharge and charge cycles, respectively, on the cathode of metal-air batteries. Thus, the construction of strategically engineered bimetallic or multimetallic SAC centers emerges as a fundamental prerequisite for developing bifunctional catalysts essential for next-generation batteries.^413,414^ Methodologies aimed at achieving multifunctionality by modifying the coordination environment are also pivotal.^375,415^ For example, combined configurations such as M-N4 and M-N3X (where X = S, P, O, F) can yield distinct catalytic activities and specificities, even when utilizing identical metals. The development of corrosion-resistant carbon-based hosts with SACs is also critical for electrochemical energy storage technologies,^416^ particularly in those cases where highly reactive radicals and superoxides are involved in the cathodic and anodic reactions. Carbon corrosion leads to severe changes of the coordination environment and subsequently to poor catalytic activity, alongside instability and limited cycle-life. In supercapacitors, the exploration of SACs has been comparatively limited, as their operation does not involve catalytic processes and primarily relies on the sorption of ionic species and/or electrochemically induced electron transfers in the electrode materials. However, the development of methods and substrates for SACs that enable higher single atom loadings could profoundly impact supercapacitors. This would be achieved by harnessing redox-active single atomic centers to enhance pseudocapacitance. Additionally, single atomic centers at catalytic concentrations may become feasible by integrating redox-active molecular species in the electrolytes or electrodes, whose redox transformations could be catalyzed by the presence of SACs embedded in separation membranes or on the bulk of the electrodes. SAC-mediated improved redox transformation kinetics with high reversibility could dramatically contribute to bypassing the current limitations of supercapacitors in the energy content, while keeping ultrafast charging/discharging rates.

### 8.2 SACs in Electrochemical Reduction of CO_2_

The electronic structure of the single-atom support structure is not well-defined. Despite progress in this area, there is room for improvement in developing better-quality carbon-based materials, with much to learn about the structure–activity relationships.

Despite extensive studies and successful cases of SACs in ECO2RR, the method of pyrolyzing MOF-based templates is still limited by the expensive organic precursors deployed during the synthesis. The direct use of high surface area carbon and other readily available conductive materials as supports to incorporate metal precursors thus offers a promising strategy toward a scalable synthesis of SACs. In addition, a universal method and protocol ought to be developed in a controlled manner.

Most SACs easily generate 2e^–^ transfer products in ECO2RR, and the utmost customary product is carbon monoxide. This is due to the intrinsic properties of the single-atom moiety exposing a single active site. There is a common consensus that SACs are unable to produce C^2+^ products because generating those products requires the dimerization of two or more C-based intermediates such as *CO, *CHO, *COH, or *CHx. In SACs, however, the metal atoms exist as atomically dispersed sites, and hence the interaction of two or more C1 intermediates is not plausible. Thus, the usage of SACs for multiple carbon products is still a challenging proposition. Recent introduction of bifunctional metal SACs (single-atom alloy) could offer opportunities for this target.

Most transition metal SACs that could be applied in ECO2RR are among metals such as Fe, Ni, and Co, using N to coordinate with them, simulating the metalloporphyrin-like M-N_4_ coordination. Thus, further research efforts ought to explore more candidate metals and related moieties, including O, S, or P coordination. Generally, the heteroatoms can tailor carbon materials’ electron distribution and electrochemical properties, and the precise control in local architecture formation is crucial to tailor the ECO2RR activity.

Some *in situ* or *operando* techniques could be introduced to monitor the reaction pathway of SACs catalyst evolution and reaction mechanism so that while evaluating the SACs catalyst under the working condition, the coordination of SACs could be altered.^417−419^ Thus, dynamically formed nanoparticles could significantly influence product distribution. Spent catalyst investigations will also provide some insights, which should also be considered as significant research concerns. Researchers often face the complexity and true usability of such materials. In an ideal scenario, to ensure that the electrochemical experiment provides an interpretable and scientifically meaningful understanding, *in situ* or *operando* techniques in GDE configuration ought to be developed. This has rendered it difficult to estimate their performance on industrial scale.

The electrochemical conversion of carbon dioxide into fuels has intrigued electrochemists for many decades and is currently undergoing a significant renaissance, although CO_2_ electrolysis is still far from a mature technology. The electrolysis of CO_2_ using SACs has remained in laboratory curiosity due to many challenges that prevented an upscale attainment of the technology at a meaningful level. Significant lingering hurdles are related to energy efficiency, reaction selectivity, and the overall conversion needing to be improved if electrochemical reduction of CO_2_ will become a viable option for storing renewable electricity. In the single atom-based catalysis field, the critical question centers on how to develop an efficient and robust catalyst candidate for the scale-up of the electrochemical process.

### 8.3 SACs in ORR

In summary, SACs represent an emerging class of low-cost, highly efficient electrocatalysts for the ORR. However, SACs still require further improvement to meet the industrial demands, such as long durability, low cost, and simple procedure of manufacture. Furthermore, especially for H_2_O_2_ production through ORR, high electrolysis efficiency with low energy input is required.

Most transition metal single-atom structures are inspired by metal–ligands-based heterocycle structures. Instead of always using M-N_4_ coordination, in the future, exploring more local structures as the host to anchor the single metal site is still at the heart of the research. Furthermore, the structure–property relationship of catalysts still does not follow a clear principle compared with homogeneous molecular catalysis.

Since the introduction of SACs was initially aimed at improving the activity, the reaction pathway could also be tuned when turning the nanostructure into an atomic dispersion in a matrix. SACs exhibit unique features in adjusting the reaction pathway. For this purpose, more efforts are needed to explore unique local configurations to tune the reaction selectivity and thus fulfill the diversity application.

Special organic and inorganic complexes can anchor metal atoms to produce atomically dispersed metal sites on carbon frameworks. Therefore, most reported SACs are synthesized based on imidazole frameworks (ZIFs) or MOF-based structures, limiting the demand on a practical scale. In the future, cheap metal nodes and a simple synthesis approach must be developed.

Some SACs show both activities in electrochemical CO_2_ reduction and ORR, but the exact mechanism for the individual active site is still unclear. Thus, the future research efforts could focus on developing catalyst design protocols. Therefore, it will represent the advanced design for a particular function.

### 8.4 SACs in HER, OER, and NRR Reactions

SACs based on transition metals have shown remarkable activity toward HER and OER in a wide range of pH. However, the catalytic performance is usually dependent on the metal instinct properties, its coordination environment, and the substrate, which could be determined by the binding strength of *H and thereby the catalytic performance for HER. Since the HER and HOR have been well-studied half-cell reactions in water electrolysis technology, exploring efficient SACs with long-term stability is still a major challenge, especially for the OER, which processes multiple electron transfer steps. First, HER and OER appear in all electrochemical reactions within the aqueous solution. Thus, the strategy for using SACs ought to aim for tuning this reaction accordingly by taking advantage of the synergistic effects between single-atom metal active sites and the substrate. For instance, when they appear as a side reaction, tuning the coordination environment to increase the overpotential could inhibit the reaction rate, and triggering the metal center bonded with oxygen could lower the adsorption barrier of the oxygenated intermediates, thus benefiting OER.

To date, most single-atom catalysts for NRR are based on the M-N-C structure. As reported, the elements Bi, Sn, and Sb show the potential for catalyzing NRR. Although novel SACs have been discovered continuously for NRR in the latest decade, the yield rate of ammonia in electrochemical NRR remains extremely low, hindering their practical application. Thus, the research of NRR is still in its early stage with many open questions. First, the activation of inert N_2_ molecules is still the main theme. The active sites on the SACs must be further understood to provide insight for highly efficient NRR catalysts design. Second, the biggest challenge for NRR is how to control the competing reaction, HER, which features a much lower activation barrier. Several strategies could be anticipated with the inspiration from the catalyst design for ECO2RR. For instance, (i) developing GDEs to enhance the efficiency, (ii) surface hydrophilicity/hydrophobicity engineering to suppress HER, and (iii) modification of metal centers in SACs to obtain high overpotentials for HER. Lastly, the stability of single-atom catalysts requires further optimization. Precise control of synthesis coordinated moiety reconstruction, and the local environment are needed to improve the stability of SACs for NRR.

Despite the fact that research into SACs as a new generation of heterogeneous catalysts is still in a rather initial stage, their inherent advantages position them as the most promising solutions for efficient, renewable, and sustainable energy conversion technologies. The compelling need for new, scalable, environmentally friendly, and cost-effective SACs underscores their potential to revolutionize access to renewable energy sources, aligning with the global pursuit of sustainability. However, three main challenges restricting the use of SACs in real applications, and ultimately attaining this target, have yet to be addressed:

Challenge I: Developing a versatile and scalable approach for the synthesis of SACs for sustainable energy applications requires overcoming significant obstacles. These include (i) fine-tuning the electronic properties and coordination environments of SACs to optimize their performance; (ii) achieving an optimal but high loading of SACs on supports to prevent their agglomeration, which is a common issue due to the high surface energy of the catalysts; and (iii) ensuring active interaction between the SACs and their supports. The next generation of heterogeneous SACs must be robust, easily customizable in terms of the transition metals used and their electronic properties, tailored to meet the specific needs of target reactions (high activity/productivity, selectivity, yield), capable of producing a wide range of products, and fully recyclable at a reasonable cost.

Challenge II: Scaling up the SACs systems for practical applications represents another significant issue. Currently, the most effective catalysts often consist of complex multicomponent hybrid structures that require intricate chemical methods for their synthesis, leading to high production costs that are economically unsustainable. Therefore, establishing a technique enabling the widespread manufacturing of SACs will require the invention of procedures that minimize the use of resources and costs, while also ensuring consistent replicability to allow their adoption in industrial environments. This issue has, however, largely remained unsolved.

Challenge III: Acquiring a deep understanding of the structural and electronic properties of SACs and the mechanisms of their reactions in sustainable energy applications is crucial. Presently, knowledge about the geometric and electronic structures of metal atomic sites within supports, as well as how these features correlate with the catalysts’ performance and the detailed reaction mechanisms at the atomic level, remains severely limited. This gap in knowledge largely depends on sophisticated techniques often requiring synchrotron-based facilities, which are not widely accessible. Nevertheless, such insights are essential for the rational design of highly efficient SAC systems for sustainable energy conversion and storage. We therefore need to focus on other *in situ*, *operando* techniques to understand the reaction mechanisms and thus the best performing SACs.

Several innovative strategies have been proposed to optimize the use of SACs based on Earth-abundant metals for sustainable energy and development, as highlighted in this review. These include, for instance, integrating different SACs into one hybrid system. Additionally, significant advancements can be achieved through leveraging rapidly evolving computational methods. These methods integrate machine learning, artificial intelligence, and DFT calculations for designing SACs that are potentially more active and selective. Furthermore, these techniques offer a comprehensive analysis of the mechanistic aspects of SACs in their application to specific reactions.

**Figure 52:**
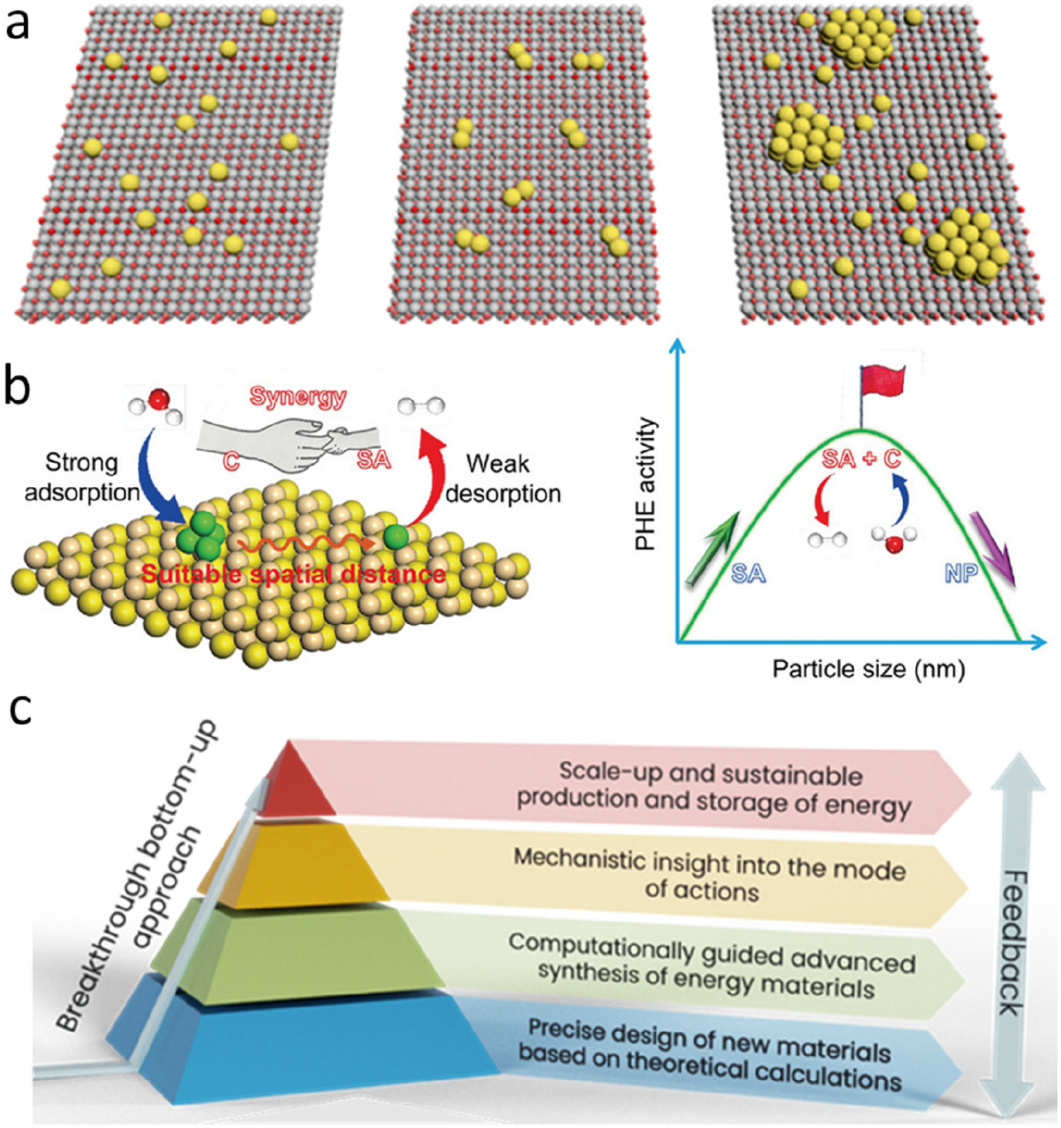
(a) Schematic representation of the distribution of metal species across (from left to right) single-atom catalysts, dual-atom site catalysts (DASCs), and nano-single-atom site catalysts (NSASCs). Reprinted with permission from ref (420). Copyright 2022 American Chemical Society. (b) Sketches illustrating a potential synergistic mechanism between single atoms and clusters that enhances the photocatalytic hydrogen evolution (PHE) activity. The schemes demonstrate the importance of both the heterogeneity and close spatial arrangement of binary active sites (SAs and NPs), each performing distinct roles in the stages of multistep photocatalytic reactions. Reprinted with permission from ref (421). Copyright 2023 Wiley-VCH. (c) An overview diagram showcasing the methodology, highlighting the critical steps in the hierarchical design process for new materials incorporating SACs.

Bimetallic SACs or dual-atom-site catalysts (DASCs) represent an advancement in this field, offering features that can extend beyond the capabilities of monometallic SACs. The DASCs are catalysts characterized by either having two metal atoms/ions at isolated active sites or featuring two distinct metal single-atom sites that exhibit synergistic effects in catalysis. The reported DASCs can be classified into two types: (i) homonuclear DASCs, where the active sites consist of identical metal atoms and (ii) heteronuclear DASCs, where the active sites are composed of two different metal atoms.^420^ Similarly, there has been an increasing number of reports on catalysts that incorporate both nanoparticle (NP) and SA sites.^421,422^ These forms are depicted schematically in Figure 52a. These catalysts are designed to leverage the combined benefits of SACs and nanocatalysts, while also addressing their individual drawbacks. The nanoparticle sites can participate in the catalytic process either directly, by interacting with the reactants and intermediates, or indirectly, by influencing another site or enhancing the reaction rate through structural effects. Nevertheless, in both cases (i.e., DASCs and NP/SACs) the catalytic activity is often higher than that of monometallic counterparts due to the presence of two different catalytic sites and/or metal atoms, which can lead to a more favorable electronic structure for catalytic reactions. These combinations can create various distinct active sites that can preferentially catalyze specific pathways, thus reducing the production of unwanted byproducts. Stability under reaction conditions can also be generally enhanced in these dual systems, since the interaction between two metal atoms/NPs can increase the resistance to common deactivation processes such as sintering and aggregation, thereby extending the catalysts’ operational life. The electronic properties of dual SACs’s systems are tunable through variations in the composition and arrangement of the metal atoms. This allows for the optimization of the catalysts for specific reactions by altering their electronic interactions with reactants. This can result from electronic modifications, geometric effects, or both, leading to improved catalytic efficiency and selectivity (see Figure 52 b).^421,423−425^

Currently, the development of new materials, including those based on SACs for energy harvesting and storage, is predominantly empirical. This traditional method often entails long-term and economically demanding optimization processes that still encounter significant challenges concerning low performance, selectivity, and stability, while also facing issues with sustainability. To address these challenges, a new generation of materials for sustainable production and storage of energy based on earth-abundant transition metals can be developed with the help of computational guidance. The integration of computational design, machine learning (ML), and artificial intelligence (AI) represents a revolutionary approach to this end.^426−431^ These advanced technologies offer novel approaches to addressing some of the key challenges in catalysis, such as enhancing activity, selectivity, and stability, while also reducing the costs. Moreover, by harnessing these technologies, we can predict and define the tailored properties of the new energy-related materials with a much higher degree of precision. ML algorithms can rapidly predict the properties of a vast array of materials, significantly speeding up the discovery process. By learning from existing data sets of material properties and catalytic performances, these algorithms can identify promising candidates for SACs without the need for extensive experimental testing. ML models can also predict how different materials will degrade over time, allowing the selection of materials that offer both high performance and durability. Additionally, AI can possibly contribute in analyzing complex relationships between the structure of catalytic materials and their activity. AI can predict how modifications to the local environment of these single atoms, such as changes in coordination number, electronic structure, and atomic spacing, will affect catalytic activity and stability. This level of precision is difficult to achieve through traditional experimental methods alone. Finally, ML and AI can be integrated with computational chemistry and materials science methods, such as density functional theory (DFT), to provide a deeper understanding of the electronic and atomic-level interactions in catalytic materials. This pioneering strategy through a hierarchical synergy (Figure 52c) can lead to the design of materials with highly optimized properties for specific catalytic reactions. Ultimately, this intelligent design approach promises to unlock a new field of possibilities in the creation of robust, selective, and sustainable materials for green energy applications.

## LIST OF ABBREVIATIONS

AEMFC: anion exchange membrane fuel cell
ATR-IR: attenuated total reflection infrared spectroscopy
CC: carbon cloth
CE: Coulombic efficiency
CF: carbon fiber
CMP: conjugated micro-/mesoporous polymer
CNTs: carbon nanotubes
CO2ER: CO_2_ evolution reaction
COFs: covalent organic frameworks
CO2RR: CO_2_ reduction reaction
CO-TPD: CO-temperature-programmed desorption
CRMs: critical raw materials
CV: cyclic voltammetry
DFT: density functional theory
ECO2RR: electrochemical CO_2_ reduction reaction
EDLC: electrical double-layer capacitance
EDS (EDX): energy dispersive X-ray spectroscopy
EELS: electron energy loss spectroscopy
EES: electrochemical energy storage
EXAFS: extended X-ray absorption fine structure
FE: Faradaic efficiency
FESEM: field emission scanning electron microscopy
FFTI: fast Fourier transformed image
FT-EXAFS: Fourier transformed-extended X-ray absorption fine structure
GDE: gas diffusion electrode
GO: graphene oxide
HAADF-STEM: high angle annular dark field scanning transmission electron microscopy
HC: hollow carbon
HER: hydrogen evolution reaction
HR-TEM: high resolution transmission electron microscopy
HR-XPS: high resolution X-ray photoelectron spectroscopy
KSBs: potassium sulfur batteries
LED: light emitting diode
LFP: lithium iron phosphate
LIBs: lithium-ion batteries
LSBs: lithium sulfur batteries
LSV: linear sweep voltammetry
M: metal
MIHCs: metal-ion hybrid capacitors
MOF: metal organic framework
MSBs: metal sulfur batteries
NaSBs: sodium sulfur batteries
NC: nitrogen doped carbon
NCA: nitrogen doped carbon aerogel
NCFs: nitrogen doped carbon nanofibers
NG: nitrogen doped graphene
NHE: normal hydrogen electrode
NRR: nitrogen reduction reaction
OER: oxygen evolution reaction
ORR: oxygen reduction reaction
PC: porous carbon
PECT: proton–electron coupling transfer
PEMFC: proton exchange membrane fuel cell
PGMs: platinum group metals
pnN: pyridinic nitrogen
prN: pyrrolic nitrogen
qN: quaternary nitrogen
rGO: reduced graphene oxide
RHE: reversible hydrogen electrode
RT-NaSBs: room temperature sodium sulfur batteries
SA: single atom
SAs: single atoms
SAC: single atom catalyst
SACs: single atom catalysts
SC: supercapacitor
SCs: supercapacitors
SCE: saturated calomel electrode
SEM: scanning electron microscopy
SHE: standard hydrogen electrode
SIHCs: sodium-ion hybrid capacitors
STEM: scanning transmission electron microscopy
TEM: transmission electron microscopy
TM: transition metal
WT-EXAFS: wavelet analysis of extended X-ray absorption fine structure
XANES: X-ray absorption near edge structure
XAS: X-ray absorption spectroscopy
XPS: X-ray photoelectron spectroscopy
XRD: X-ray diffraction analysis
ZABs: zinc air batteries
ZIF: zeolitic imidazolate framework
